# Supplementary material for: Stereoselective assembly of C-oligosaccharides via modular difunctionalization of glycals
Source: Nat Commun. 2024 Mar 30;15:2794. doi: 10.1038/s41467-024-47060-7 (PMC10981691; doi:10.1038/s41467-024-47060-7)
Supplement: Supplementary file 1 — Supplementary Information [file 41467_2024_47060_MOESM1_ESM.pdf]

Supplementary Information

Stereoselective Assembly of *C*-oligosaccharides via Modular Difunctionalization of Glycals.

Ya-Nan Ding,[1] Mei-Ze Xu,[1] Yan-Chong Huang,[1] Lutz Ackermann*[2], Xiangtao Kong,*[3] Xue-Yuan Liu*[1] and Yong-Min Liang*[1]

[1]State Key Laboratory of Functional Organic Molecular Chemistry, Lanzhou University, Lanzhou University 73000, China.

[2]Institut für Organische und Biomolekulare Chemie and Wöhler-Research Institute for Sustainable Chemistry (WISCh), Georg-August-Universität, Tammannstrasse 2, 37077 Göttingen, Germany.

[3]Henan Key Laboratory of New Optoelectronic Functional Materials, College of Chemistry and Chemical Engineering, Anyang Normal University, Anyang 455000, People's Republic of China.

Table of Contents

I. Supplementary Methods 3

1. General Remarks 3
2. General Procedure for the Preparation of Starting Materials. 4
3. General Procedure. 14
4. Characterization Data 18
5. Computational Details 45

II. Supplementary Figures 50

III. Supplementary References 123

I. Supplementary Methods

1. General Remarks

Unless otherwise noted, all of these reactions were carried out under an argon atmosphere. Solvent was freshly distilled prior to use unless otherwise noted. For column chromatography, silica gel (200-300 mesh) was employed. Analytical TLC was performed with silica gel GF254 plates. Room temperature = r.t.

Materials. Commercial reagents were purchased from Acros, Accela, Adamas, Alfa, Ark, Leyan or TCI and used as received with the following exceptions.

Instrumentation. Deuterated solvents were purchased from Cambridge Isotope Laboratories. ^{1}H NMR spectra were recorded on Bruker AANCE III 400, 600 with a 400 MHz and 600 MHz frequencies, and ^{13}C NMR spectra were recorded on Bruker AVANCE III 400 and 600 with 101 MHz and 125 MHz frequencies. ^{19}F NMR spectra were recorded on a Bruker AVANCE III 400 spectrometer with a ^{19}F operating frequency of 376 MHz. Chemical shifts (δ) were reported in ppm relative to the residual solvent signal (TMS δ = 0 for ^{1}H NMR and $CDCl_3$ δ = 77.0 for ^{13}C NMR). Multiplicities are given as s (singlet), d (doublet), t (triplet), dd (doublet of doublets), td (triplet of doublets) or m (multiplet). HRMS obtained using a Q-TOF instrument equipped with an ESI source. Optical rotations were measured on a Rudolph Autoplo IV polarimeter.

2. General Procedure for the Preparation of Starting Materials.

Preparation of sugar **1a-1h** based on literature procedures：

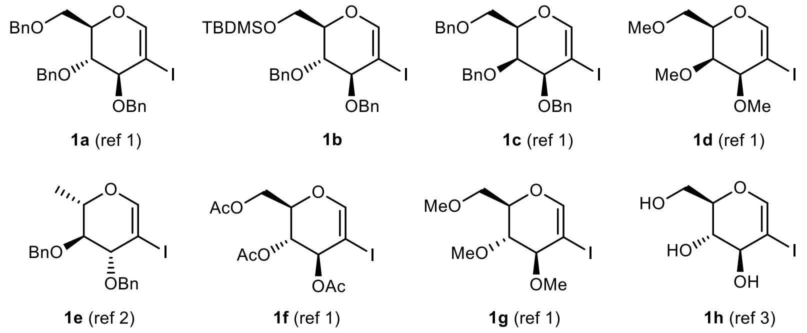

Supplementary Figure 1. Scope of 2-iodoglycals

(((2R,3R,4S)-3,4-bis(benzyloxy)-5-iodo-3,4-dihydro-2H-pyran-2-yl)methoxy)(tert-butyl)dimethylsilane (1b)

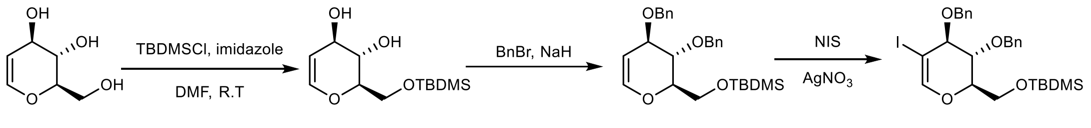

1b-1 **1b-2** **1b-3** **1b**

Step 1: D-Glucal (**1b-1**) (1.0 g, 6.84 mmol), tertbutyldimethylsilyl chloride (1.13 g, 7.52 mmol), and imidazole (930 mg, 13.68 mmol) were stirred in anhydrous dimethylformamide (DMF, 10 mL) at room temperature for 12 h. After completion of the reaction as monitored by TLC, DMF was evaporated under reduced pressure and the resulting mixture was diluted with ethyl acetate EtOAc) and washed with saturated $NaHCO_3$ (aq) and brine. The combined organic layer was dried over anhydrous Na_2SO_4. The filtrate was condensed under reduced pressure and purified by silica gel lash column chromatography (EtOAc: Petroleum ether = 1:1, v/v) to provide the desired product **1b-2** (1.35 g, 76%) as an amorphous white solid.

Step 2: To a solution of compound **1b-2** (1.21 g, 4.647 mmol) in anhydrous THF (25 mL) stirred at 0°C was added sodium hydride (690 mg in 60% mineral oil, 17.19 mmol) over a 15 min period. The reaction mixture was stirred for 30 min and warmed up to room temperature. Then, benzyl bromide (1.77 mL, 14.87 mmol) and *tetra*-n-butylammonium iodide (TBAI, 170 mg, 0.4647 mmol) were sequentially added in dropwise. The mixture was stirred at room temperature overnight under a nitrogen atmosphere. After completion of the reaction as monitored by TLC, the resultant was diluted with EtOAc and washed twice with brine. The combined organic layer was dried over anhydrous Na_2SO_4. The filtrate was condensed under reduced pressure

and purified by silica gel flash column chromatography (EtOAc: Petroleum ether = 1:3, v/v) to provide the desired products **1b-3** (1.60 g, 78%) as an amorphous white solid.

Step 3: To a flame-dried 25-mL reaction tube were added the corresponding *O*-protected glycal (2.64 mmol, 1.0 equiv.), N-iodosuccinimide (713 mg, 3.17 mmol, 1.2 equiv.), $AgNO_3$ (90 mg, 0.53 mmol, 20 mol%) and dry MeCN (12 mL) (**Note: Non-HPLC grade acetonitrile gave no product for this reaction.**). The mixture was stirred at 70°C for 1 h and then filtered through a plug of silica, which was thoroughly washed with EtOAc in sequence. The liquid was then evaporated under reduced pressure and the residue was purified by flash column chromatography to provide the desired products **1b** (1.05 g, 70%) as an amorphous white solid. ^{1}H NMR (600 MHz, Chloroform-d) δ 7.45 (d, J = 7.2 Hz, 2H), 7.41 – 7.35 (m, 8H), 6.75 (s, 1H), 4.76 (q, J = 12.3, 11.9 Hz, 3H), 4.71 (d, J = 10.9 Hz, 1H), 4.14 (s, 2H), 4.07 (t, J = 6.0 Hz, 1H), 3.97 (dd, J = 11.3, 3.5 Hz, 1H), 3.92 (d, J = 10.9 Hz, 1H), 0.93 (s, 9H), 0.11 – 0.08 (m, 6H). ^{13}C NMR (151 MHz, Chloroform-d) δ 148.5, 137.8, 137.7, 128.5, 128.3, 128.2, 127.9, 127.9, 127.8, 79.2, 78.2, 73.9, 73.4, 72.5, 70.4, 61.0, 25.8, 18.2, -5.3, -5.4.

HRMS (ESI) m/z: [M + Na]$^+$ Calcd for $C_{26}H_{35}IO_4SiNa$ 589.1242 found 589.1247.

Preparation of chloride donors **2a**-**2l** based on literature procedures：

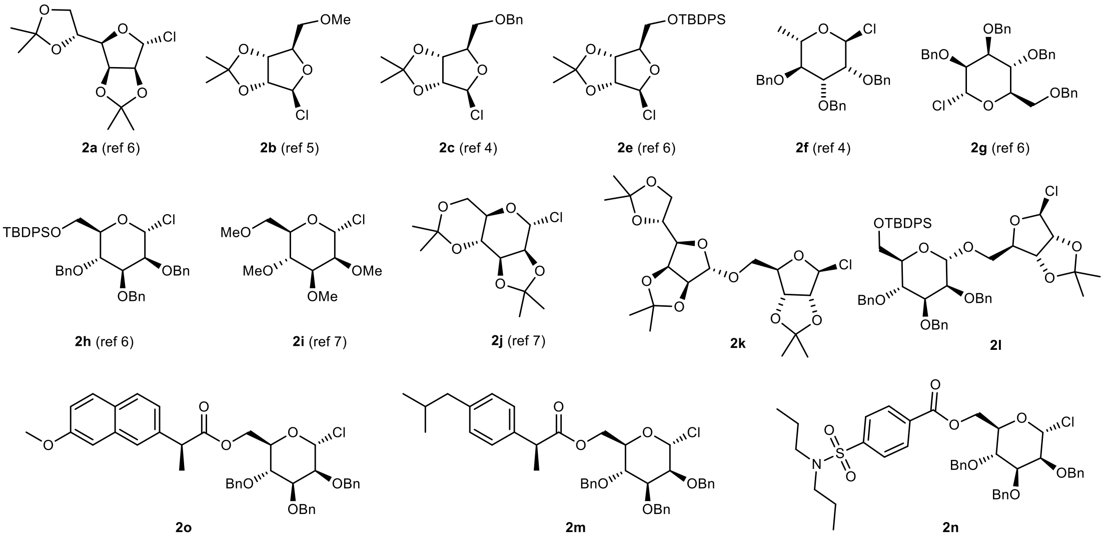

Supplementary Figure 2. Scope of glycosyl chloride donors

(3aR,4S,6R,6aR)-4-chloro-6-((((3aS,4S,6R,6aS)-6-((R)-2,2-dimethyl-1,3-dioxolan-4-yl)-2,2-dimethyltetrahydrofuro[3,4-d][1,3]dioxol-4-yl)oxy)methyl)-2,2-dimethyltetrahydro-furo[3,4-d][1,3]dioxole (2k).

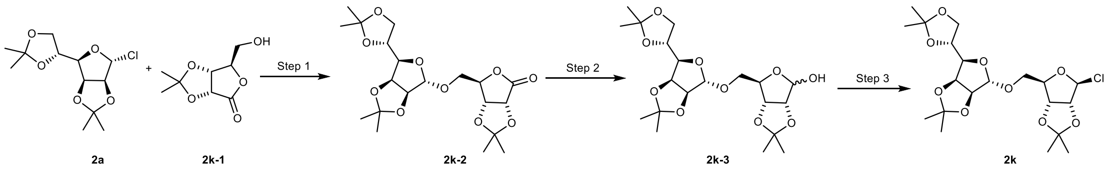

Step 1: **2k-1** (2 mmol, 1 equiv), $Pd(OAc)_2$ (0.1 mmol, 0.05 equiv), and glycosyl chloride **2a** (3 mmol, 1.5 equiv) in chloroform in a 50 mL glass vial (purged with Ar, sealed with PTFE cap) was heated at 60°C for 24 h. The reaction mixture was cooled to r.t. and concentrated in vacuo. The resulting residue was purified by silica gel flash chromatography to give the *O*-glycosylation product (**2k-2)** as an amorphous white solid[8].

Step 2: **2k-2** (1 mmol) was dissolved in DCM (5 ml) and cooled to -78°C. Diisobutylaluminum hydride (1.2 ml, 1.2 mmol, 1M in hexanes) was added slowly over 10-15 minutes and the reaction mixture kept at - 78°C for 1h. Methanol (2 ml) was added dropwise to quench the reaction and the solution allowed to warm to room temperature. The solution was diluted with DCM and washed with brine. The solution was dried over anhydrous Na_2SO_4, filtered and concentrated under reduced pressure. The product was purified by flash chromatography to give compound **2k-3**.

Step 3: DMF (0.31 ml, 4.0 mmol) was added to 2,4,6-trichloro-[1,3,5]-triazine (TCT) (0.1 g, 1.1 mmol) and the solution was stirred at room temperature for 15 minutes under N_2. Compound **2k-3**. (0.5 mmol) in DCE was added to the TCT-DMF suspension, the followed by addition of DBU (0.16 ml, 1.1 mmol). The mixture was stirred at 60°C and monitored by TLC. The temperature was brought to room temperature and Et_2O was added to the mixture for the precipitation of cyanuric salt. Cyanuric salt was removed by filtration and the filtrate was concentrated in vacuo. The resulting residue was purified by silica gel flash chromatography to give compound **2k**. ^{1}H NMR (600 MHz, Chloroform-*d*) δ 6.15 (s, 1H), 5.03 – 4.98 (m, 2H), 4.83 – 4.78 (m, 2H), 4.65 (d, *J* = 5.9 Hz, 1H), 4.47 (t, *J* = 7.2 Hz, 1H), 4.42 – 4.36 (m, 1H), 4.10 (dd, *J* = 8.6, 6.5 Hz, 1H), 4.04 (dd, *J* = 8.6, 4.6 Hz, 1H), 3.97 (dd, *J* = 7.8, 3.5 Hz, 1H), 3.87 (dd, *J* = 10.4, 6.4 Hz, 1H), 3.63 (dd, *J* = 10.4, 8.1 Hz, 1H), 1.48 (s, 3H), 1.46 (s, 3H), 1.44 (s, 3H), 1.38 (s, 3H), 1.34 (s, 3H), 1.32 (s, 3H). ^{13}C NMR (151 MHz, Chloroform-*d*) δ 113.4, 112.6, 109.2, 106.9, 98.3, 89.5, 88.1, 84.9, 81.5, 80.6, 79.4, 73.0, 67.1, 66.9, 26.8, 26.5, 25.8, 25.3, 25.2, 24.5.

HRMS (ESI) m/z: $[M + Na]^+$ Calcd for $C_{20}H_{31}ClO_9Na$ 473.1549; Found 473.1538

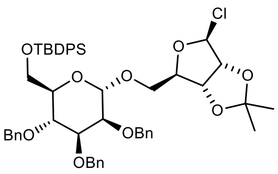

2l

According to the general procedure (**2k**): ^{1}H NMR (400 MHz, Chloroform-*d*) δ 7.72 (dd, *J* = 15.6, 7.5 Hz, 4H), 7.43 – 7.26 (m, 17H), 7.25 – 7.22 (m, 2H), 7.21 – 7.15 (m, 2H), 6.08 (s, 1H), 4.93 (d, *J* = 10.7 Hz, 1H), 4.87 (d, *J* = 5.5 Hz, 2H), 4.80 (d, *J* = 12.4 Hz, 1H), 4.74 – 4.66 (m, 4H), 4.61 (d, *J* = 10.7 Hz, 1H), 4.41 (t, *J* = 5.7 Hz, 1H), 4.12 (t, *J* = 9.6 Hz, 1H), 3.98 (dd, *J* = 11.1, 4.3 Hz, 1H), 3.91 (dd, *J* = 9.8, 3.1 Hz, 2H), 3.88 – 3.81 (m, 2H), 3.69 – 3.63 (m, 1H), 3.58 (dd, *J* = 10.7, 6.9 Hz, 1H), 1.42 (s, 3H), 1.30 (s, 3H), 1.06 (s, 9H). ^{13}C NMR (101 MHz, Chloroform-*d*) δ 138.6, 138.6, 136.0, 135.7, 134.0, 133.5, 129.6, 129.6, 128.4, 128.4, 128.4, 128.1, 127.8, 127.7, 127.7, 127.6, 127.6, 113.4, 98.8, 98.6, 89.8, 88.2, 81.6, 80.0, 75.3, 75.3, 74.7, 73.6, 72.9, 72.3, 67.5, 63.1, 26.9, 26.7, 25.5, 19.4.

HRMS (ESI) m/z: [M + Na]$^+$ Calcd for $C_{51}H_{59}ClO_9SiNa$ 901.3509; Found 901.3549.

((2R,3R,4S,5S,6R)-3,4,5-tris(benzyloxy)-6-chlorotetrahydro-2H-pyran-2-yl)methanol[9].

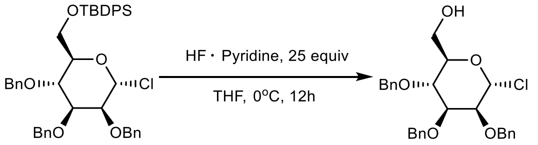

To a 50 mL round bottom flask was added compound **2h** [6] HRMS (ESI) m/z: [M + Na]$^+$ Calcd for $C_{43}H_{47}ClO_5SiNa$ 729.2773; Found 729.2750. (706 mg, 1.0 mmol, 1.0 equiv), anhydrous tetrahydrofuran (4.8 mL, 0.21 M), and HF-Py (0.66 mL, 70% HF in pyridine). The reaction mixture was stirred under nitrogen at 0°C for 12 h. The solvent and excess HF-Py was removed by sparging N_2 at 0°C and the residue was purified by silica gel column chromatography, eluting with Hexanes: EtOAc（7:1 to 3:1 (v/v)) to afford the title compound (234 mg, 0.5 mmol, 50%) as a white foam. ^{1}H NMR (600 MHz, Chloroform-d) δ 7.36 – 7.31 (m, 15H), 6.02 (s, 1H), 4.95 (d, J = 10.8 Hz, 1H), 4.74 (d, J = 12.2 Hz, 1H), 4.69 – 4.66 (m, 3H), 4.65 (s, 1H), 4.61 (d, J = 11.7 Hz, 1H), 4.19 (dd, J = 9.6, 3.1 Hz, 1H), 4.06 (t, J = 9.7 Hz, 1H), 3.90 (dt, J = 9.8, 3.2 Hz, 1H), 3.87 (dd, J = 2.9, 1.9 Hz, 1H), 3.84 (dd, J = 12.2, 2.6 Hz, 1H), 3.80 (dd, J = 12.2, 4.1 Hz, 1H). ^{13}C NMR (151 MHz, Chloroform-d) δ 138.1, 138.0, 137.5, 128.5, 128.4, 128.1, 128.0, 127.9, 127.8, 127.8, 91.4, 78.3, 77.9, 75.3, 75.1, 73.8, 73.2, 72.6, 61.6.

HRMS (ESI) m/z: [M + Na]+ Calcd for $C_{27}H_{29}ClO_5Na$ 491.1596; Found 491.1577

((2R,3R,4S,5S,6R)-3,4,5-tris(benzyloxy)-6-chlorotetrahydro-2H-pyran-2-yl)methyl (S)-2-(4-isobutylphenyl)propanoate (2m)[10-11]

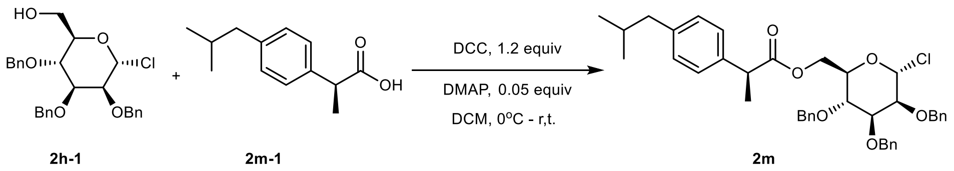

A suspension of Ibuprofen **2m-1** (115 mg, 0.60 mmol, 1.50 equiv) and DMAP (3.0 mg, 0.025 mmol, 5.00 mol%) in DCM (3.0 mL) was added a solution of DCC (124 mg, 0.6 mmol, 1.2 equiv) in DCM (1.00 mL) at 0°C. After stirring for 10 min at 0°C, **2h-1** (234 mg, 0.5 mmol, 1.0 equiv) was added. The reaction mixture was stirred at room temperature for 12 h, quenched with saturated $NaHCO_3$ solution (6.00 mL), and extracted with DCM (2×30 mL). The organic layer was collected, washed with brine, dried with anhydrous Na_2SO_4 and filtered. The filtrate was concentrated in vacuo. The residue was purified by flash column chromatography on silica gel, eluting with Hexanes: EtOAc to afford **2m** (420 mg, 0.32 mmol, 64%) as a white solid. ^1H NMR (600 MHz, Chloroform-d) δ 7.40 (d, J = 7.2 Hz, 2H), 7.38 – 7.28 (m, 8H), 7.26 (d, J = 8.0 Hz, 3H), 7.20 (d, J = 8.0 Hz, 2H), 7.14 – 7.05 (m, 2H), 6.99 (d, J = 7.9 Hz, 2H), 6.07 (s, 1H), 4.72 (q, J = 12.0 Hz, 2H), 4.64 (d, J = 11.7 Hz, 1H), 4.59 (d, J = 11.7 Hz, 1H), 4.55 (d, J = 10.5 Hz, 1H), 4.33 (d, J = 2.5 Hz, 2H), 4.13 (dd, J = 9.6, 2.9 Hz, 1H), 4.02 – 3.96 (m, 1H), 3.94 (d, J = 10.5 Hz, 1H), 3.91 – 3.84 (m, 2H), 3.76 (q, J = 7.1 Hz, 1H), 2.34 (dd, J = 7.1, 2.4 Hz, 2H), 1.77 – 1.67 (m, 1H), 1.49 (d, J = 7.1 Hz, 3H), 0.81 (dd, J = 6.6, 1.9 Hz, 6H). ^{13}C NMR (151 MHz, Chloroform-d) δ 174.3, 140.6, 138.0, 137.9, 137.7, 137.5, 129.4, 128.5, 128.5, 128.4, 128.0, 127.9, 127.8, 127.8, 127.3, 91.3, 78.3, 78.0, 75.2, 73.6, 73.1, 72.9, 72.5, 62.5, 45.0, 44.9, 30.1, 22.4, 22.4, 17.9. HRMS (ESI) m/z: [M + Na]+ Calcd for $C_{40}H_{45}ClO_6Na$ 679.2797; Found 679.2782

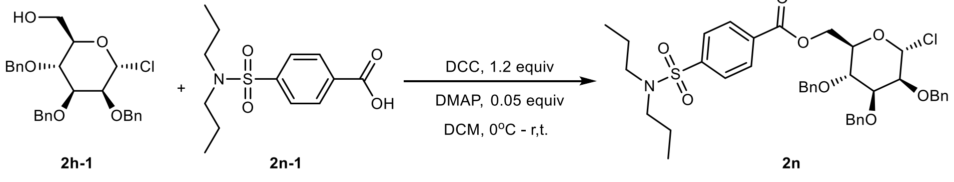

The reaction was performed according to the same procedure as synthesizing **2m**. Probenecid **2n-1** (171 mg, 0.6 mmol, 1.5 equiv) was used as the coupling partner. After work up, the reaction mixture was purified by flash column chromatography on silica gel, eluting with Hexanes: EtOAc to afford **2n** (264.6 mg, 0.36 mmol, 60%) as a white solid. ^{1}H NMR (600 MHz, Chloroform-d) δ 8.03 (d, J = 7.9 Hz, 2H), 7.72 (d, J = 8.2 Hz, 2H), 7.40 – 7.27 (m, 12H), 7.25 – 7.21 (m, 2H), 7.18 – 7.13 (m, 1H), 6.14 – 6.08 (m, 1H), 4.95 (d, J = 10.9 Hz, 1H), 4.75 – 4.67 (m, 3H), 4.65 (d, J = 11.8 Hz, 2H), 4.58 – 4.50 (m, 2H), 4.32 – 4.27 (m, 1H), 4.18 – 4.12 (m, 2H), 3.95 – 3.92 (m, 1H), 3.09 – 3.04 (m, 4H), 1.58 – 1.50 (m, 4H), 0.89 – 0.84 (m, 6H). ^{13}C NMR (151 MHz, Chloroform-d) δ 164.8, 144.3, 137.9, 137.8, 137.7, 133.2, 130.4, 128.6, 128.6, 128.5, 128.4, 128.3, 128.1, 128.1, 128.0, 128.0, 127.8, 127.0, 91.1, 78.5, 78.0, 77.5, 77.5, 77.3, 77.3, 77.1, 75.3, 73.2, 73.0, 73.0, 72.7, 72.5, 63.3, 50.1, 22.1, 11.3.

HRMS (ESI) m/z: [M + Na]$^+$ Calcd for $C_{40}H_{45}ClO_6Na$ 758.2525; Found. 758.2517.

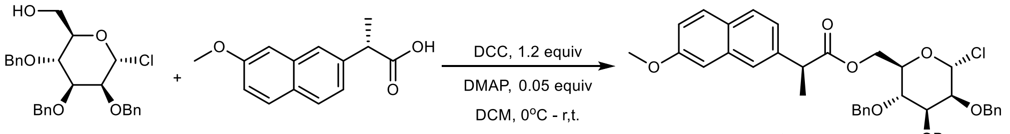

2h-1 **2o-1** **2o**

The reaction was performed according to the same procedure as synthesizing **2m**. Naproxen **2o-1** (138 mg, 0.60 mmol, 1.50 equiv) was used as the coupling partner. After work up, the reaction mixture was purified by flash column chromatography on silica gel, eluting with Hexanes: EtOAc to afford **2o** (265 mg, 0.39 mmol, 65%) as a white solid. ^{1}H NMR (600 MHz, Chloroform-d) δ 7.66 (s, 1H), 7.59 (dd, J = 8.6, 5.8 Hz, 2H), 7.41 – 7.28 (m, 10H), 7.25 (d, J = 5.3 Hz, 1H), 7.20 – 7.15 (m, 3H), 7.06 (dd, J = 8.9, 2.5 Hz, 1H), 7.01 (d, J = 2.3 Hz, 1H), 6.91 (dd, J = 7.1, 2.0 Hz, 2H), 6.07 – 6.04 (m, 1H), 4.74 – 4.67 (m, 2H), 4.60 – 4.52 (m, 2H), 4.39 – 4.34 (m, 2H), 4.28 (dd, J = 12.3, 3.6 Hz, 1H), 4.10 (dd, J = 9.5, 3.0 Hz, 1H), 4.01 – 3.96 (m, 1H), 3.92 (q, J = 7.1 Hz, 1H), 3.89 – 3.82 (m, 6H), 1.57 (d, J = 7.1 Hz, 3H). ^{13}C NMR (151 MHz, Chloroform-d) δ 174.3, 157.6, 138.0, 137.8, 137.7, 135.4, 133.8, 129.3, 129.0, 128.5, 128.4, 128.2, 128.0, 127.9, 127.8, 127.7, 127.6, 127.3, 126.4, 126.1, 119.0, 105.5, 91.3, 78.2, 77.9, 74.9, 73.6, 73.1, 72.8, 72.4, 62.6, 55.3, 45.3, 18.0.

HRMS (ESI) m/z: [M + Na]$^+$ Calcd for $C_{41}H_{41}ClO_7Na$ 703.2433; Found 703.2409.

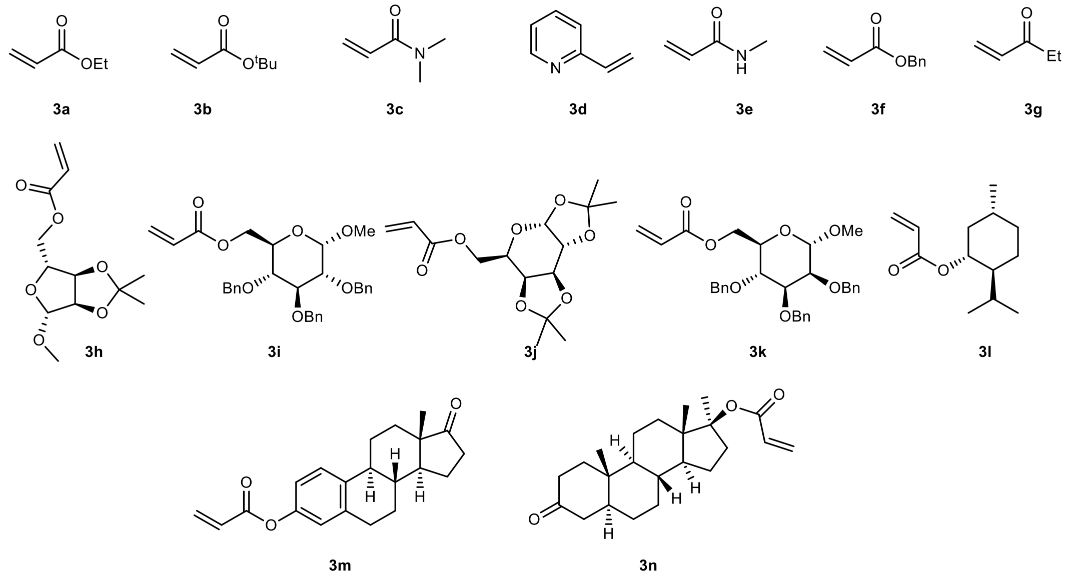

Supplementary Figure 3. Scope of alkene substrates

3a-**3g** are commercially available. **3h**-**3n** was synthesized as follows:

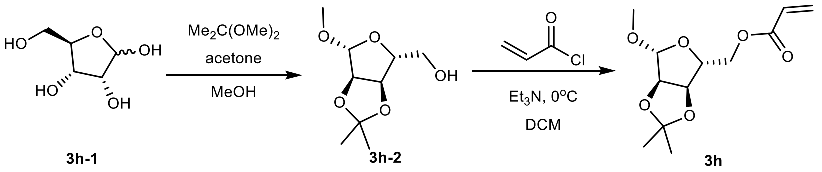

Methyl 2,3-O-isopropylidene-5-deoxy-b-d-ribofuranoside (**3h-2**):

Step 1: 2,2-dimethoxy-propane (30 mL) was added to a suspension of d-ribose (**3h-1**, 12.3 g, 81.7 mmol) in acetone and methanol (120 mL, 2:1, anhydrous). After cooling to 0°C, HCl (4 M in dioxane, 6 mL) was added dropwise. The mixture was allowed to warm to room temperature under nitrogen overnight. After cooling to 0°C, the mixture was neutralized with pyridine (3.9 mL, 48.4 mmol), stirred for 30 min, and then concentrated in vacuo. The residue was dissolved in di-chloromethane (200 mL), and then washed successively with aqueous 1M HCl solution (2*50 mL), brine (50 mL), saturated $NaHCO_3$ aqueous solution (50 mL), brine (50 mL) and then dried over $MgSO_4$.Filtration and then evaporation in vacuo gave the **3h-2** as a yellow oil (33 mmol, 12.0 g), which was used in the next step without further purification.

Step 2: **((3aR,4R,6R,6aR)-6-methoxy-2,2-dimethyltetrahydrofuro[3,4-d] [1,3] dioxol-4-yl) methyl acrylate (3h).**

The acryloyl chloride (1.5 mmol, 0.32 mL) in CH_2Cl_2 was added dropwise to a solution of **3h-2** (1.0 mmol), and triethylamine (Et_3N) (2.0 mmol, 0.72 mL) in CH_2Cl_2 at 0°C and then stirred at

room temperature for 16 h. Upon completion, the reaction mixture was washed by brine (3×20 mL), dried over anhydrous Na_2SO_4, and concentrated under vacuum. The residue was purified with column chromatography on silica gel (petroleum ether: ethyl acetate= 8:1) to yield compound **3h** as colorless liquid.

^{1}H NMR (400 MHz, Chloroform-d) δ 6.46 (d, J = 17.3 Hz, 1H), 6.15 (dd, J = 17.3, 10.4 Hz, 1H), 5.87 (d, J = 10.4 Hz, 1H), 4.99 (s, 1H), 4.70 (d, J = 5.8 Hz, 1H), 4.62 (d, J = 5.9 Hz, 1H), 4.42 (t, J = 6.9 Hz, 1H), 4.25 – 4.14 (m, 2H), 3.32 (s, 3H), 1.49 (s, 3H), 1.33 (s, 3H). ^{13}C NMR (101 MHz, Chloroform-d) δ 165.7, 131.4, 128.0, 112.6, 109.4, 85.2, 84.2, 81.9, 64.7, 54.9, 26.4, 25.0. HRMS (ESI) m/z: [M + Na]$^+$ Calcd for $C_{12}H_{18}O_6Na$ 281.0996 Found 281.0995.

((2R,3R,4S,5R,6S)-3,4,5-tris(benzyloxy)-6-methoxytetrahydro-2H-pyran-2-yl) methyl acrylate (3i).

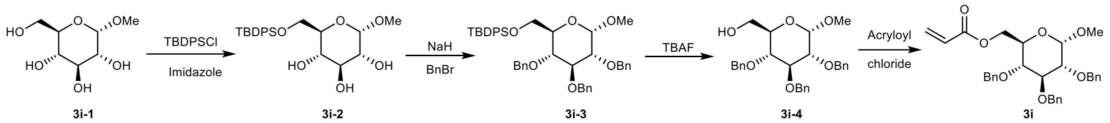

Compound **3i-1** (25 mmol) and Imidazole (30 mmol) were dissolved in anhydrous DMF (50 mL) and the reaction mixture was cooled to 0°C. To this solution was added TBDPSCl (35 mmol) and the reaction mixture was brought to room temperature slowly with stirring. After 16h, the reaction mixture was quenched by adding water. The resulting residue was dissolved in ethyl acetate. The organic solution was washed with water and brine, and then dried over anhydrous Na_2SO_4. After the volatiles were removed by rotary evaporation under reduced pressure, the crude product was purified by flash chromatography to yield **3i-2** as white solid. Compound **3i-2** (20 mmol) was dissolved in anhydrous DMF (80 mL) and cooled to 0°C. The solution was then carefully treated with sodium hydride (60%, 80 mmol) and stirred at 0°C for 30 min. Benzyl bromide (80 mmol) was added slowly and the reaction mixture was brought to room temperature. After overnight, the reaction mixture was quenched by adding water. The resulting residue was mixed with water and extracted with ethyl ether. The combined organic layers were dried over anhydrous Na_2SO_4. After the volatiles were removed by rotary evaporation under reduced pressure, the crude product was purified using flash chromatography to yield **3i-3**. Compound **3i-3** (6 mmol) was dissolved in anhydrous THF (20 mL) and added tetrabutylammonium fluoride solution (1.0 M in THF, 12 mL). The resulting mixture was stirred at room temperature for over-

night. The reaction was quenched by adding saturated ammonium chloride solution and extracted with ethyl acetate. The combined organic layers were dried over anhydrous Na_2SO_4. After the volatiles were removed by rotary evaporation, the crude product was purified using flash chromatography to yield **3i-4** as colorless solid. According to the general procedure (**3h**, Step 2), the crude residue was purified by flash column chromatography on silica gel to yield compound **3i.** ^{1}H NMR (400 MHz, Chloroform-d) δ 7.50 – 7.05 (m, 15H), 6.52 – 6.28 (m, 1H), 6.10 (dd, J = 17.3, 10.4 Hz, 1H), 5.95 – 5.72 (m, 1H), 5.00 (d, J = 10.8 Hz, 1H), 4.94 – 4.73 (m, 3H), 4.71 – 4.49 (m, 3H), 4.35 (d, J = 3.3 Hz, 2H), 4.02 (t, J = 9.2 Hz, 1H), 3.95 – 3.77 (m, 1H), 3.61 – 3.44 (m, 2H), 3.37 (s, 3H). ^{13}C NMR (101 MHz, Chloroform-*d*) δ 165.8, 138.6, 138.1, 137.8, 131.2, 128.5, 128.5, 128.1, 128.1, 128.0, 128.0, 127.9, 127.7, 98.0, 82.1, 79.9, 77.5, 75.9, 75.1, 73.4, 68.7, 63.1, 55.2.

HRMS (ESI) m/z: [M + Na]$^+$ Calcd for $C_{31}H_{34}O_7Na$ 541.2197; Found 541.2169.

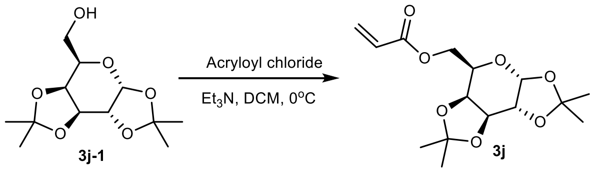

According to the general procedure (**3h**, Step 2)[12]. ^{1}H NMR (400 MHz, Chloroform-*d*) δ 6.44 (d, *J* = 17.3 Hz, 1H), 6.17 (dd, *J* = 17.5, 10.3 Hz, 1H), 5.84 (d, *J* = 10.4 Hz, 1H), 5.55 (d, *J* = 4.9 Hz, 1H), 4.64 (d, *J* = 7.8 Hz, 1H), 4.42 – 4.36 (m, 1H), 4.34 (dd, *J* = 4.8, 2.4 Hz, 1H), 4.31 – 4.18 (m, 2H), 4.11 – 4.01 (m, 1H), 1.49 (d, *J* = 20.7 Hz, 6H), 1.34 (d, *J* = 5.3 Hz, 6H). ^{13}C NMR (101 MHz, Chloroform-*d*) δ 166.1, 131.1, 128.2, 109.7, 108.8, 96.3, 71.1, 70.7, 70.5, 66.0, 63.5, 26.0, 26.0, 25.0, 24.5.

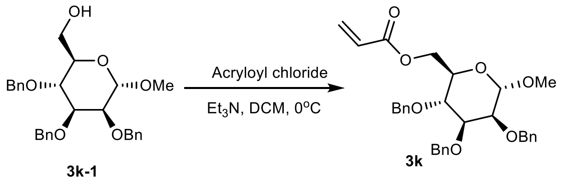

According to the general procedure (**3i**). ^{1}H NMR (400 MHz, Chloroform-*d*) δ 7.45 – 7.15 (m, 15H), 6.41 (dd, *J* = 17.3, 1.4 Hz, 1H), 6.14 (dd, *J* = 17.3, 10.4 Hz, 1H), 5.81 (dd, *J* = 10.4, 1.4 Hz, 1H), 4.93 (d, *J* = 10.7 Hz, 1H), 4.79 – 4.66 (m, 3H), 4.62 (s, 2H), 4.58 (d, *J* = 10.8 Hz, 1H), 4.47 – 4.37 (m, 2H), 4.03 – 3.86 (m, 2H), 3.85 – 3.74 (m, 2H), 3.31 (s, 3H). ^{13}C NMR (101 MHz,

Chloroform-*d*) δ 166.0, 138.3, 138.3, 138.2, 131.0, 128.4, 128.4, 128.4, 128.3, 128.1, 127.8, 127.8, 127.7, 127.7, 98.9, 80.2, 75.2, 74.5, 74.5, 72.6, 72.1, 70.0, 63.6, 54.8.

HRMS (ESI) m/z: $[M+Na]^+$ Calcd for $C_{31}H_{34}O_7Na$ 541.2197; Found 541.2173.

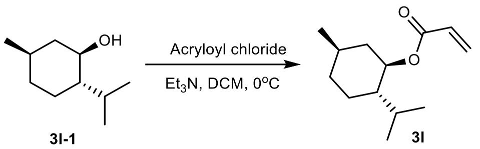

3l-1 **3l**

According to the general procedure (**3h**, Step 2). ^{1}H NMR (400 MHz, Chloroform-*d*) δ 6.39 (d, *J* = 17.3 Hz, 1H), 6.11 (dd, *J* = 17.3, 10.4 Hz, 1H), 5.80 (d, *J* = 10.4 Hz, 1H), 4.76 (td, *J* = 10.9, 4.3 Hz, 1H), 2.09 – 1.99 (m, 1H), 1.93 – 1.82 (m, 1H), 1.76 – 1.64 (m, 2H), 1.58 – 1.47 (m, 1H), 1.47 – 1.38 (m, 1H), 1.14 – 0.96 (m, 2H), 0.93 – 0.83 (m, 7H), 0.77 (d, *J* = 6.9 Hz, 3H). ^{13}C NMR (101 MHz, Chloroform-*d*) δ 165.8, 130.2, 129.0, 74.3, 47.1, 40.8, 34.2, 31.4, 26.3, 23.5, 22.0, 20.7, 16.4.

HRMS (ESI) m/z: $[M + Na]^+$ Calcd for $C_{13}H_{22}O_2Na$ 233.1512; Found 233.1505.

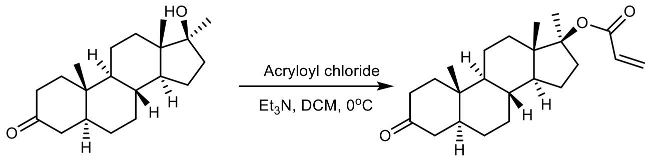

3n-1 **3n**

According to the general procedure (**3h**, Step 2)[13]. ^{1}H NMR (400 MHz, Chloroform-*d*) δ 6.38 (d, *J* = 17.3 Hz, 1H), 6.26 – 5.98 (m, 1H), 5.81 (d, *J* = 10.3 Hz, 1H), 4.90 – 4.41 (m, 1H), 2.71 (dd, *J* = 14.5, 5.7 Hz, 1H), 2.31 – 2.16 (m, 2H), 2.15 – 1.97 (m, 3H), 1.87 – 1.74 (m, 2H), 1.74 – 1.61 (m, 2H), 1.60 – 1.44 (m, 3H), 1.44 – 1.26 (m, 4H), 1.24 – 1.18 (m, 1H), 1.16 – 1.10 (m, 3H), 1.06 – 0.95 (m, 2H), 0.87 (d, *J* = 7.2 Hz, 3H), 0.86 – 0.77 (m, 3H). ^{13}C NMR (101 MHz, Chloroform-*d*) δ 212.0, 166.2, 130.2, 128.9, 82.8, 50.7, 48.5, 45.9, 44.9, 43.0, 39.8, 39.5, 37.8, 36.8, 35.3, 31.1, 28.7, 27.5, 23.6, 20.0, 14.7, 14.4, 12.2.

3. General Procedure.

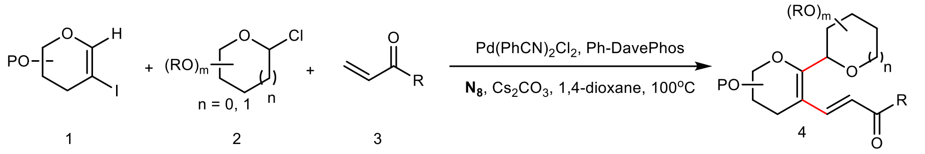

In a dried 10 ml tube equipped with a stirring bar, **1** (0.15 mmol, 1.5 equiv), **2** (0.2 mmol, 2.0 equiv), $Pd(PhCN)_2Cl_2$ (0.01 mmol, 0.1 equiv), Ph-Davephos (0.02 mmol, 0.2 equiv) and Cs_2CO_3 (0.2 mmol, 2.0 equiv) were added. The tube charged with argon more than three times. **3** (0.1 mmol, 1.0 equiv), $\mathbf{N_8}$ (0.2 mmol, 2.0 equiv) and 1,4-dioxane (1.0 mL) were injected into the tube via microsyringe and plastic syringe, respectively. The resulting suspension was placed in an oil bath that had been preheated to 100°C for 16 h, and then the mixture was cooled to r.t. The reaction mixture filtered and concentrated in vacuo. The residue was purified with chromatography column on silica gel to give 4. (Petroleum ether/EtOAc).

General Procedure for gram experiment：

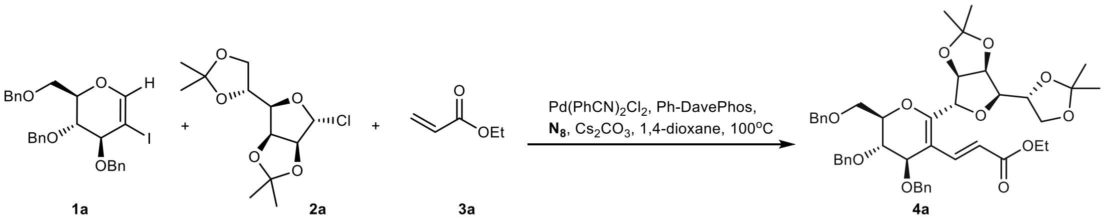

In a dried 50 ml tube equipped with a stirring bar, **1a** (3.6 mmol, 1.5 equiv, 1.95 g), **2a** (4.8 mmol, 2.0 equiv, 1.33 g), $Pd(PhCN)_2Cl_2$ (0.24 mmol, 0.1 equiv), Ph-Davephos (0.48 mmol, 0.2 equiv), and Cs_2CO_3 (4.8 mmol, 2.0 equiv) were added. The tube charged with argon more than three times. **3a** (2.4 mmol, 1 equiv), $\mathbf{N_8}$ (4.8 mmol, 2.0 equiv) and 1,4-dioxane (24.0 mL) were injected into the tube via microsyringe or plastic syringe. The resulting suspension was placed in an oil bath that had been preheated to 100°C for 16 h, and then the mixture was cooled to r.t. The reaction mixture filtered and concentrated in vacuo. The residue was purified with chromatography column on silica gel to give **4a** in 46% yield (837.6 mg). (Petroleum ether/EtOAc= 8:1-4:1).

Procedure for Synthesis of 5a：

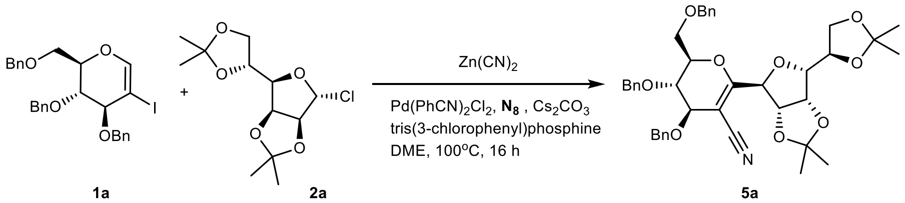

In a dried 10 ml tube equipped with a stirring bar, **1a** (0.1 mmol, 1.0 equiv), **2a** (0.2 mmol, 2 equiv), $Zn(CN)_2$ (0.15 mmol, 1.5 equiv), $Pd(PhCN)_2Cl_2$ (0.01 mmol, 0.1 equiv), P(*p*-$ClC_6H_4)_3$ (0.02 mmol, 0.2 equiv) and Cs_2CO_3 (0.2 mmol, 2.0 equiv) were added. The tube charged with argon more than three times. $\mathbf{N_8}$ (0.2 mmol, 2.0 equiv) and DME (1.0 mL) was injected into the tube via microsyringe and plastic syringe, respectively. The resulting suspension was placed in an oil bath that had been preheated to 100°C for 16 h, and then the mixture was cooled to r.t. The reaction mixture filtered, and concentrated in vacuo. The residue was purified with chromatography column on silica gel to give **5a** in 30% yield. (Petroleum ether/EtOAc= 8:1-4:1).

Procedure for Synthesis of 7a：

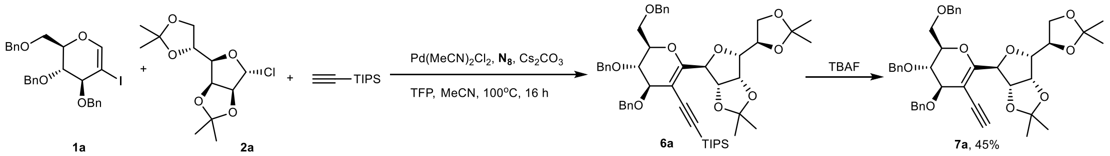

In a dried 10 ml tube equipped with a stirring bar, **1a** (0.15 mmol, 1.0 equiv), **2a** (0.2 mmol, 2.0 equiv), $Pd(PhCN)_2Cl_2$ (0.01 mmol, 0.1 equiv), TFP (0.02 mmol, 0.2 equiv) and Cs_2CO_3 (0.2 mmol, 2.0 equiv) were added. The tube charged with argon more than three times. $\mathbf{N_8}$ (0.2 mmol, 2.0 equiv), (triisopropylsilyl)acetylene (0.1 mmol, 1.0 equiv) and DME (1.0 mL) were injected into the tube via microsyringe and plastic syringe, respectively. The resulting suspension was placed in an oil bath that had been preheated to 100°C for 16 h, and then the mixture was cooled to r.t. The reaction mixture filtered, and concentrated in vacuo. The residue was purified with chromatography column on silica gel to give **6a** in 46% yield. (Petroleum ether/EtOAc= 8:1-5:1).

(((2R,3S)-3,4-bis(benzyloxy)-2-((benzyloxy)methyl)-6-((3aS,4S,6R,6aS)-6-((R)-2,2-dimethyl-1,3-dioxolan-4-yl)-2,2-dimethyltetrahydrofuro[3,4-d][1,3]dioxol-4-yl)-3,4-dihydro-2H-pyran-5-yl)ethynyl)triisopropylsilane ^1H NMR (600 MHz, Chloroform-*d*) δ 7.30 (dd, *J* = 14.0, 6.9 Hz, 13H), 7.22 (d, *J* = 7.8 Hz, 2H), 5.22 (s, 1H), 4.97 (d, *J* = 11.3 Hz, 1H), 4.86 (d, *J* = 6.1 Hz, 1H), 4.76 – 4.74 (m, 1H), 4.70 (dd, *J* = 11.3, 9.1 Hz, 2H), 4.59 (d, *J* = 11.5 Hz, 1H), 4.50 – 4.49 (m, 2H), 4.33 (d, *J* = 7.6 Hz, 1H), 4.20 – 4.18 (m, 2H), 4.13 (dd, *J* = 7.8, 3.8 Hz, 1H), 4.09 – 4.07 (m, 1H), 4.05 – 4.03 (m, 1H), 3.82 – 3.80 (m, 1H), 3.78 – 3.76 (m, 1H), 3.63 (dd, *J* = 10.9, 2.9 Hz, 1H), 1.47 (s, 3H), 1.39 (s, 3H), 1.37 (s, 3H), 1.32 (s, 3H), 1.08 (s, 18H). ^{13}C NMR (151 MHz, Chloroform-*d*) δ 160.7, 138.2, 137.9, 137.9, 128.4, 128.3, 127.8, 127.8, 127.8, 127.7,

127.6, 127.4, 112.7, 109.0, 97.2, 95.2, 84.8, 83.4, 82.4, 81.6, 73.7, 73.3, 73.0, 68.2, 67.1, 26.9, 26.3, 25.3, 25.1, 18.7, 11.4. HRMS (ESI) *m/z*: $[M + Na]^+$ Calcd for $C_{50}H_{66}NaO_9Si$ 861.4368 found 861.4355. $[\alpha]_D^{25}$=47.0 (c=5 mg/ml, CH_2Cl_2).

In a dry 50 mL round-bottom flask equipped with a stirring bar, **5a** (1 mmol) was dissolved in dry Et_2O (15 mL) with stirring. After 1 M solution of $^nBu_4N^+F^-$ in THF (1.5 mL) was added, the mixture was stirred for 20 minutes at r.t. The mixture was concentrated under vacuum, and the resulting residue was purified by column chromatography (Petroleum ether/EtOAc= 8:1 - 5:1). to give **7a** in 98% yield.

Procedure for Synthesis of 8a：

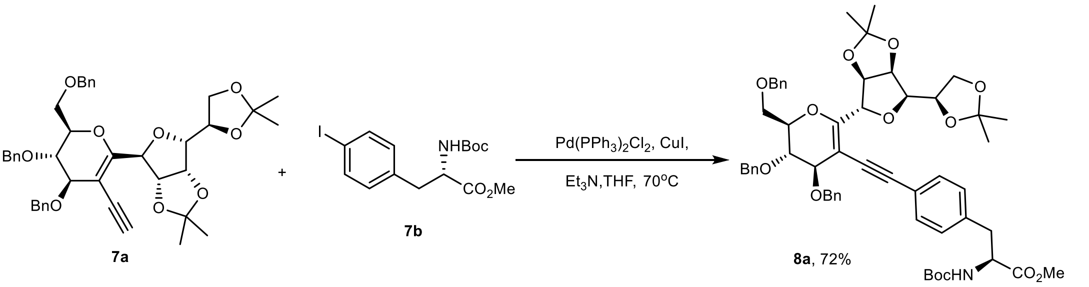

To a dried 10 mL Schlenk tube were added **7a** (68.2 mg, 0.1 mmol), methyl (S)-2-((tert-butoxycarbonyl)amino)-3-(4-iodophenyl)propanoate **7b** (44.6 mg, 0.11 mmol, 1.1 equiv), $Pd(PPh_3)_2Cl_2$ (3.5 mg, 5 mol%) and CuI (3.8 mg, 20 mol%). The tube charged with argon more than three times. Then Et_3N (15.2 mg, 0.15 mmol, 1.5 equiv) and THF (1 ml) were injected into the tube via microsyringe and plastic syringe, respectively. The reaction mixture was heated to 70°C for 12 h under vigorous stirring. Upon completion, the reaction mixture was cooled to room temperature. The reaction mixture filtered, and concentrated in vacuo. The residue was purified with chromatography column on silica gel to give **8a** in 72% yield. (Petroleum ether/EtOAc= 5:1 - 3:1).

Procedure for Synthesis of 9a：

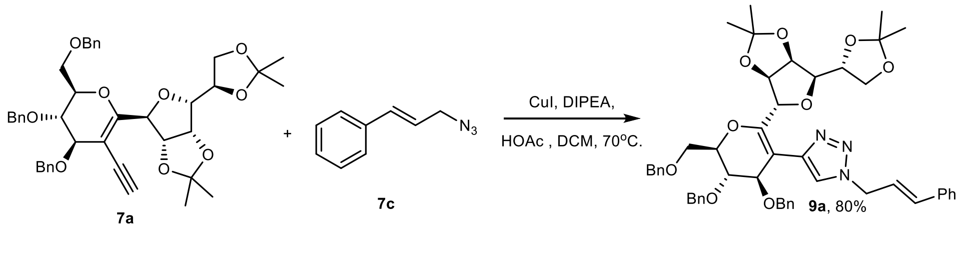

A dried 10 mL Schlenk tube was charged with **7a** (68.2 mg, 0.1 mmol), (E)-(3-azidoprop-1-en-1-yl)benzene **7c** (31.8 mg, 0.15 mmol, 1.5 equiv), N-ethyl-N-isopropylpropan-2-amine (DIEPA, 1.3mg, 10 mol%), AcOH (0.6 mg, 10 mol%) and CuI (1.9 mg, 10 mol%). The tube charged with

argon more than three times. DME (1.0 mL) was injected into the tube via plastic syringe. The reaction mixture was heated to 70°C for 25 h under vigorous stirring. Upon completion, the reaction mixture was cooled to room temperature. The reaction mixture filtered, and concentrated in vacuo. The residue was purified with chromatography column on silica gel to give **9a** in 80% yield. (Petroleum ether/EtOAc= 4:1 - 2:1).

Procedure for Synthesis of 10a:

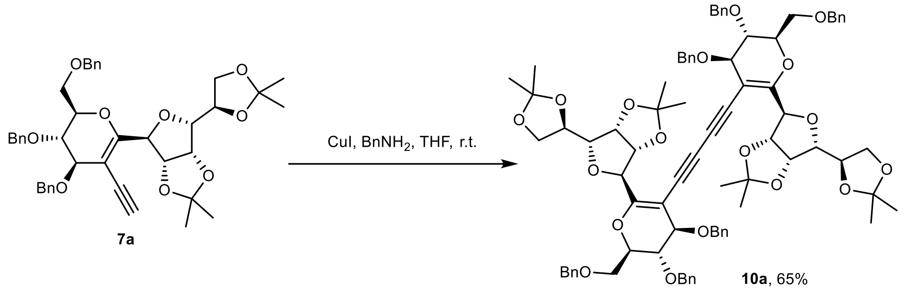

To a dried 10 mL Schlenk tube were added **7a** (68.2 mg, 0.1 mmol), CuI (1.9 mg, 10 mol%), $BnNH_2$ (2.2 mg, 20 mol%). The tube was evacuated and back filled with oxygen balloon. Then THF (1 ml) was injected into the tube via plastic syringe, the mixture was stirred for 16 h in r.t. The reaction mixture filtered, and concentrated in vacuo. The residue was purified with chromatography column on silica gel to give **10a** in 65% yield. (Petroleum ether/EtOAc= 5:1 - 3:1).

Procedure for Synthesis of 11a:

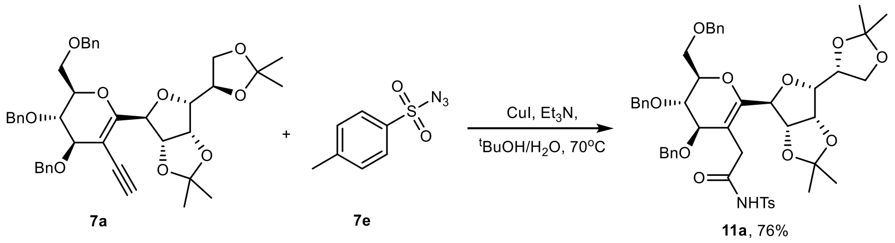

A dried 10 mL Schlenk tube was charged with *p*-toluenesulfonyl azide **7e** (0.12 mmol), **7a** (68.2 mg, 0.1 mmol) and CuI (3.8 mg, 20 mol%), and then the tube was evacuated and back filled with nitrogen (5 times). Et_3N (15.2 mg, 0.15 mmol, 1.5 equiv) and $^tBuOH/H_2O$ (2:1, 1 mL) were injected into the tube via microsyringe and plastic syringe, respectively. The reaction mixture was heated to 70°C for 12 h under vigorous stirring. Upon completion, the reaction mixture was cooled to room temperature. The mixture was concentrated under vacuum, and the resulting residue was purified with chromatography column on silica gel ethyl to give **11a** in 76% yield. (Petroleum ether/EtOAc= 5:1 - 2:1).

4. Characterization Data

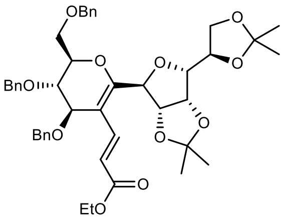

4a

ethyl (E)-3-((2R,3S,4R)-3,4-bis(benzyloxy)-2-((benzyloxy)methyl)-6-((3aS,4S,6R,6aS)-6-((R)-2,2-dimethyl-1,3-dioxolan-4-yl)-2,2-dimethyltetrahydrofuro[3,4-d][1,3]dioxol-4-yl)-3,4-dihydro-2H-pyran-5-yl)acrylate

Colorless liquid, 49.1 mg, 64%. Purified by chromatography on silicagel, eluting with petroleum ether/ethyl acetate 8:1-4:1 (v/v). $[\alpha]_D^{24}$=60.0 (c=5 mg/ml, CH_2Cl_2).

^{1}H NMR (600 MHz, Chloroform-*d*) δ 7.70 (d, *J* = 15.6 Hz, 1H), 7.37 – 7.29 (m, 11H), 7.27 – 7.26 (m, 1H), 7.26 – 7.24 (m, 1H), 7.20 (dd, *J* = 7.4, 2.1 Hz, 2H), 5.78 (d, *J* = 15.6 Hz, 1H), **5.14 (s, 1H) (anomeric H),** 4.92 (d, *J* = 6.1 Hz, 1H), 4.82 (dd, *J* = 6.0, 4.0 Hz, 1H), 4.66 (d, *J* = 2.3 Hz, 2H), 4.46 (d, *J* = 4.7 Hz, 2H), 4.44 – 4.40 (m, 3H), 4.35 – 4.31 (m, 1H), 4.26 (dd, *J* = 3.5, 1.4 Hz, 1H), 4.19 (q, *J* = 7.1 Hz, 2H), 4.09 – 4.06 (m, 2H), 4.01 (dd, *J* = 8.7, 4.8 Hz, 1H), 3.95 (t, *J* = 3.9 Hz, 1H), 3.76 (dd, *J* = 10.6, 7.1 Hz, 1H), 3.57 (dd, *J* = 10.6, 4.1 Hz, 1H), 1.53 (s, 3H), 1.40 (s, 3H), 1.37 (s, 3H), 1.32 (s, 3H), 1.28 (t, *J* = 7.1 Hz, 3H).

^{13}C NMR (151 MHz, Chloroform-*d*) δ 167.2, 157.9, 139.1, 137.6, 137.6, 137.2, 128.6, 128.5, 128.0, 128.0, 128.0, 127.9, 127.7, 127.6, 115.8, 112.7, 109.1, 108.7, 84.0, 83.4, 81.6, 80.1, 75.8, 73.5, 73.3, 71.8, 71.8, 70.3, 70.2, 68.0, 67.1, 60.1, 26.9, 26.1, 25.2, 24.5, 14.4.

HRMS (ESI) *m/z*: $[M + Na]^+$ Calcd for $C_{44}H_{52}NaO_{11}$ 779.3402 found 779.3373.

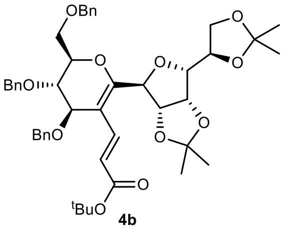

4b

tert-butyl (E)-3-((2R,3S,4R)-3,4-bis(benzyloxy)-2-((benzyloxy)methyl)-6-((3aS,4S,6R,6aS)-6-((R)-2,2-dimethyl-1,3-dioxolan-4-yl)-2,2-dimethyltetrahydrofuro[3,4-d][1,3]dioxol-4-yl)-3,4-dihydro-2H-pyran-5-yl)acrylate

Colorless liquid, 54.9mg, 70%. Purified by chromatography on silicagel, eluting with petroleum ether/ethyl acetate 8:1-4:1 (v/v). $[\alpha]_D^{24}$=78.0 (c=5 mg/ml, CH_2Cl_2).

^{1}H NMR (400 MHz, Chloroform-*d*) δ 7.61 (d, *J* = 15.6 Hz, 1H), 7.36 – 7.27 (m, 13H), 7.23 – 7.20 (m, 2H), 5.72 (d, *J* = 15.5 Hz, 1H), 5.14 (s, 1H) **(anomeric H)**, 4.91 (d, *J* = 6.0 Hz, 1H), 4.83 – 4.79 (m, 1H), 4.67 (s, 2H), 4.47 (d, *J* = 2.3 Hz, 2H), 4.44 – 4.40 (m, 3H), 4.35 – 4.30 (m, 1H), 4.28 – 4.24 (m, 1H), 4.10 – 4.06 (m, 2H), 4.01 (dd, *J* = 8.6, 4.8 Hz, 1H), 3.95 (t, *J* = 3.8 Hz, 1H), 3.76 (dd, *J* = 10.4, 7.1 Hz, 1H), 3.57 (dd, *J* = 10.5, 3.8 Hz, 1H), 1.52 (s, 3H), 1.48 (s, 9H), 1.40 (s, 3H), 1.37 (s, 3H), 1.32 (s, 3H).

^{13}C NMR (101 MHz, Chloroform-*d*) δ 166.5, 157.6, 137.9, 137.6, 137.5, 137.2, 128.5, 128.4, 128.1, 128.0, 127.9, 127.8, 127.8, 127.7, 127.6, 117.6, 112.7, 109.0, 108.6, 84.0, 83.4, 81.6, 80.1, 79.8, 77.2, 75.7, 73.5, 73.3, 71.9, 71.7, 70.3, 70.0, 68.0, 67.1, 28.2, 26.8, 26.1, 25.2, 24.6.

HRMS (ESI) *m/z*: $[M + Na]^+$ Calcd for $C_{46}H_{56}NaO_{11}$ 807.3715 found 807.3703.

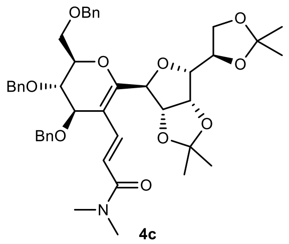

4c

(E)-3-((2R,3S,4R)-3,4-bis(benzyloxy)-2-((benzyloxy)methyl)-6-((3aS,4S,6R,6aS)-6-((R)-2,2-dimethyl-1,3-dioxolan-4-yl)-2,2-dimethyltetrahydrofuro[3,4-d][1,3]dioxol-4-yl)-3,4-dihydro-2H-pyran-5-yl)-N,N-dimethylacrylamide

Colorless liquid, 40 mg, 53%. Purified by chromatography on silicagel, eluting with petroleum ether/ethyl acetate 1:1-1:3 (v/v). $[\alpha]_D^{24}$=104.0 (c=5 mg/ml, CH_2Cl_2).

^{1}H NMR (400 MHz, Chloroform-*d*) δ 7.65 (d, *J* = 15.0 Hz, 1H), 7.35 – 7.26 (m, 13H), 7.25 – 7.21 (m, 2H), 6.36 (d, *J* = 15.0 Hz, 1H), 5.20 (s, 1H) **(anomeric H)**, 4.93 (d, *J* = 6.0 Hz, 1H), 4.84 – 4.79 (m, 1H), 4.72 (q, *J* = 11.7 Hz, 2H), 4.54 – 4.47 (m, 2H), 4.44 – 4.36 (m, 3H), 4.35 – 4.30 (m, 1H), 4.28 – 4.23 (m, 1H), 4.10 – 3.99 (m, 4H), 3.78 (dd, *J* = 10.5, 6.1 Hz, 1H), 3.64 (dd, *J* = 10.6, 3.1 Hz, 1H), 2.97 (s, 3H), 2.85 (s, 3H), 1.52 (s, 3H), 1.39 (s, 3H), 1.37 (s, 3H), 1.33 (s, 3H).

^{13}C NMR (101 MHz, Chloroform-*d*) δ 166.9, 158.0, 137.7, 137.6, 137.6, 136.4, 128.5, 128.4, 128.4, 127.9, 127.8, 127.8, 127.7, 127.7, 127.6, 115.8, 112.7, 109.3, 109.0, 84.1, 83.4, 81.7, 80.1, 76.1, 74.3, 73.5, 73.3, 72.3, 70.9, 68.4, 68.0, 67.0, 37.1, 35.8, 26.9, 26.2, 25.2, 24.7.

HRMS (ESI) *m/z*: $[M + Na]^+$ Calcd for $C_{44}H_{53}NNaO_{10}$ 778.3562 found 778.3580.

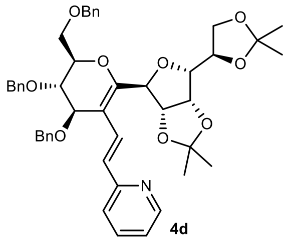

4d

2-((E)-2-((2R,3S,4R)-3,4-bis(benzyloxy)-2-((benzyloxy)methyl)-6-((3aS,4S,6R,6aS)-6-((R)-2,2-dimethyl-1,3-dioxolan-4-yl)-2,2-dimethyltetrahydrofuro[3,4-d][1,3]dioxol-4-yl)-3,4-dihydro-2H-pyran-5-yl)vinyl)pyridine

Yellow liquid, 26.6 mg, 35%. Purified by chromatography on silicagel, eluting with petroleum ether/ethyl acetate 8:1-4:1 (v/v). $[\alpha]_D^{25}$=194.0 (c=5 mg/ml, CH_2Cl_2).

^{1}H NMR (600 MHz, Chloroform-*d*) δ 8.53 (d, *J* = 4.3 Hz, 1H), 7.61 (d, *J* = 15.7 Hz, 1H), 7.56 (td, *J* = 7.7, 1.6 Hz, 1H), 7.37 – 7.24 (m, 15H), 7.15 (d, *J* = 7.9 Hz, 1H), 7.06 (dd, *J* = 7.0, 5.1 Hz, 1H), 6.61 (d, *J* = 15.7 Hz, 1H), 5.27 (s, 1H) **(anomeric H)**, 4.97 (d, *J* = 6.0 Hz, 1H), 4.87 – 4.82 (m, 1H), 4.76 – 4.68 (m, 2H), 4.55 – 4.46 (m, 5H), 4.36 – 4.30 (m, 2H), 4.12 (dd, *J* = 7.9, 4.1 Hz, 1H), 4.07 (dd, *J* = 8.5, 6.4 Hz, 1H), 4.05 – 3.99 (m, 2H), 3.80 (dd, *J* = 10.6, 6.7 Hz, 1H), 3.62 (dd, *J* = 10.6, 3.6 Hz, 1H), 1.54 (s, 3H), 1.39 (s, 3H), 1.37 (s, 3H), 1.34 (s, 3H).

^{13}C NMR (151 MHz, Chloroform-*d*) δ 156.1, 154.8, 149.4, 137.8, 137.8, 137.7, 136.2, 128.5, 128.5, 128.4, 128.1, 127.9, 127.8, 127.8, 127.6, 127.1, 126.5, 121.5, 121.4, 112.4, 110.3, 109.0, 84.0, 83.3, 81.7, 80.0, 75.8, 73.6, 73.3, 71.9, 71.1, 69.3, 68.3, 67.2, 26.9, 26.1, 25.3, 24.4.

HRMS (ESI) *m/z*: $[M + H]^+$ Calcd for $C_{46}H_{52}NO_9$ 762.3637 found 762.3650.

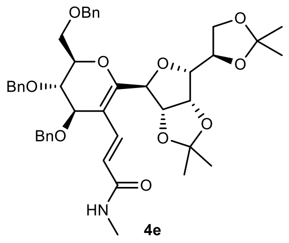

4e

(E)-3-((2R,3S,4R)-3,4-bis(benzyloxy)-2-((benzyloxy)methyl)-6-((3aS,4S,6R,6aS)-6-((R)-2,2-dimethyl-1,3-dioxolan-4-yl)-2,2-dimethyltetrahydrofuro[3,4-d][1,3]dioxol-4-yl)-3,4-dihydro-2H-pyran-5-yl)-N-methylacrylamide

White solid, 38.5 mg, 52%. Purified by chromatography on silicagel, eluting with petroleum ether/ethyl acetate 1:1-1:3 (v/v). $[\alpha]_D^{25}$=124.0 (c=5 mg/ml, CH_2Cl_2). melting point: 73-75°C.

^{1}H NMR (600 MHz, Chloroform-*d*) δ 7.55 (d, *J* = 15.2 Hz, 1H), 7.38 – 7.27 (m, 13H), 7.23 (d, *J* = 6.4 Hz, 2H), 5.52 (d, *J* = 15.2 Hz, 1H), 5.16 (s, 1H) **(anomeric H)**, 5.13 – 5.08 (m, 1H), 4.92

(d, *J* = 6.0 Hz, 1H), 4.83 – 4.78 (m, 1H), 4.70 (s, 2H), 4.54 – 4.47 (m, 2H), 4.41 (d, *J* = 11.4 Hz, 1H), 4.36 – 4.31 (m, 3H), 4.29 (d, *J* = 3.8 Hz, 1H), 4.10 – 4.04 (m, 2H), 4.03 – 3.97 (m, 2H), 3.78 (dd, *J* = 10.5, 6.5 Hz, 1H), 3.63 (dd, *J* = 10.5, 3.8 Hz, 1H), 2.81 (d, *J* = 4.9 Hz, 3H), 1.52 (s, 3H), 1.39 (s, 3H), 1.36 (s, 3H), 1.33 (s, 3H).

^{13}C NMR (151 MHz, Chloroform-*d*) δ 166.7, 157.6, 137.7, 137.7, 137.5, 134.7, 128.5, 128.5, 128.5, 127.9, 127.8, 127.8, 127.6, 118.7, 112.7, 109.0, 108.5, 84.0, 83.3, 81.6, 80.1, 75.8, 73.5, 73.3, 72.5, 72.0, 70.6, 68.9, 68.0, 67.0, 26.9, 26.3, 26.2, 25.2, 24.6.

HRMS (ESI) *m/z*: [M + Na]$^+$ Calcd for $C_{43}H_{51}NNaO_{10}$ 764.3405 found 764.3415.

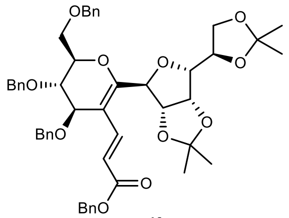

4f

benzyl (E)-3-((2R,3S,4R)-3,4-bis(benzyloxy)-2-((benzyloxy)methyl)-6-((3aS,4S,6R,6aS)-6-((R)-2,2-dimethyl-1,3-dioxolan-4-yl)-2,2-dimethyltetrahydrofuro[3,4-d][1,3]dioxol-4-yl)-3,4-dihydro-2H-pyran-5-yl)acrylate

Colorless liquid, 49.1 mg, 60%. Purified by chromatography on silicagel, eluting with petroleum ether/ethyl acetate 8:1-4:1 (v/v). $[\alpha]_D^{25}$=74.0 (c=5 mg/ml, CH_2Cl_2).

^{1}H NMR (600 MHz, Chloroform-*d*) δ 7.77 (d, *J* = 15.6 Hz, 1H), 7.37 – 7.29 (m, 15H), 7.27 – 7.25 (m, 3H), 7.19 – 7.17 (m, 2H), 5.81 (d, *J* = 15.6 Hz, 1H), 5.18 (s, 2H), 5.13 (s, 1H) **(anomeric H),** 4.91 (d, *J* = 6.0 Hz, 1H), 4.81 (dd, *J* = 5.9, 4.1 Hz, 1H), 4.67 – 4.63 (m, 2H), 4.49 – 4.45 (m, 2H), 4.44 – 4.43 (m, 1H), 4.42 – 4.40 (m, 2H), 4.34 – 4.31 (m, 1H), 4.24 (d, *J* = 2.3 Hz, 1H), 4.08 – 4.05 (m, 2H), 4.00 (dd, *J* = 8.7, 4.8 Hz, 1H), 3.95 (t, *J* = 3.8 Hz, 1H), 3.76 (dd, *J* = 10.5, 7.2 Hz, 1H), 3.57 (dd, *J* = 10.5, 4.1 Hz, 1H), 1.52 (s, 3H), 1.39 (s, 3H), 1.37 (s, 3H), 1.32 (s, 3H).

^{13}C NMR (151 MHz, Chloroform-*d*) δ 167.0, 158.3, 139.7, 137.6, 137.5, 137.0, 136.4, 128.5, 128.5, 128.4, 128.4, 128.1, 128.1, 128.0, 127.8, 127.7, 127.6, 115.1, 112.7, 109.1, 108.6, 84.0, 83.4, 81.5, 80.1, 75.8, 73.4, 73.3, 71.7, 71.6, 70.2, 70.2, 68.0, 67.1, 65.8, 26.8, 26.1, 25.2, 24.5.

HRMS (ESI) *m/z*: [M + Na]$^+$ Calcd for $C_{49}H_{54}NaO_{11}$ 841.3558 found 841.3555.

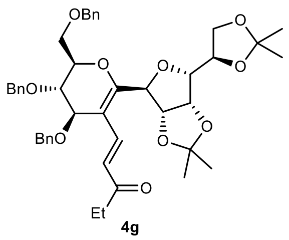
4g

(E)-1-((2R,3S,4R)-3,4-bis(benzyloxy)-2-((benzyloxy)methyl)-6-((3aS,4S,6R,6aS)-6-((R)-2,2-dimethyl-1,3-dioxolan-4-yl)-2,2-dimethyltetrahydrofuro[3,4-d][1,3]dioxol-4-yl)-3,4-dihydro-2H-pyran-5-yl)pent-1-en-3-one

Colorless liquid, 44.4 mg, 60%. Purified by chromatography on silicagel, eluting with petroleum ether/ethyl acetate 8:1-4:1 (v/v). $[\alpha]_D^{25}$=152.0 (c=5 mg/ml, CH_2Cl_2).

^{1}H NMR (600 MHz, Chloroform-*d*) δ 7.59 (d, *J* = 15.7 Hz, 1H), 7.39 – 7.25 (m, 13H), 7.22 – 7.18 (m, 2H), 6.05 (d, *J* = 15.7 Hz, 1H), 5.14 (s, 1H) **(anomeric H)**, 4.93 (d, *J* = 6.0 Hz, 1H), 4.83 – 4.79 (m, 1H), 4.71 – 4.63 (m, 2H), 4.50 – 4.40 (m, 5H), 4.36 – 4.31 (m, 1H), 4.27 – 4.23 (m, 1H), 4.09 – 4.05 (m, 2H), 4.01 – 3.96 (m, 2H), 3.77 (dd, *J* = 10.5, 7.1 Hz, 1H), 3.59 (dd, *J* = 10.5, 4.1 Hz, 1H), 2.48 (q, *J* = 7.3 Hz, 2H), 1.53 (s, 3H), 1.40 (s, 3H), 1.37 (s, 3H), 1.32 (s, 3H), 1.07 (t, *J* = 7.3 Hz, 3H).

^{13}C NMR (151 MHz, Chloroform-*d*) δ 200.5, 158.6, 137.6, 137.5, 137.1, 137.0, 128.6, 128.5, 128.5, 128.2, 128.1, 128.0, 127.9, 127.7, 127.6, 123.7, 112.7, 109.1, 108.7, 83.9, 83.3, 81.5, 80.1, 75.8, 73.4, 73.3, 71.8, 71.5, 70.1, 68.0, 67.0, 33.8, 26.8, 26.1, 25.2, 24.5, 8.3.

HRMS (ESI) *m/z*: [M + Na]$^+$ Calcd for $C_{44}H_{52}NaO_{10}$ 763.3453 found 763.3430.

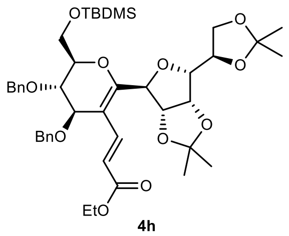
4h

ethyl (E)-3-((2R,3S,4R)-3,4-bis(benzyloxy)-2-(((tert-butyldimethylsilyl)oxy)methyl)-6-((3aS,4S,6R,6aS)-6-((R)-2,2-dimethyl-1,3-dioxolan-4-yl)-2,2-dimethyltetrahydrofuro[3,4-d][1,3]dioxol-4-yl)-3,4-dihydro-2H-pyran-5-yl)acrylate

Colorless liquid, 12.5 mg, 16%. Purified by chromatography on silicagel, eluting with petroleum ether/ethyl acetate 8:1-4:1 (v/v). $[\alpha]_D^{25}$=44.0 (c=5 mg/ml, CH_2Cl_2).

^1H NMR (400 MHz, Chloroform-*d*) δ 7.69 (d, *J* = 15.6 Hz, 1H), 7.40 – 7.28 (m, 8H), 7.26 – 7.22 (m, 2H), 5.82 (d, *J* = 15.6 Hz, 1H), 5.15 (s, 1H) **(anomeric H)**, 4.94 (d, *J* = 6.1 Hz, 1H), 4.89 – 4.83 (m, 1H), 4.70 (d, *J* = 1.8 Hz, 2H), 4.51 – 4.43 (m, 2H), 4.34 (dt, *J* = 14.4, 4.8 Hz, 2H), 4.19 (q, *J* = 7.1 Hz, 3H), 4.11 – 4.06 (m, 2H), 4.05 – 3.99 (m, 2H), 3.92 (dd, *J* = 11.3, 5.8 Hz, 1H), 3.71 (dd, *J* = 11.2, 3.9 Hz, 1H), 1.54 (s, 3H), 1.43 (s, 3H), 1.38 (s, 3H), 1.34 (s, 3H), 1.28 (t, *J* = 7.1 Hz, 3H), 0.89 (s, 9H), 0.05 (s, 3H), 0.03 (s, 3H).

^{13}C NMR (151 MHz, Chloroform-*d*) δ 167.2, 158.4, 139.0, 137.7, 137.4, 128.6, 128.5, 128.0, 128.0, 127.9, 127.7, 116.0, 112.7, 109.1, 109.0, 83.9, 83.4, 81.6, 80.1, 78.0, 73.5, 72.6, 72.2, 70.6, 70.0, 67.1, 61.1, 60.1, 26.9, 26.1, 25.8, 25.8, 25.2, 24.4, 18.2, 14.4, -5.4, -5.5.

HRMS (ESI) *m/z*: $[M + Na]^+$ Calcd for $C_{43}H_{60}NaO_{11}Si$ 803.3797 found 803.3785.

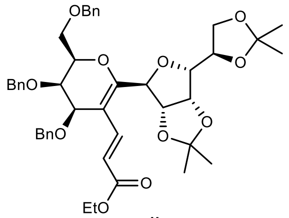

4i

ethyl (E)-3-((2R,3R,4R)-3,4-bis(benzyloxy)-2-((benzyloxy)methyl)-6-((3aS,4S,6R,6aS)-6-((R)-2,2-dimethyl-1,3-dioxolan-4-yl)-2,2-dimethyltetrahydrofuro[3,4-d][1,3]dioxol-4-yl)-3,4-dihydro-2H-pyran-5-yl)acrylate

Colorless liquid, 44.6 mg, 59%. Purified by chromatography on silicagel, eluting with petroleum ether/ethyl acetate 8:1-4:1 (v/v). $[\alpha]_D^{25}$=74.0 (c=5 mg/ml, CH_2Cl_2).

^1H NMR (600 MHz, Chloroform-*d*) δ 7.62 (d, *J* = 15.6 Hz, 1H), 7.39 – 7.36 (m, 2H), 7.35 – 7.29 (m, 10H), 7.28 – 7.27 (m, 3H), 5.76 (d, *J* = 15.5 Hz, 1H), 5.10 (s, 1H) **(anomeric H)**, 5.01 (d, *J* = 10.7 Hz, 1H), 4.82 – 4.78 (d, 2H), 4.76 – 4.70 (m, 2H), 4.66 – 4.57 (m, 2H), 4.46 (s, 2H), 4.34 – 4.30 (m, 2H), 4.19 (q, *J* = 7.1 Hz, 2H), 4.14 (dd, *J* = 7.4, 3.4 Hz, 1H), 4.08 – 4.00 (m, 3H), 3.96 (dd, *J* = 11.2, 9.1 Hz, 1H), 3.86 – 3.82 (m, 1H), 1.50 (s, 3H), 1.44 (s, 3H), 1.36 (s, 3H), 1.31 – 1.25 (m, 6H).

^{13}C NMR (151 MHz, Chloroform-*d*) δ 167.0, 156.7, 139.1, 137.9, 137.9, 137.5, 128.6, 128.4, 128.3, 128.1, 128.0, 127.8, 127.7, 127.7, 127.5, 115.5, 112.7, 110.1, 109.0, 84.3, 83.5, 81.6, 80.1, 75.3, 74.6, 74.5, 73.7, 73.3, 72.4, 69.4, 68.0, 66.7, 60.1, 26.8, 26.1, 25.1, 24.3, 14.4.

HRMS (ESI) *m/z*: $[M + Na]^+$ Calcd for $C_{44}H_{52}NaO_{11}$ 779.3402 found 779.3388.

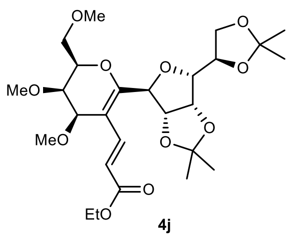

4j

ethyl (E)-3-((2R,3R,4R)-6-((3aS,4S,6R,6aS)-6-((R)-2,2-dimethyl-1,3-dioxolan-4-yl)-2,2-dimethyltetrahydrofuro[3,4-d][1,3]dioxol-4-yl)-3,4-dimethoxy-2-(methoxymethyl)-3,4-dihydro-2H-pyran-5-yl)acrylate

Colorless liquid, 37.8 mg, 64 %. Purified by chromatography on silicagel, eluting with petroleum ether/ethyl acetate 3:1-1:1 (v/v). $[\alpha]_D^{25}$=102.0 (c=5 mg/ml, CH_2Cl_2).

^{1}H NMR (600 MHz, Chloroform-*d*) δ 7.71 (d, *J* = 15.6 Hz, 1H), 7.41 – 7.34 (m, 4H), 7.34 – 7.30 (m, 1H), 5.85 (d, *J* = 15.5 Hz, 1H), 5.20 (q, *J* = 12.4 Hz, 2H), 5.10 (s, 1H) **(anomeric H)**, 4.87 – 4.82 (m, 2H), 4.42 (dt, *J* = 7.3, 3.0 Hz, 1H), 4.35 – 4.30 (m, 1H), 4.12 – 4.08 (m, 2H), 4.06 (dd, *J* = 8.7, 6.3 Hz, 1H), 4.02 (dd, *J* = 8.7, 4.7 Hz, 1H), 3.77 (t, *J* = 4.1 Hz, 1H), 3.73 (dd, *J* = 11.2, 8.5 Hz, 1H), 3.64 (dd, *J* = 11.2, 3.0 Hz, 1H), 3.57 (s, 3H), 3.54 (s, 3H), 3.38 (s, 3H), 1.51 (s, 3H), 1.44 (s, 3H), 1.37 (s, 3H), 1.33 (s, 3H).

^{13}C NMR (151 MHz, Chloroform-*d*) δ 167.0, 157.5, 139.9, 136.3, 128.5, 128.2, 128.1, 115.0, 112.7, 109.8, 109.0, 84.2, 83.4, 81.6, 80.1, 75.7, 75.4, 73.7, 71.2, 69.9, 66.7, 66.0, 59.8, 59.0, 58.8, 26.9, 26.1, 25.1, 24.4.

HRMS (ESI) *m/z*: $[M + Na]^+$ Calcd for $C_{31}H_{42}NaO_{11}$ 613.2619 found 613.2615.

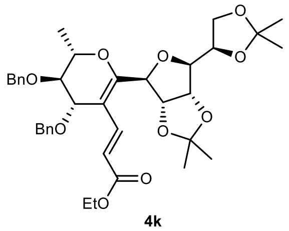

4k

ethyl (E)-3-((2S,3S,4S)-3,4-bis(benzyloxy)-6-((3aS,4S,6S,6aS)-6-((R)-2,2-dimethyl-1,3-dioxolan-4-yl)-2,2-dimethyltetrahydrofuro[3,4-d][1,3]dioxol-4-yl)-2-methyl-3,4-dihydro-2H-pyran-5-yl)acrylate

Colorless liquid, 32.5 mg, 50%. Purified by chromatography on silicagel, eluting with petroleum ether/ethyl acetate 8:1-4:1 (v/v). $[\alpha]_D^{25}$=70.0 (c=5 mg/ml, CH_2Cl_2).

^{1}H NMR (600 MHz, Chloroform-*d*) δ 7.71 (d, *J* = 15.6 Hz, 1H), 7.39 – 7.36 (m, 2H), 7.34 – 7.31 (m, 5H), 7.30 – 7.28 (m, 1H), 7.26 – 7.23 (m, 2H), 5.77 (d, *J* = 15.6 Hz, 1H), 5.15 (s, 1H)

(anomeric H), 4.93 (d, *J* = 6.0 Hz, 1H), 4.84 (dd, *J* = 5.9, 4.0 Hz, 1H), 4.68 – 4.64 (m, 2H), 4.49 – 4.44 (m, 2H), 4.41 – 4.37 (m, 1H), 4.34 (ddd, *J* = 7.9, 6.2, 4.7 Hz, 1H), 4.24 – 4.17 (m, 3H), 4.09 – 4.05 (m, 2H), 3.99 (dd, *J* = 8.7, 4.6 Hz, 1H), 3.69 (t, *J* = 3.2 Hz, 1H), 1.54 (s, 3H), 1.42 (s, 3H), 1.38 – 1.34 (m, 9H), 1.29 (t, *J* = 7.1 Hz, 3H).

^{13}C NMR (151 MHz, Chloroform-*d*) δ 167.2, 157.2, 139.4, 137.6, 137.4, 128.6, 128.5, 128.1, 128.0, 127.9, 127.7, 115.5, 112.7, 109.1, 108.3, 83.8, 83.4, 81.6, 80.1, 73.5, 73.5, 73.4, 71.6, 71.4, 70.2, 67.1, 60.1, 26.9, 26.1, 25.2, 24.5, 16.5, 14.4.

HRMS (ESI) *m/z*: [M + Na]$^+$ Calcd for $C_{37}H_{46}NaO_{10}$ 673.2983 found 673.2972.

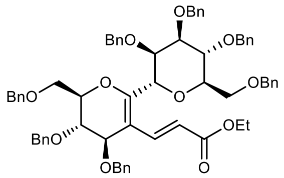

4l

ethyl (E)-3-((2R,3S,4R)-3,4-bis(benzyloxy)-2-((benzyloxy)methyl)-6-((2S,3S,4S,5R,6R)-3,4,5-tris(benzyloxy)-6-((benzyloxy)methyl)tetrahydro-2H-pyran-2-yl)-3,4-dihydro-2H-pyran-5-yl)acrylate

Yellow liquid, 41.4 mg, 40%. Purified by chromatography on silicagel, eluting with petroleum ether/ethyl acetate 8:1-5:1 (v/v). $[\alpha]_D^{25}$=39.0 (c=10 mg/ml, CH_2Cl_2).

^{1}H NMR (600 MHz, Chloroform-*d*) δ 7.88 (d, *J* = 15.7 Hz, 1H), 7.32 – 7.20 (m, 33H), 7.17 – 7.15 (m, 2H), 5.77 (d, *J* = 15.7 Hz, 1H), 5.12 (d, *J* = 4.7 Hz, 1H) **(anomeric H)**, 4.70 (d, *J* = 11.4 Hz, 1H), 4.64 (d, *J* = 12.1 Hz, 1H), 4.61 – 4.56 (m, 5H), 4.54 – 4.48 (m, 3H), 4.37 – 4.32 (m, 5H), 4.29 (d, *J* = 2.8 Hz, 1H), 4.18 – 4.13 (m, 3H), 3.99 (t, *J* = 4.0 Hz, 1H), 3.96 – 3.93 (m, 2H), 3.85 (s, 1H), 3.79 (dd, *J* = 10.8, 5.1 Hz, 1H), 3.71 (dd, *J* = 10.8, 3.5 Hz, 1H), 3.64 (d, *J* = 5.4 Hz, 2H), 1.25 (t, *J* = 7.1 Hz, 3H).

^{13}C NMR (151 MHz, Chloroform-*d*) δ 167.2, 157.3, 139.6, 138.5, 138.5, 138.4, 138.2, 137.8, 137.6, 137.3, 128.5, 128.3, 128.2, 128.2, 128.2, 128.0, 128.0, 127.9, 127.8, 127.7, 127.7, 127.5, 115.1, 109.4, 75.9, 75.3, 74.7, 73.9, 73.6, 73.3, 73.2, 72.1, 72.1, 71.7, 69.8, 69.6, 68.8, 67.8, 59.9, 14.4.

HRMS (ESI) *m/z*: [M + Na]$^+$ Calcd for $C_{66}H_{68}NaO_{11}$ 1059.4654 found 1059.4634.

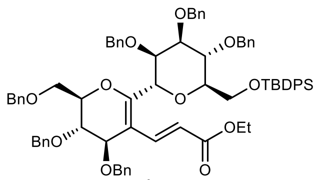

4m

ethyl (E)-3-((2R,3S,4R)-3,4-bis(benzyloxy)-2-((benzyloxy)methyl)-6-((2S,3S,4S,5R,6R)-3,4,5-tris(benzyloxy)-6-(((tert-butyldiphenylsilyl)oxy)methyl)tetrahydro-2H-pyran-2-yl)-3,4-dihydro-2H-pyran-5-yl)acrylate

Colorless liquid, 53.3 mg, 45%. Purified by chromatography on silicagel, eluting with petroleum ether/ethyl acetate 8:1-5:1 (v/v). $[\alpha]_D^{25}$=40.0 (c=5 mg/ml, CH_2Cl_2).

^{1}H NMR (600 MHz, Chloroform-*d*) δ 7.79 (d, *J* = 15.7 Hz, 1H), 7.66 (dd, *J* = 19.8, 7.8 Hz, 4H), 7.40 – 7.35 (m, 2H), 7.34 – 7.29 (m, 6H), 7.27 – 7.24 (m, 11H), 7.24 – 7.13 (m, 17H), 5.73 (d, *J* = 15.7 Hz, 1H), 5.08 (d, *J* = 4.4 Hz, 1H) **(anomeric H)**, 4.74 (d, *J* = 11.3 Hz, 1H), 4.64 – 4.55 (m, 7H), 4.37 – 4.31 (m, 5H), 4.25 (s, 1H), 4.13 – 4.01 (m, 5H), 4.00 – 3.96 (m, 2H), 3.87 (dd, *J* = 10.7, 4.7 Hz, 1H), 3.78 – 3.73 (m, 1H), 3.61 (d, *J* = 5.7 Hz, 2H), 1.15 (t, *J* = 7.1 Hz, 3H), 1.01 (s, 9H).

^{13}C NMR (151 MHz, Chloroform-*d*) δ 167.1, 157.7, 139.6, 138.6, 138.5, 138.4, 137.8, 137.6, 137.4, 135.8, 135.6, 133.8, 133.4, 129.5, 129.4, 128.5, 128.4, 128.3, 128.2, 128.2, 128.0, 127.9, 127.9, 127.8, 127.8, 127.6, 127.6, 127.5, 127.5, 115.1, 109.2, 76.5, 75.9, 74.6, 74.3, 73.7, 73.2, 72.2, 72.1, 71.8, 71.7, 69.7, 67.9, 62.8, 59.8, 26.8, 19.2, 14.4.

HRMS (ESI) m/z: $[M + H]^+$ Calcd for $C_{75}H_{81}O_{11}Si$ 1185.5543; Found 1185.5525.

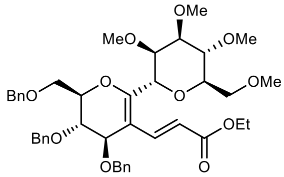

4n

ethyl (E)-3-((2R,3S,4R)-3,4-bis(benzyloxy)-2-((benzyloxy)methyl)-6-((2S,3S,4S,5R,6R)-3,4,5-trimethoxy-6-(methoxymethyl)tetrahydro-2H-pyran-2-yl)-3,4-dihydro-2H-pyran-5-yl)acrylate

Colorless liquid, 46.1 mg, 63%. Purified by chromatography on silicagel, eluting with petroleum ether/ethyl acetate 3:1-1:1 (v/v). $[\alpha]_D^{25}$=80.0 (c=5 mg/ml, CH_2Cl_2).

^{1}H NMR (600 MHz, Chloroform-*d*) δ 7.84 (d, *J* = 15.7 Hz, 1H), 7.33 – 7.28 (m, 13H), 7.20 (dd, *J* = 7.3, 1.9 Hz, 2H), 5.77 (d, *J* = 15.7 Hz, 1H), 5.06 (d, *J* = 4.2 Hz, 1H) **(anomeric H)**, 4.66 (d, *J* = 3.9 Hz, 1H), 4.47 (s, 2H), 4.43 (s, 2H), 4.30 (d, *J* = 2.5 Hz, 1H), 4.19 (q, *J* = 7.1 Hz, 2H), 4.01 (t, *J* = 3.9 Hz, 1H), 3.87 – 3.85 (m, 1H), 3.80 – 3.77 (m, 1H), 3.75 – 3.74 (m, 1H), 3.67 – 3.65 (m, 1H), 3.62 – 3.59 (m, 1H), 3.56 – 3.55 (m, 1H), 3.50 – 3.48 (m, 5H), 3.45 – 3.44 (m, 4H), 3.43 – 3.42 (m, 4H), 3.36 (s, 3H), 1.28 (d, *J* = 7.1 Hz, 3H).

^{13}C NMR (151 MHz, Chloroform-*d*) δ 167.1, 157.1, 139.4, 137.7, 137.6, 137.3, 128.5, 128.4, 128.3, 128.0, 127.9, 127.8, 127.7, 127.7, 127.5, 115.2, 109.3, 80.1, 77.2, 76.3, 76.1, 76.0, 74.7, 73.2, 71.7, 71.0, 70.1, 70.0, 69.9, 67.9, 59.9, 59.5, 59.1, 58.2, 57.9, 14.4.

HRMS (ESI) *m/z*: [M + Na]$^+$ Calcd for $C_{42}H_{52}NaO_{11}$ 755.3402 found 755.3410.

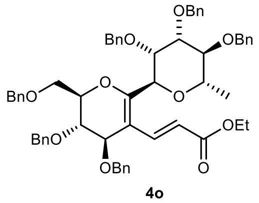

4o

ethyl (E)-3-((2R,3S)-3,4-bis(benzyloxy)-2-((benzyloxy)methyl)-6-((2R,3R,4R,5S,6S)-3,4,5-tris(benzyloxy)-6-methyltetrahydro-2H-pyran-2-yl)-3,4-dihydro-2H-pyran-5-yl)acrylate

Colorless liquid, 49.3 mg, 53%. Purified by chromatography on silicagel, eluting with petroleum ether/ethyl acetate 8:1-5:1 (v/v). $[\alpha]_D^{25}$=-10.0 (c=1 mg/ml, CH_2Cl_2).

^{1}H NMR (600 MHz, Chloroform-*d*) δ 7.94 (d, *J* = 15.7 Hz, 1H), 7.34 – 7.26 (m, 17H), 7.26 – 7.17 (m, 13H), 5.76 (d, *J* = 15.7 Hz, 1H), 5.10 (d, *J* = 4.2 Hz, 1H) **(anomeric H)**, 4.74 (d, *J* = 11.3 Hz, 1H), 4.68 (d, *J* = 12.1 Hz, 1H), 4.62 – 4.59 (m, 3H), 4.58 – 4.49 (m, 3H), 4.46 – 4.38 (m, 4H), 4.37 – 4.34 (m, 1H), 4.26 – 4.23 (m, 1H), 4.21 – 4.16 (m, 3H), 3.97 – 3.92 (m, 2H), 3.76 (p, *J* = 6.3 Hz, 1H), 3.71 (dd, *J* = 10.5, 7.1 Hz, 1H), 3.60 (dd, *J* = 10.5, 4.5 Hz, 1H), 3.56 (t, *J* = 7.5 Hz, 1H), 1.31 (d, *J* = 6.3 Hz, 3H), 1.27 (t, *J* = 7.1 Hz, 3H).

^{13}C NMR (151 MHz, Chloroform-*d*) δ 167.3, 157.4, 140.0, 138.7, 138.5, 138.4, 137.8, 137.5, 137.3, 128.5, 128.4, 128.3, 128.3, 128.2, 128.0, 127.9, 127.9, 127.7, 127.7, 114.9, 109.1, 80.4, 79.2, 74.6, 74.1, 73.4, 72.6, 72.2, 71.8, 71.7, 71.5, 71.3, 70.0, 69.9, 68.3, 60.0, 18.2, 14.4, 14.1.

HRMS (ESI) *m/z*: [M + Na]$^+$ Calcd for $C_{59}H_{62}NaO_{10}$ 953.4235 found 953.4242.

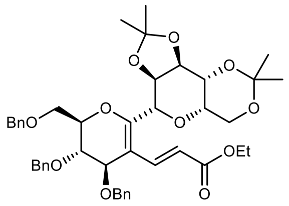

4p

ethyl (E)-3-((2R,3S,4R)-3,4-bis(benzyloxy)-2-((benzyloxy)methyl)-6-((3aS,4S,5aS,9aR,9bS)-2,2,8,8-tetramethylhexahydro-[1,3]dioxolo[4',5':4,5]pyrano[3,2-d][1,3]dioxin-4-yl)-3,4-dihydro-2H-pyran-5-yl)acrylate

White liquid, 46.9 mg, 62%. Purified by chromatography on silicagel, eluting with petroleum ether/ethyl acetate 8:1-4:1 (v/v). $[\alpha]_D^{25}$=100.0 (c=2 mg/ml, CH_2Cl_2). melting point: 58-60°C.

^{1}H NMR (600 MHz, Chloroform-*d*) δ 7.71 (d, *J* = 15.6 Hz, 1H), 7.36 – 7.33 (m, 3H), 7.31 – 7.26 (m, 10H), 7.21 (d, *J* = 6.5 Hz, 2H), 5.79 (d, *J* = 15.6 Hz, 1H), 5.14 (s, 1H) **(anomeric H)**, 4.92 (d, *J* = 5.9 Hz, 1H), 4.83 – 4.80 (m, 1H), 4.66 (s, 2H), 4.49 – 4.43 (m, 5H), 4.35 – 4.32 (m, 1H), 4.27 – 4.25 (m, 1H), 4.19 (q, *J* = 7.1 Hz, 2H), 4.09 – 4.06 (m, 2H), 4.01 (dd, *J* = 8.6, 4.8 Hz, 1H), 3.95 (t, *J* = 3.7 Hz, 1H), 3.76 (dd, *J* = 10.4, 7.2 Hz, 1H), 3.57 (dd, *J* = 10.5, 4.0 Hz, 1H), 1.53 (s, 3H), 1.40 (s, 3H), 1.37 (s, 3H), 1.32 (s, 3H), 1.28 (t, *J* = 7.1 Hz, 3H).

^{13}C NMR (151 MHz, Chloroform-*d*) δ 167.1, 157.9, 139.0, 137.6, 137.5, 137.2, 128.5, 128.4, 128.0, 128.0, 127.9, 127.8, 127.7, 127.6, 115.7, 112.7, 109.1, 108.7, 84.0, 83.4, 81.5, 80.1, 75.8, 73.5, 73.3, 71.8, 71.7, 70.3, 70.2, 68.0, 67.1, 60.0, 26.8, 26.1, 25.2, 24.5, 14.4.

HRMS (ESI) *m/z*: $[M + Na]^+$ Calcd for $C_{44}H_{52}NaO_{11}$ 779.3402 found 779.3385.

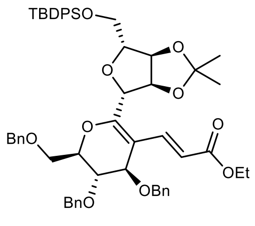

4q

ethyl (E)-3-((2R,3S,4R)-3,4-bis(benzyloxy)-2-((benzyloxy)methyl)-6-((3aR,4R,6R,6aR)-6-(((tert-butyldiphenylsilyl)oxy)methyl)-2,2-dimethyltetrahydrofuro[3,4-d][1,3]dioxol-4-yl)-3,4-dihydro-2H-pyran-5-yl)acrylate

Colorless liquid, 48.9 mg, 53%. Purified by chromatography on silicagel, eluting with petroleum ether/ethyl acetate 8:1-4:1 (v/v). $[\alpha]_D^{25}$=-22.0 (c=5 mg/ml, CH_2Cl_2).

^{1}H NMR (600 MHz, Chloroform-*d*) δ 7.76 (d, *J* = 15.6 Hz, 1H), 7.66 (d, *J* = 6.6 Hz, 4H), 7.35 – 7.30 (m, 11H), 7.26 – 7.22 (m, 6H), 7.17 – 7.13 (m, 2H), 7.09 – 7.05 (m, 2H), 5.73 (d, *J* = 15.6

Hz, 1H), 5.10 (d, *J* = 3.6 Hz, 1H) **(anomeric H)**, 4.82 (dd, *J* = 6.1, 3.8 Hz, 1H), 4.69 (dd, *J* = 6.2, 3.9 Hz, 1H), 4.62 (q, *J* = 12.2 Hz, 2H), 4.43 – 4.35 (m, 3H), 4.20 – 4.11 (m, 6H), 4.03 (t, *J* = 2.9 Hz, 1H), 3.80 – 3.74 (m, 2H), 3.62 (dd, *J* = 9.7, 7.0 Hz, 1H), 3.44 (dd, *J* = 9.8, 6.5 Hz, 1H), 1.59 (s, 3H), 1.34 (s, 3H), 1.27 (t, *J* = 7.1 Hz, 3H), 1.03 (s, 9H).

^{13}C NMR (151 MHz, Chloroform-*d*) δ 167.3, 156.1, 139.6, 137.9, 137.6, 137.2, 135.6, 135.6, 133.4, 133.3, 129.6, 129.6, 128.5, 128.3, 128.2, 128.0, 128.0, 127.9, 127.8, 127.7, 127.6, 127.5, 127.4, 115.1, 113.8, 108.9, 85.5, 82.8, 82.5, 80.0, 75.5, 73.0, 71.4, 70.6, 70.4, 69.3, 67.5, 64.1, 60.0, 27.6, 26.7, 25.6, 19.2, 14.4.

HRMS (ESI) *m/z*: [M + Na]$^+$ Calcd for $C_{56}H_{64}NaO_{10}Si$ 947.4161 found 947.4134.

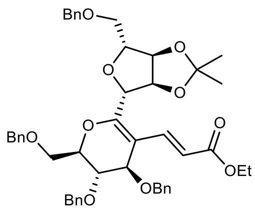

4r

ethyl (E)-3-((2R,3S,4R)-3,4-bis(benzyloxy)-2-((benzyloxy)methyl)-6-((3aR,4R,6R,6aR)-6-((benzyloxy)methyl)-2,2-dimethyltetrahydrofuro[3,4-d][1,3]dioxol-4-yl)-3,4-dihydro-2H-pyran-5-yl)acrylate

Colorless liquid, 52 mg, 67%. Purified by chromatography on silicagel, eluting with petroleum ether/ethyl acetate 8:1-4:1 (v/v). $[\alpha]_D^{25}$=-40.0 (c=5 mg/ml, CH_2Cl_2).

^{1}H NMR (600 MHz, Chloroform-*d*) δ 7.76 (d, *J* = 15.6 Hz, 1H), 7.38 – 7.34 (m, 2H), 7.33 – 7.23 (m, 14H), 7.20 (d, *J* = 6.8 Hz, 2H), 7.16 (dd, *J* = 6.4, 2.8 Hz, 2H), 5.72 (d, *J* = 15.6 Hz, 1H), 5.14 (d, *J* = 3.1 Hz, 1H) **(anomeric H)**, 4.83 (dd, *J* = 6.3, 3.2 Hz, 1H), 4.67 – 4.60 (m, 3H), 4.52 – 4.45 (m, 3H), 4.42 – 4.31 (m, 4H), 4.23 – 4.16 (m, 4H), 3.98 (t, *J* = 2.7 Hz, 1H), 3.67 – 3.61 (m, 2H), 3.60 – 3.55 (m, 2H), 1.58 (s, 3H), 1.33 (s, 3H), 1.28 (t, *J* = 7.1 Hz, 3H).

^{13}C NMR (151 MHz, Chloroform-*d*) δ 167.2, 155.9, 139.5, 138.1, 137.9, 137.6, 137.2, 128.6, 128.4, 128.3, 128.3, 128.0, 128.0, 127.9, 127.8, 127.6, 127.6, 127.5, 127.5, 115.2, 114.0, 108.9, 84.5, 83.2, 82.9, 80.2, 76.0, 73.3, 73.2, 71.4, 70.6, 70.5, 69.7, 68.0, 60.0, 27.5, 25.6, 14.4.

HRMS (ESI) *m/z*: [M + Na]$^+$ Calcd for $C_{47}H_{52}NaO_{10}$ 799.3453 found 799.3440.

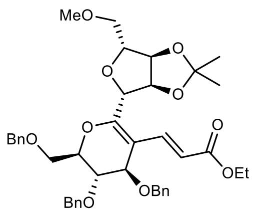

4s

ethyl (E)-3-((2R,3S,4R)-3,4-bis(benzyloxy)-2-((benzyloxy)methyl)-6-((3aR,4R,6R,6aR)-6-(methoxymethyl)-2,2-dimethyltetrahydrofuro[3,4-d][1,3]dioxol-4-yl)-3,4-dihydro-2H-pyran-5-yl)acrylate

Colorless liquid, 40.6 mg, 58%. Purified by chromatography on silicagel, eluting with petroleum ether/ethyl acetate 8:1-4:1 (v/v). $[\alpha]_D^{25}$=-24.0 (c=5 mg/ml, CH_2Cl_2).

^{1}H NMR (600 MHz, Chloroform-*d*) δ 7.75 (d, *J* = 15.6 Hz, 1H), 7.38 – 7.35 (m, 2H), 7.34 – 7.29 (m, 5H), 7.29 – 7.26 (m, 4H), 7.23 (d, *J* = 7.0 Hz, 2H), 7.17 (dd, *J* = 6.6, 2.6 Hz, 2H), 5.72 (d, *J* = 15.6 Hz, 1H), 5.14 (d, *J* = 3.0 Hz, 1H) **(anomeric H)**, 4.83 (dd, *J* = 6.4, 3.0 Hz, 1H), 4.65 (q, *J* = 12.1 Hz, 2H), 4.58 – 4.52 (m, 2H), 4.48 (d, *J* = 11.9 Hz, 1H), 4.43 – 4.38 (m, 3H), 4.22 – 4.14 (m, 4H), 3.98 (t, *J* = 2.8 Hz, 1H), 3.73 (dd, *J* = 10.5, 7.0 Hz, 1H), 3.64 (dd, *J* = 10.6, 5.1 Hz, 1H), 3.49 (dd, *J* = 5.8, 2.4 Hz, 2H), 3.28 (s, 3H), 1.58 (s, 3H), 1.33 (s, 3H), 1.29 (t, *J* = 7.1 Hz, 3H).

^{13}C NMR (151 MHz, Chloroform-*d*) δ 167.2, 155.8, 139.5, 137.9, 137.6, 137.2, 128.6, 128.4, 128.3, 128.0, 127.9, 127.9, 127.8, 127.6, 127.5, 115.2, 114.1, 108.9, 84.2, 83.2, 82.9, 80.2, 76.2, 73.3, 73.0, 71.4, 70.6, 70.5, 69.8, 68.1, 60.0, 59.3, 27.5, 25.5, 14.4.

HRMS (ESI) *m/z*: [M + Na]$^+$ Calcd for $C_{41}H_{48}NaO_{10}$ 723.3140 found 723.3134.

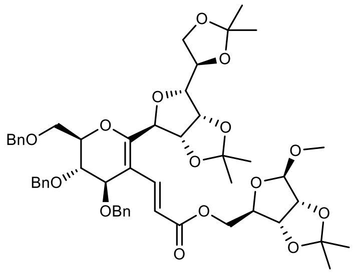

4v

((3aR,4R,6R,6aR)-6-methoxy-2,2-dimethyltetrahydrofuro[3,4-d][1,3]dioxol-4-yl)methyl (E)-3-((2R,3S,4R)-3,4-bis(benzyloxy)-2-((benzyloxy)methyl)-6-((3aS,4S,6R,6aS)-6-((R)-2,2-dimethyl-1,3-dioxolan-4-yl)-2,2-dimethyltetrahydrofuro[3,4-d][1,3]dioxol-4-yl)-3,4-dihydro-2H-pyran-5-yl)acrylate

Colorless liquid, 59.4 mg, 65%. Purified by chromatography on silicagel, eluting with petroleum ether/ethyl acetate 8:1-4:1 (v/v). $[\alpha]_D^{25}$=64.0 (c=5 mg/ml, CH_2Cl_2).

^{1}H NMR (600 MHz, Chloroform-*d*) δ 7.77 (d, *J* = 15.6 Hz, 1H), 7.38 – 7.30 (m, 12H), 7.27 – 7.26 (m, 1H), 7.21 – 7.19 (m, 2H), 5.78 (d, *J* = 15.5 Hz, 1H), <u>5.15 (s, 1H) **(anomeric H)**</u>, 4.99 (s, 1H), 4.91 (d, *J* = 6.0 Hz, 1H), 4.83 – 4.80 (m, 1H), 4.69 (d, *J* = 5.5 Hz, 1H), 4.67 (d, *J* = 6.0 Hz, 1H), 4.63 (d, *J* = 5.9 Hz, 1H), 4.49 – 4.40 (m, 7H), 4.35 – 4.31 (m, 1H), 4.26 – 4.23 (m, 1H), 4.20 – 4.15 (m, 2H), 4.10 – 4.06 (m, 2H), 4.01 (dd, *J* = 8.7, 4.8 Hz, 1H), 3.96 (t, *J* = 3.7 Hz, 1H), 3.76 (dd, *J* = 10.5, 7.2 Hz, 1H), 3.57 (dd, *J* = 10.5, 4.1 Hz, 1H), 3.31 (s, 3H), 1.53 (s, 3H), 1.49 (s, 3H), 1.40 (s, 3H), 1.37 (s, 3H), 1.34 – 1.30 (m, 6H).

^{13}C NMR (151 MHz, Chloroform-*d*) δ 166.7, 158.5, 140.1, 137.6, 137.5, 137.0, 128.6, 128.5, 128.4, 128.1, 128.0, 128.0, 127.8, 127.7, 127.6, 114.6, 112.7, 112.4, 109.4, 109.1, 108.6, 85.2, 84.3, 84.0, 83.4, 81.8, 81.6, 80.1, 75.8, 73.4, 73.3, 71.7, 71.5, 70.2, 70.1, 68.0, 67.1, 64.3, 54.8, 26.8, 26.4, 26.1, 25.2, 24.9, 24.6.

HRMS (ESI) *m/z*: [M + Na]$^+$ Calcd for $C_{51}H_{62}NaO_{15}$ 937.3981 found 937.3978.

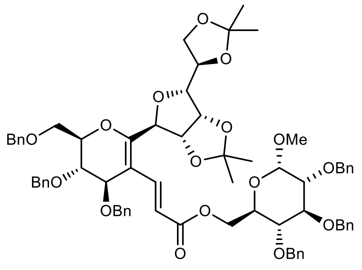

4w

((2R,3R,4S,5R,6S)-3,4,5-tris(benzyloxy)-6-methoxytetrahydro-2H-pyran-2-yl)methyl (E)-3-((2R,3S,4R)-3,4-bis(benzyloxy)-2-((benzyloxy)methyl)-6-((3aS,4S,6R,6aS)-6-((R)-2,2-dimethyl-1,3-dioxolan-4-yl)-2,2-dimethyltetrahydrofuro[3,4-d][1,3]dioxol-4-yl)-3,4-dihydro-2H-pyran-5-yl)acrylate

Colorless liquid, 72.8 mg, 62%. Purified by chromatography on silicagel, eluting with petroleum ether/ethyl acetate 8:1-4:1 (v/v). $[\alpha]_D^{25}$=94.0 (c=5 mg/ml, CH_2Cl_2).

^{1}H NMR (600 MHz, Chloroform-d) δ 7.73 (d, J = 15.5 Hz, 1H), 7.36 – 7.25 (m, 28H), 7.19 – 7.16 (m, 2H), 5.80 (d, J = 15.5 Hz, 1H), <u>5.11 (s, 1H) **(anomeric H)**</u>, 5.00 (d, J = 10.8 Hz, 1H), 4.91 (d, J = 6.0 Hz, 1H), 4.85 (dd, J = 18.8, 10.8 Hz, 2H), 4.82 – 4.77 (m, 2H), 4.67 – 4.64 (m, 3H), 4.60 – 4.56 (m, 2H), 4.48 – 4.44 (m, 2H), 4.43 – 4.38 (m, 4H), 4.33 – 4.27 (m, 2H), 4.25 (d, J =

2.9 Hz, 1H), 4.07 – 4.04 (m, 2H), 4.02 – 3.98 (m, 2H), 3.96 (t, J = 3.9 Hz, 1H), 3.85 (dd, J = 9.8, 4.0 Hz, 1H), 3.75 (dd, J = 10.5, 7.1 Hz, 1H), 3.54 (ddd, J = 20.2, 10.1, 3.7 Hz, 2H), 3.47 (t, J = 9.4 Hz, 1H), 3.35 (s, 3H), 1.50 (s, 3H), 1.39 (s, 3H), 1.35 (s, 3H), 1.30 (s, 3H).

^{13}C NMR (151 MHz, Chloroform-*d*) δ 166.8, 158.5, 139.9, 138.6, 138.0, 137.8, 137.6, 137.5, 137.1, 128.5, 128.5, 128.4, 128.4, 128.4, 128.0, 128.0, 128.0, 128.0, 127.9, 127.9, 127.9, 127.8, 127.7, 127.6, 127.6, 114.8, 112.7, 109.0, 108.7, 97.8, 83.9, 83.4, 82.0, 81.5, 80.1, 79.9, 79.6, 77.6, 75.9, 75.7, 75.1, 73.4, 73.3, 73.2, 71.8, 71.7, 70.2, 70.0, 68.8, 68.0, 67.0, 62.8, 55.1, 26.8, 26.1, 25.2, 24.5.

HRMS (ESI) *m/z*: [M + Na]$^+$ Calcd for $C_{70}H_{78}NaO_{16}$ 1197.5182 found 1197.5168.

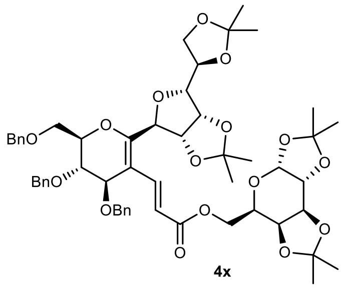

4x

((3aR,5R,5aS,8aS,8bR)-2,2,7,7-tetramethyltetrahydro-5H-bis([1,3]dioxolo)[4,5-b:4',5'-d]pyran-5-yl)methyl (E)-3-((2R,3S,4R)-3,4-bis(benzyloxy)-2-((benzyloxy)methyl)-6-((3aS,4S,6R,6aS)-6-((R)-2,2-dimethyl-1,3-dioxolan-4-yl)-2,2-dimethyltetrahydrofuro[3,4-d][1,3]dioxol-4-yl)-3,4-dihydro-2H-pyran-5-yl)acrylate

Yellow solid, 69.8 mg, 72%. Purified by chromatography on silicagel, eluting with petroleum ether/ethyl acetate 8:1-4:1 (v/v). $[\alpha]_D^{25}$=49.0 (c=10 mg/ml, CH_2Cl_2). melting point: 80-82°C.

^{1}H NMR (600 MHz, Chloroform-*d*) δ 7.74 (d, *J* = 15.6 Hz, 1H), 7.35 – 7.27 (m, 13H), 7.21 – 7.18 (m, 2H), 5.84 (d, *J* = 15.5 Hz, 1H), 5.55 (d, *J* = 4.9 Hz, 1H), 5.13 (s, 1H) **(anomeric H)**, 4.91 (d, *J* = 6.0 Hz, 1H), 4.81 (dd, *J* = 5.9, 4.1 Hz, 1H), 4.66 (s, 2H), 4.62 (dd, *J* = 7.9, 2.4 Hz, 1H), 4.46 (d, *J* = 5.7 Hz, 2H), 4.44 – 4.40 (m, 3H), 4.34 – 4.30 (m, 3H), 4.29 – 4.25 (m, 3H), 4.09 – 4.05 (m, 3H), 4.00 (dd, *J* = 8.7, 4.8 Hz, 1H), 3.95 (t, *J* = 3.8 Hz, 1H), 3.76 (dd, *J* = 10.6, 7.1 Hz, 1H), 3.56 (dd, *J* = 10.6, 4.0 Hz, 1H), 1.53 – 1.50 (m, 6H), 1.46 (s, 3H), 1.39 (s, 3H), 1.37 (s, 3H), 1.35 (s, 3H), 1.33 – 1.31 (m, 6H).

^{13}C NMR (151 MHz, Chloroform-*d*) δ 166.9, 158.3, 139.8, 137.6, 137.5, 137.1, 128.5, 128.5, 128.4, 128.0, 128.0, 127.9, 127.8, 127.7, 127.6, 115.0, 112.7, 109.5, 109.1, 108.7, 108.7, 96.2,

83.9, 83.4, 81.5, 80.0, 75.9, 73.4, 73.3, 71.7, 71.6, 71.0, 70.6, 70.5, 70.2, 70.1, 68.0, 67.1, 66.0, 63.1, 26.8, 26.1, 26.1, 25.9, 25.2, 25.0, 24.5, 24.5.

HRMS (ESI) *m/z*: [M + Na]$^+$ Calcd for $C_{54}H_{66}NaO_{16}$ 993.4243 found 993.4249.

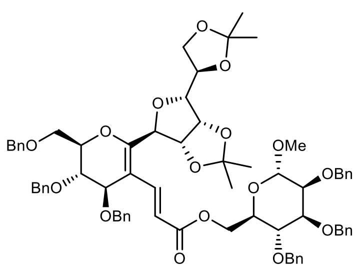

4y

((2R,3R,4S,5S,6S)-3,4,5-tris(benzyloxy)-6-methoxytetrahydro-2H-pyran-2-yl)methyl (E)-3-((2R,3S,4R)-3,4-bis(benzyloxy)-2-((benzyloxy)methyl)-6-((3aS,4S,6R,6aS)-6-((R)-2,2-dimethyl-1,3-dioxolan-4-yl)-2,2-dimethyltetrahydrofuro[3,4-d][1,3]dioxol-4-yl)-3,4-dihydro-2H-pyran-5-yl)acrylate

Yellow liquid, 81 mg, 69%. Purified by chromatography on silicagel, eluting with petroleum ether/ethyl acetate 8:1-4:1 (v/v). $[\alpha]_D^{25}$=67.0 (c=5 mg/ml, CH_2Cl_2).

^{1}H NMR (600 MHz, Chloroform-*d*) δ 7.74 (d, *J* = 15.6 Hz, 1H), 7.37 – 7.28 (m, 26H), 7.26 (s, 2H), 7.18 (d, *J* = 6.7 Hz, 2H), 5.84 (d, *J* = 15.5 Hz, 1H), 5.12 (s, 1H) **(anomeric H)**, 4.93 – 4.89 (m, 2H), 4.81 (dd, *J* = 5.9, 4.1 Hz, 1H), 4.73 – 4.70 (m, 3H), 4.65 (s, 2H), 4.61 – 4.58 (m, 3H), 4.48 – 4.45 (m, 3H), 4.41 (d, *J* = 13.4 Hz, 3H), 4.35 (dd, *J* = 11.8, 6.3 Hz, 1H), 4.29 (ddd, *J* = 7.9, 6.2, 4.9 Hz, 1H), 4.25 (d, *J* = 2.6 Hz, 1H), 4.07 – 4.02 (m, 2H), 3.98 (dd, *J* = 8.7, 4.8 Hz, 1H), 3.94 (t, *J* = 3.9 Hz, 1H), 3.91 – 3.88 (m, 2H), 3.83 – 3.80 (m, 1H), 3.78 – 3.74 (m, 2H), 3.55 (dd, *J* = 10.6, 4.0 Hz, 1H), 3.28 (s, 3H), 1.49 (s, 3H), 1.38 (s, 3H), 1.35 (s, 3H), 1.30 (s, 3H).

^{13}C NMR (151 MHz, Chloroform-*d*) δ 167.0, 158.3, 139.7, 138.4, 138.2, 138.1, 137.6, 137.5, 137.1, 128.6, 128.5, 128.5, 128.4, 128.3, 128.3, 128.1, 128.0, 127.9, 127.8, 127.7, 127.7, 127.6, 127.6, 115.1, 112.7, 109.1, 108.8, 98.8, 83.9, 83.4, 81.6, 80.2, 80.0, 75.9, 75.2, 74.8, 74.2, 73.4, 73.3, 72.5, 72.0, 71.7, 71.7, 70.3, 70.1, 68.1, 67.1, 63.6, 54.7, 26.9, 26.1, 25.2, 24.5.

HRMS (ESI) *m/z*: [M + Na]$^+$ Calcd for $C_{70}H_{78}NaO_{16}$ 1197.5182 found 1197.5173.

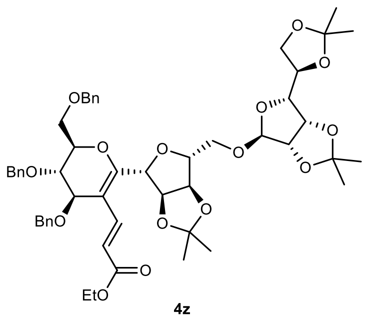

4z

ethyl (E)-3-((2R,3S,4R)-3,4-bis(benzyloxy)-2-((benzyloxy)methyl)-6-((3aR,4R,6R,6aR)-6-((((3aS,4S,6R,6aS)-6-((R)-2,2-dimethyl-1,3-dioxolan-4-yl)-2,2-dimethyltetrahydrofuro[3,4-d][1,3]dioxol-4-yl)oxy)methyl)-2,2-dimethyltetrahydrofuro[3,4-d][1,3]dioxol-4-yl)-3,4-di-hydro-2H-pyran-5-yl)acrylate

Colorless liquid, 61.2 mg, 66%. Purified by chromatography on silicagel, eluting with petroleum ether/ethyl acetate 8:1-3:1 (v/v). $[\alpha]_D^{25}$=-12.0 (c=5 mg/ml, CH_2Cl_2).

^{1}H NMR (600 MHz, Chloroform-*d*) δ 7.73 (d, *J* = 15.6 Hz, 1H), 7.36 – 7.26 (m, 13H), 7.17 – 7.14 (m, 2H), 5.72 (d, *J* = 15.5 Hz, 1H), 5.1 (d, J = 2.7 Hz, 1H) **(anomeric H)**, 5.02 (s, 1H), 4.84 – 4.82 (m, 1H), 4.75 – 4.73 (m, 1H), 4.65 – 4.63 (m, 2H), 4.62 – 4.59 (m, 2H), 4.57 – 4.55 (m, 1H), 4.49 (d, *J* = 11.9 Hz, 1H), 4.41 (s, 2H), 4.37 – 4.34 (m, 2H), 4.21 – 4.17 (m, 4H), 4.07 – 4.05 (m, 1H), 4.02 – 3.99 (m, 2H), 3.92 (dd, *J* = 7.5, 3.2 Hz, 1H), 3.71 – 3.65 (m, 3H), 3.62 – 3.59 (m, 1H), 1.59 (s, 3H), 1.44 (s, 3H), 1.41 (s, 3H), 1.36 (s, 3H), 1.34 (s, 3H), 1.30 – 1.27 (m, 6H).

^{13}C NMR (151 MHz, Chloroform-*d*) δ 167.2, 155.9, 139.4, 137.9, 137.6, 137.2, 128.5, 128.4, 128.3, 128.0, 128.0, 127.9, 127.8, 127.7, 127.6, 127.5, 115.3, 113.9, 112.5, 109.1, 108.9, 107.0, 85.0, 84.2, 83.2, 83.0, 80.5, 80.4, 79.5, 75.9, 73.2, 73.1, 71.4, 70.7, 70.4, 69.6, 67.9, 67.7, 67.0, 60.0, 27.5, 26.8, 25.9, 25.5, 25.2, 24.5, 14.4.

HRMS (ESI) *m/z*: $[M + Na]^+$ Calcd for $C_{52}H_{64}NaO_{15}$ 951.4137 found 951.4128.

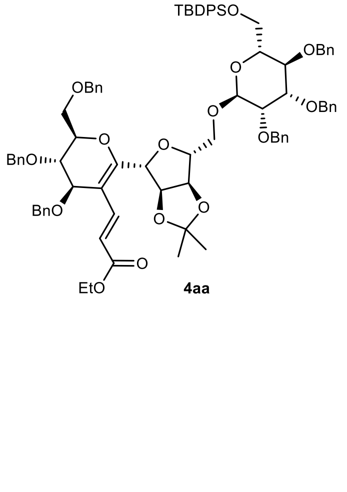

4aa

ethyl (E)-3-((2R,3S,4R)-3,4-bis(benzyloxy)-2-((benzyloxy)methyl)-6-((3aR,4R,6R,6aR)-2,2-dimethyl-6-((((2S,3S,4S,5R,6R)-3,4,5-tris(benzyloxy)-6-(((tert-butyldiphenylsilyl)oxy)methyl)tetrahydro-2H-pyran-2-yl)oxy)methyl)tetrahydrofuro[3,4-d][1,3]dioxol-4-yl)-3,4-dihydro-2H-pyran-5-yl)acrylate

Colorless liquid, 81.4 mg, 60%. Purified by chromatography on silicagel, eluting with petroleum ether/ethyl acetate 8:1-4:1 (v/v). $[\alpha]_D^{25}$=-11.0 (c=10 mg/ml, CH_2Cl_2).

^{1}H NMR (600 MHz, Chloroform-*d*) δ 7.8 (d, *J* = 15.6 Hz, 1H), 7.7 – 7.7 (m, 2H), 7.7 – 7.7 (m, 2H), 7.3 (dd, *J* = 13.1, 6.5 Hz, 9H), 7.3 – 7.3 (m, 13H), 7.2 – 7.2 (m, 9H), 7.2 – 7.1 (m, 2H), 7.1 – 7.1 (m, 3H), 5.7 (d, *J* = 15.6 Hz, 1H), 5.1 (d, *J* = 3.4 Hz, 1H) **(anomeric H)**, 4.9 – 4.9 (m, 2H), 4.8 – 4.8 (m, 1H), 4.8 – 4.7 (m, 1H), 4.7 – 4.6 (m, 4H), 4.6 (dd, *J* = 12.4, 7.3 Hz, 3H), 4.5 (dd, *J* = 6.3, 4.0 Hz, 2H), 4.4 (d, *J* = 11.0 Hz, 3H), 4.2 (d, *J* = 11.9 Hz, 1H), 4.2 – 4.1 (m, 4H), 4.1 (d, *J* = 9.5 Hz, 1H), 4.0 (t, *J* = 2.6 Hz, 1H), 3.9 – 3.9 (m, 2H), 3.9 – 3.9 (m, 1H), 3.8 – 3.8 (m, 1H), 3.7 (dd, *J* = 10.9, 5.4 Hz, 1H), 3.7 – 3.6 (m, 1H), 3.6 (d, *J* = 5.7 Hz, 1H), 3.6 – 3.6 (m, 1H), 1.5 (s, 3H), 1.3 (s, 3H), 1.3 (t, *J* = 7.3 Hz, 3H), 1.0 (s, 9H).

^{13}C NMR (151 MHz, Chloroform-*d*) δ 167.2, 155.7, 139.5, 138.7, 138.6, 137.8, 137.5, 137.1, 135.9, 135.6, 133.9, 133.4, 129.4, 128.6, 128.4, 128.3, 128.3, 128.2, 128.2, 128.0, 127.9, 127.8, 127.7, 127.6, 127.5, 127.4, 127.3, 115.2, 114.0, 108.9, 98.0, 83.7, 83.2, 83.0, 80.3, 80.2, 76.0, 75.6, 75.1, 74.6, 73.3, 73.2, 72.7, 72.2, 71.4, 70.6, 70.4, 69.6, 67.9, 67.3, 63.1, 60.0, 29.7, 27.5, 26.8, 25.6, 19.3, 14.4, 14.1.

HRMS (ESI) *m/z*: [M + Na]$^+$ Calcd for $C_{83}H_{92}NaO_{15}Si$ 1379.6098 found 1379.6085.

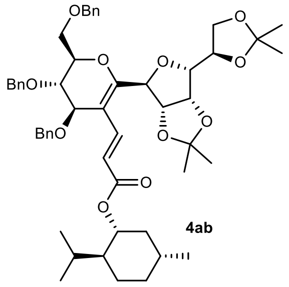

(1R,2S,5R)-2-isopropyl-5-methylcyclohexyl (E)-3-((2R,3S,4R)-3,4-bis(benzyloxy)-2-((benzyloxy)methyl)-6-((3aS,4S,6R,6aS)-6-((R)-2,2-dimethyl-1,3-dioxolan-4-yl)-2,2-dimethyltetrahydrofuro[3,4-d][1,3]dioxol-4-yl)-3,4-dihydro-2H-pyran-5-yl)acrylate

Colorless liquid, 48.5 mg, 56%. Purified by chromatography on silicagel, eluting with petroleum ether/ethyl acetate 8:1-4:1 (v/v). $[\alpha]_D^{25}$=71.0 (c=10 mg/ml, CH_2Cl_2).

^{1}H NMR (600 MHz, Chloroform-*d*) δ 7.71 (d, *J* = 15.6 Hz, 1H), 7.36 – 7.26 (m, 13H), 7.22 – 7.19 (m, 2H), 5.82 (d, *J* = 15.5 Hz, 1H), 5.15 (s, 1H) **(anomeric H)**, 4.91 (d, *J* = 6.0 Hz, 1H), 4.83 – 4.80 (m, 1H), 4.76 (dt, *J* = 10.9, 5.4 Hz, 1H), 4.68 (s, 2H), 4.50 – 4.44 (m, 2H), 4.44 – 4.39 (m, 3H), 4.35 – 4.31 (m, 1H), 4.28 (d, *J* = 3.0 Hz, 1H), 4.10 – 4.06 (m, 2H), 4.03 – 3.97 (m, 2H), 3.76 (dd, *J* = 10.5, 7.0 Hz, 1H), 3.59 (dd, *J* = 10.5, 4.1 Hz, 1H), 2.01 (d, *J* = 11.4 Hz, 1H), 1.92 – 1.86 (m, 1H), 1.71 – 1.66 (m, 2H), 1.55 – 1.49 (m, 4H), 1.43 – 1.39 (m, 4H), 1.37 (s, 3H), 1.32 (s, 3H), 1.08 (qd, *J* = 13.7, 3.9 Hz, 1H), 1.01 – 0.96 (m, 1H), 0.92 – 0.86 (m, 7H), 0.76 (d, *J* = 6.9 Hz, 3H).

^{13}C NMR (151 MHz, Chloroform-*d*) δ 166.7, 158.0, 138.9, 137.6, 137.5, 137.1, 128.5, 128.4, 128.2, 128.0, 128.0, 127.8, 127.7, 127.6, 116.1, 112.7, 109.1, 108.7, 84.0, 83.4, 81.6, 80.0, 75.8, 73.7, 73.5, 73.3, 72.1, 71.7, 70.1, 70.1, 68.0, 67.1, 47.1, 41.0, 34.3, 31.3, 26.8, 26.2, 26.1, 25.2, 24.6, 23.5, 22.0, 20.8, 16.4.

HRMS (ESI) *m/z*: [M + Na]$^+$ Calcd for $C_{52}H_{66}NaO_{11}$ 889.4497 found 889.4480.

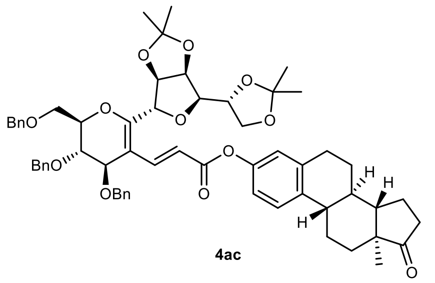

4ac

(8R,9S,13S,14S)-13-methyl-17-oxo-7,8,9,11,12,13,14,15,16,17-decahydro-6H-cyclopenta[a]phenanthren-3-yl (E)-3-((2R,3S,4R)-3,4-bis(benzyloxy)-2-((benzyloxy)methyl)-6-((3aS,4S,6R,6aS)-6-((R)-2,2-dimethyl-1,3-dioxolan-4-yl)-2,2-dimethyltetrahydrofuro[3,4-d][1,3]dioxol-4-yl)-3,4-dihydro-2H-pyran-5-yl)acrylate

Colorless liquid, 41.1 mg, 42%. Purified by chromatography on silicagel, eluting with petroleum ether/ethyl acetate 8:1-4:1 (v/v). $[\alpha]_D^{25}$=110.0 (c=10 mg/ml, CH_2Cl_2).

^{1}H NMR (600 MHz, Chloroform-*d*) δ 7.88 (d, *J* = 15.6 Hz, 1H), 7.39 – 7.36 (m, 2H), 7.34 – 7.29 (m, 10H), 7.28 – 7.26 (m, 2H), 7.24 – 7.22 (m, 2H), 6.88 (dd, *J* = 8.4, 2.2 Hz, 1H), 6.85 – 6.82 (m, 1H), 5.90 (d, *J* = 15.5 Hz, 1H), 5.15 (s, 1H) **(anomeric H)**, 4.94 (d, *J* = 6.0 Hz, 1H), 4.83 (dd, *J* = 5.9, 4.1 Hz, 1H), 4.70 – 4.66 (m, 2H), 4.51 – 4.47 (m, 5H), 4.35 – 4.32 (m, 1H), 4.30 – 4.28 (m, 1H), 4.09 – 4.07 (m, 2H), 4.02 (dd, *J* = 8.7, 4.7 Hz, 1H), 3.98 (t, *J* = 3.6 Hz, 1H), 3.78 (dd, *J* = 10.5, 7.2 Hz, 1H), 3.59 (dd, *J* = 10.5, 4.2 Hz, 1H), 2.94 – 2.90 (m, 2H), 2.53 – 2.48 (m,

1H), 2.43 – 2.40 (m, 1H), 2.30 (td, *J* = 11.6, 11.1, 4.1 Hz, 1H), 2.17 – 2.13 (m, 1H), 2.08 (d, *J* = 4.3 Hz, 1H), 1.99 – 1.96 (m, 1H), 1.64 – 1.62 (m, 1H), 1.62 – 1.59 (m, 1H), 1.53 – 1.51 (m, 4H), 1.49 (d, *J* = 4.1 Hz, 1H), 1.47 – 1.45 (m, 2H), 1.41 (s, 3H), 1.38 – 1.35 (m, 4H), 1.32 (s, 3H), 0.91 (s, 3H).

^{13}C NMR (151 MHz, Chloroform-*d*) δ 141.0, 137.5, 128.6, 128.5, 128.5, 128.1, 128.1, 128.0, 127.9, 127.7, 127.6, 126.3, 121.7, 118.9, 114.5, 112.7, 109.1, 83.9, 83.4, 81.5, 80.1, 77.2, 75.9, 73.4, 73.3, 71.7, 71.4, 70.4, 70.1, 68.0, 67.1, 50.4, 47.9, 44.1, 38.0, 35.8, 31.5, 29.4, 26.9, 26.3, 26.1, 25.7, 25.2, 24.5, 21.6, 13.8.

HRMS (ESI) m/z: [M + H]$^+$ Calcd for $C_{60}H_{69}O_{12}$ 981.4784; Found 981.4788.

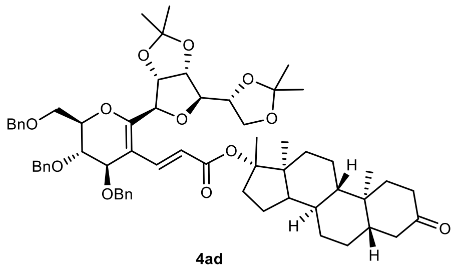

4ad

(5S,8R,9S,10S,13S,17S)-10,13,17-trimethyl-3-oxohexadecahydro-1H-cyclopenta[a]phenanthren-17-yl (E)-3-((2S,3R,4S)-3,4-bis(benzyloxy)-2-((benzyloxy)methyl)-6-((3aR,4R,6R,6aR)-6-((R)-2,2-dimethyl-1,3-dioxolan-4-yl)-2,2-dimethyltetrahydrofuro[3,4-d][1,3]dioxol-4-yl)-3,4-dihydro-2H-pyran-5-yl)acrylate

White solid, 72 mg, 71%. Purified by chromatography on silicagel, eluting with petroleum ether/ethyl acetate 5:1-3:1 (v/v). $[\alpha]_D^{25}$=92.0 (c=10 mg/ml, CH_2Cl_2). melting point: 69-71°C.

^{1}H NMR (600 MHz, Chloroform-*d*) δ 7.69 (d, *J* = 15.6 Hz, 1H), 7.36 – 7.29 (m, 13H), 7.22 – 7.21 (m, 2H), 5.79 (d, *J* = 15.5 Hz, 1H), 5.13 (s, 1H) **(anomeric H)**, 4.91 (d, *J* = 6.0 Hz, 1H), 4.82 – 4.80 (m, 1H), 4.69 – 4.65 (m, 3H), 4.47 (d, *J* = 4.3 Hz, 2H), 4.43 (d, *J* = 5.9 Hz, 2H), 4.35 – 4.32 (m, 1H), 4.27 (d, *J* = 2.9 Hz, 1H), 4.08 (dd, *J* = 8.1, 4.9 Hz, 2H), 4.01 (dd, *J* = 8.6, 4.7 Hz, 1H), 3.98 (t, *J* = 3.8 Hz, 1H), 3.77 (dd, *J* = 10.5, 7.0 Hz, 1H), 3.58 (dd, *J* = 10.5, 4.0 Hz, 1H), 2.69 (dd, *J* = 14.6, 6.0 Hz, 1H), 2.26 – 2.19 (m, 2H), 2.14 – 2.12 (m, 1H), 2.09 – 2.06 (m, 2H), 2.01 – 1.97 (m, 1H), 1.83 – 1.79 (m, 1H), 1.79 – 1.74 (m, 2H), 1.71 – 1.69 (m, 1H), 1.66 – 1.63 (m, 1H), 1.54 – 1.50 (m, 5H), 1.48 – 1.44 (m, 3H), 1.41 – 1.40 (m, 3H), 1.38 – 1.36 (m, 6H), 1.33 – 1.31 (m, 4H), 1.12 (s, 3H), 0.87 (d, *J* = 7.2 Hz, 4H), 0.84 (s, 3H).

^{13}C NMR (151 MHz, Chloroform-*d*) δ 212.1, 167.2, 158.1, 138.8, 137.6, 137.5, 137.0, 128.5, 128.4, 128.4, 128.2, 128.0, 127.9, 127.8, 127.7, 127.5, 115.8, 112.7, 109.0, 108.5, 84.1, 83.4, 82.3, 81.5, 80.2, 75.7, 73.4, 73.2, 71.8, 71.7, 70.2, 70.1, 67.9, 67.0, 50.6, 48.4, 45.8, 44.9, 42.9, 39.8, 39.4, 37.7, 36.7, 35.2, 31.0, 28.6, 27.6, 26.8, 26.2, 25.2, 24.6, 23.5, 20.0, 14.6, 14.4, 12.2.

HRMS (ESI) m/z: $[M + K]^+$ Calcd for $C_{62}H_{78}KO_{12}$ 1053.5125; Found 1053.5103.

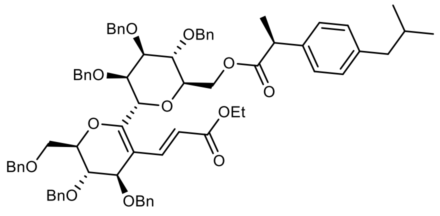

4ae

ethyl (E)-3-((2R,3S,4R)-3,4-bis(benzyloxy)-2-((benzyloxy)methyl)-6-((2S,3S,4S,5R,6R)-3,4,5-tris(benzyloxy)-6-((((S)-2-(4-isobutylphenyl)propanoyl)oxy)methyl)tetrahydro-2H-pyran-2-yl)-3,4-dihydro-2H-pyran-5-yl)acrylate

Colorless liquid, 38.6 mg, 34%. Purified by chromatography on silicagel, eluting with petroleum ether/ethyl acetate 8:1-4:1 (v/v). $[\alpha]_D^{25}$=26.0 (c=5 mg/ml, CH_2Cl_2).

^{1}H NMR (400 MHz, Chloroform-*d*) δ 7.86 (d, *J* = 15.7 Hz, 1H), 7.38 – 7.36 (m, 2H), 7.25 – 7.20 (m, 28H), 7.07 – 7.04 (m, 2H), 6.99 – 6.96 (m, 2H), 5.72 (d, *J* = 15.7 Hz, 1H), 5.12 (d, *J* = 3.6 Hz, 1H) **(anomeric H)**, 4.68 – 4.65 (m, 2H), 4.54 – 4.52 (m, 3H), 4.51 (s, 1H), 4.39 – 4.37 (m, 2H), 4.36 – 4.34 (m, 3H), 4.32 – 4.30 (m, 1H). 4.28 (d, *J* = 3.7 Hz, 1H), 4.22 – 4.21 (m, 1H), 4.20 – 4.16 (m, 2H), 4.12 – 4.10 (m, 1H), 4.02 – 3.98 (m, 1H), 3.95 – 3.92 (m, 2H), 3.90 – 3.89 (m, 1H), 3.79 – 3.75 (m, 3H), 3.61 – 3.58 (m, 2H), 2.33 – 2.31 (m, 2H), 1.73 – 1.70 (m, 1H), 1.45 (d, *J* = 7.1 Hz, 3H), 1.26 (t, *J* = 7.1 Hz, 3H), 0.80 (d, *J* = 6.6 Hz, 6H).

^{13}C NMR (101 MHz, Chloroform-*d*) δ 174.5, 167.0, 156.4, 140.4, 139.4, 138.4, 138.2, 137.7, 137.7, 137.5, 137.2, 129.3, 128.5, 128.4, 128.4, 128.3, 128.2, 128.2, 128.2, 128.1, 128.0, 127.9, 127.9, 127.8, 127.8, 127.7, 127.7, 127.7, 127.5, 127.3, 115.2, 109.2, 78.8, 75.8, 74.5, 74.4, 74.0, 73.5, 73.2, 72.5, 72.0, 71.5, 71.3, 70.2, 69.7, 67.8, 63.3, 60.0, 44.9, 30.0, 29.7, 22.4, 22.3, 18.0, 14.4.

HRMS (ESI) *m/z*: $[M + Na]^+$ Calcd for $C_{72}H_{78}NaO_{12}$ 1157.5385 found 1157.5389.

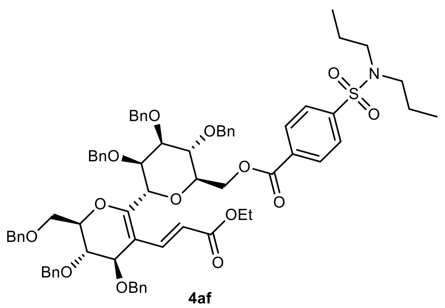

4af

((2R,3R,4S,5S,6S)-3,4,5-tris(benzyloxy)-6-((2R,3S,4R)-3,4-bis(benzyloxy)-2-((benzyloxy)methyl)-5-((E)-3-ethoxy-3-oxoprop-1-en-1-yl)-3,4-dihydro-2H-pyran-6-yl)tetrahydro-2H-pyran-2-yl)methyl 4-(N,N-dipropylsulfamoyl)benzoate

Colorless liquid, 38.8 mg, 32%. Purified by chromatography on silicagel, eluting with petroleum ether/ethyl acetate 8:1-4:1 (v/v). $[\alpha]_D^{25}$=58.0 (c=5 mg/ml, CH_2Cl_2).

^{1}H NMR (600 MHz, Chloroform-*d*) δ 8.02 (d, *J* = 8.5 Hz, 2H), 7.87 (d, *J* = 15.7 Hz, 1H), 7.73 (d, *J* = 8.5 Hz, 2H), 7.36 (dd, *J* = 6.5, 2.9 Hz, 2H), 7.30 – 7.24 (m, 19H), 7.23 – 7.21 (m, 7H), 7.18 (dd, *J* = 6.6, 2.9 Hz, 2H), 5.70 (d, *J* = 15.7 Hz, 1H), 5.14 (d, *J* = 3.8 Hz, 1H) **(anomeric H)**, 4.80 (d, *J* = 11.3 Hz, 1H), 4.68 – 4.63 (m, 3H), 4.59 – 4.50 (m, 5H), 4.45 (dd, *J* = 11.8, 2.3 Hz, 1H), 4.43 – 4.38 (m, 5H), 4.22 (s, 1H), 4.18 (t, *J* = 3.3 Hz, 1H), 4.11 (qt, *J* = 7.1, 3.9 Hz, 2H), 4.03 (dd, *J* = 7.6, 2.9 Hz, 1H), 3.98 – 3.93 (m, 2H), 3.93 – 3.89 (m, 1H), 3.67 – 3.60 (m, 2H), 3.10 – 3.05 (m, 4H), 1.53 (dt, *J* = 14.9, 7.5 Hz, 4H), 1.22 (t, *J* = 7.1 Hz, 3H), 0.87 (t, *J* = 7.4 Hz, 6H).

^{13}C NMR (151 MHz, Chloroform-*d*) δ 167.0, 164.9, 156.3, 143.9, 139.6, 138.2, 138.1, 138.0, 137.7, 137.4, 137.1, 133.4, 130.3, 128.5, 128.5, 128.4, 128.4, 128.3, 128.3, 128.1, 128.0, 128.0, 127.9, 127.8, 127.8, 127.7, 127.7, 127.7, 127.6, 126.8, 115.1, 109.3, 75.9, 74.4, 74.2, 74.1, 73.5, 73.3, 72.5, 72.1, 71.6, 71.2, 70.3, 69.7, 67.9, 64.1, 60.0, 50.0, 22.0, 14.3, 11.2.

HRMS (ESI) *m/z*: $[M + Na]^+$ Calcd for $C_{72}H_{79}NNaO_{14}S$ 1236.5113 found 1236.5111.

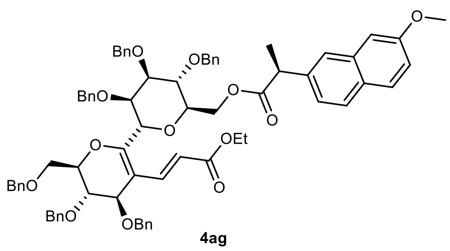

4ag

ethyl (E)-3-((2R,3S,4R)-3,4-bis(benzyloxy)-2-((benzyloxy)methyl)-6-((2S,3S,4S,5R,6R)-3,4,5-tris(benzyloxy)-6-((((S)-2-(7-methoxynaphthalen-2-yl)propanoyl)oxy)methyl)tetrahydro-2H-pyran-2-yl)-3,4-dihydro-2H-pyran-5-yl)acrylate

Colorless liquid, 34.7 mg, 30%. Purified by chromatography on silicagel, eluting with petroleum ether/ethyl acetate 8:1-4:1 (v/v). $[\alpha]_D^{25}$=38.0 (c=5 mg/ml, CH_2Cl_2).

^{1}H NMR (600 MHz, Chloroform-*d*) δ 7.84 (d, *J* = 15.7 Hz, 1H), 7.66 (s, 1H), 7.58 (t, *J* = 8.6 Hz, 2H), 7.41 – 7.39 (m, 1H), 7.35 (d, *J* = 6.5 Hz, 2H), 7.27 – 7.24 (m, 13H), 7.22 – 7.20 (m, 6H), 7.17 – 7.15 (m, 7H), 7.06 – 7.05 (m, 1H), 6.99 (d, *J* = 2.3 Hz, 1H), 6.87 (d, *J* = 6.7 Hz, 2H), 5.74 (d, *J* = 15.7 Hz, 1H), 5.11 (d, *J* = 3.9 Hz, 1H) **(anomeric H)**, 4.64 (d, *J* = 16.7 Hz, 2H), 4.54 – 4.53 (m, 2H), 4.48 – 4.43 (m, 2H), 4.37 – 4.34 (m, 4H), 4.29 – 4.26 (m, 2H), 4.25 – 4.22 (m, 2H), 4.20 (s, 2H), 4.19 (dd, *J* = 7.1, 1.9 Hz, 1H), 4.10 – 4.08 (m, 1H), 3.94 – 3.93 (m, 1H), 3.91 (d, *J* = 7.1 Hz, 1H), 3.86 – 3.85 (m, 4H), 3.82 (d, *J* = 11.2 Hz, 1H), 3.76 – 3.72 (m, 2H), 3.60 – 3.57 (m, 2H), 1.53 (d, *J* = 7.1 Hz, 3H), 1.26 (t, *J* = 7.1 Hz, 3H).

^{13}C NMR (151 MHz, Chloroform-*d*) δ 174.4, 167.1, 157.5, 156.5, 139.4, 138.3, 138.2, 138.1, 137.7, 137.5, 137.2, 135.7, 133.6, 129.3, 128.9, 128.5, 128.5, 128.4, 128.4, 128.4, 128.4, 128.4, 128.3, 128.3, 128.3, 128.2, 128.2, 128.2, 128.0, 128.0, 128.0, 128.0, 127.9, 127.8, 127.8, 127.7, 127.7, 127.7, 127.7, 127.7, 127.7, 127.6, 127.6, 127.5, 127.5, 127.4, 127.3, 127.2, 126.4, 126.0, 118.8, 115.2, 109.3, 105.5, 75.8, 74.5, 74.2, 73.7, 73.4, 73.2, 72.4, 72.0, 71.6, 70.0, 69.7, 67.8, 63.4, 60.0, 55.2, 55.2, 45.2, 18.0, 14.4.

HRMS (ESI) *m/z*: [M + Na]$^+$ Calcd for $C_{73}H_{74}NaO_{13}$ 1181.5022 found 1181.5038.

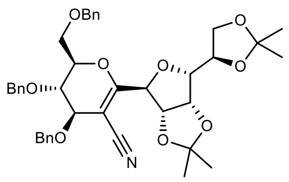

5a

(2R,3S,4R)-3,4-bis(benzyloxy)-2-((benzyloxy)methyl)-6-((3aS,4S,6R,6aS)-6-((R)-2,2-dimethyl-1,3-dioxolan-4-yl)-2,2-dimethyltetrahydrofuro[3,4-d][1,3]dioxol-4-yl)-3,4-dihydro-2H-pyran-5-carbonitrile

Colorless liquid, 20.5 mg, 30%. Purified by chromatography on silicagel, eluting with petroleum ether/ethyl acetate 8:1-4:1 (v/v). $[\alpha]_D^{25}$=38.0 (c=5 mg/ml, CH_2Cl_2).

^{1}H NMR (600 MHz, Chloroform-*d*) δ 7.36 – 7.29 (m, 13H), 7.23 (d, *J* = 7.2 Hz, 2H), 4.91 (s, 1H), 4.82 (dd, *J* = 17.6, 8.5 Hz, 2H), 4.77 – 4.75 (m, 1H), 4.65 (dd, *J* = 19.2, 11.4 Hz, 2H), 4.58 (d, *J* = 11.6 Hz, 1H), 4.49 – 4.45 (m, 2H), 4.35 (dd, *J* = 12.3, 5.8 Hz, 2H), 4.16 (d, *J* = 4.7 Hz, 1H),

4.11 – 4.07 (m, 3H), 3.81 (t, *J* = 5.5 Hz, 1H), 3.75 (dd, *J* = 10.7, 6.0 Hz, 1H), 3.62 (dd, *J* = 10.8, 3.1 Hz, 1H), 1.52 (s, 3H), 1.41 (s, 3H), 1.37 (s, 3H), 1.33 (s, 3H).

^{13}C NMR (151 MHz, Chloroform-*d*) δ 166.2, 137.5, 137.2, 137.0, 128.6, 128.5, 128.5, 128.2, 128.1, 127.9, 127.8, 127.6, 116.9, 113.3, 109.1, 87.2, 84.2, 83.6, 82.8, 81.2, 77.8, 73.4, 73.4, 73.3, 73.2, 73.1, 71.9, 67.3, 66.9, 26.9, 26.3, 25.2, 24.8.

HRMS (ESI) *m/z*: [M + Na]$^+$ Calcd for $C_{40}H_{45}NNaO_9$ 706.2987 found 706.3018.

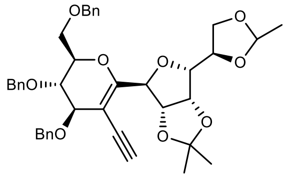

7a

(3aS,4S,6R,6aS)-4-((2R,3S,4R)-3,4-bis(benzyloxy)-2-((benzyloxy)methyl)-5-ethynyl-3,4-dihydro-2H-pyran-6-yl)-2,2-dimethyl-6-((4R)-2-methyl-1,3-dioxolan-4-yl)tetrahydro-furo[3,4-d][1,3]dioxole

Colorless liquid, 30.7 mg, 45%. Purified by chromatography on silicagel, eluting with petroleum ether/ethyl acetate 8:1-4:1 (v/v). $[\alpha]_D^{25}$=54.0 (c=10 mg/ml, CH_2Cl_2).

^{1}H NMR (400 MHz, Chloroform-*d*) δ 7.33 – 7.23 (m, 15H), 5.16 (s, 1H) **(anomeric H)**, 4.89 – 4.83 (m, 2H), 4.78 (dd, *J* = 5.9, 4.2 Hz, 1H), 4.74 – 4.66 (m, 2H), 4.65 – 4.58 (m, 2H), 4.50 – 4.47 (m, 2H), 4.36 – 4.31 (m, 1H), 4.18 (d, *J* = 5.7 Hz, 1H), 4.15 (dd, *J* = 7.9, 4.0 Hz, 1H), 4.08 – 4.03 (m, 2H), 3.82 – 3.74 (m, 2H), 3.61 (dd, *J* = 10.8, 2.8 Hz, 1H), 3.12 (s, 1H), 1.52 (s, 3H), 1.40 (s, 3H), 1.36 (s, 3H), 1.32 (s, 3H).

^{13}C NMR (101 MHz, Chloroform-*d*) δ 160.4, 137.9, 137.7, 137.7, 128.4, 128.3, 128.0, 127.8, 127.7, 127.7, 127.4, 112.6, 108.9, 96.0, 84.3, 83.3, 81.8, 81.7, 81.4, 76.7, 76.2, 73.6, 73.2, 73.1, 72.9, 72.8, 67.9, 66.9, 26.9, 26.0, 25.2, 24.4.

HRMS (ESI) *m/z*: [M + Na]$^+$ Calcd for $C_{41}H_{46}NaO_9$ 705.3034 found 705.3029.

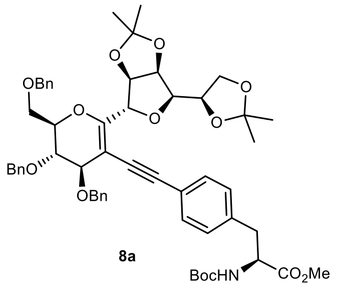

8a

methyl (S)-3-(4-(((2R,3S,4R)-3,4-bis(benzyloxy)-2-((benzyloxy)methyl)-6-((3aS,4S,6R,6aS)-6-((R)-2,2-dimethyl-1,3-dioxolan-4-yl)-2,2-dimethyltetrahydrofuro[3,4-

d][1,3]dioxol-4-yl)-3,4-dihydro-2H-pyran-5-yl)ethynyl)phenyl)-2-((tert-butoxycarbonyl)amino)propanoate

White solid, 69 mg, 72%. Purified by chromatography on silicagel, eluting with petroleum ether/ethyl acetate 5:1-3:1 (v/v). $[\alpha]_D^{25}$=43.0 (c=5 mg/ml, CH_2Cl_2). melting point: 62-64°C.

^{1}H NMR (600 MHz, Chloroform-*d*) δ 7.36 – 7.26 (m, 17H), 7.05 (d, *J* = 7.7 Hz, 2H), 5.21 (s, 1H) **(anomeric H)**, 4.98 (d, *J* = 7.8 Hz, 1H), 4.93 – 4.88 (m, 2H), 4.81 – 4.78 (m, 1H), 4.74 (dd, *J* = 14.9, 11.4 Hz, 2H), 4.63 (d, *J* = 11.5 Hz, 1H), 4.58 (q, *J* = 6.7 Hz, 1H), 4.54 – 4.50 (m, 2H), 4.36 – 4.32 (m, 1H), 4.26 (d, *J* = 5.7 Hz, 1H), 4.25 – 4.22 (m, 1H), 4.18 (dd, *J* = 7.8, 4.1 Hz, 1H), 4.10 – 4.05 (m, 2H), 3.87 – 3.85 (m, 1H), 3.80 (dd, *J* = 10.9, 5.4 Hz, 1H), 3.70 (s, 3H), 3.65 (dd, *J* = 10.8, 2.6 Hz, 1H), 3.07 (ddd, *J* = 40.1, 13.8, 5.9 Hz, 2H), 1.53 (s, 3H), 1.43 – 1.40 (m, 12H), 1.37 (s, 3H), 1.33 (s, 3H).

^{13}C NMR (151 MHz, Chloroform-*d*) δ 172.1, 159.0, 138.0, 137.8, 137.8, 135.9, 131.3, 129.1, 128.4, 128.4, 128.3, 128.0, 127.8, 127.7, 127.7, 127.4, 112.6, 109.0, 97.1, 93.3, 84.5, 83.4, 81.9, 81.4, 76.6, 73.7, 73.3, 73.0, 72.9, 68.1, 67.0, 52.2, 28.2, 26.9, 26.1, 25.2, 24.5.

HRMS (ESI) m/z: $[M + H]^+$ Calcd for $C_{56}H_{66}NO_{13}$ 960.4529; Found 960.4532.

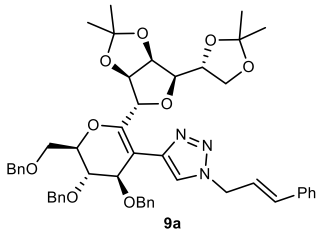

9a

4-((2R,3S,4R)-3,4-bis(benzyloxy)-2-((benzyloxy)methyl)-6-((3aS,4S,6R,6aS)-6-((R)-2,2-dimethyl-1,3-dioxolan-4-yl)-2,2-dimethyltetrahydrofuro[3,4-d][1,3]dioxol-4-yl)-3,4-dihydro-2H-pyran-5-yl)-1-cinnamyl-1H-1,2,3-triazole

Colorless liquid, 67.3 mg, 80%. Purified by chromatography on silicagel, eluting with petroleum ether/ethyl acetate 5:1-2:1 (v/v). $[\alpha]_D^{25}$=26.0 (c=10 mg/ml, CH_2Cl_2).

^{1}H NMR (600 MHz, Chloroform-*d*) δ 7.46 (s, 1H), 7.36 – 7.27 (m, 15H), 7.21 – 7.16 (m, 3H), 7.09 – 7.05 (m, 2H), 6.63 (d, *J* = 15.8 Hz, 1H), 6.31 – 6.26 (m, 1H), 5.07 (d, *J* = 6.6 Hz, 2H), 4.98 (s, 1H) **(anomeric H)**, 4.88 (d, *J* = 6.0 Hz, 1H), 4.82 – 4.79 (m, 1H), 4.73 (d, *J* = 11.7 Hz, 1H), 4.66 (d, *J* = 11.8 Hz, 1H), 4.56 (d, *J* = 4.6 Hz, 1H), 4.52 (d, *J* = 11.1 Hz, 3H), 4.45 (d, *J* = 11.1 Hz, 1H), 4.38 – 4.35 (m, 1H), 4.31 – 4.27 (m, 1H), 4.23 – 4.19 (m, 1H), 4.05 – 4.01 (m,

2H), 3.95 (t, *J* = 5.4 Hz, 1H), 3.86 (dd, *J* = 10.7, 6.1 Hz, 1H), 3.66 (dd, *J* = 10.7, 2.8 Hz, 1H), 1.41 – 1.38 (m, 6H), 1.35 (s, 3H), 1.26 (s, 3H).

^{13}C NMR (151 MHz, Chloroform-*d*) δ 152.9, 143.3, 138.0, 137.9, 137.9, 135.5, 135.3, 128.7, 128.5, 128.4, 128.2, 127.9, 127.8, 127.8, 127.7, 127.6, 127.5, 126.7, 122.5, 121.9, 112.6, 109.0, 103.2, 84.7, 83.5, 81.7, 81.2, 76.1, 76.0, 73.7, 73.3, 73.2, 72.5, 71.9, 68.2, 67.1, 52.2, 26.9, 26.1, 25.3, 24.5.

HRMS (ESI) *m/z*: [M + Na]$^+$ Calcd for $C_{50}H_{55}N_3NaO_9$ 864.3831 found 864.3809.

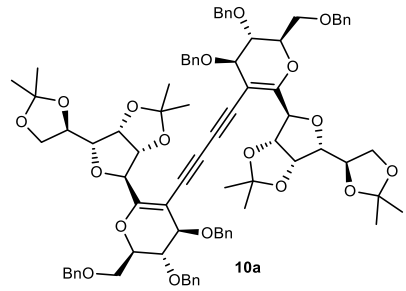

10a

1-((2R,3S,4R)-3,4-bis(benzyloxy)-2-((benzyloxy)methyl)-6-((3aS,4S,6R,6aS)-6-((R)-2,2-dimethyl-1,3-dioxolan-4-yl)-2,2-dimethyltetrahydrofuro[3,4-d][1,3]dioxol-4-yl)-3,4-dihydro-2H-pyran-5-yl)-4-((2S,3R,4S)-3,4-bis(benzyloxy)-2-((benzyloxy)methyl)-6-((3aS,4S,6R,6aS)-6-((R)-2,2-dimethyl-1,3-dioxolan-4-yl)-2,2-dimethyltetrahydrofuro[3,4-d][1,3]dioxol-4-yl)-3,4-dihydro-2H-pyran-5-yl)buta-1,3-diyne

White solid, 88.5 mg, 65%. Purified by chromatography on silicagel, eluting with petroleum ether/ethyl acetate 8:1-4:1 (v/v). $[\alpha]_D^{25}$=61.0 (c=5 mg/ml, CH_2Cl_2). melting point: 74-76°C.

^{1}H NMR (400 MHz, Chloroform-*d*) δ 7.33 – 7.23 (m, 30H), 5.14 (s, 2H) **(anomeric H)**, 4.83 (d, *J* = 9.6 Hz, 4H), 4.79 – 4.76 (m, 2H), 4.72 (d, *J* = 11.5 Hz, 2H), 4.61 (dd, *J* = 16.9, 11.3 Hz, 4H), 4.51 – 4.46 (m, 4H), 4.33 (q, *J* = 5.8 Hz, 2H), 4.26 – 4.22 (m, 2H), 4.18 – 4.12 (m, 4H), 4.10 – 4.04 (m, 4H), 3.77 (dt, *J* = 10.7, 5.9 Hz, 4H), 3.61 (d, *J* = 10.7 Hz, 2H), 1.50 (s, 6H), 1.40 (s, 6H), 1.37 (s, 6H), 1.30 (s, 6H).

^{13}C NMR (101 MHz, Chloroform-*d*) δ 162.0, 137.7, 137.7, 137.6, 128.4, 128.4, 128.3, 127.8, 127.8, 127.8, 127.5, 112.7, 109.0, 96.6, 84.2, 83.5, 81.9, 81.5, 78.7, 77.8, 75.9, 73.7, 73.3, 73.3, 73.2, 72.9, 68.0, 67.0, 26.9, 26.1, 25.2, 24.4.

HRMS (ESI) *m/z*: [M + Na]$^+$ Calcd for $C_{82}H_{90}NaO_{18}$ 1385.6019 found 1385.6018.

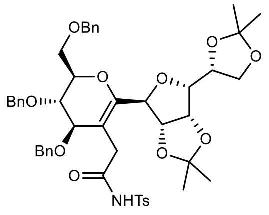

11a

2-((2R,3S,4R)-3,4-bis(benzyloxy)-2-((benzyloxy)methyl)-6-((3aS,4S,6R,6aS)-6-((R)-2,2-dimethyl-1,3-dioxolan-4-yl)-2,2-dimethyltetrahydrofuro[3,4-d][1,3]dioxol-4-yl)-3,4-dihydro-2H-pyran-5-yl)-N-tosylacetamide

Colorless liquid, 66 mg, 76%. Purified by chromatography on silicagel, eluting with petroleum ether/ethyl acetate 5:1-2:1 (v/v). $[\alpha]_D^{25}$=-8.0 (c=10 mg/ml, CH_2Cl_2).

^{1}H NMR (600 MHz, Chloroform-*d*) δ 9.54 (s, 1H), 7.79 (dd, *J* = 15.2, 8.2 Hz, 3H), 7.37 – 7.35 (m, 3H), 7.33 – 7.31 (m, 5H), 7.30 – 7.27 (m, 4H), 7.21 (d, *J* = 7.8 Hz, 4H), 4.97 (s, 1H) **(anomeric H)**, 4.80 (d, *J* = 6.0 Hz, 1H), 4.77 – 4.74 (m, 1H), 4.68 – 4.66 (m, 2H), 4.64 (d, *J* = 13.8 Hz, 1H), 4.55 – 4.49 (m, 2H), 4.46 (d, *J* = 11.3 Hz, 1H), 4.32 – 4.28 (m, 1H), 4.24 (q, *J* = 5.7 Hz, 1H), 4.08 – 4.03 (m, 3H), 3.88 – 3.86 (m, 1H), 3.83 (d, *J* = 4.6 Hz, 1H), 3.74 (dd, *J* = 10.6, 6.0 Hz, 1H), 3.58 (dd, *J* = 10.6, 3.6 Hz, 1H), 3.08 (d, *J* = 16.1 Hz, 1H), 2.75 (d, *J* = 16.1 Hz, 1H), 2.37 (s, 3H), 1.47 (s, 3H), 1.40 (s, 3H), 1.36 (s, 3H), 1.31 (s, 3H).

^{13}C NMR (151 MHz, Chloroform-*d*) δ 168.4, 152.4, 144.5, 143.4, 139.2, 137.6, 137.4, 136.6, 135.9, 129.6, 129.3, 128.7, 128.6, 128.5, 128.4, 128.4, 128.2, 128.1, 127.9, 127.8, 127.6, 126.4, 112.5, 108.9, 103.1, 83.2, 82.9, 81.4, 80.2, 76.5, 75.5, 73.5, 73.4, 73.3, 73.3, 72.7, 67.5, 66.9, 37.2, 26.9, 26.1, 25.2, 24.5, 21.6.

HRMS (ESI) *m/z*: $[M + Na]^+$ Calcd for $C_{48}H_{55}NNaO_{12}S$ 892.3337 found 892.3324.

5. Computational Details

All the structures were optimized by the B3LYP hybrid functional with the Grimme's dispersion correction at the D3 level (B3LYP-D3) including solvation effects via integral equation formalism model by using the Gaussian 09 program[14-18]. The 6-31G* basis set was used to C, H, O, N, P, and Cl atoms, while Lanl2DZ basis set was employed to Pd, I, and Cs atoms[19-20]. All of the energy minima and transition states have been verified by vibrational frequency analyses at the same level with no or only one imaginary frequency, respectively. The Gibbs free energy of solutes in the liquid phase is calculated as:

$$G_{liq}=E_{sp}+G_{ther}+\Delta G_{sol}$$

The single point energies were revised at the PBE0-D3(BJ)/ def2-TZVPD level with RIJCOSX approximation by ORCA package [21-24]. All positive frequencies which are less than 100 cm^{-1} are set to 100 cm^{-1} for thermodynamics calculations [25]. The total Gibbs free energies include electronic energies and thermal corrections to Gibbs free energies at the experimental temperature. ΔG_{sol} is the solvation free energy at M05-2X/6-31G* level [26]. The Pd-C1 bond of the Pd(IV) species (**F-α**) dissociates to form the oxocarbenium ion with absorbing 42.4 kcal/mol, which hints that the mannofuransyl Pd(IV) intermediate **F-α** is configurationally stable [27].

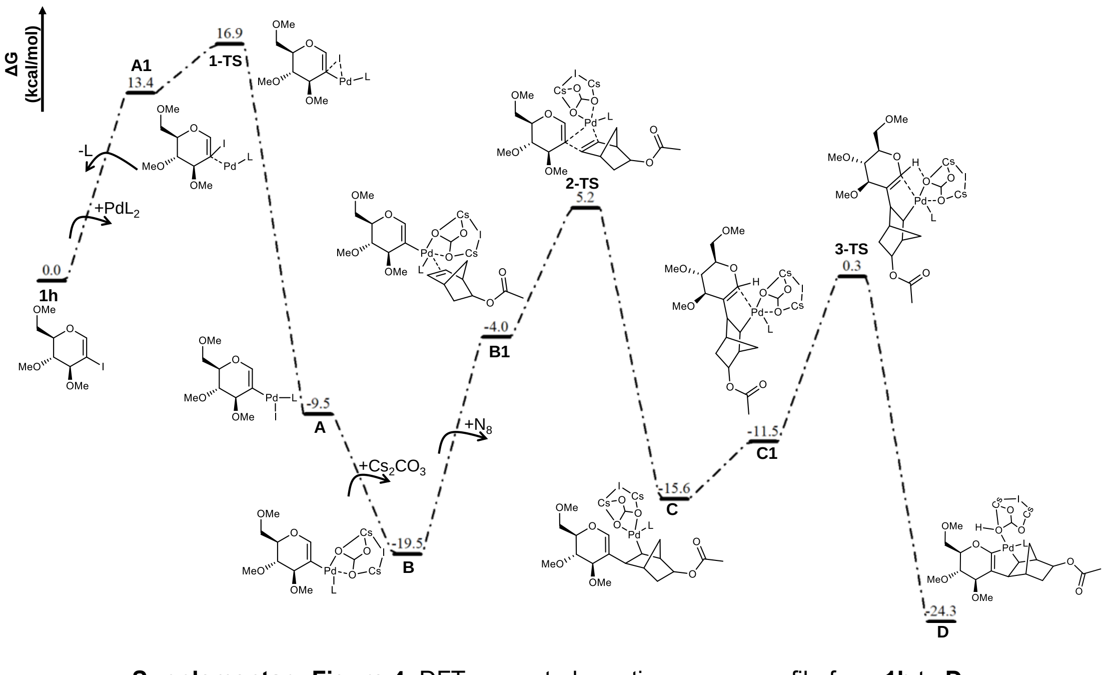

Supplementary Figure 4. DFT-computed reaction energy profile from **1h** to **D.**

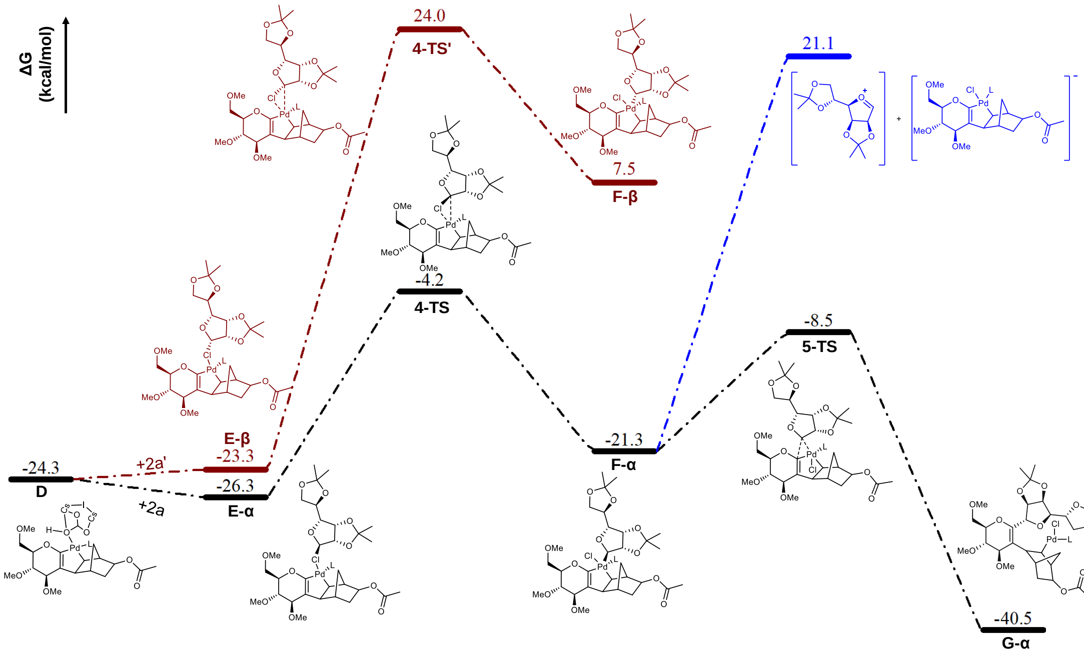

Supplementary Figure 5. DFT-computed reaction energy profile from **D** to **G-α.**

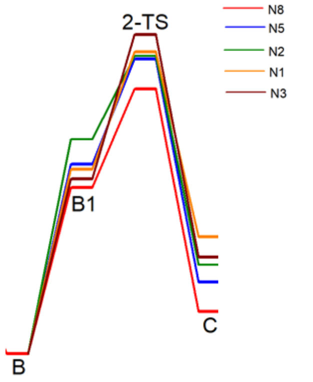

	B	B1	2-TS	C
**N8**	-19.5	-4.0	5.2	-15.6
**N5**	-19.5	-1.8	8.0	-12.8
**N2**	-19.5	0.5	8.3	-11.2
**N1**	-19.5	-2.3	8.7	-8.6
**N3**	-19.5	-3.2	10.3	-10.5

Supplementary Figure 6. DFT-computed reaction energy profile from **B** to **C.**

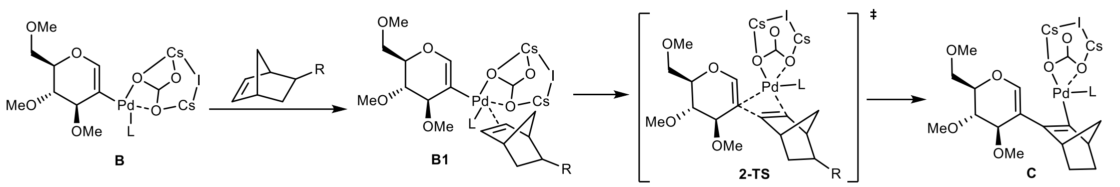

NBE	R	ΔG (B1)	$\Delta G^{\#}$ (2-TS)
N8	$OCOCH_3$	15.5	24.7
N5	$COOCH_3$	17.7	27.5
N2	CN	20.0	27.8
N1	H	17.2	28.2
N3	$COCH_3$	16.3	29.8

Supplementary Figure 7. The computed reaction profile for the migratory insertion with other C5-substituted smNBEs.

From the Figure, it could be found that the total energy barriers of **2-TS** *(*$\mathbf{N_8}$, $\mathbf{N_5}$, $\mathbf{N_2}$, $\mathbf{N_1}$ and $\mathbf{N_3}$*)* are 24.7, 27.5, 27.8, 28.2 and 29.8 kcal/mol, respectively*.* The computational results are in agreement with the experimental observations.

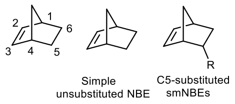

Supplementary Figure 8. Simple unsubstituted NBE and C5-substituted smNBEs

In general, C5-substituted smNBEs exhibit reactivity similar to that of simple unsubstituted NBE, and the exact reason is unclear [28-30]. The steric and electronic environment of the NBE alkene in these smNBEs is almost identical to that of simple NBE, though the remote steric effect or coordinative properties of the C5 substituents could play an important role in modulating reaction selectivity. In our reaction, we speculate that the possible reason is that the electronic effects of the C5 substituents play an important role in the reaction. The C5-substituted smNBEs (-$OCOCH_3$ for $\mathbf{N_8}$, -$COOCH_3$ for $\mathbf{N_5}$, -CN for $\mathbf{N_2}$, and -$COCH_3$ for $\mathbf{N_3}$) could have electrostatic attractions with one Cs atom which is denoted by O(N)-Cs distances and could lower the barrier. However, the smNBEs could have repulsive interactions with the neighbor Cs_2ICO_3 group which is denoted by C-Cs distances and could elevate the barrier. For $\mathbf{N_8}$, it has the shortest O-Cs distance and the longest C-Cs distances, which indicates that it has the strongest attractions and the weakest repulsions, and therefore has the lowest barrier and the optimal reactivity (**Figure 9**). On the other hand, we cannot rule out whether there are other steric effect or coordination properties affecting the activity of C5-substituted smNBEs. We really hope to provide more detailed explanation of the activity of C5 substituted smNBEs from remote steric effect or coordinative properties in our laboratory in the future.

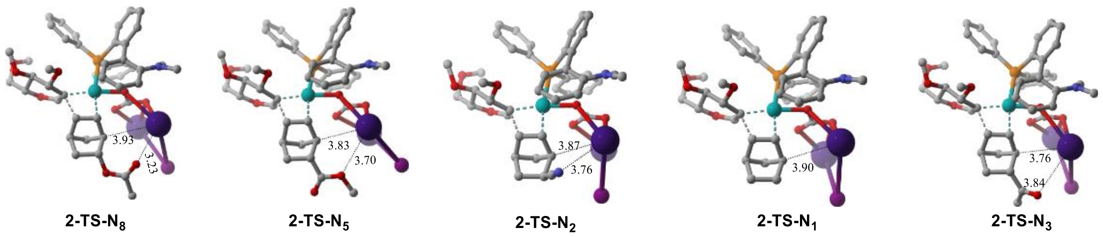

Supplementary Figure 9. Migratory-insertion transitions states with $\mathbf{N_8}$, $\mathbf{N_5}$, $\mathbf{N_2}$, $\mathbf{N_1}$, and $\mathbf{N_3}$. Bond distances are in Å.

To prove that the oxocarbenium could not form during the experiments, calculations of intrinsic reaction coordinate (IRC) of transition state **4-TS** (**Figure 10**) and scan of C-Cl bond of F-α (**Figure 11**) are also performed. The energy curve of scan of C-Cl bond has a maximum, and could be considered similar to the curve of IRC. The calculated results support the concerted three-membered cyclic transition state and our conclusions are also consistent with the previous work[27].

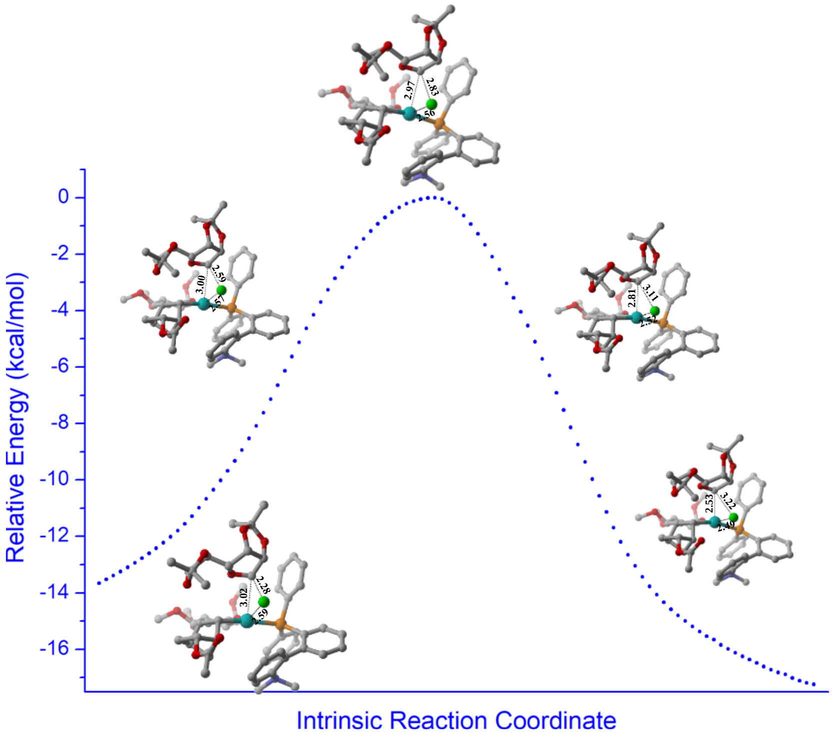

Supplementary Figure 10. Intrinsic reaction coordinate (IRC) of transition state **4-TS**. Some selective structures along IRC are also shown. The energy of transition state is set to zero.

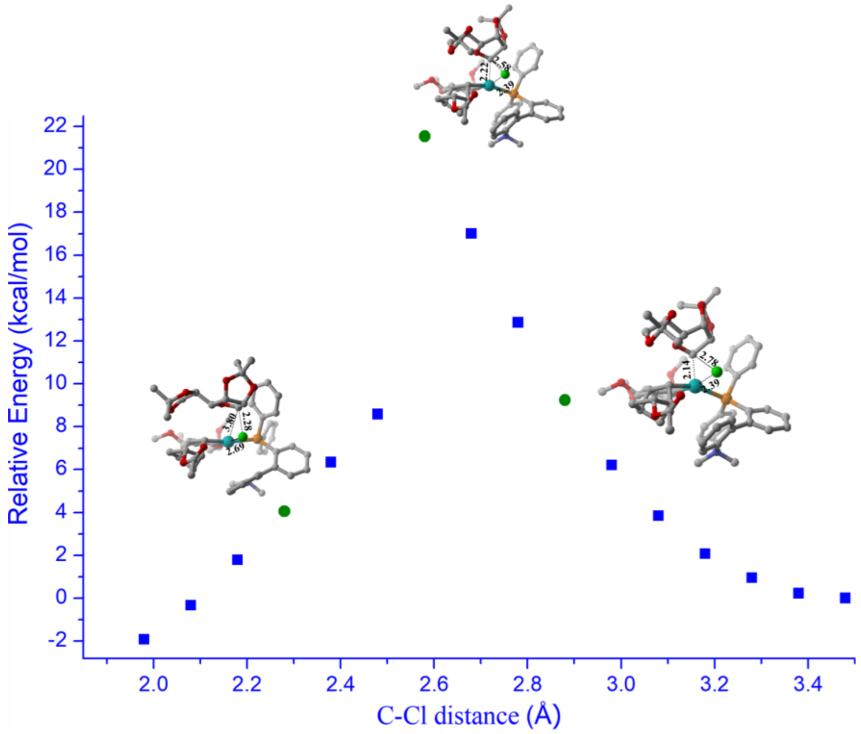

Supplementary Figure 11. Relaxed scan of C-Cl bond of the intermediate **F-α**. The energy of **F-α** is set to zero.

II. Supplementary Figures

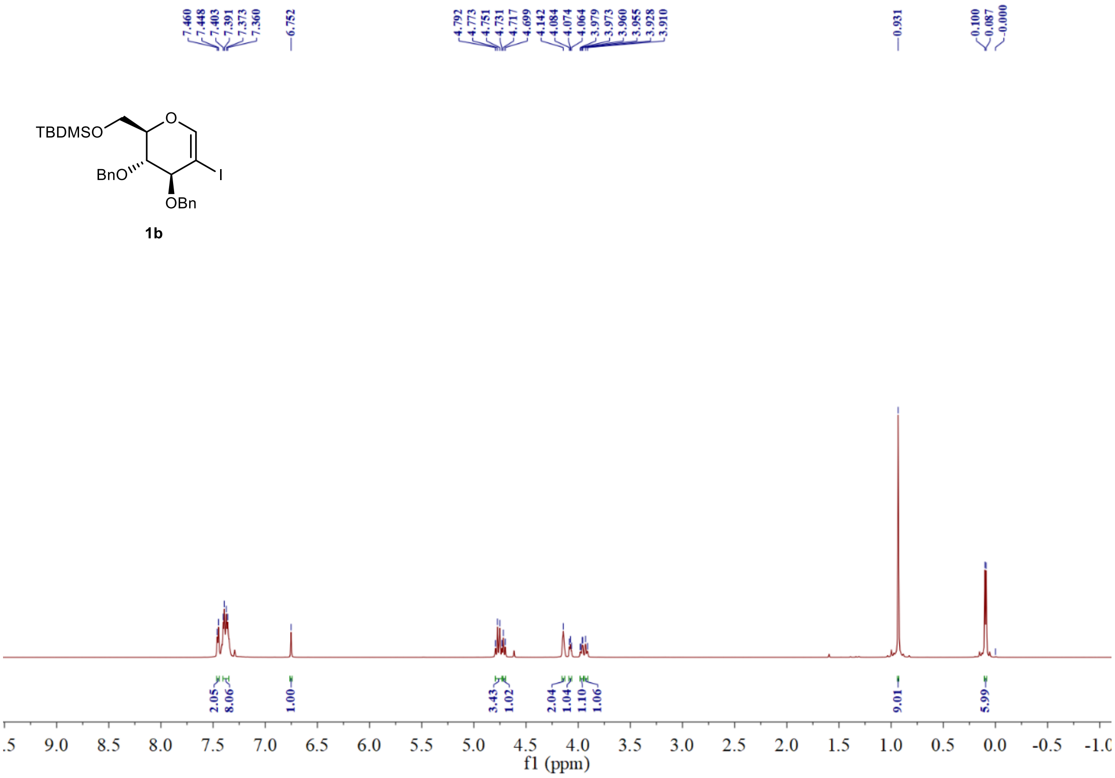

Supplementary Figure 12. ^{1}H NMR spectra of (600 MHz, $CDCl_3$) compound **1b**

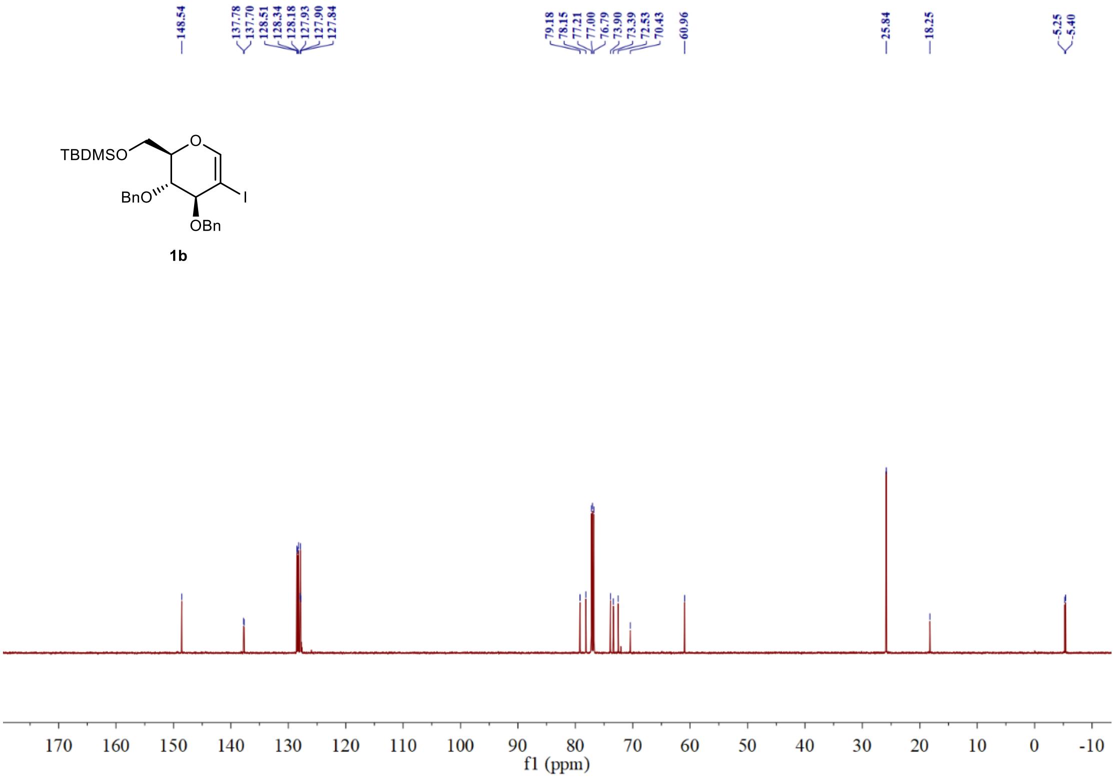

Supplementary Figure 13. ^{13}C spectra of (151 MHz, $CDCl_3$) compound **1b**

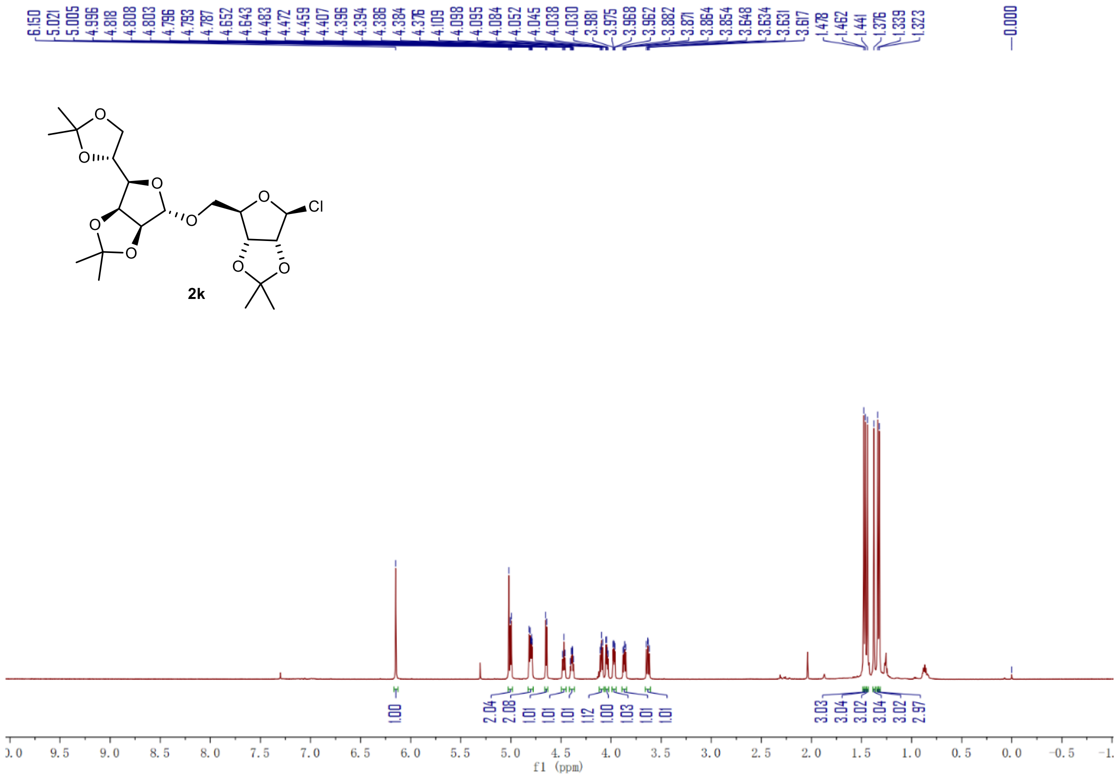

Supplementary Figure 14. ^{1}H NMR spectra of (600 MHz, $CDCl_3$) compound **2k**

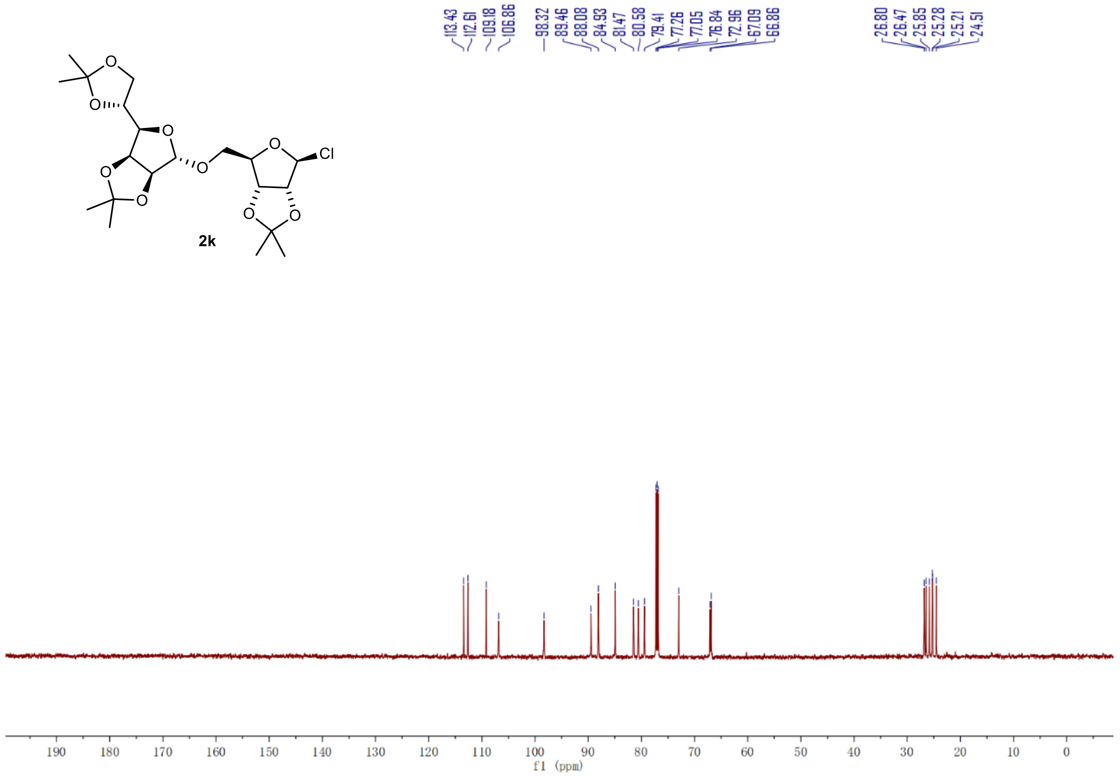

Supplementary Figure 15. ^{13}C spectra of (151 MHz, $CDCl_3$) compound **2k**

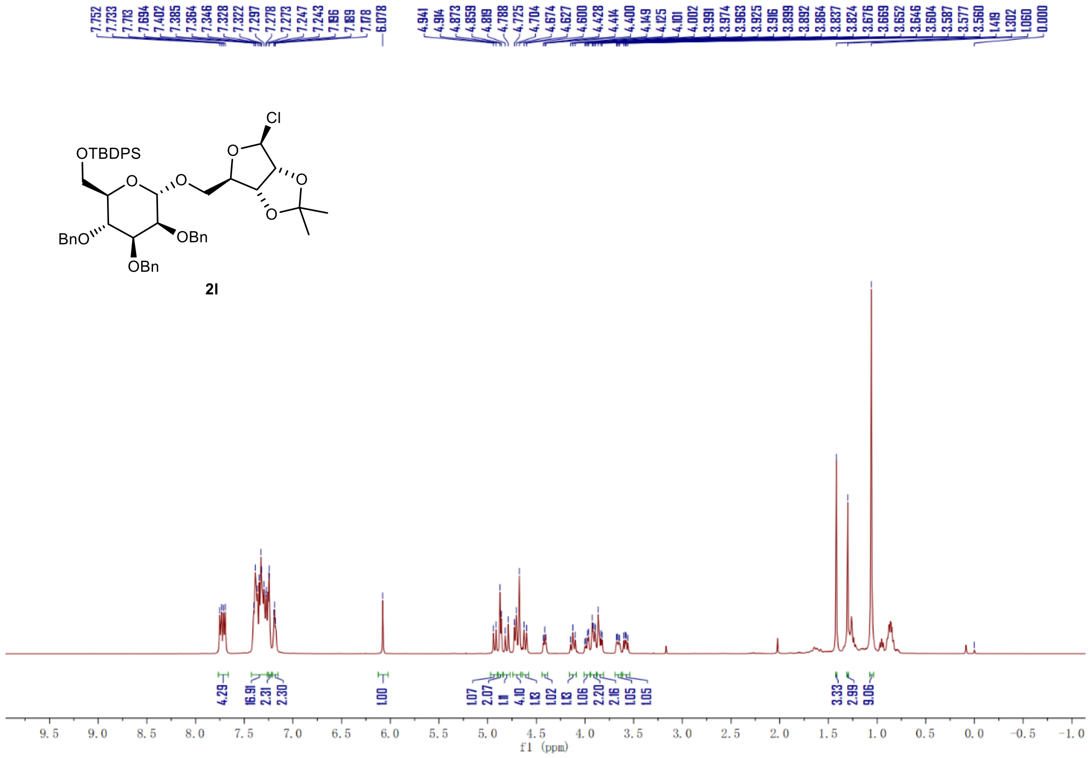

Supplementary Figure 16. ^{1}H NMR spectra of (400 MHz, $CDCl_3$) compound **2l**

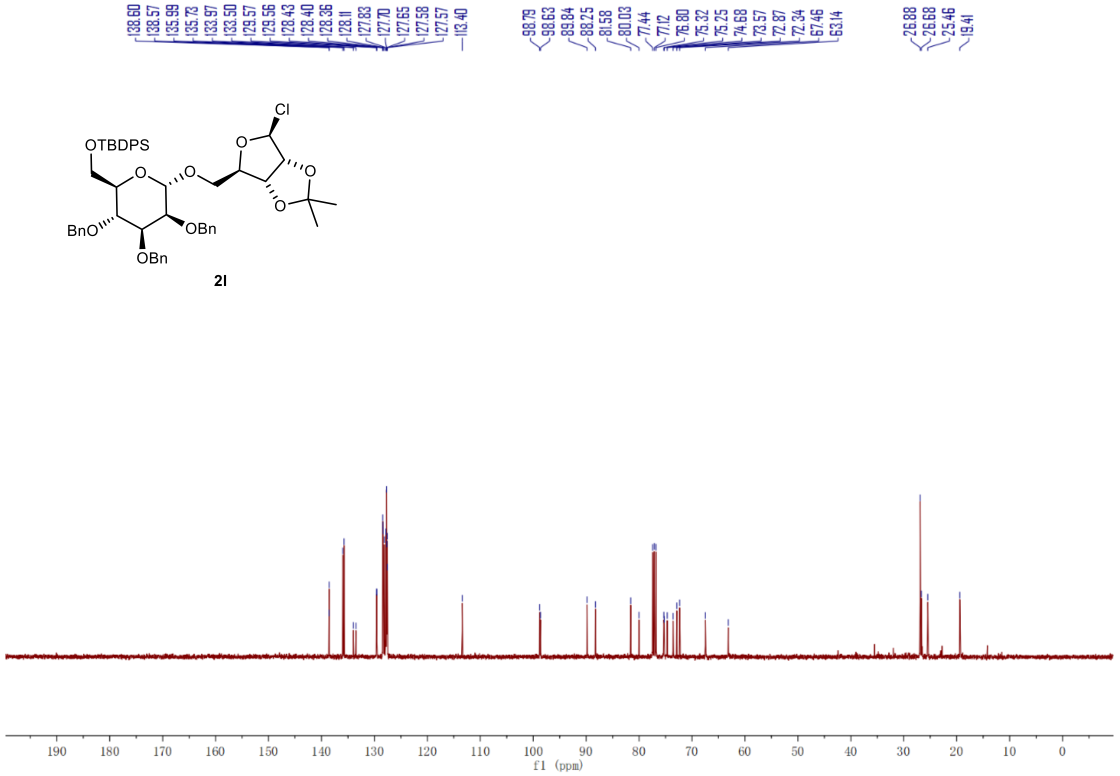

Supplementary Figure 17. ^{13}C spectra of (101 MHz, $CDCl_3$) compound **2l**

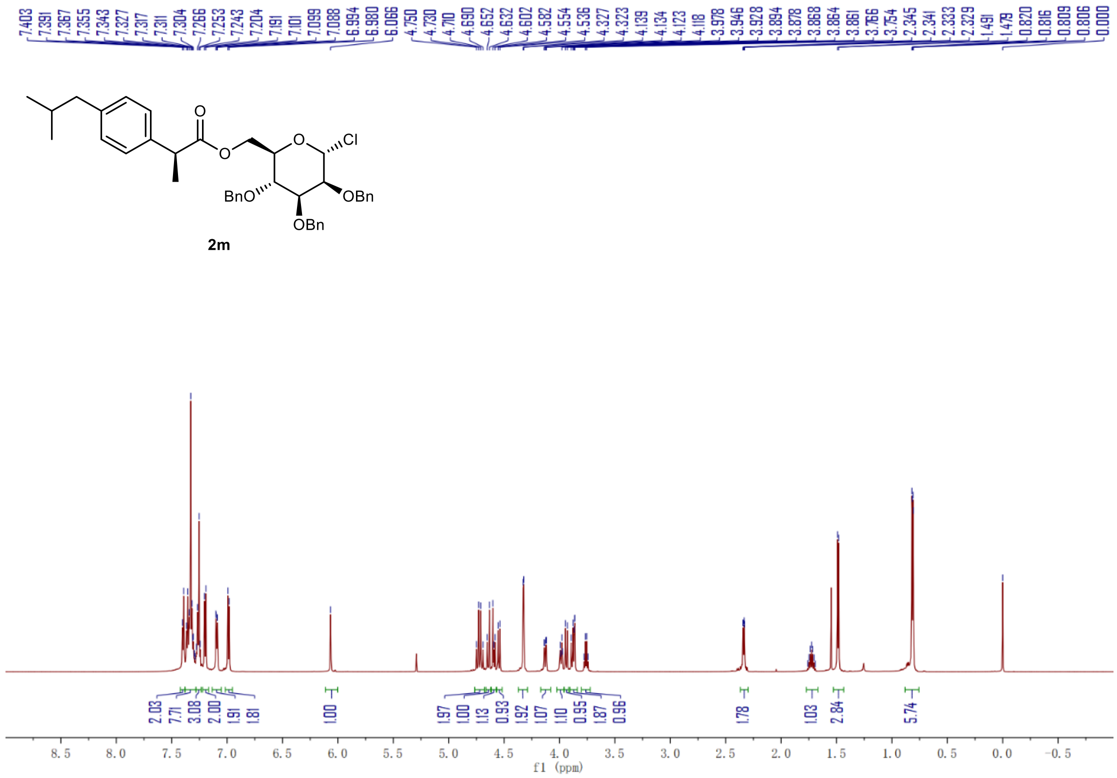

Supplementary Figure 18 ^{1}H NMR spectra of (600 MHz, $CDCl_3$) compound **2m**

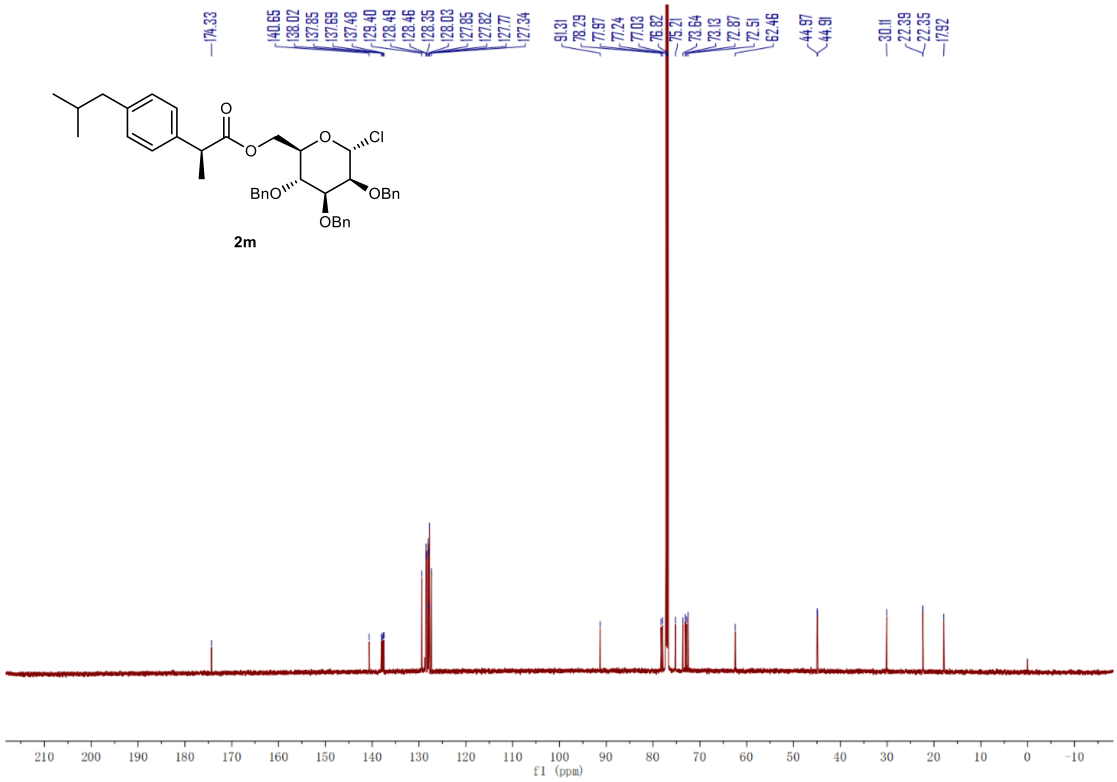

Supplementary Figure 19 ^{13}C spectra of (151 MHz, $CDCl_3$) compound **2m**

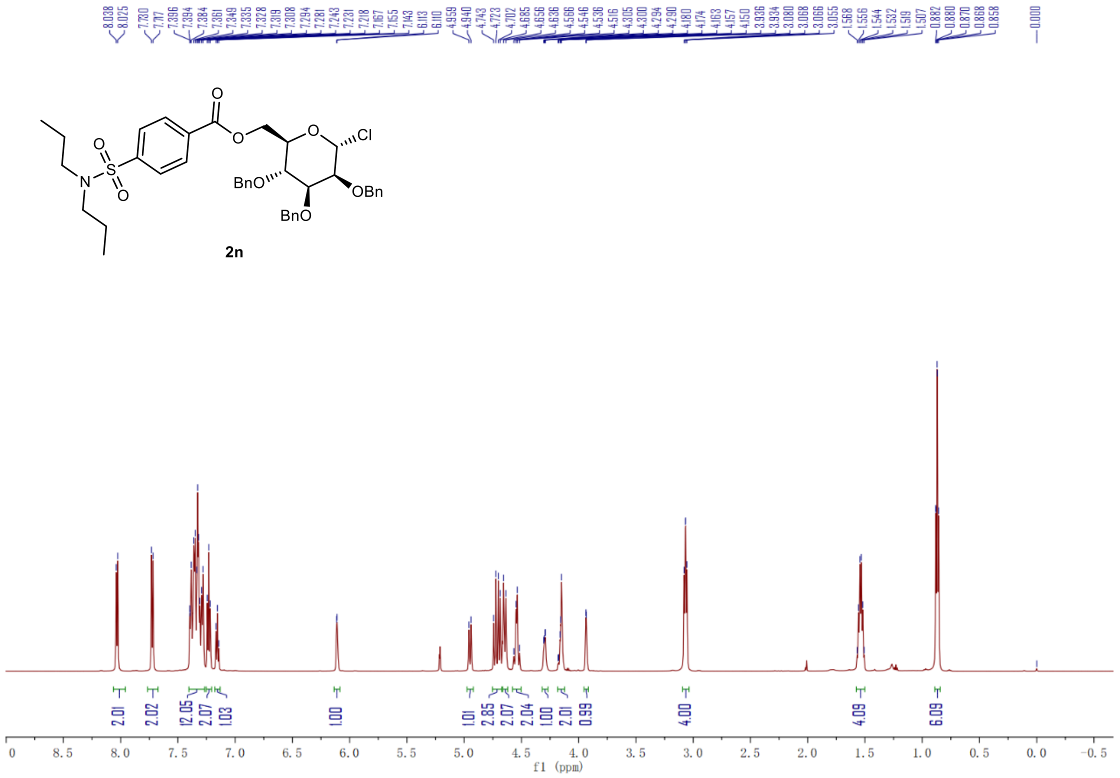

Supplementary Figure 20 ^{1}H NMR spectra of (600 MHz, $CDCl_3$) compound **2n**

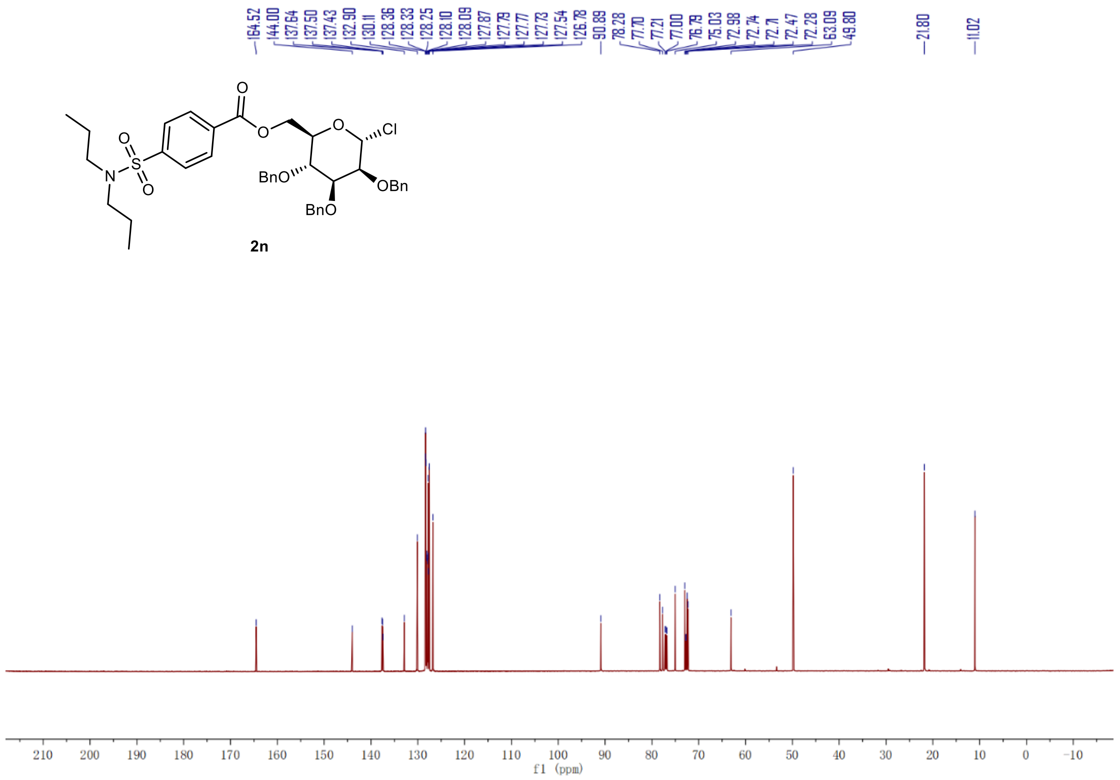

Supplementary Figure 21 ^{13}C spectra of (151 MHz, $CDCl_3$) compound **2n**

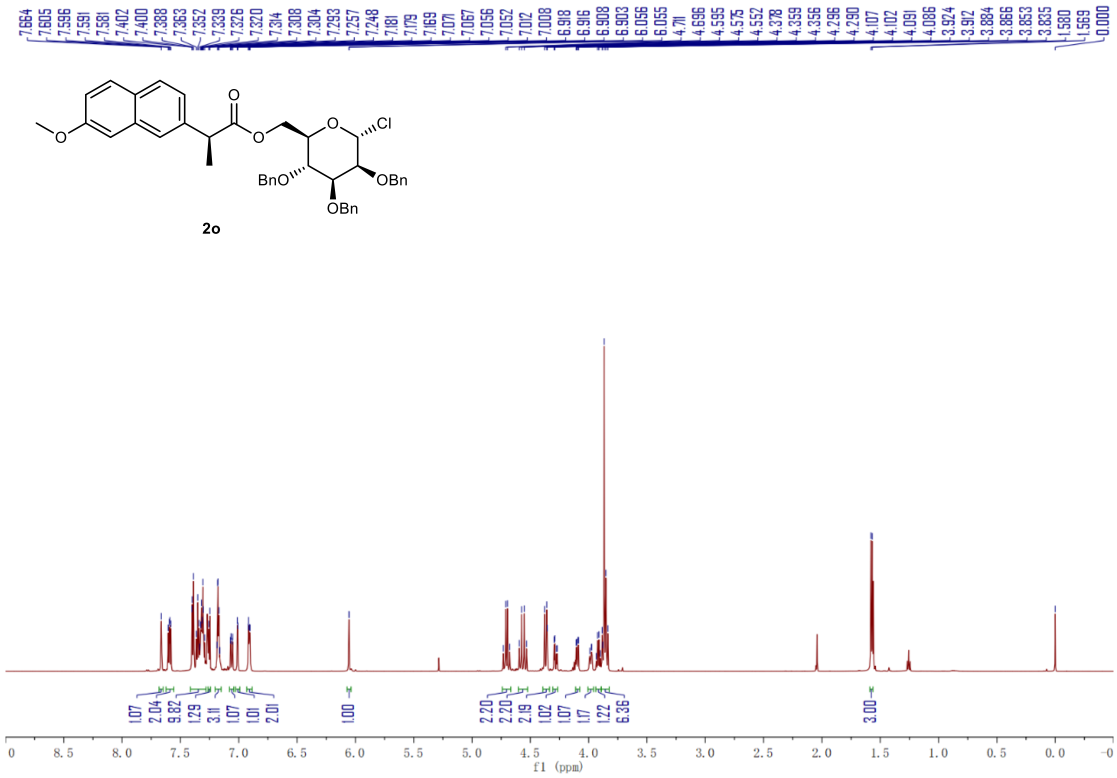

Supplementary Figure 22 ^{1}H NMR spectra of (600 MHz, $CDCl_3$) compound **2o**

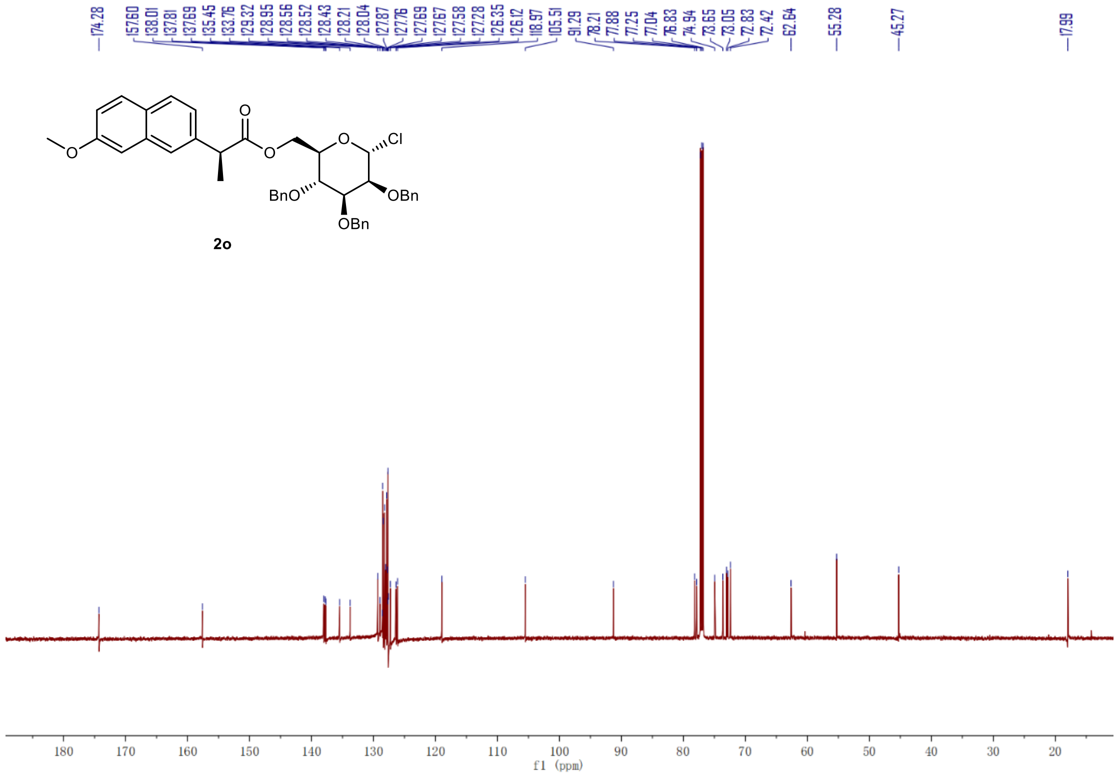

Supplementary Figure 23 ^{13}C spectra of (151 MHz, $CDCl_3$) compound **2o**

(¹H NMR, 400 MHz, CDCl₃; ¹³C {¹H} NMR, 101 MHz, CDCl₃)

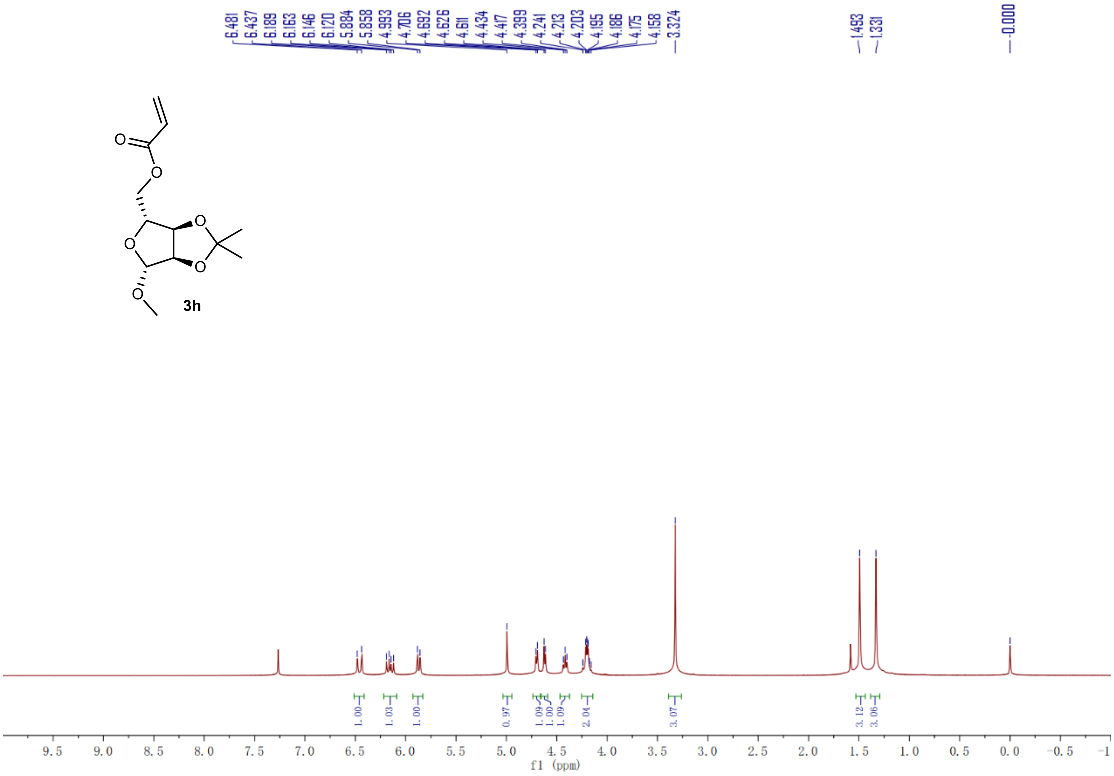

Supplementary Figure 24 ^{1}H NMR spectra of (400 MHz, $CDCl_3$) compound **3h**

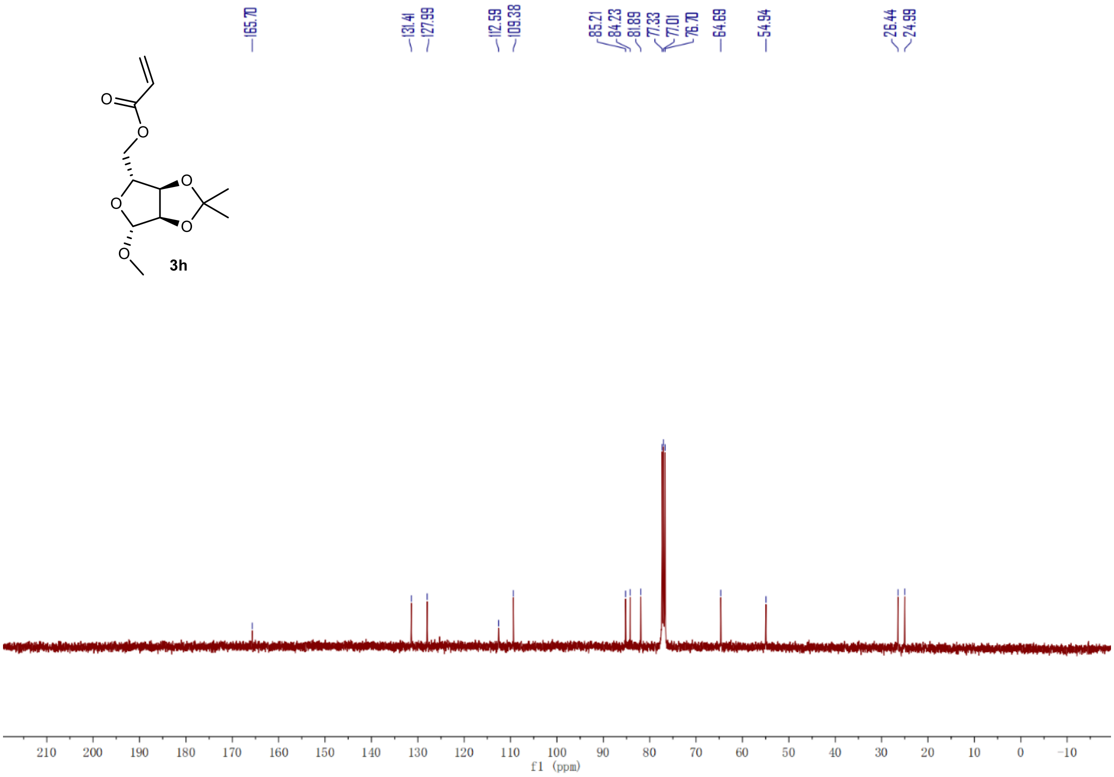

Supplementary Figure 25 ^{13}C spectra of (101 MHz, $CDCl_3$) compound **3h**

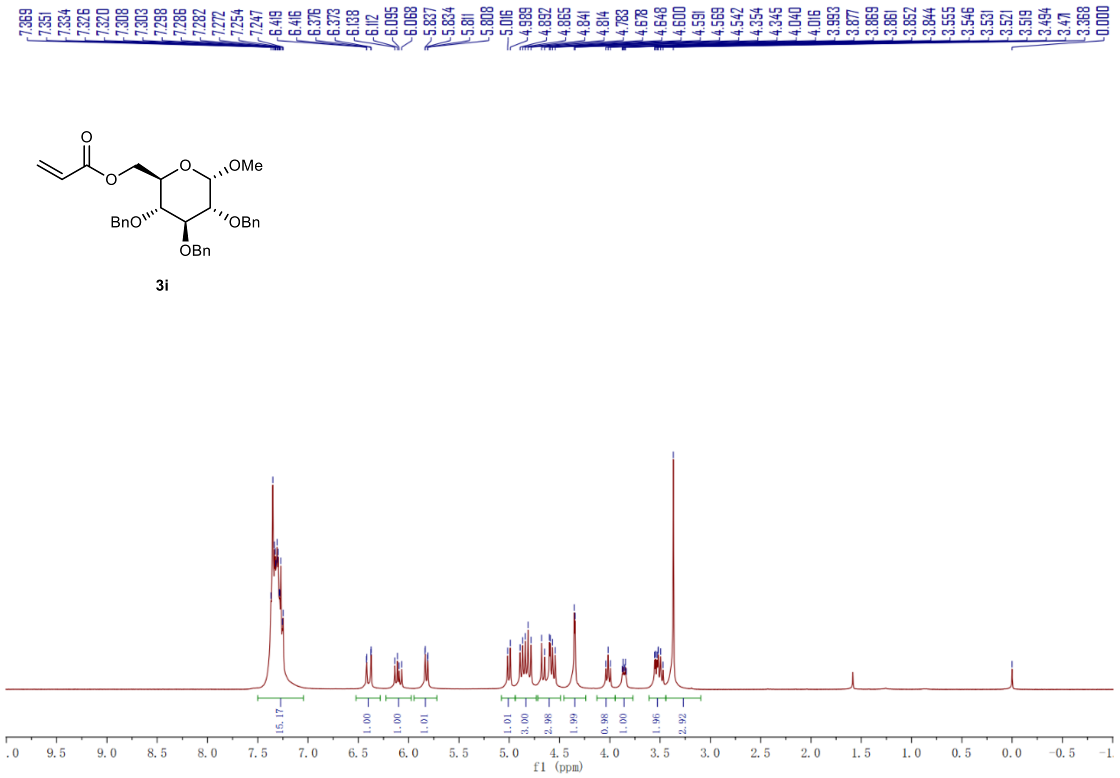

Supplementary Figure 26 ^{1}H NMR spectra of (400 MHz, $CDCl_3$) compound **3i**

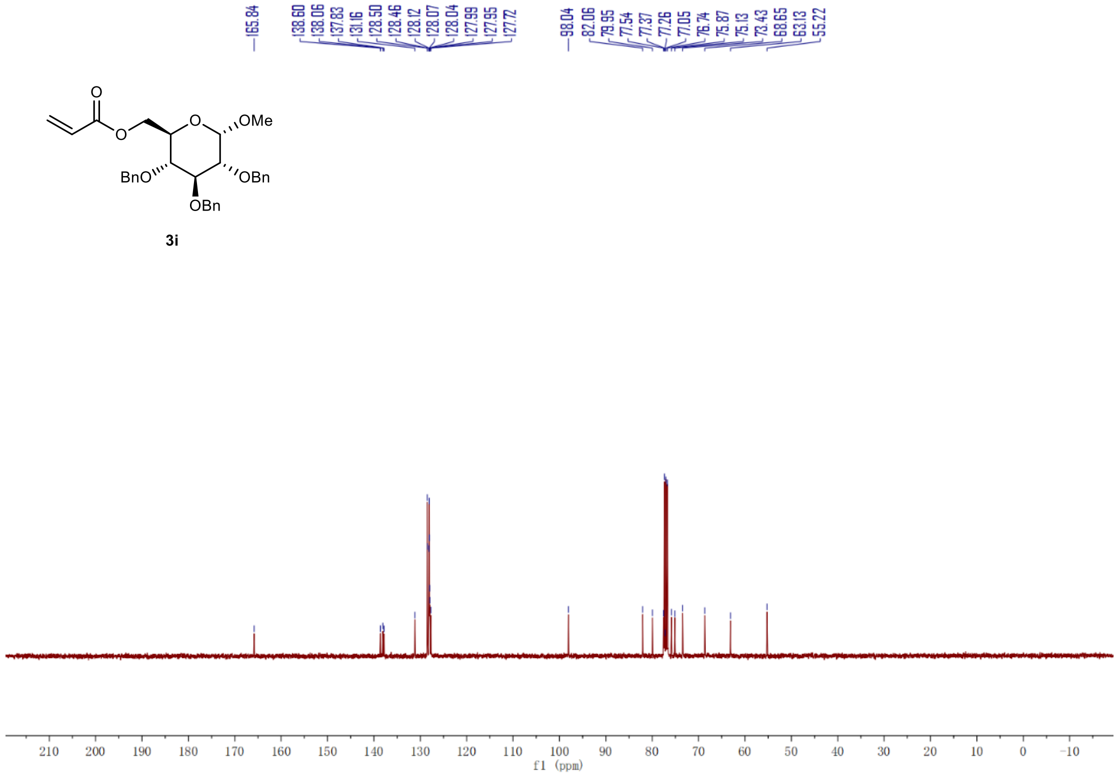

Supplementary Figure 27 ^{13}C spectra of (101 MHz, $CDCl_3$) compound **3h**

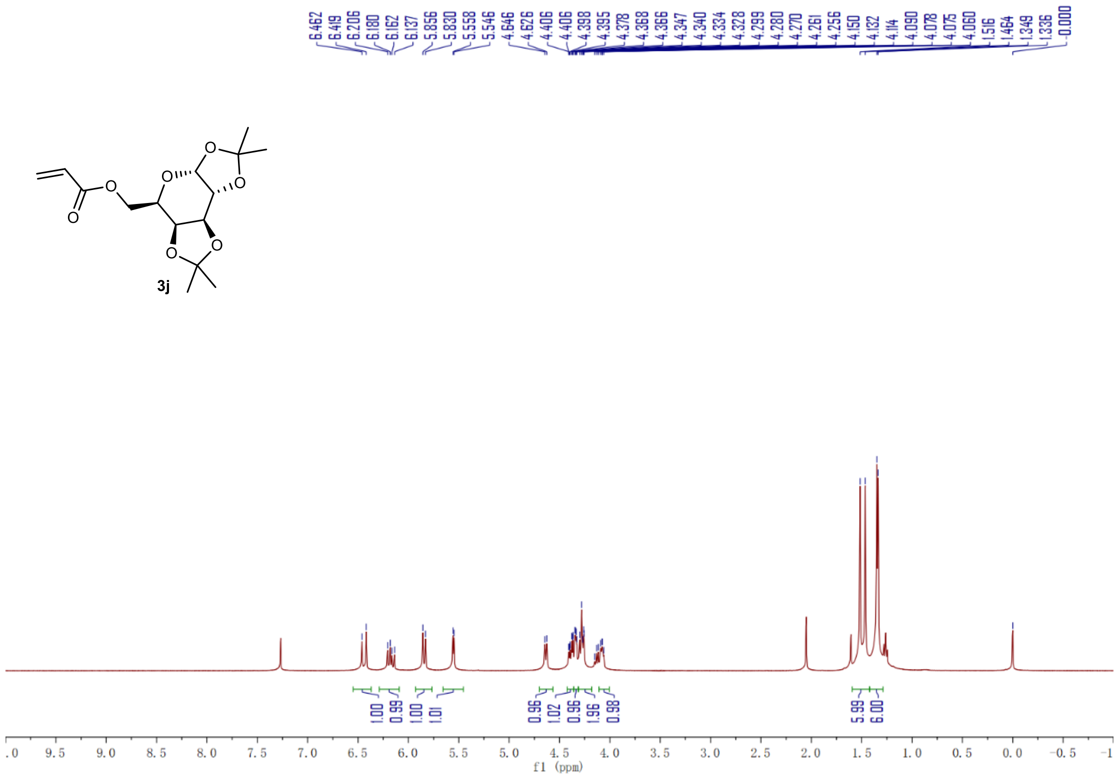

Supplementary Figure 28 ^{1}H NMR spectra of (400 MHz, $CDCl_3$) compound **3j**

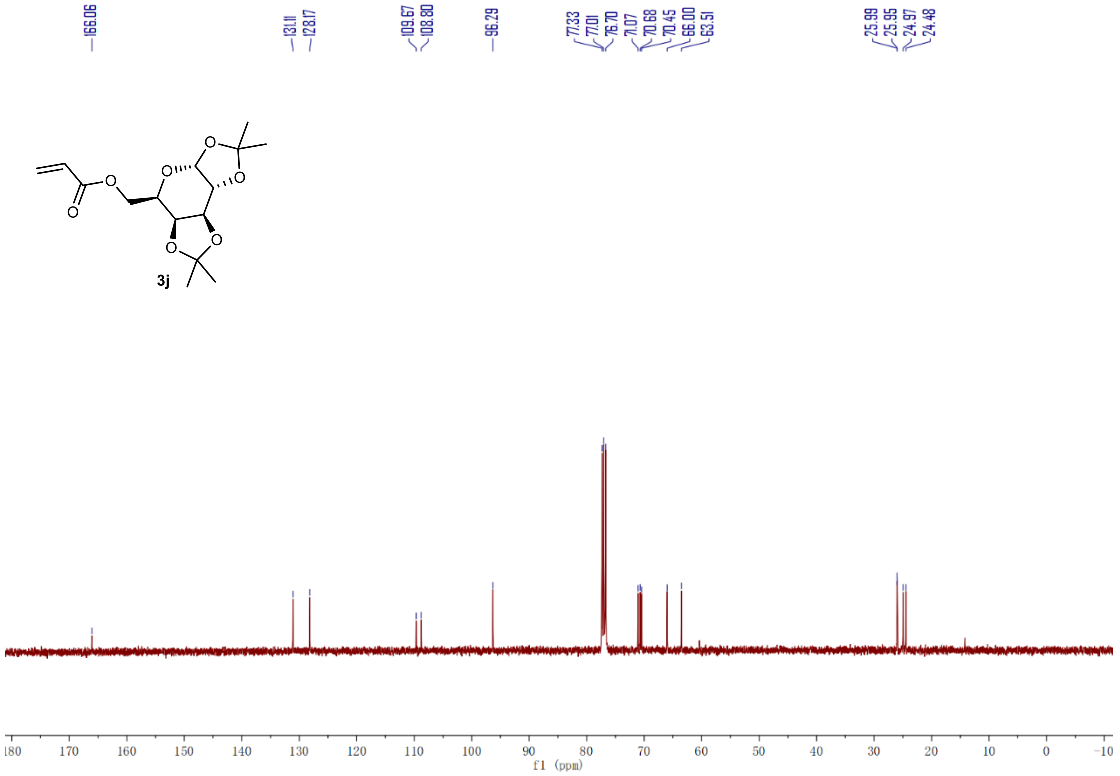

Supplementary Figure 29 ^{13}C spectra of (101 MHz, $CDCl_3$) compound **3j**

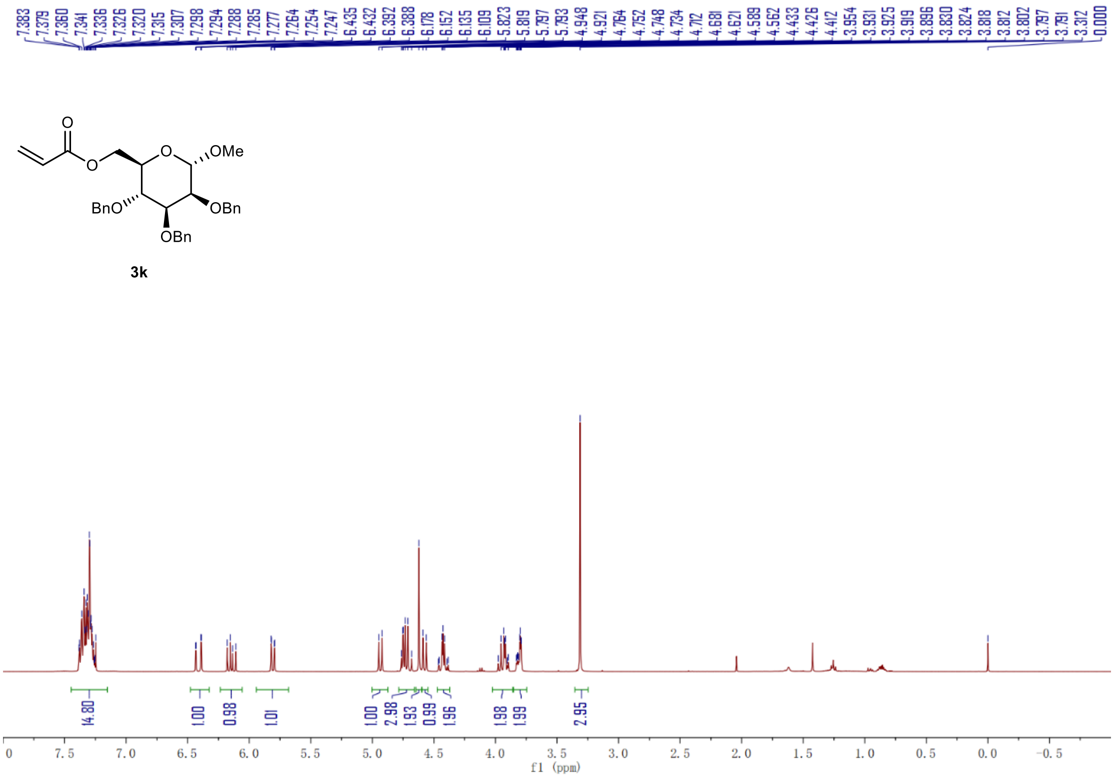

Supplementary Figure 30 ^{1}H NMR spectra of (400 MHz, $CDCl_3$) compound **3k**

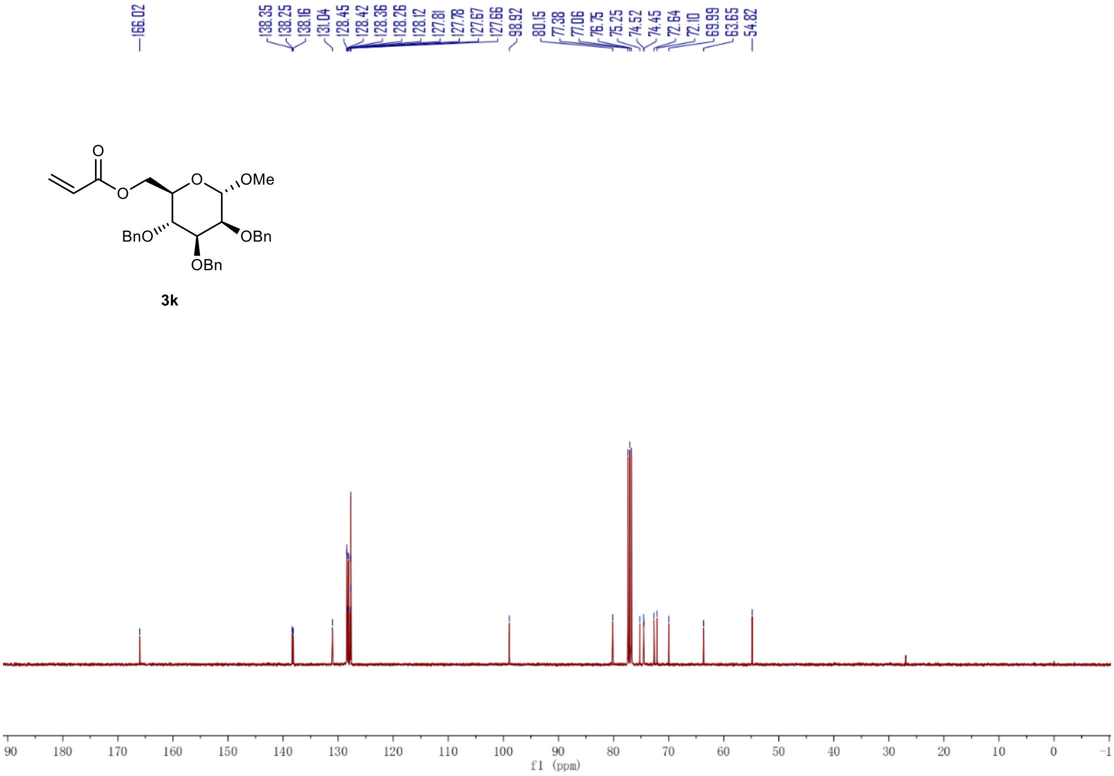

Supplementary Figure 31 ^{13}C spectra of (101 MHz, $CDCl_3$) compound **3k**

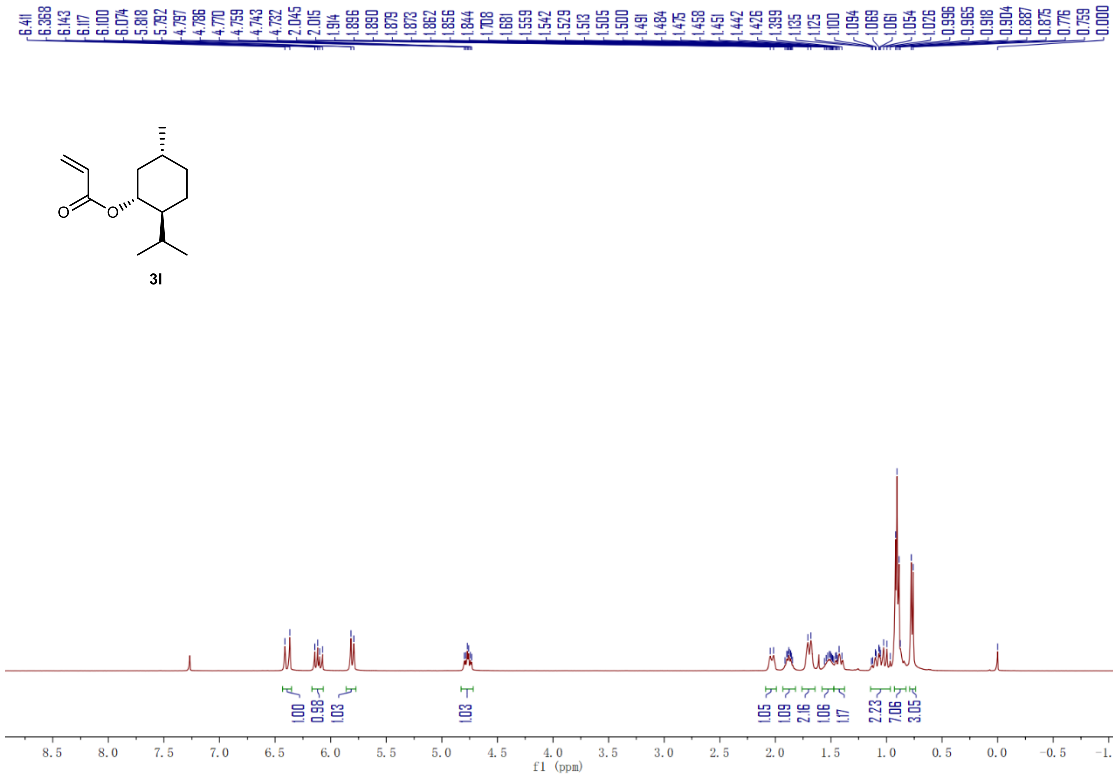

Supplementary Figure 32 ^{1}H NMR spectra of (400 MHz, $CDCl_3$) compound **3l**

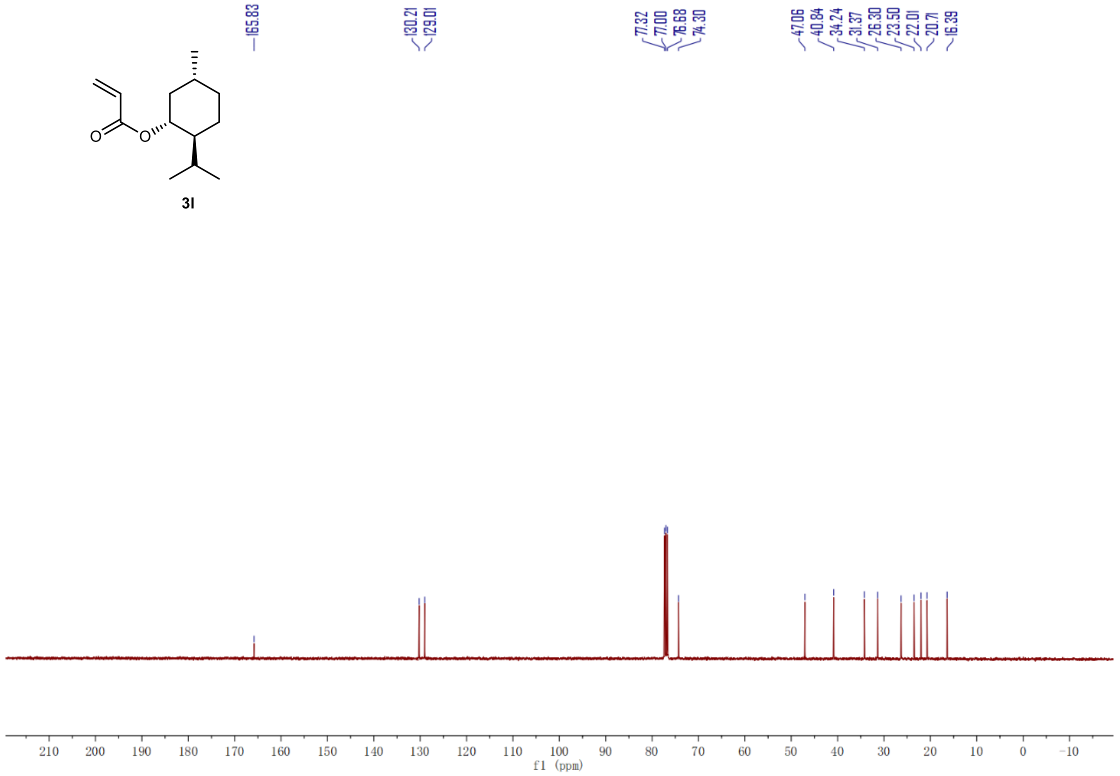

Supplementary Figure 33 ^{13}C spectra of (101 MHz, $CDCl_3$) compound **3l**

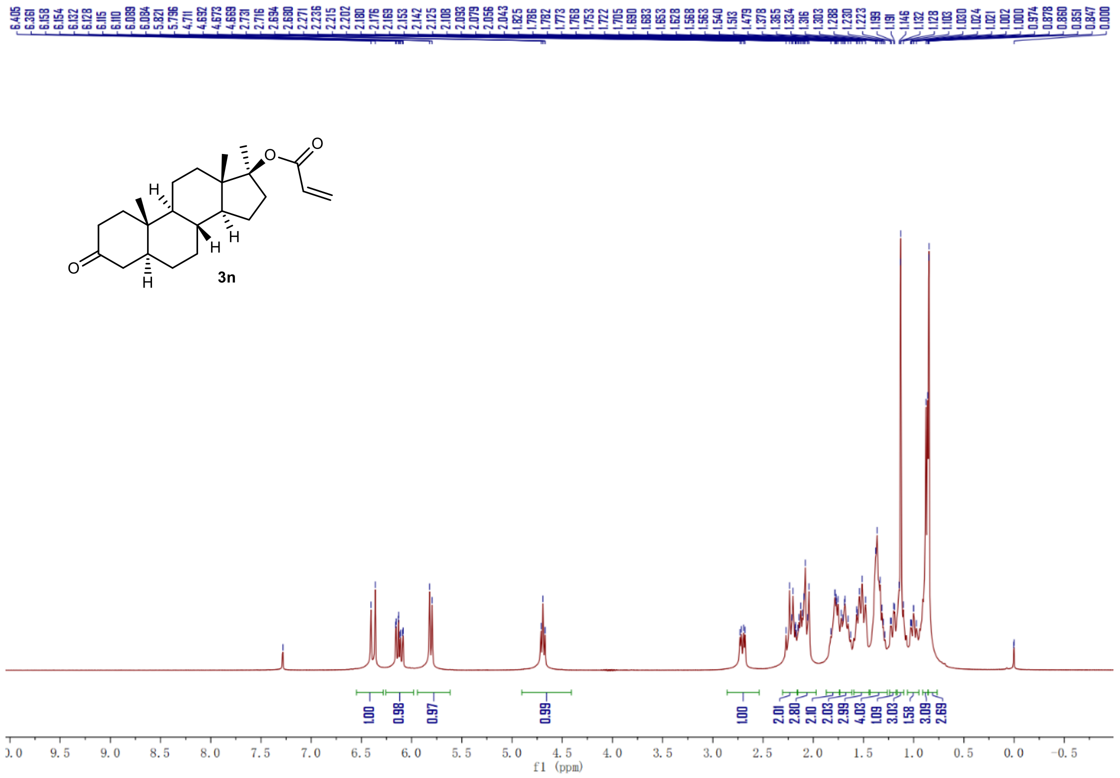

Supplementary Figure 34 ^{1}H NMR spectra of (400 MHz, $CDCl_3$) compound **3n**

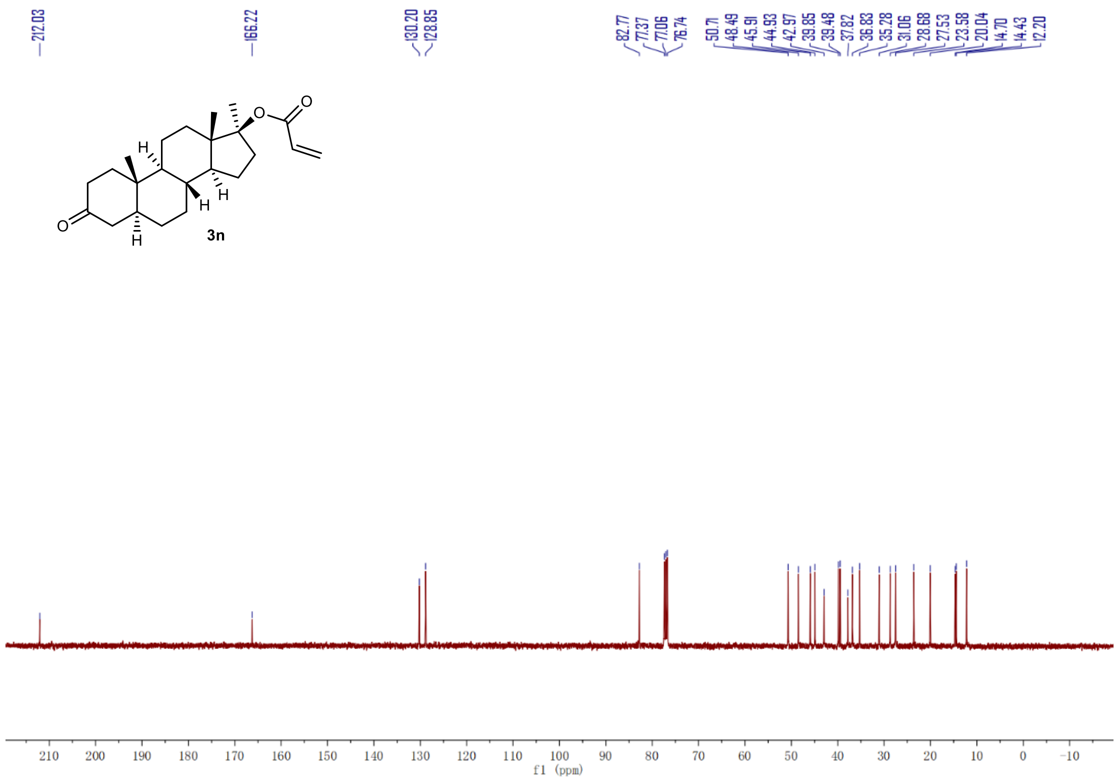

Supplementary Figure 35 ^{13}C spectra of (101 MHz, $CDCl_3$) compound **3n**

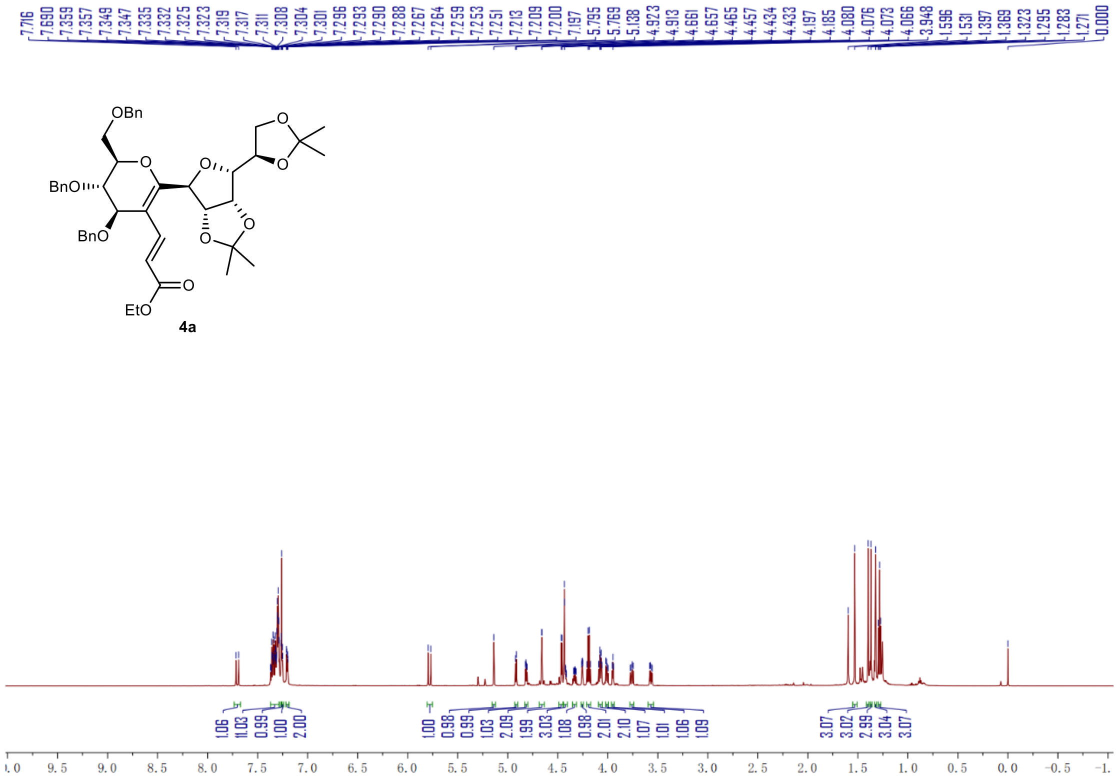

Supplementary Figure 36 ^{1}H NMR spectra of (600 MHz, $CDCl_3$) compound **4a**

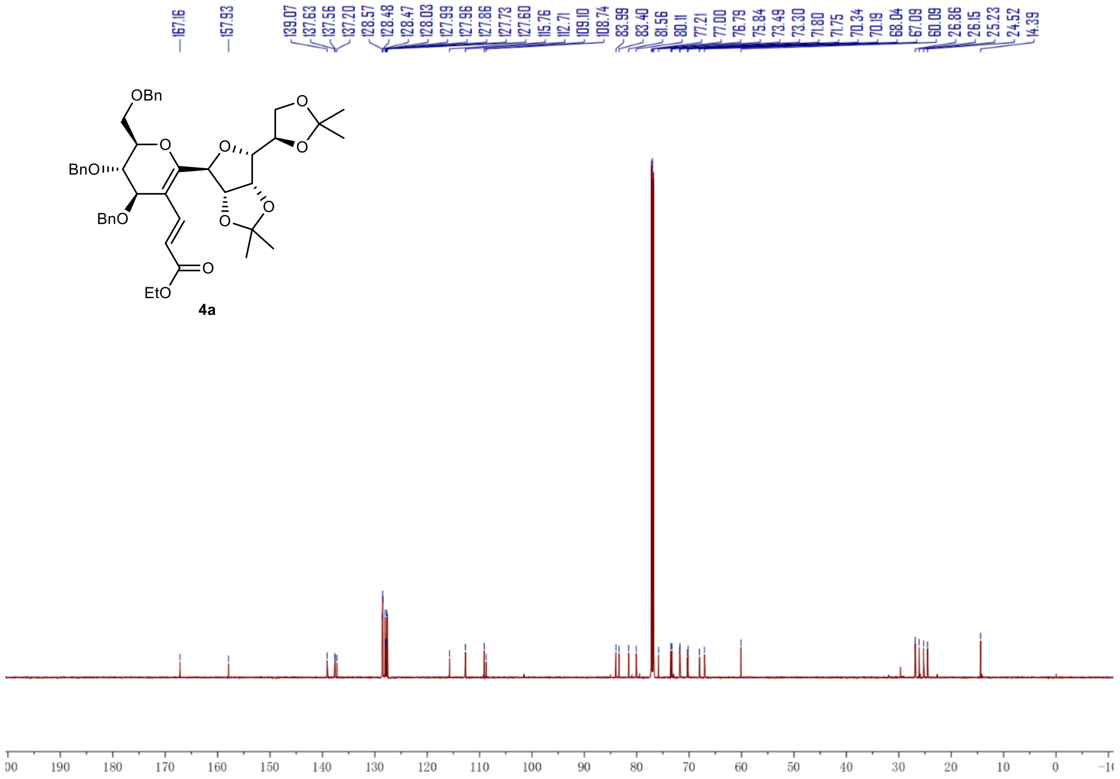

Supplementary Figure 37 ^{13}C spectra of (151 MHz, $CDCl_3$) compound **4**a

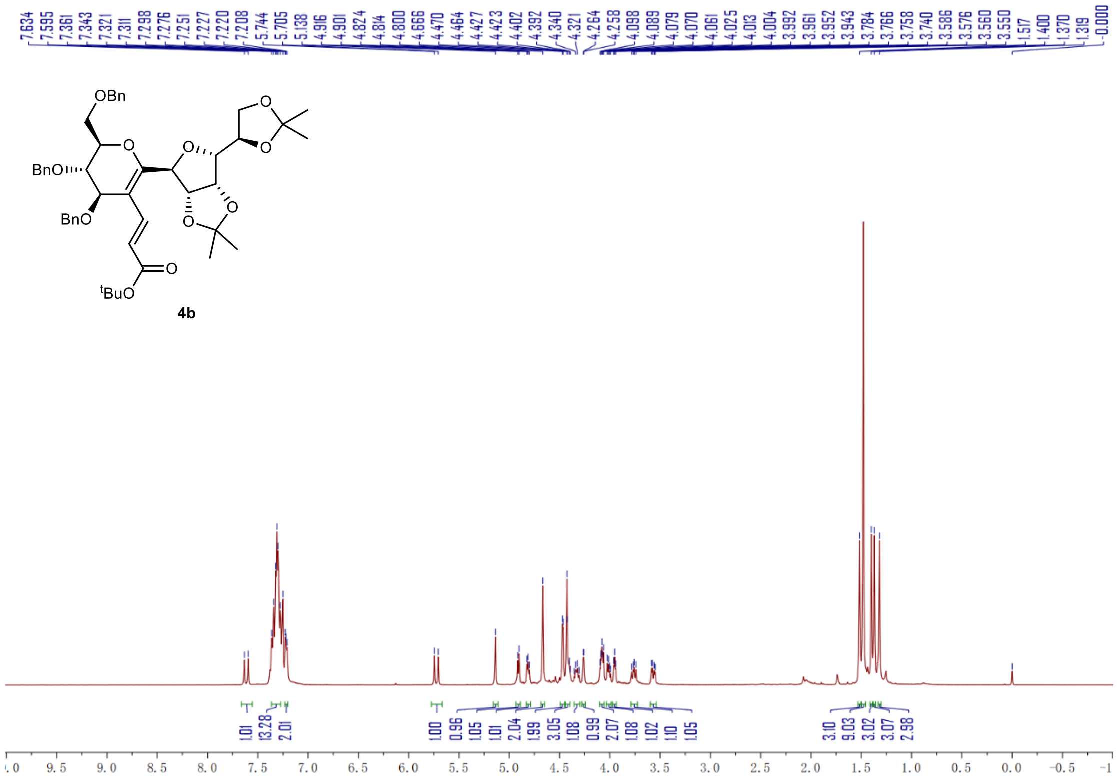

Supplementary Figure 38 ^{1}H NMR spectra of (400 MHz, $CDCl_3$) compound **4b**

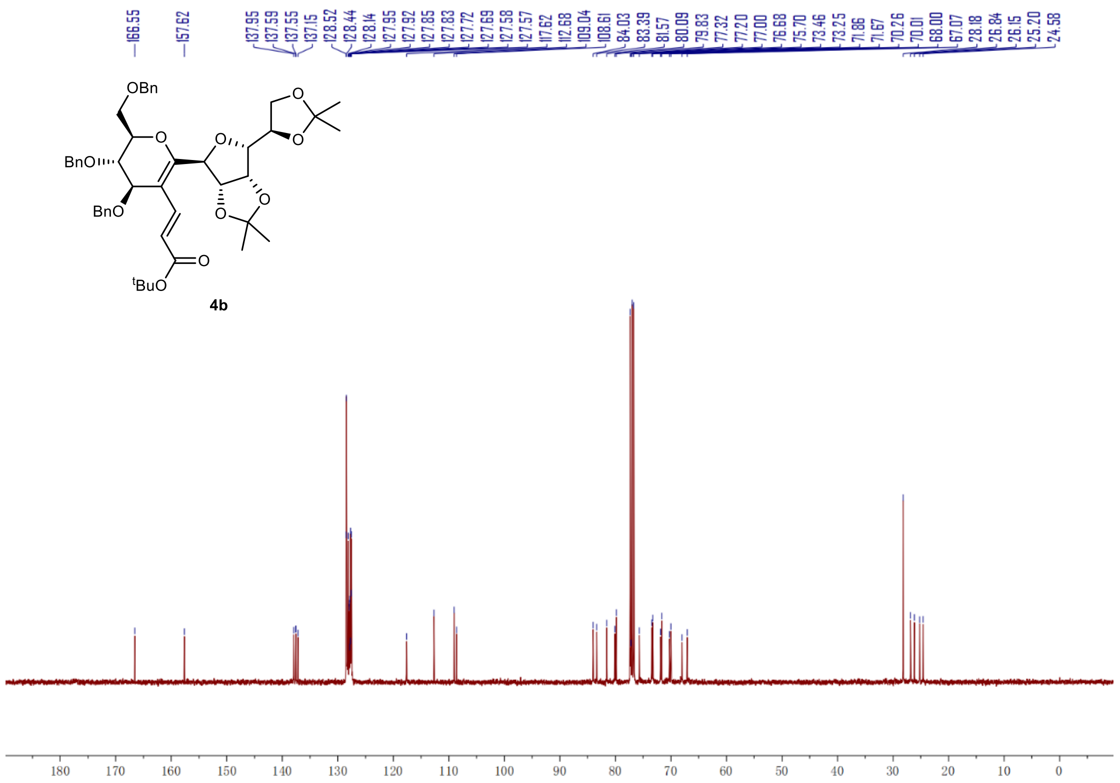

Supplementary Figure 39 ^{13}C spectra of (101 MHz, $CDCl_3$) compound **4b**

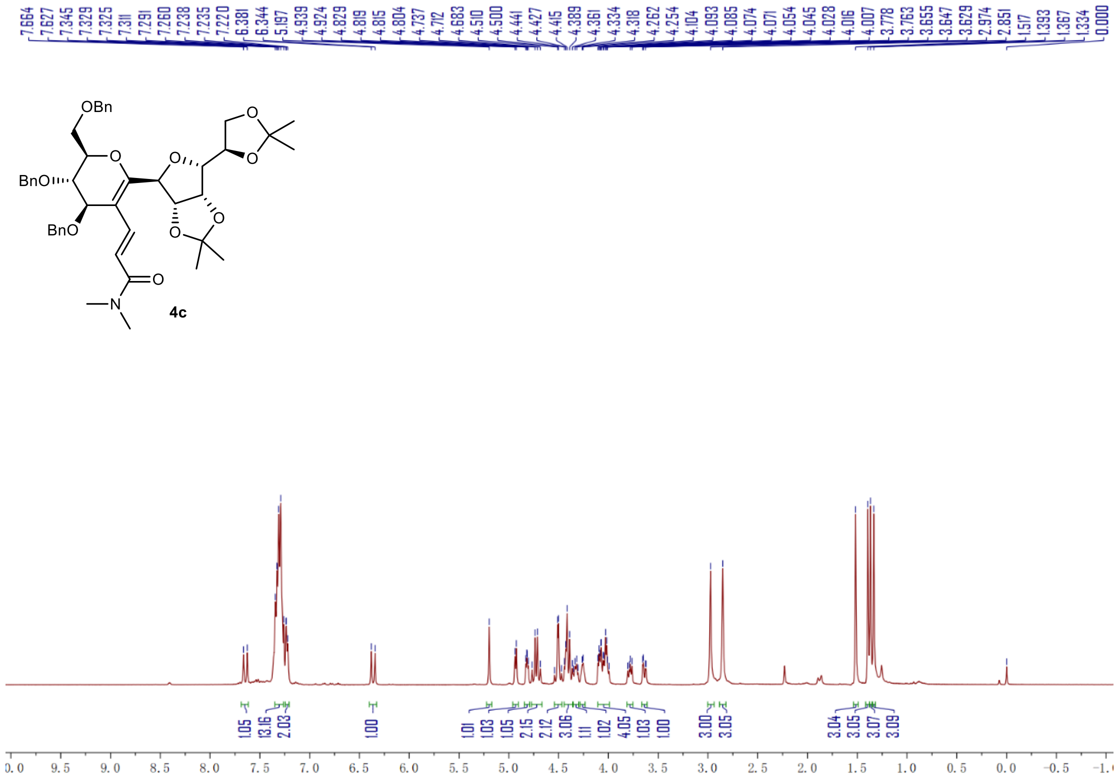

Supplementary Figure 40 ^{1}H NMR spectra of (400 MHz, $CDCl_3$) compound **4c**

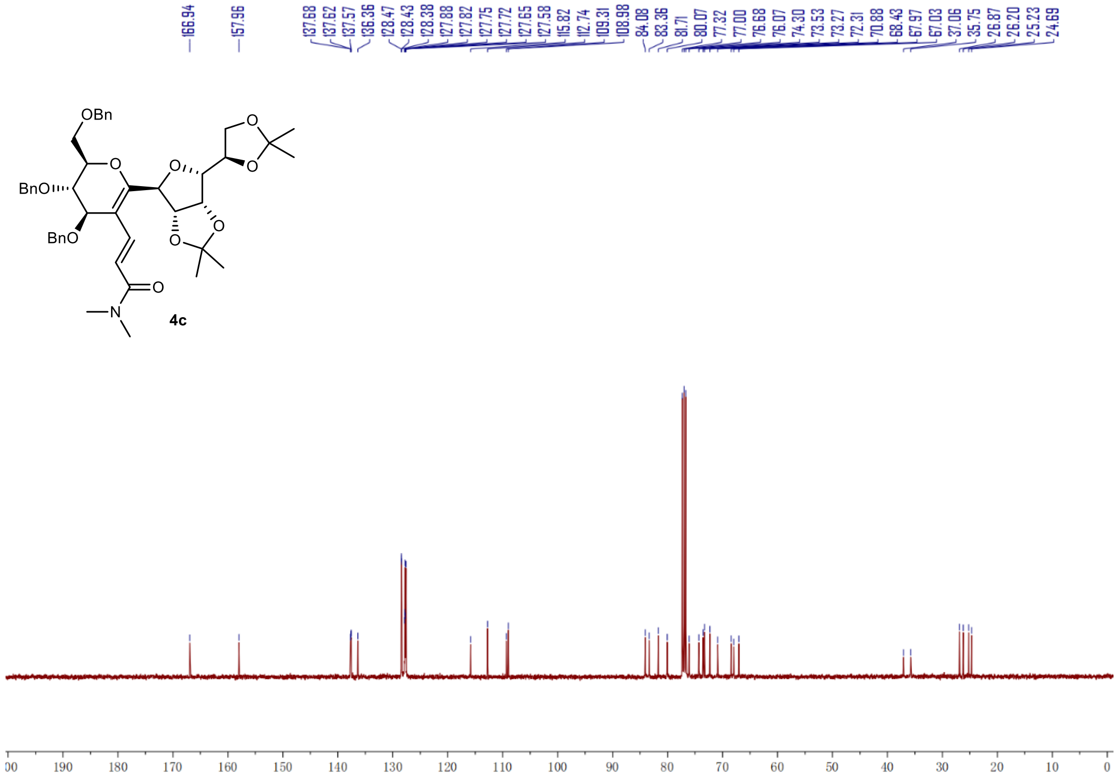

Supplementary Figure 41 ^{13}C spectra of (101 MHz, $CDCl_3$) compound **4c**

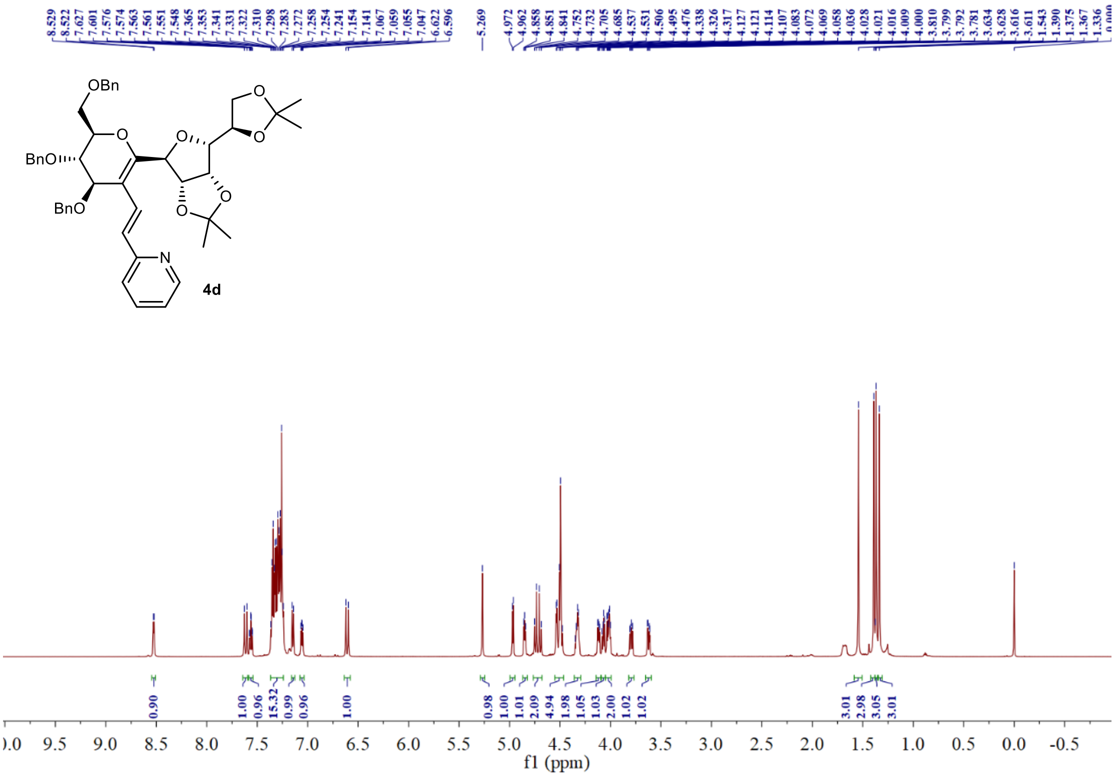

Supplementary Figure 42 ^{1}H NMR spectra of (400 MHz, $CDCl_3$) compound **4d**

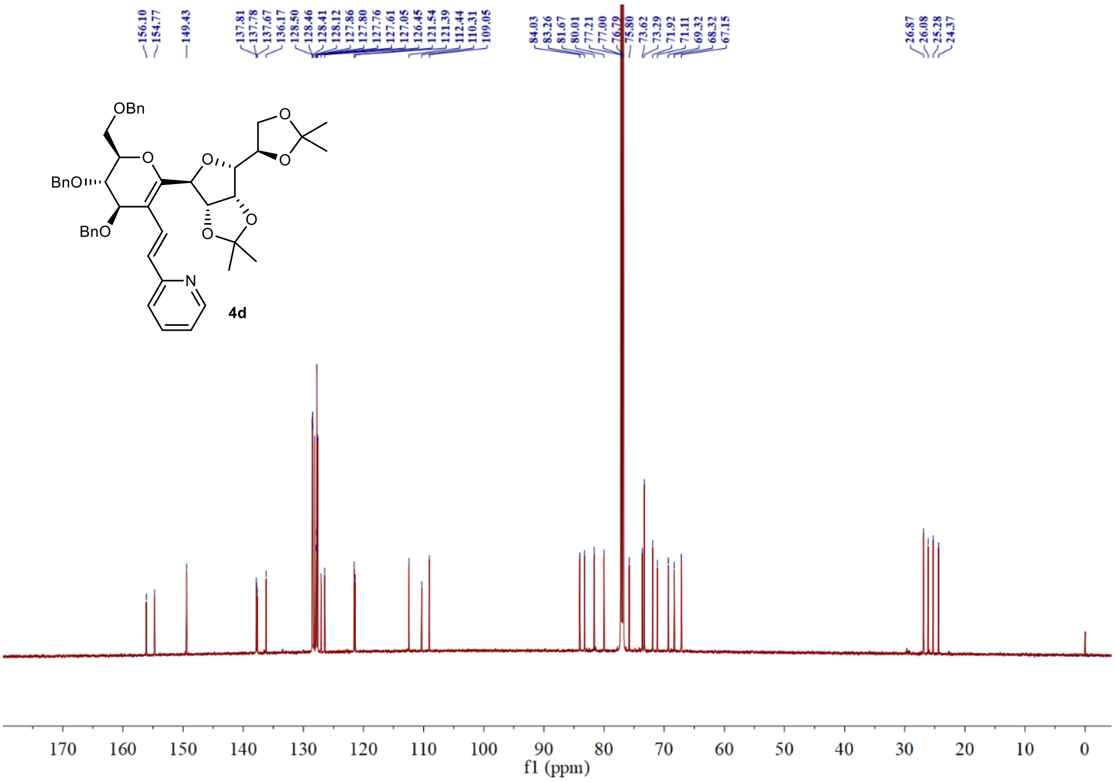

Supplementary Figure 43 ^{13}C spectra of (101 MHz, $CDCl_3$) compound **4d**

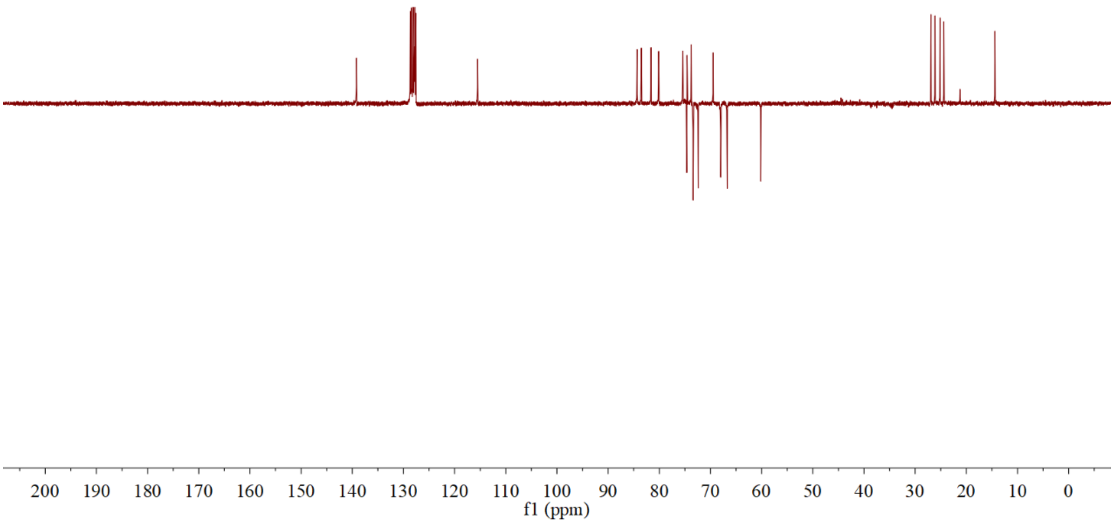

Supplementary Figure 44 DEPT spectra of (101 MHz, $CDCl_3$) compound **4d**

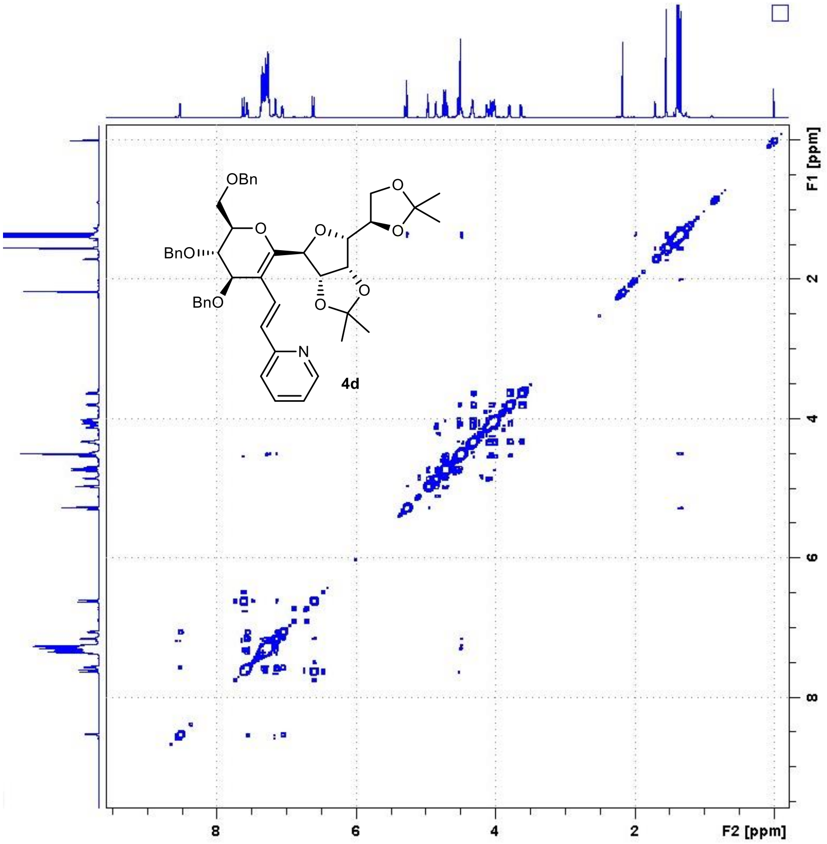

Supplementary Figure 45 COSY spectra of compound **4d**

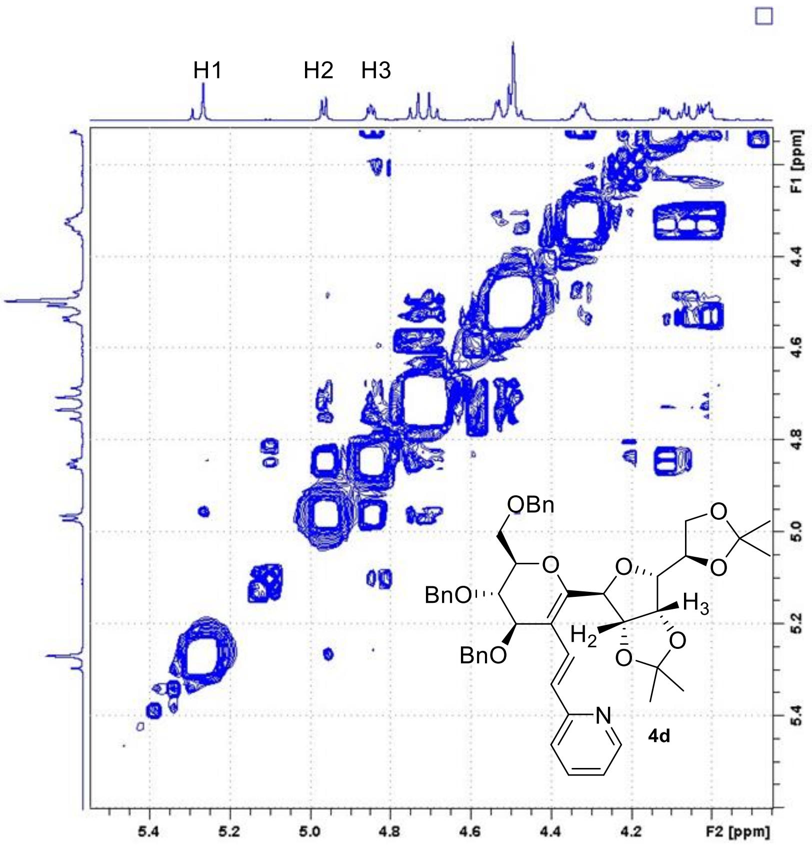

Supplementary Figure 46 COSY spectra of compound **4d**

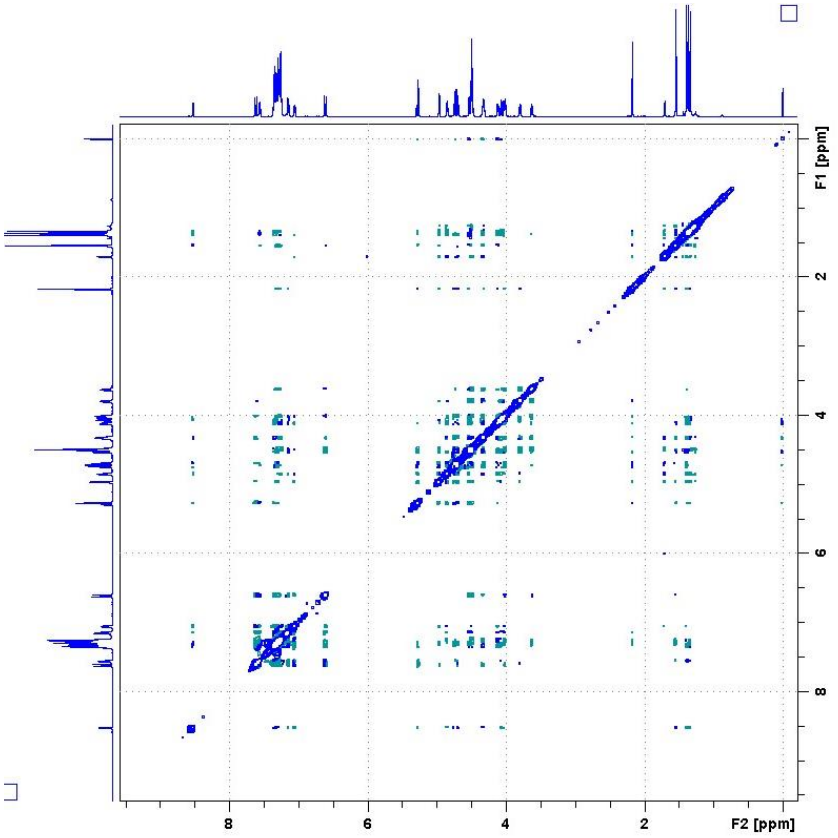

Supplementary Figure 47 NOESY spectra of compound **4d**

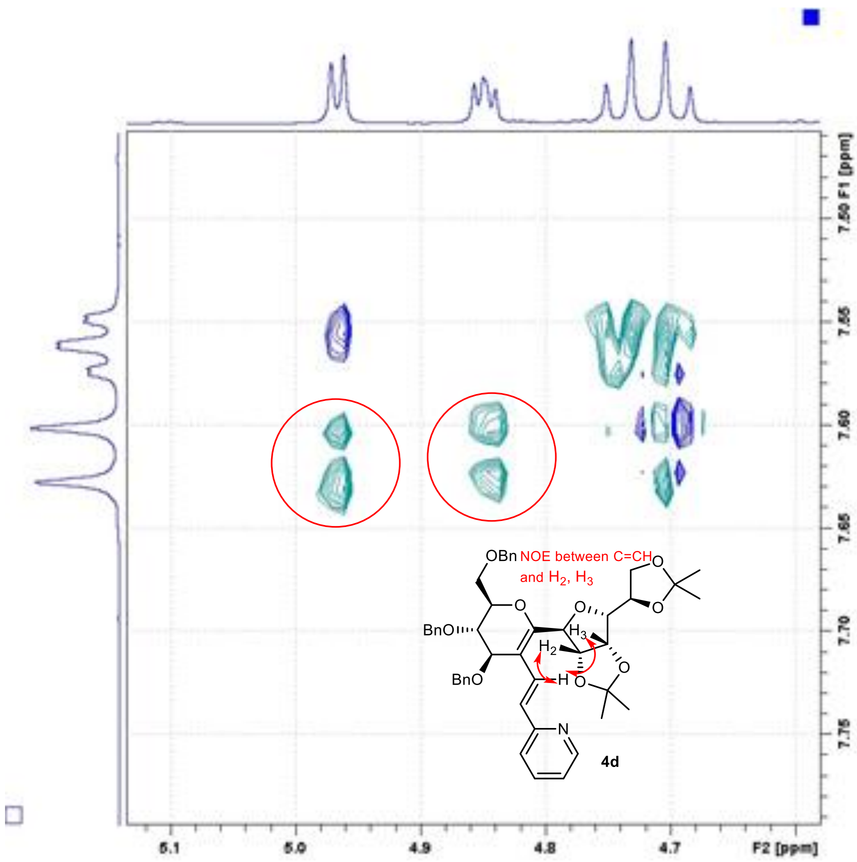

Supplementary Figure 48 NOESY spectra of compound **4d**

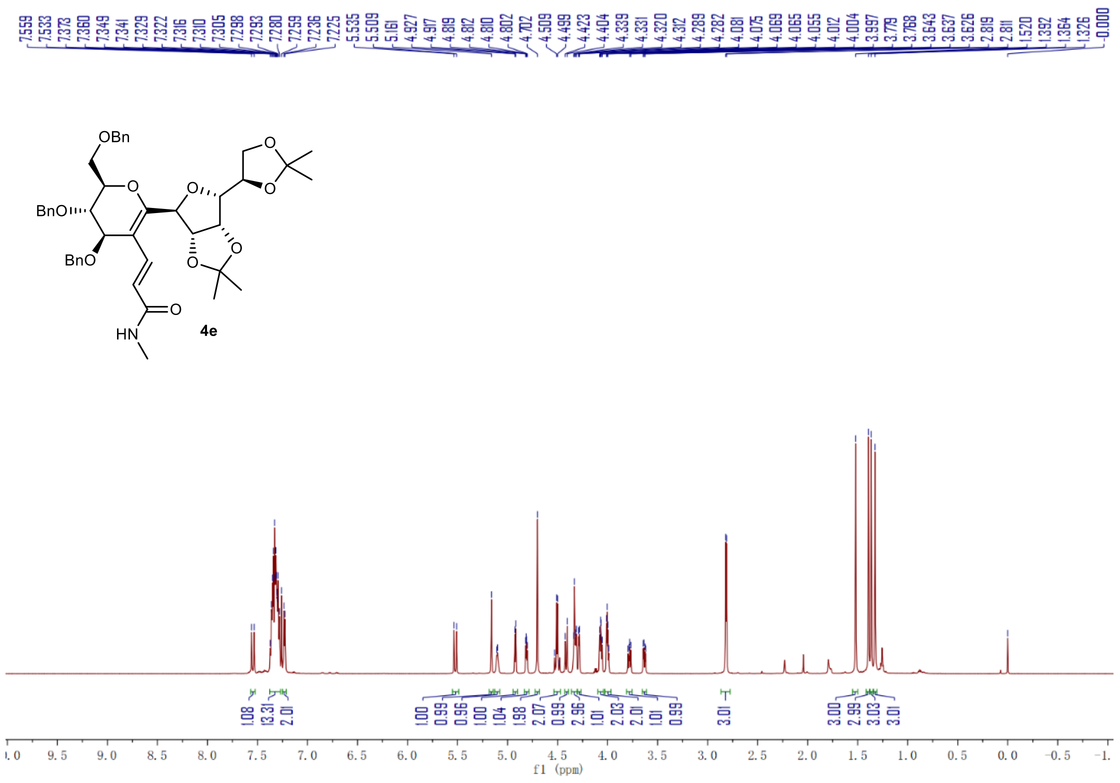

Supplementary Figure 49 ^{1}H NMR spectra of (600 MHz, $CDCl_3$) compound **4e**

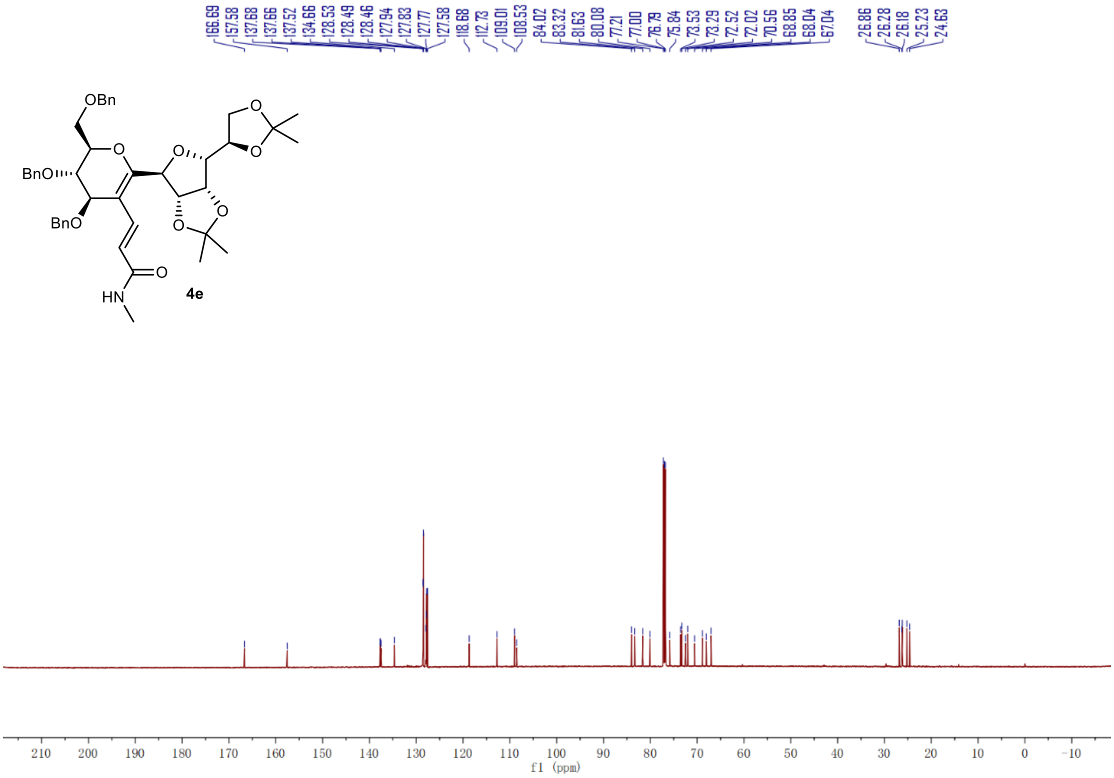

Supplementary Figure 50 ^{13}C spectra of (151 MHz, $CDCl_3$) compound **4e**

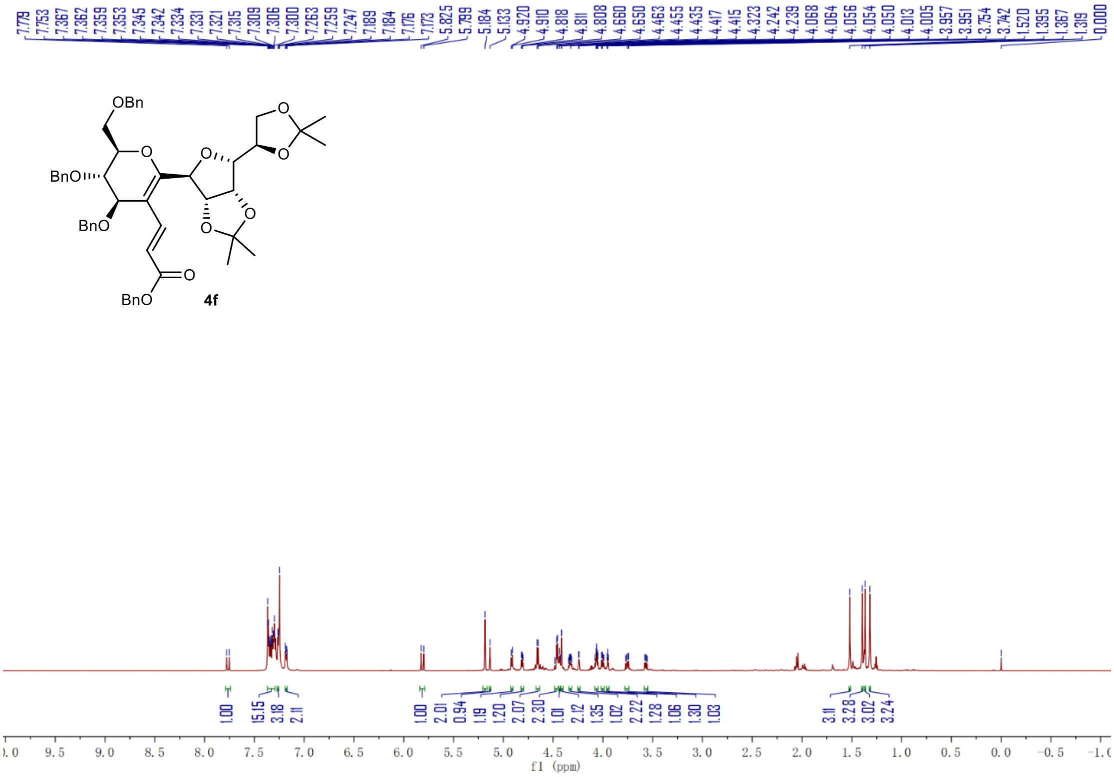

Supplementary Figure 51 ^{1}H NMR spectra of (600 MHz, $CDCl_3$) compound **4f**

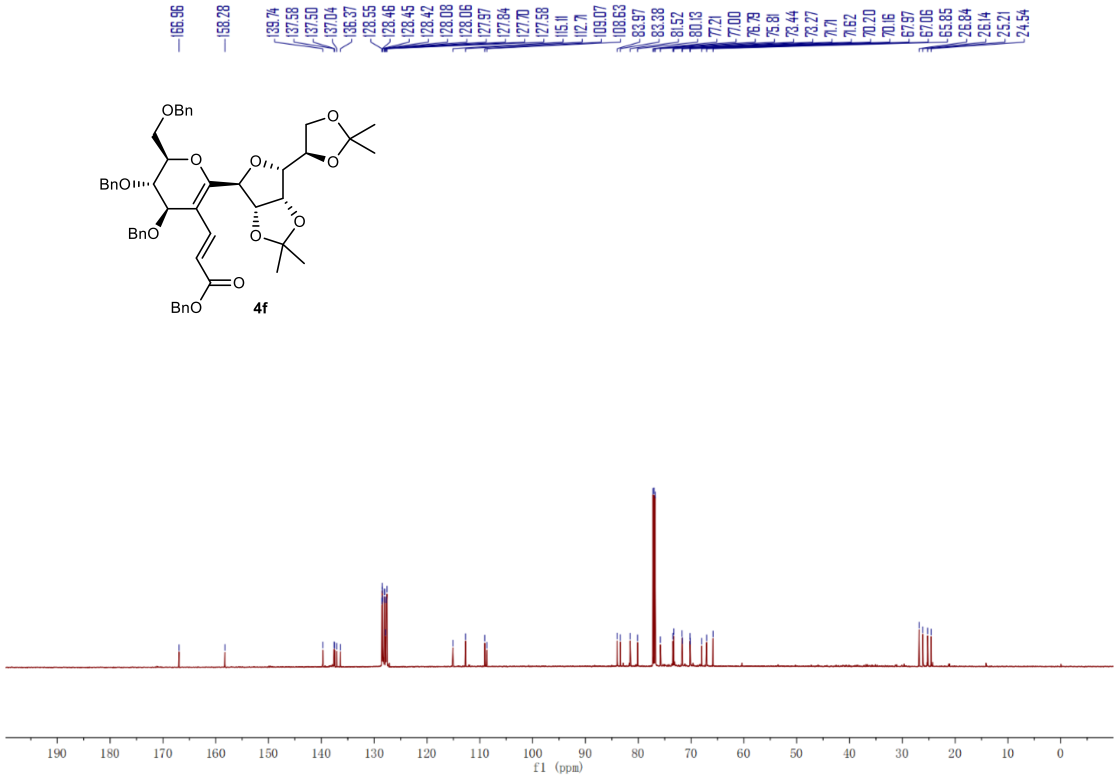

Supplementary Figure 52 ^{13}C spectra of (151 MHz, $CDCl_3$) compound **4f**

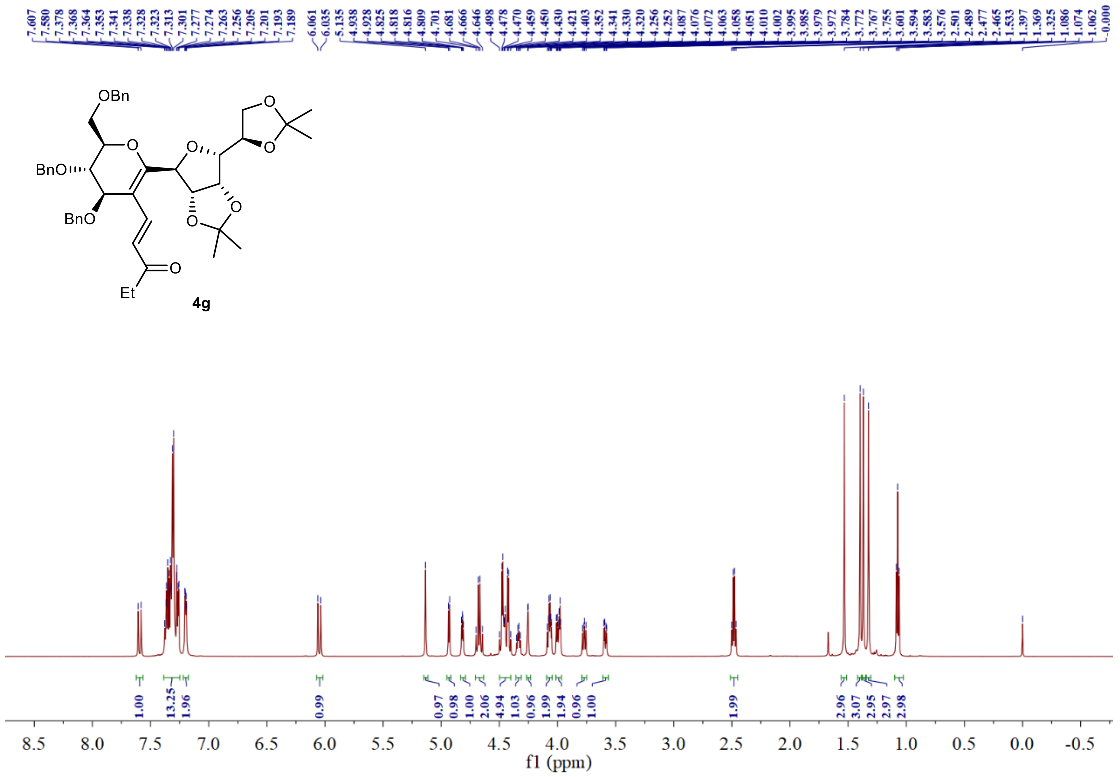

Supplementary Figure 53 ^{1}H NMR spectra of (600 MHz, $CDCl_3$) compound **4g**

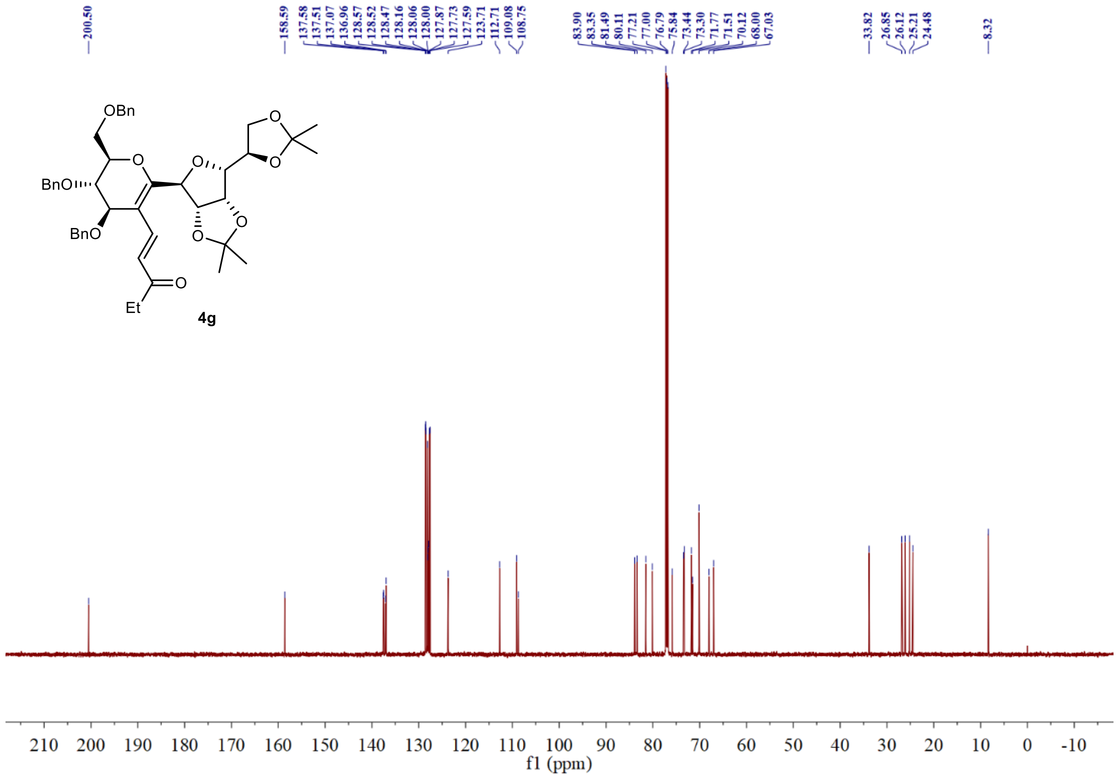

Supplementary Figure 54 ^{13}C spectra of (151 MHz, $CDCl_3$) compound **4g**

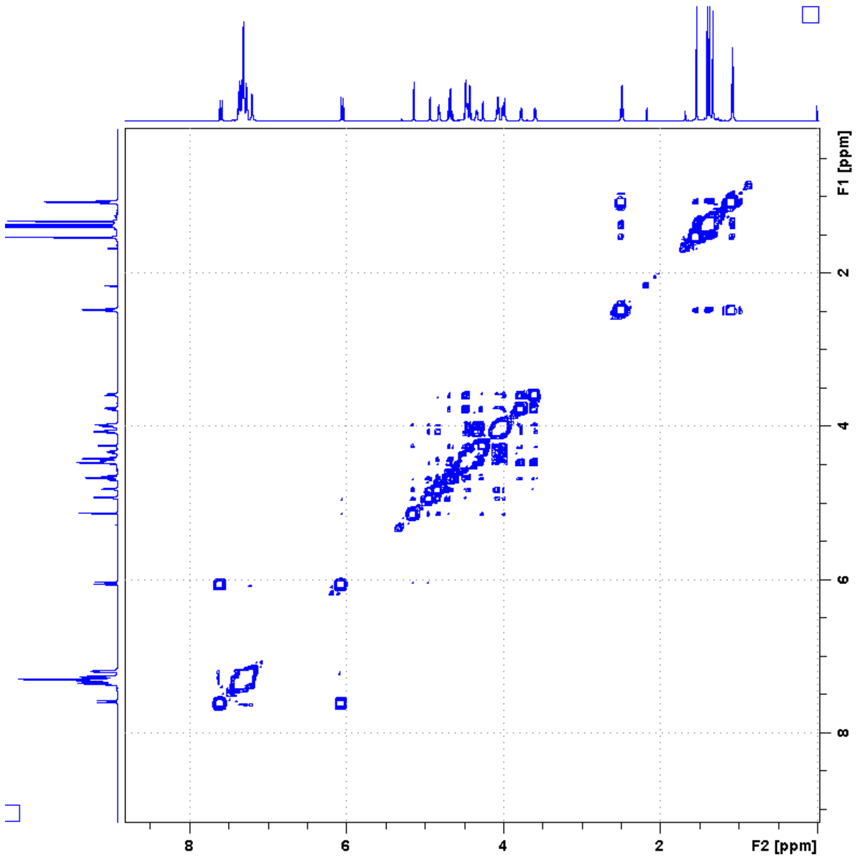

Supplementary Figure 55 COSY spectra of compound **4g**

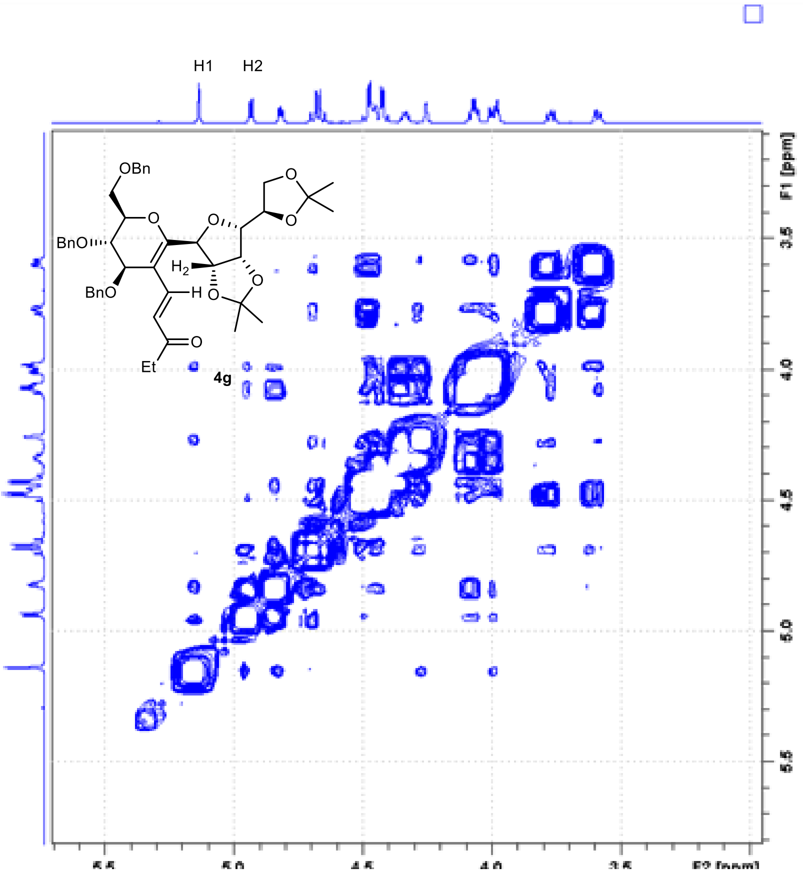

Supplementary Figure 56 COSY spectra of compound **4g**

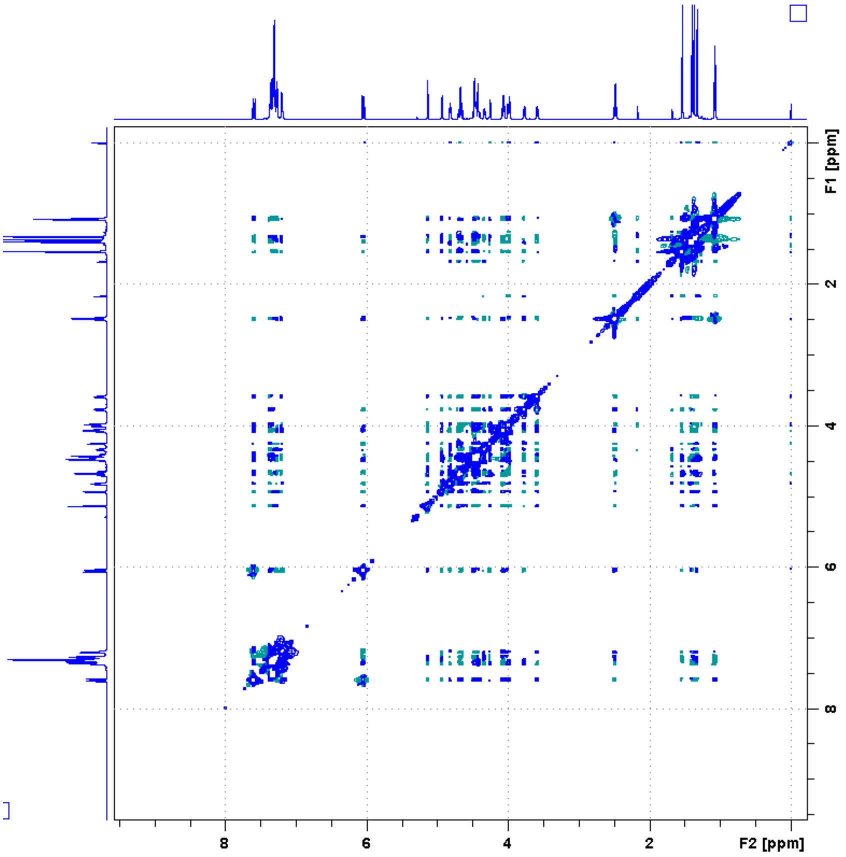

Supplementary Figure 57 NOESY spectra of compound **4g**

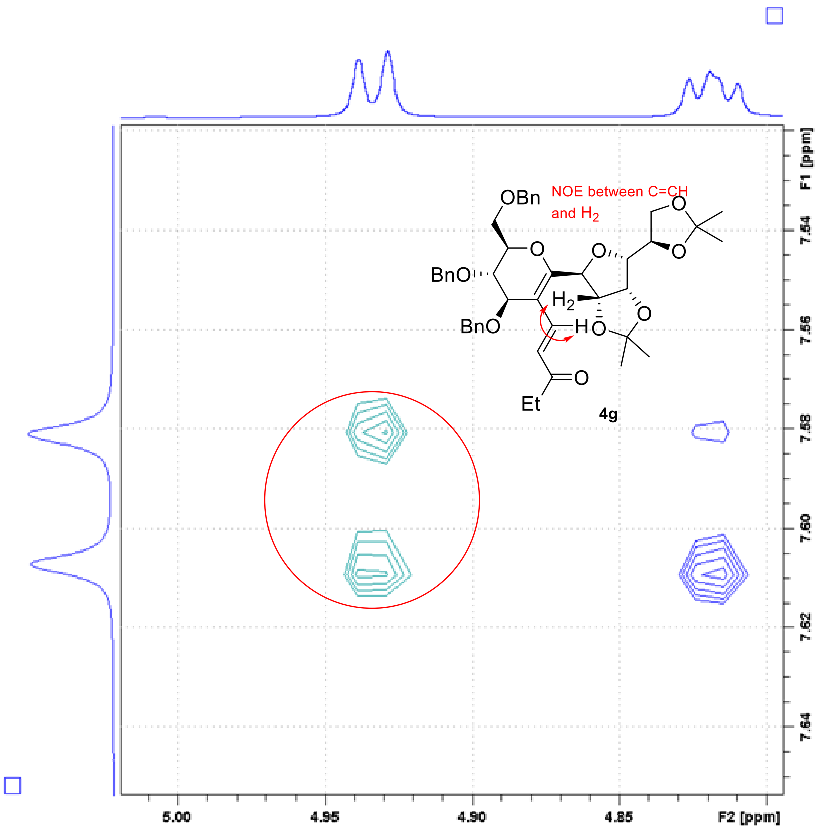

Supplementary Figure 58 NOESY spectra of compound **4g**

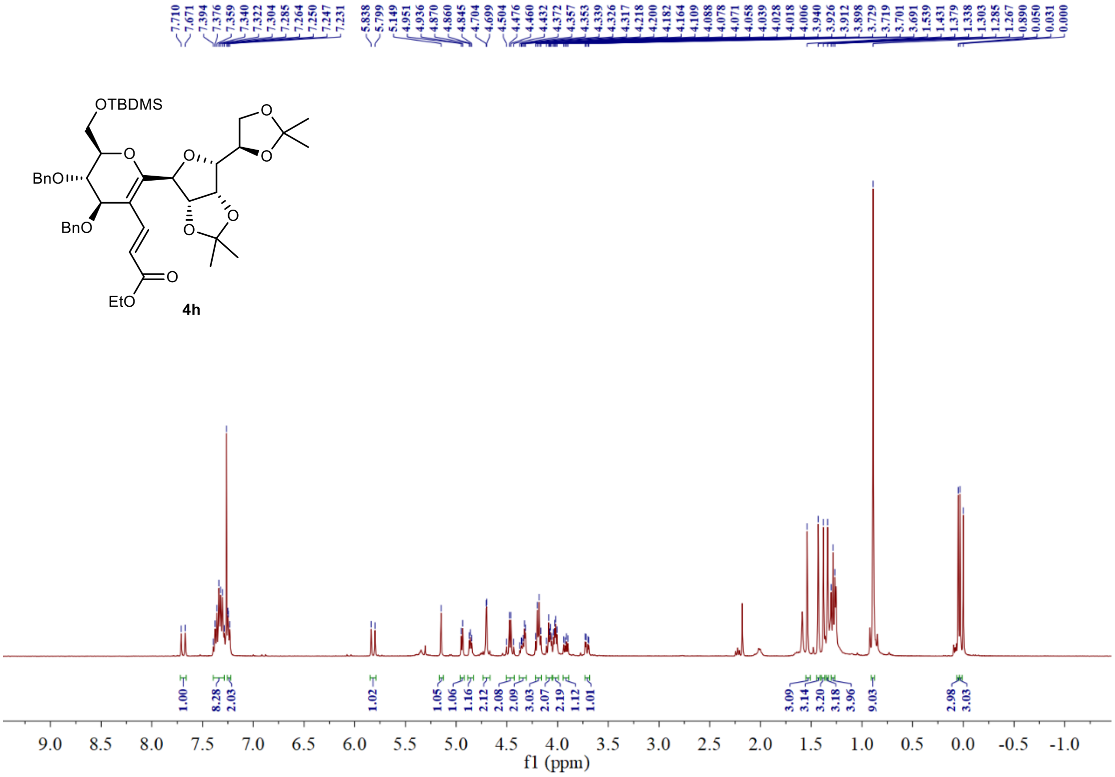

Supplementary Figure 59 ^{1}H NMR spectra of (400 MHz, $CDCl_3$) compound **4h**

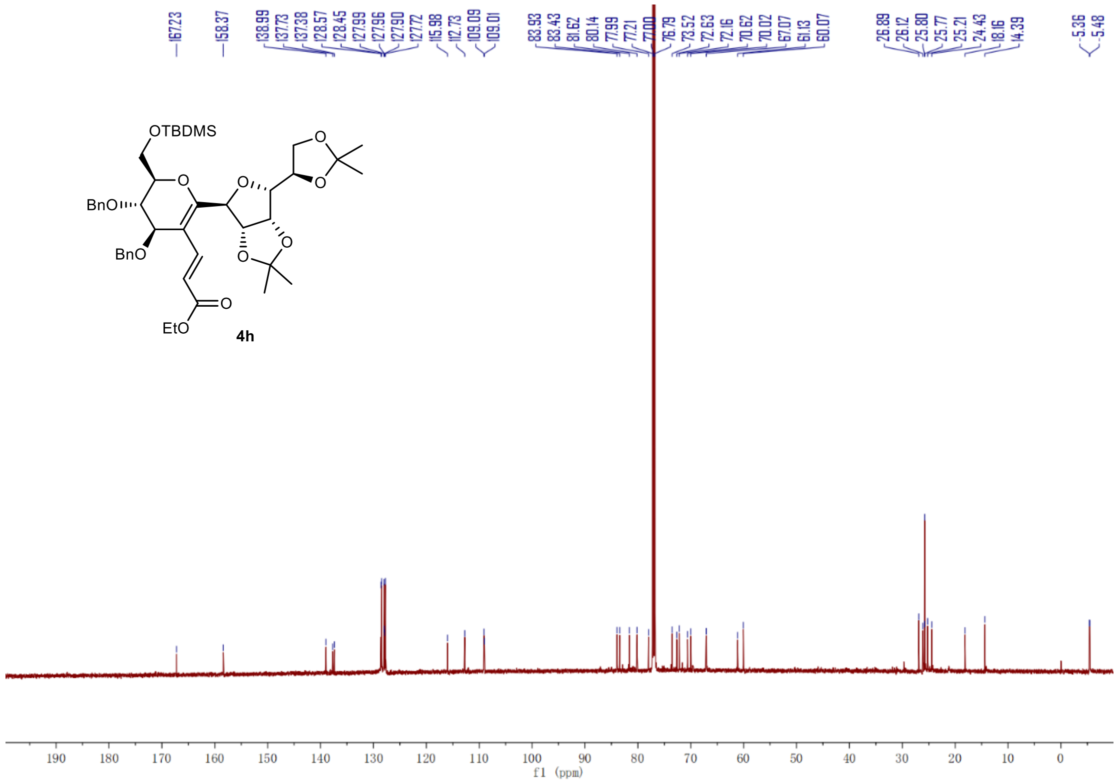

Supplementary Figure 60 ^{13}C spectra of (151 MHz, $CDCl_3$) compound **4h**

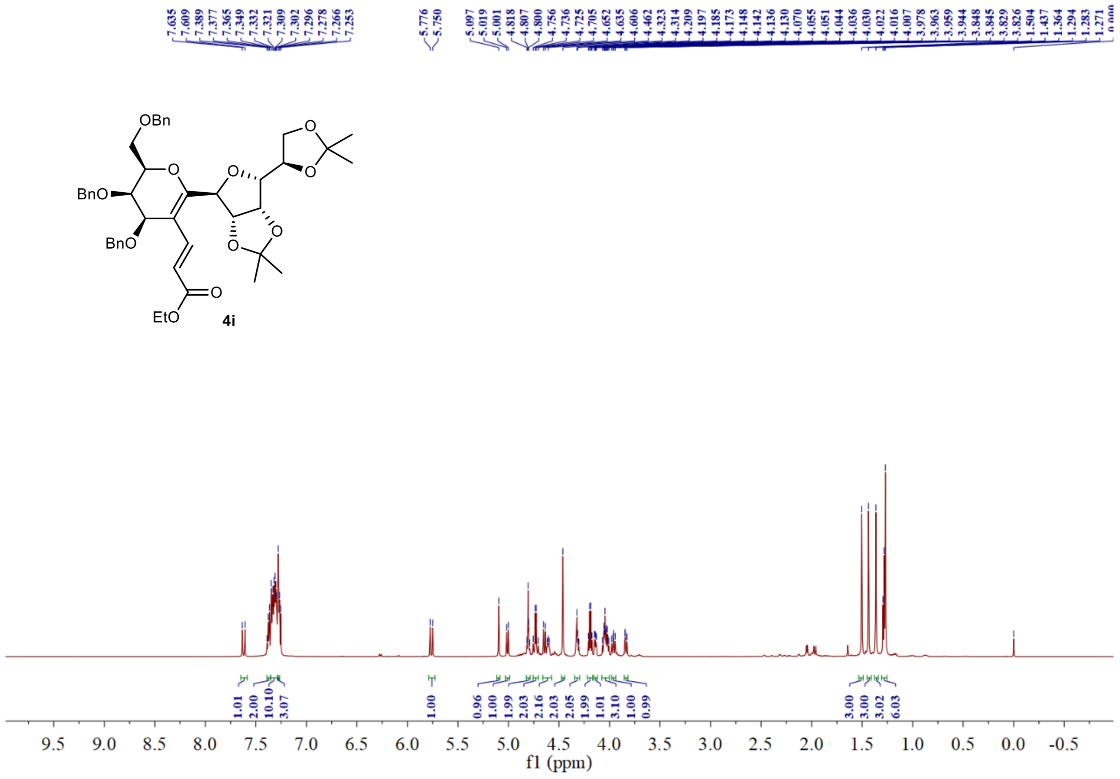

Supplementary Figure 61 ^{1}H NMR spectra of (600 MHz, $CDCl_3$) compound **4i**

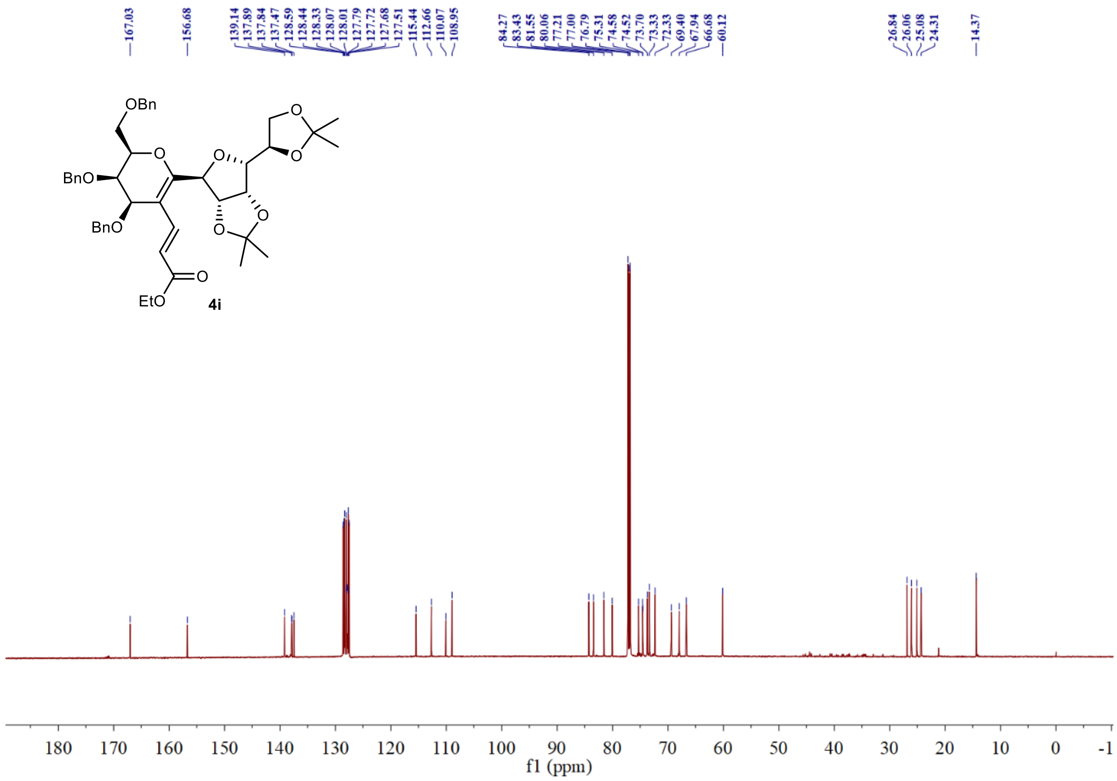

Supplementary Figure 62 ^{13}C spectra of (151 MHz, $CDCl_3$) compound **4i**

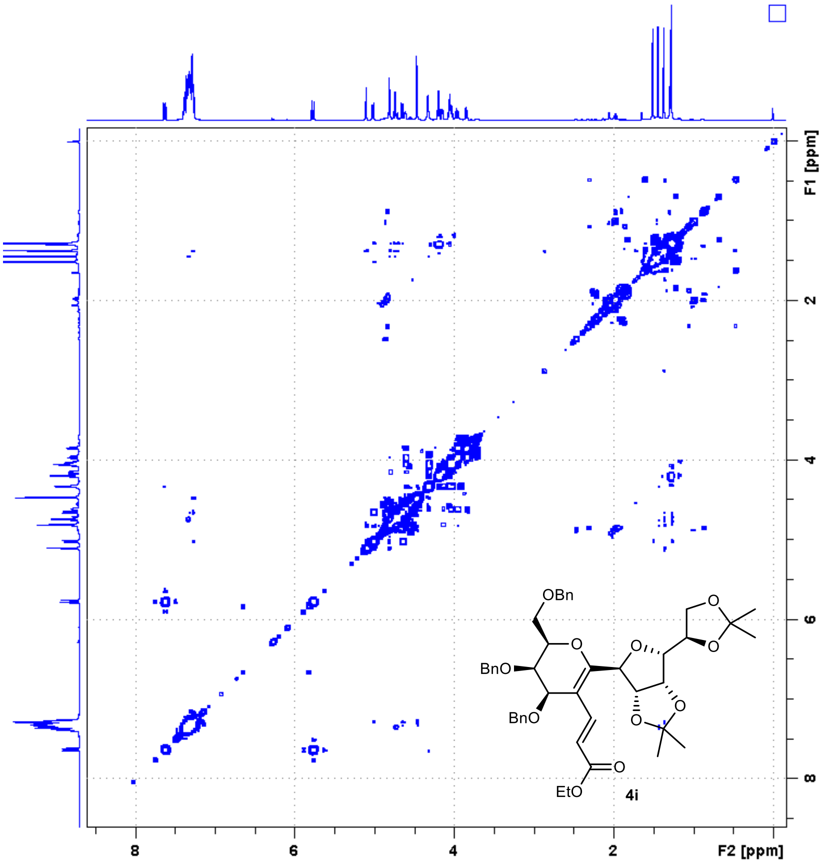

Supplementary Figure 63 COSY spectra of compound **4i**

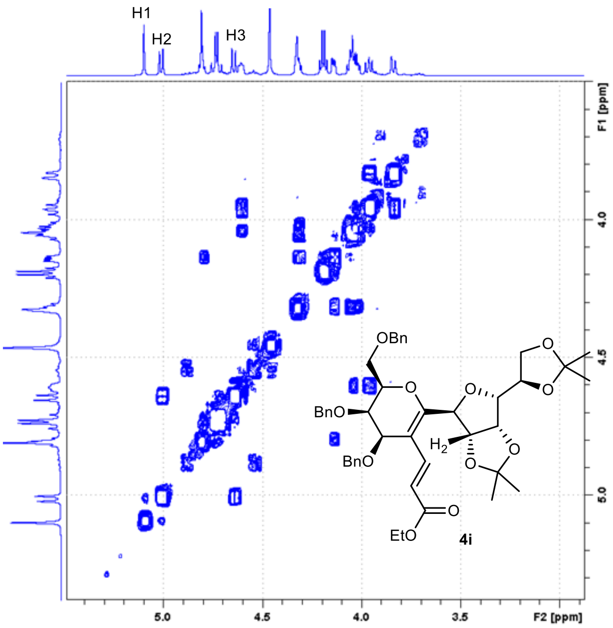

Supplementary Figure 64 COSY spectra of compound **4i**

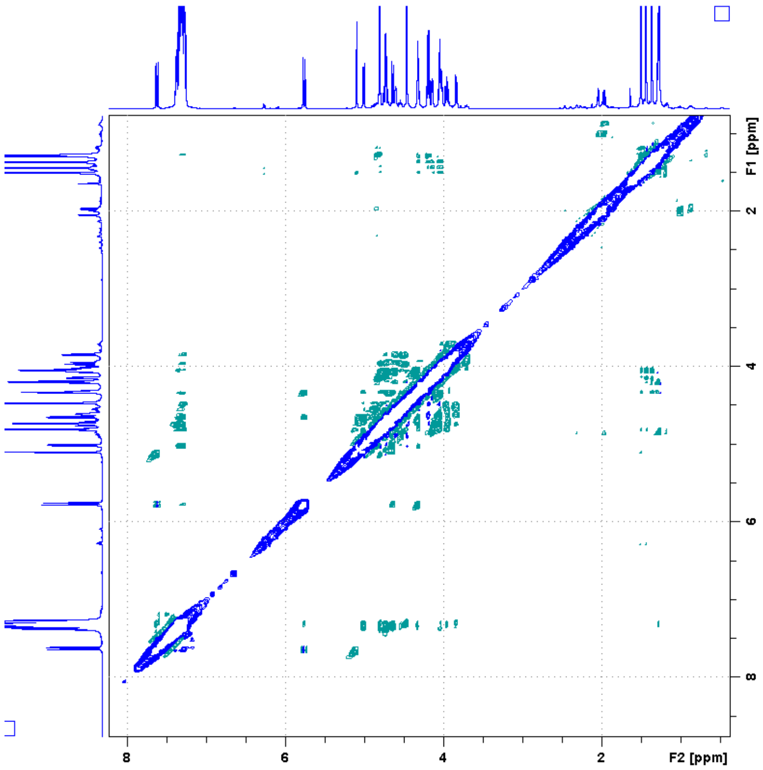

Supplementary Figure 65 NOESY spectra of compound **4i**

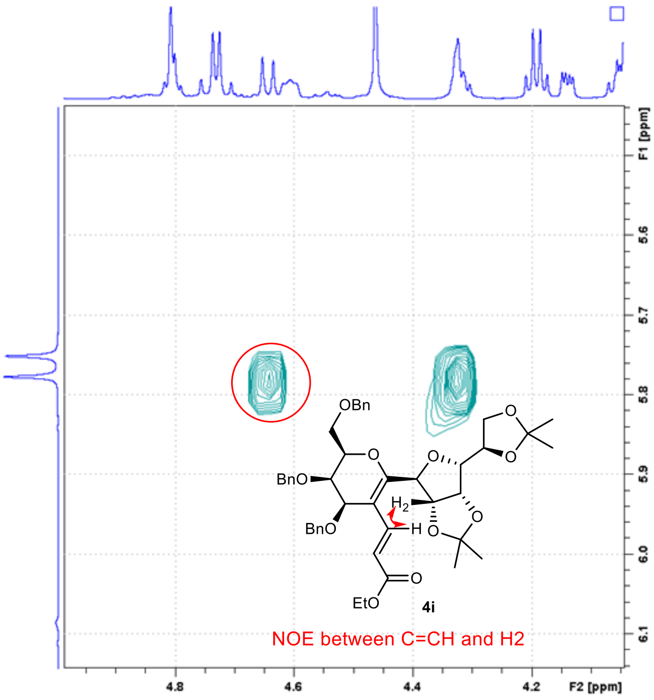

Supplementary Figure 66 NOESY spectra of compound **4i**

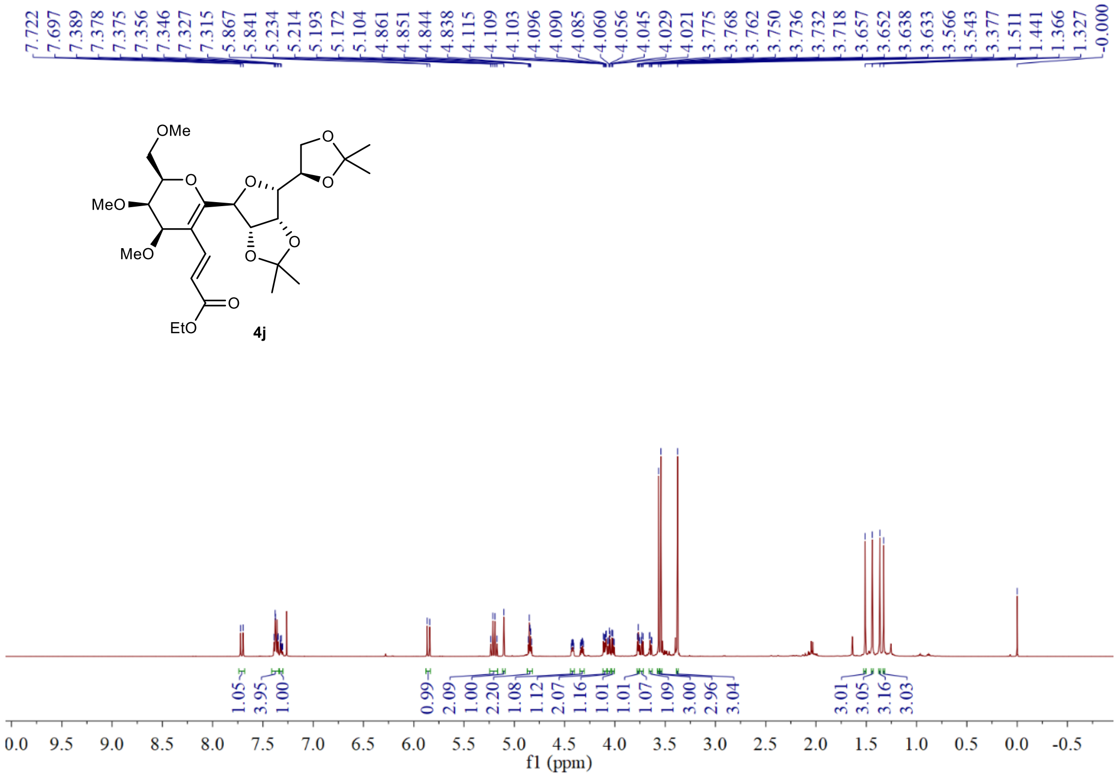

Supplementary Figure 67 ^{1}H NMR spectra of (600 MHz, $CDCl_3$) compound **4j**

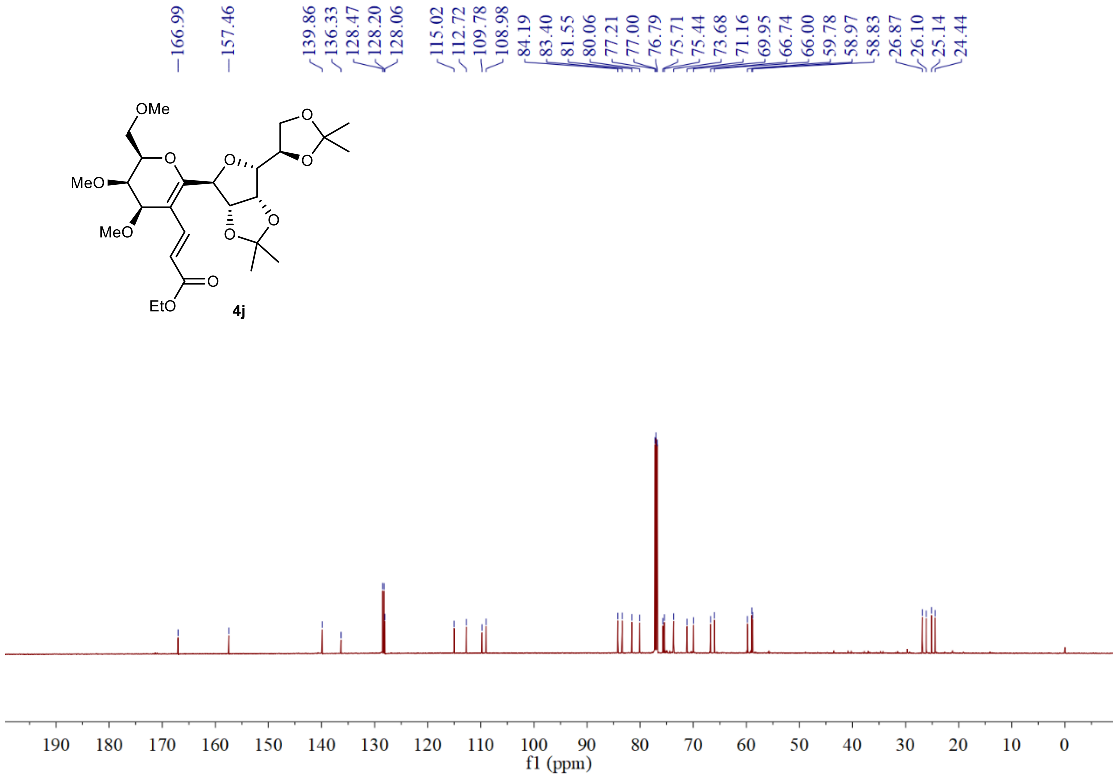

Supplementary Figure 68 ^{13}C spectra of (151 MHz, $CDCl_3$) compound **4j**

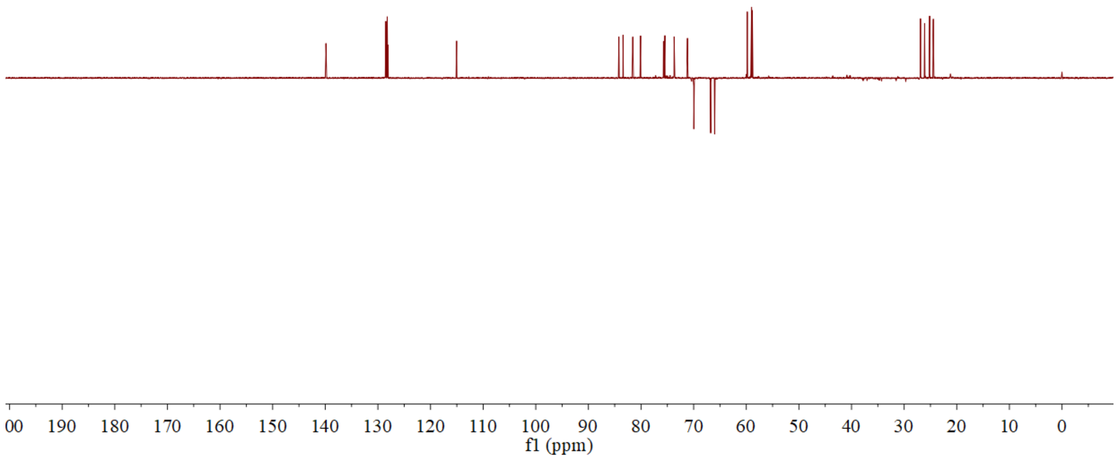

Supplementary Figure 69 DEPT spectra of compound **4j**

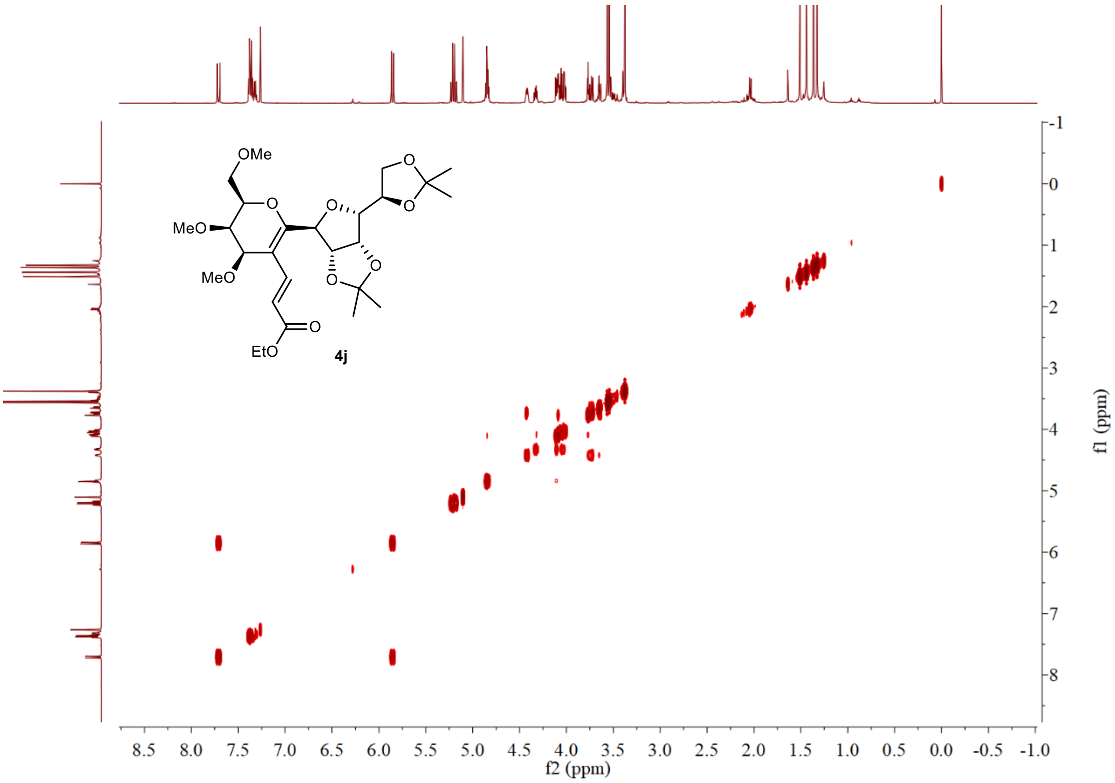

Supplementary Figure 70 COSY spectra of compound **4j**

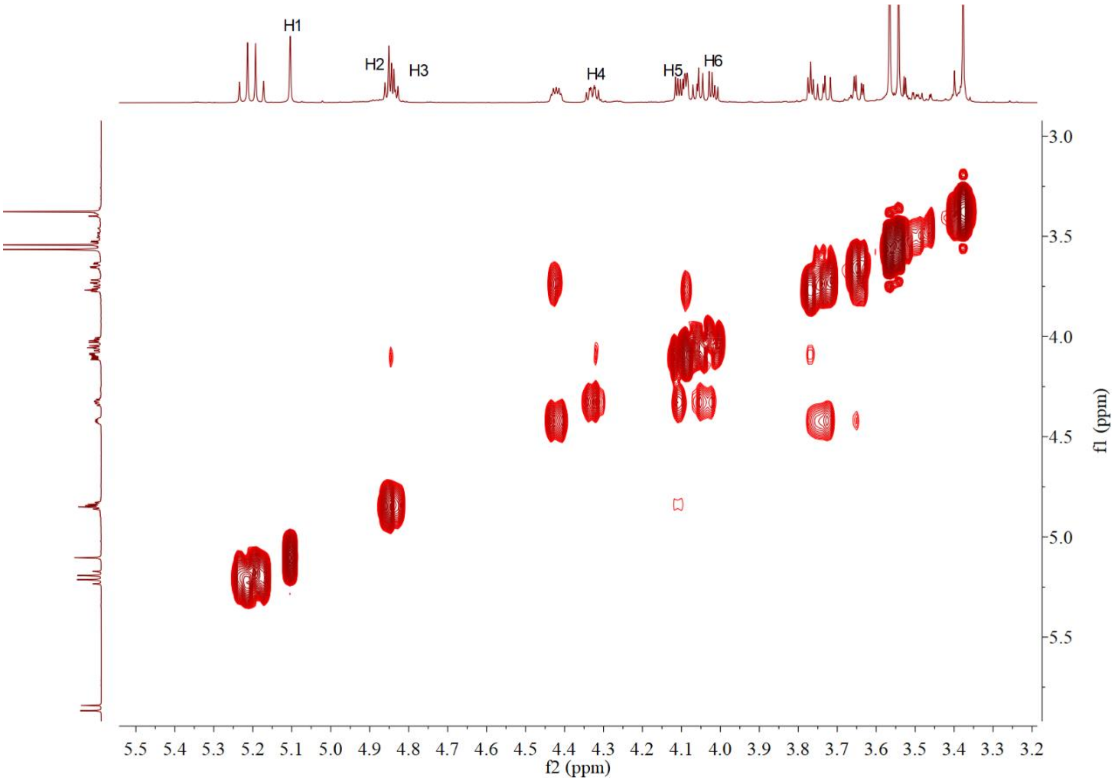

Supplementary Figure 71 COSY spectra of compound **4j**

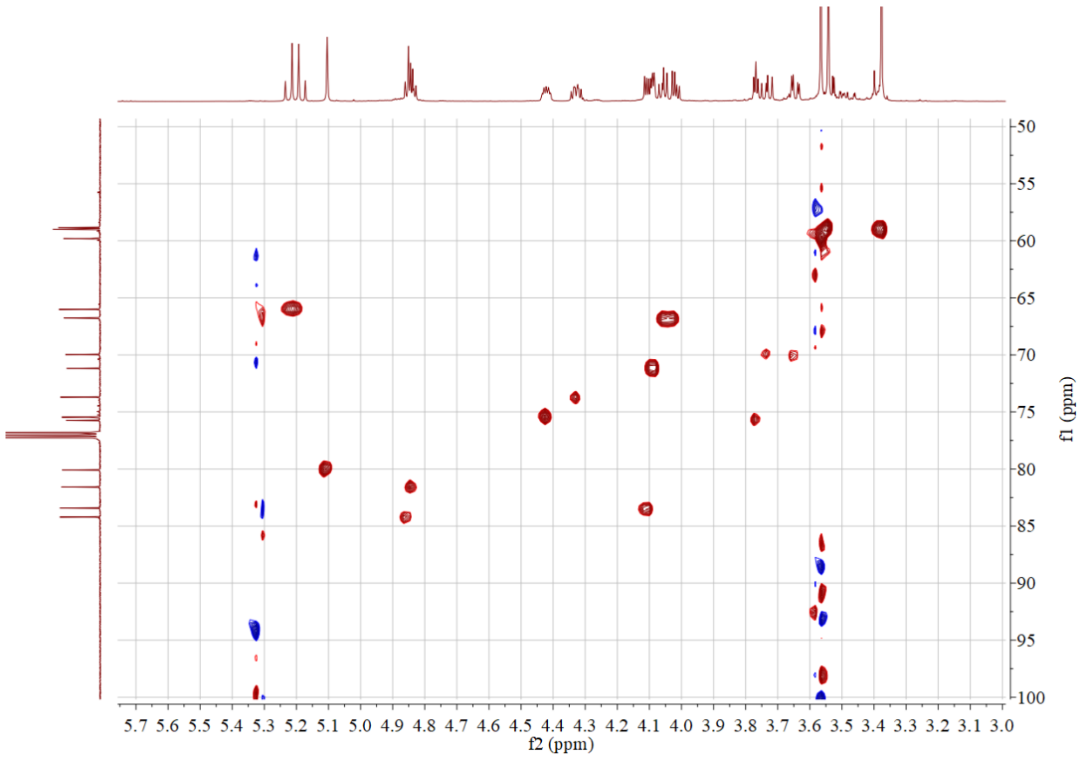

Supplementary Figure 72 HMBC spectra of compound **4j**

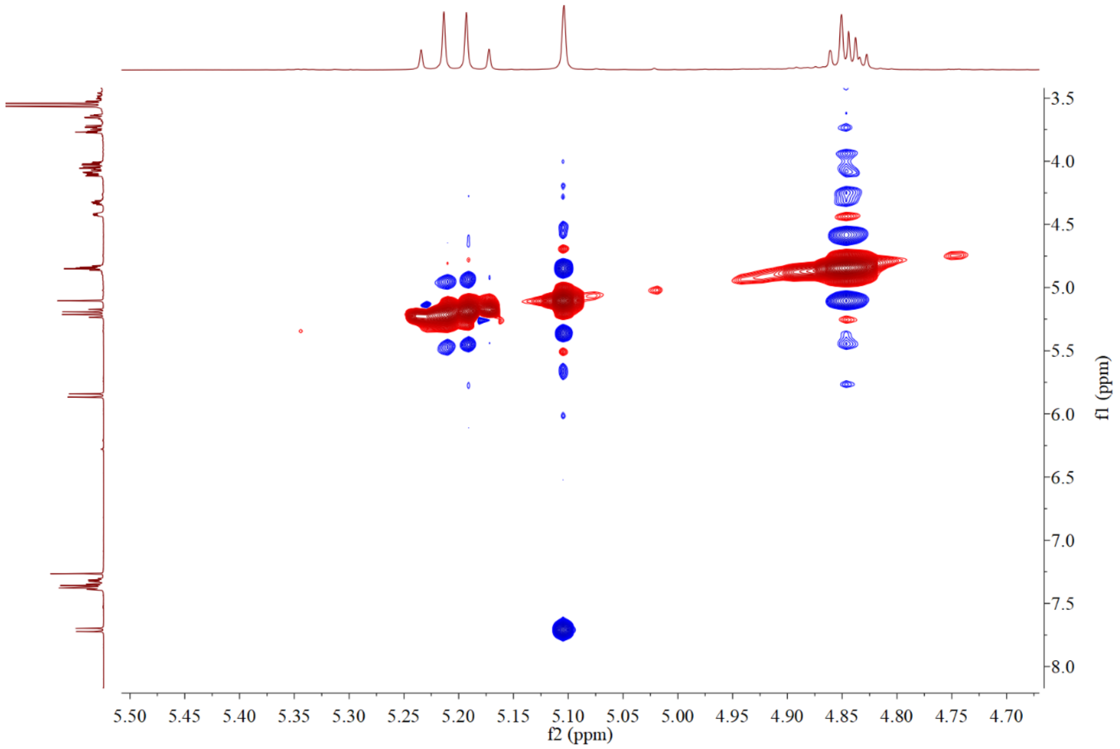

Supplementary Figure 73 NOESY spectra of compound **4j**

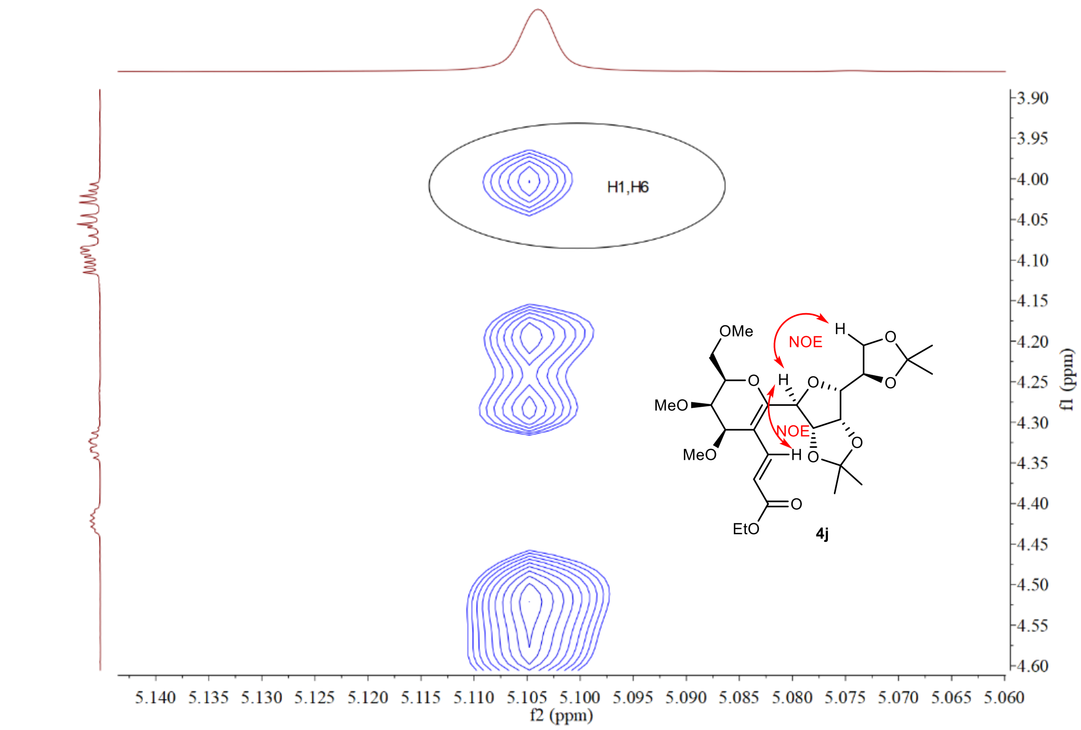

Supplementary Figure 74 NOESY spectra of compound **4j**

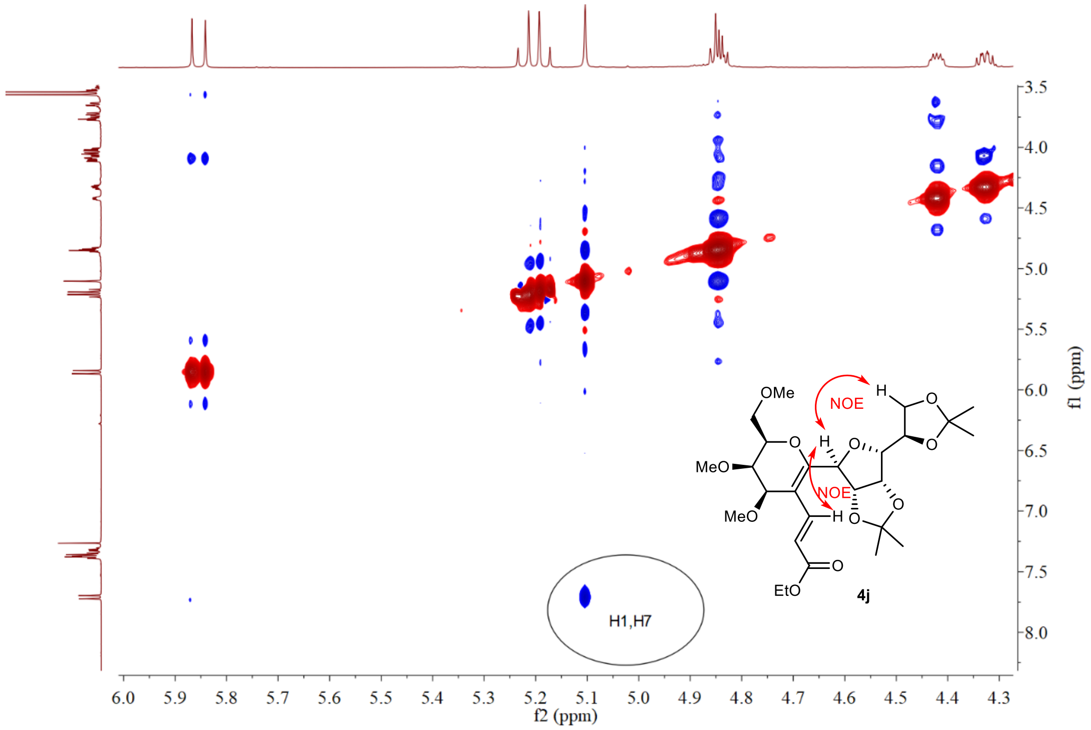

Supplementary Figure 75 NOESY spectra of compound **4j**

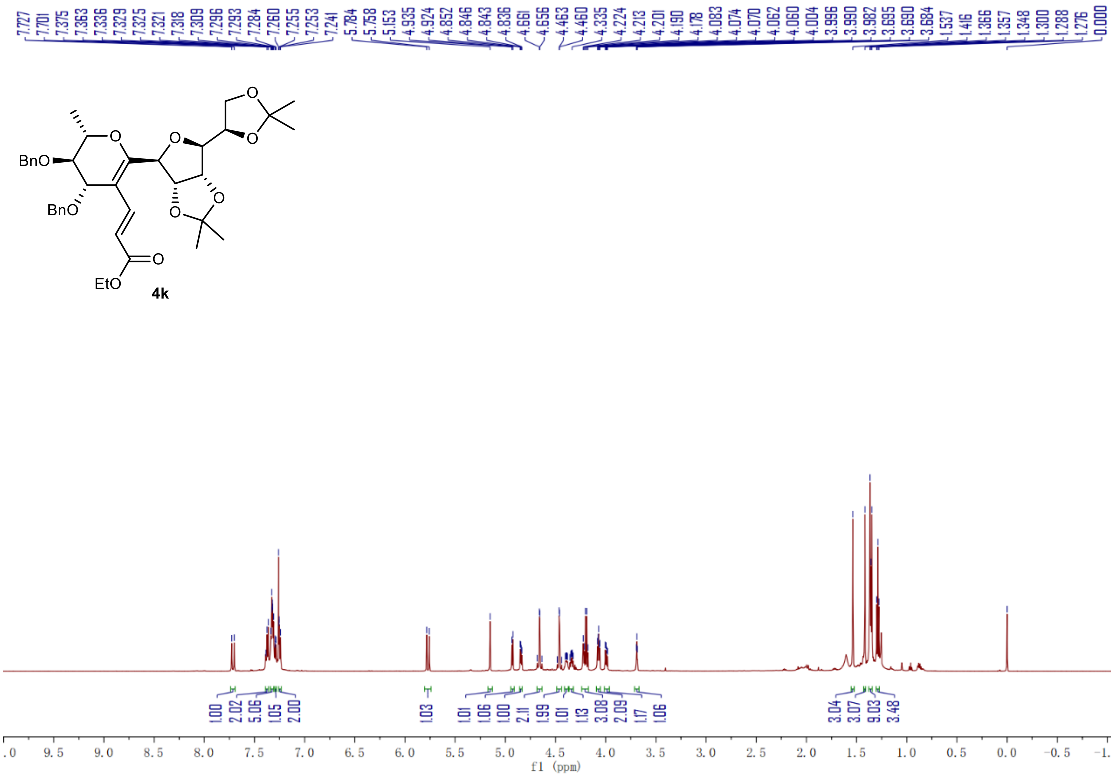

Supplementary Figure 76 ^{1}H NMR spectra of (600 MHz, $CDCl_3$) compound **4k**

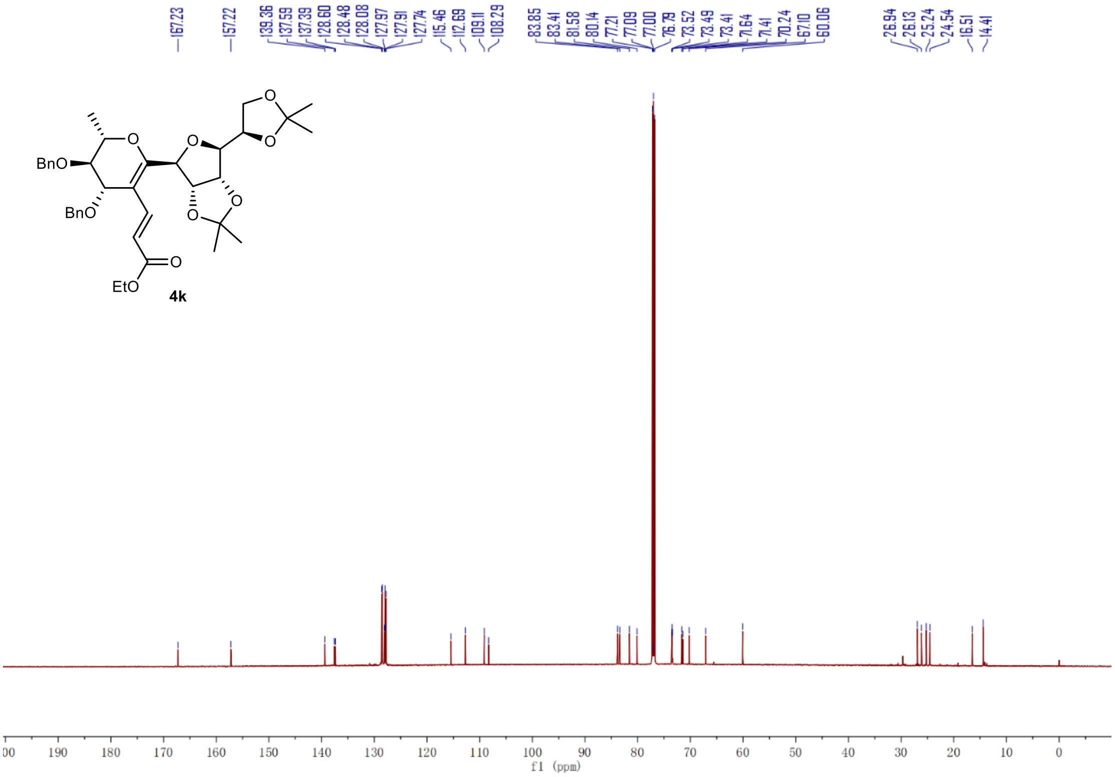

Supplementary Figure 77 ^{13}C spectra of (151 MHz, $CDCl_3$) compound **4k**

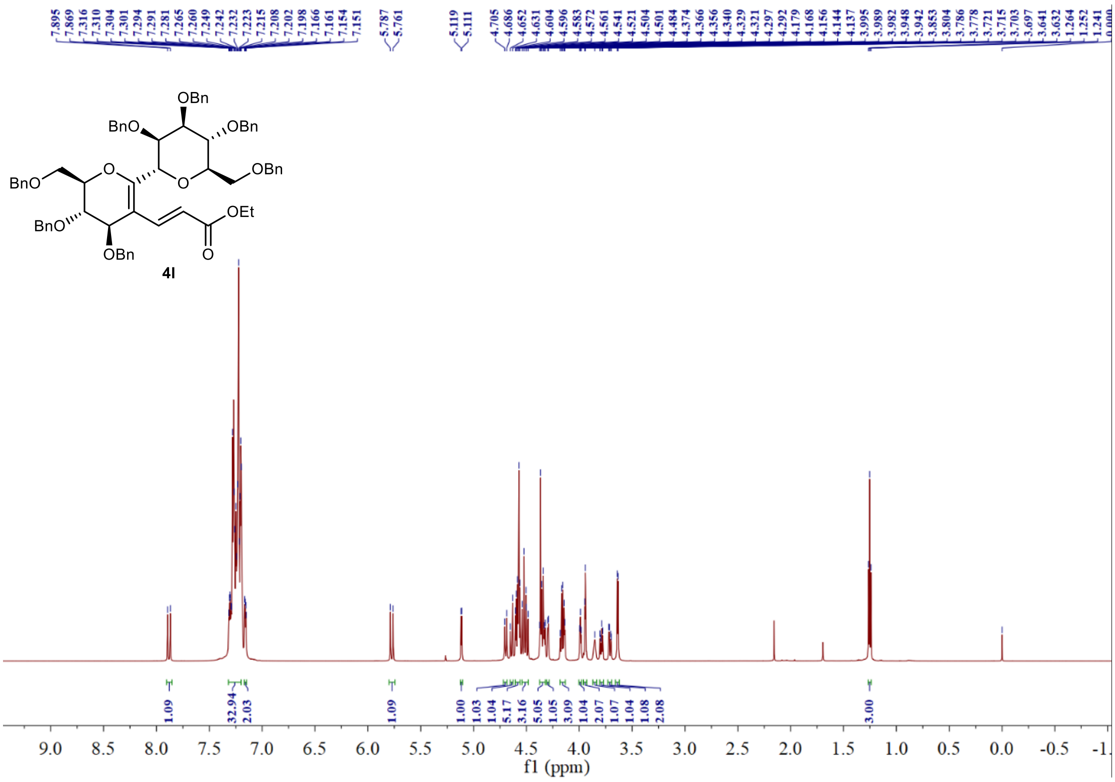

Supplementary Figure 78 ^{1}H NMR spectra of (600 MHz, $CDCl_3$) compound **4l**

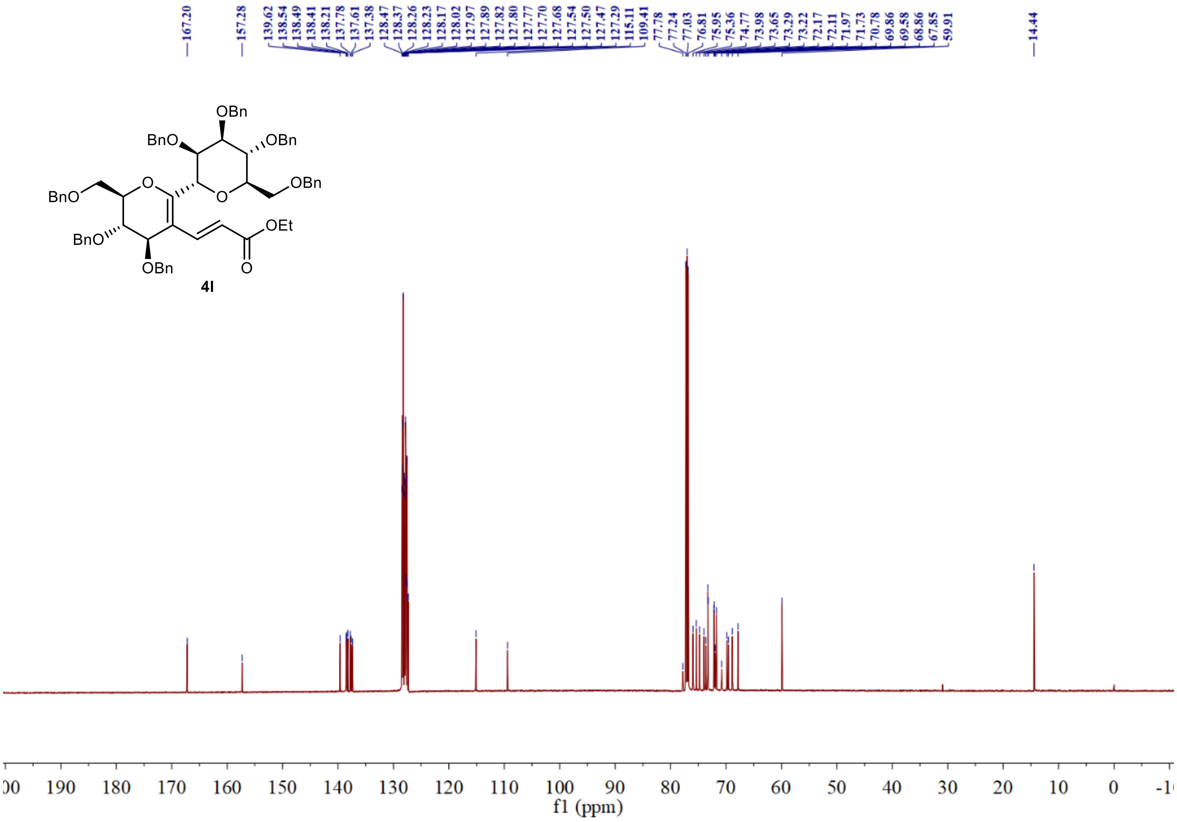

Supplementary Figure 79 ^{13}C spectra of (151 MHz, $CDCl_3$) compound **4l**

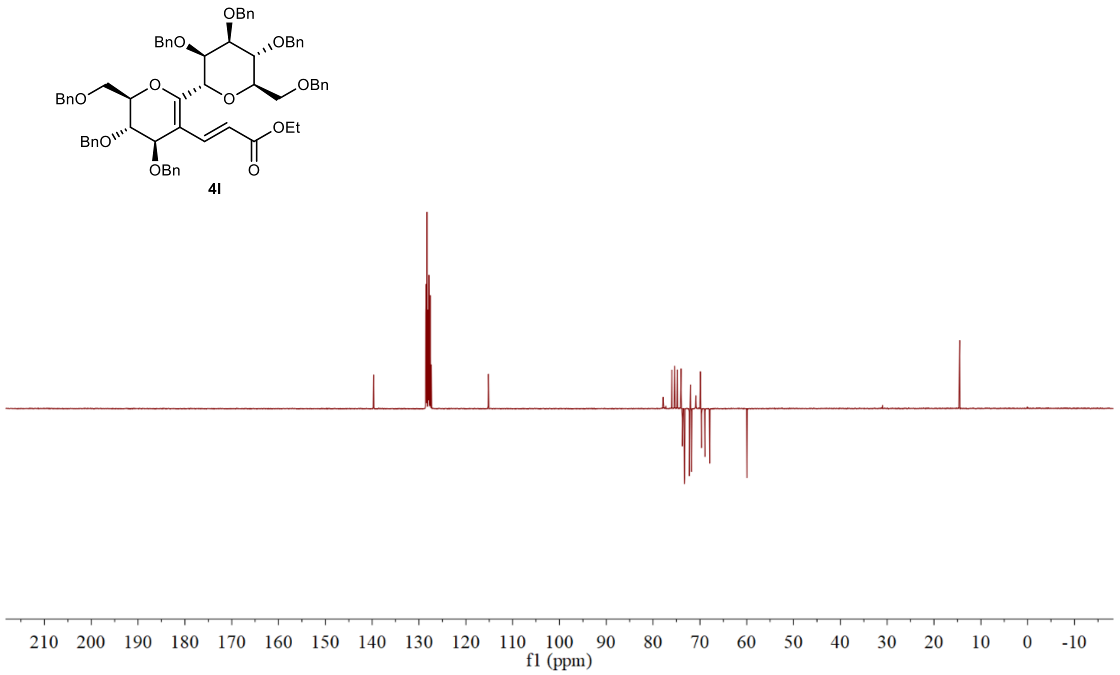

Supplementary Figure 80 DEPT spectra of compound **4l**

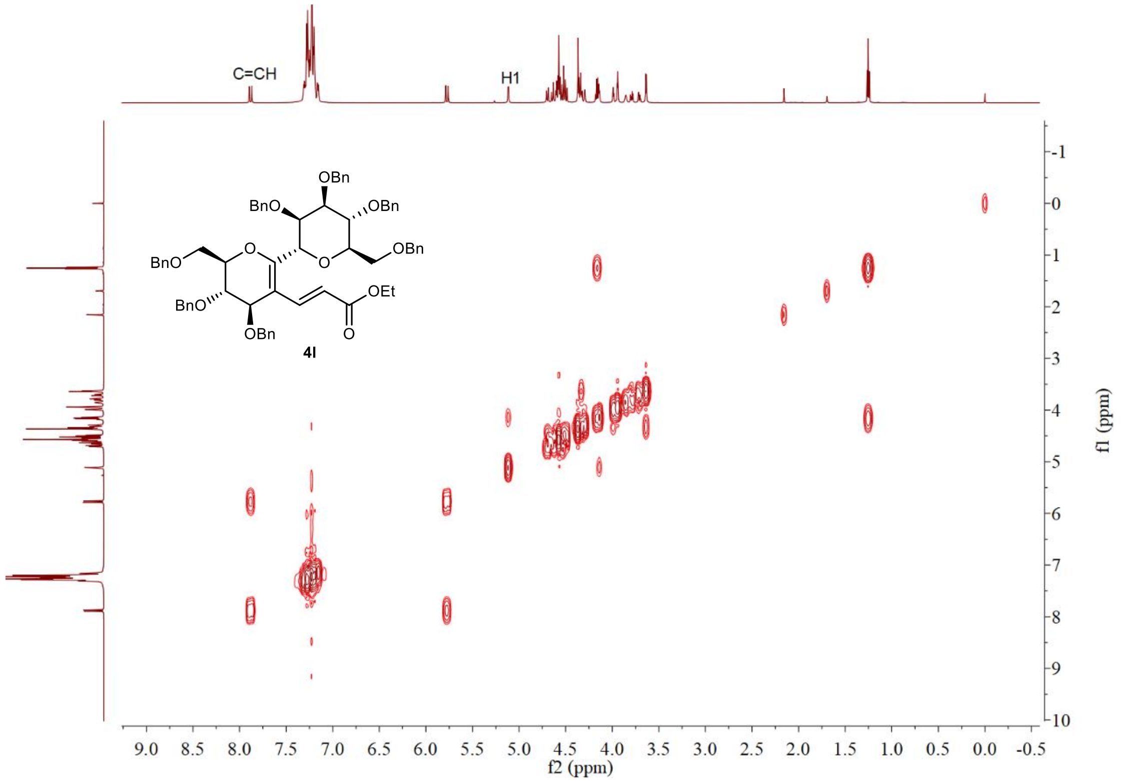

Supplementary Figure 81 COSY spectra of compound **4l**

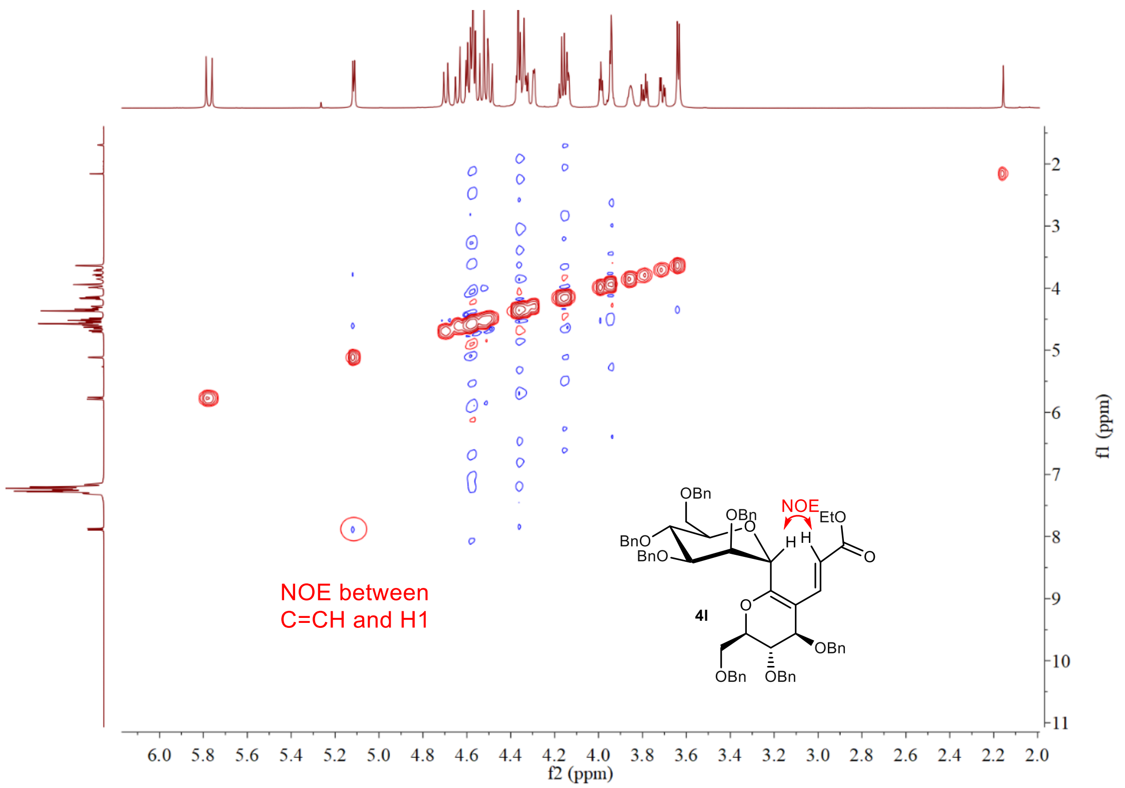

Supplementary Figure 82 NOESY spectra of compound **4l**

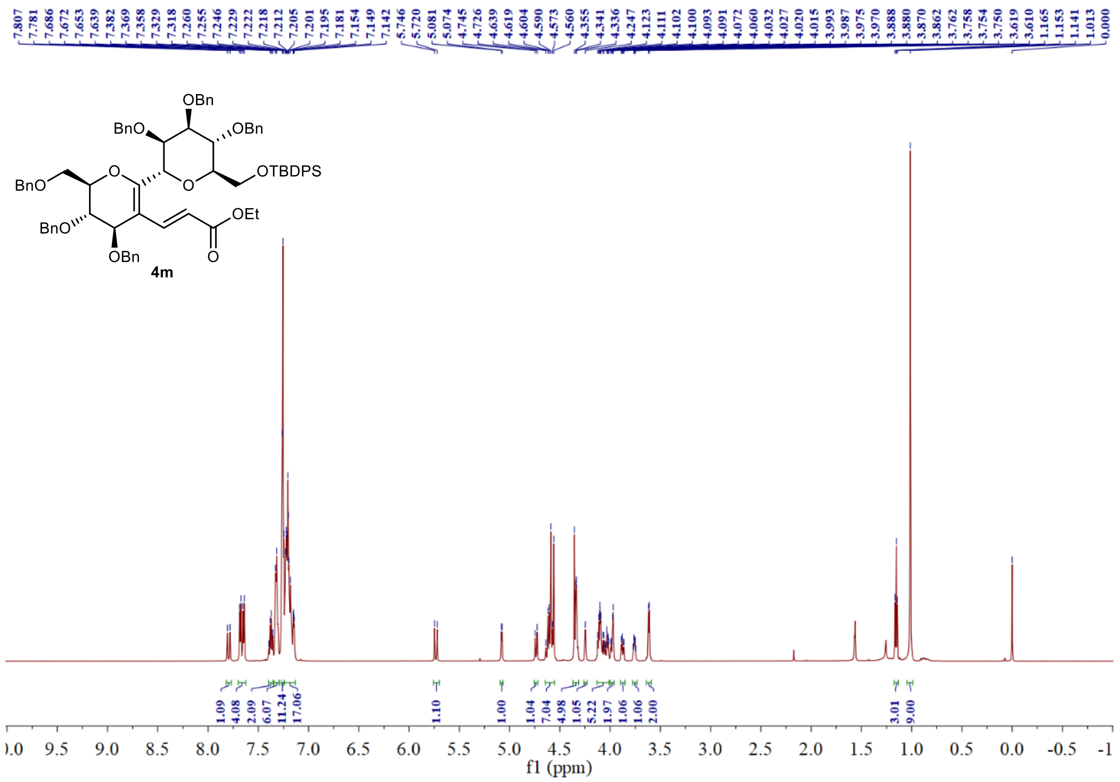

Supplementary Figure 83 ^{1}H NMR spectra of (600 MHz, $CDCl_3$) compound **4m**

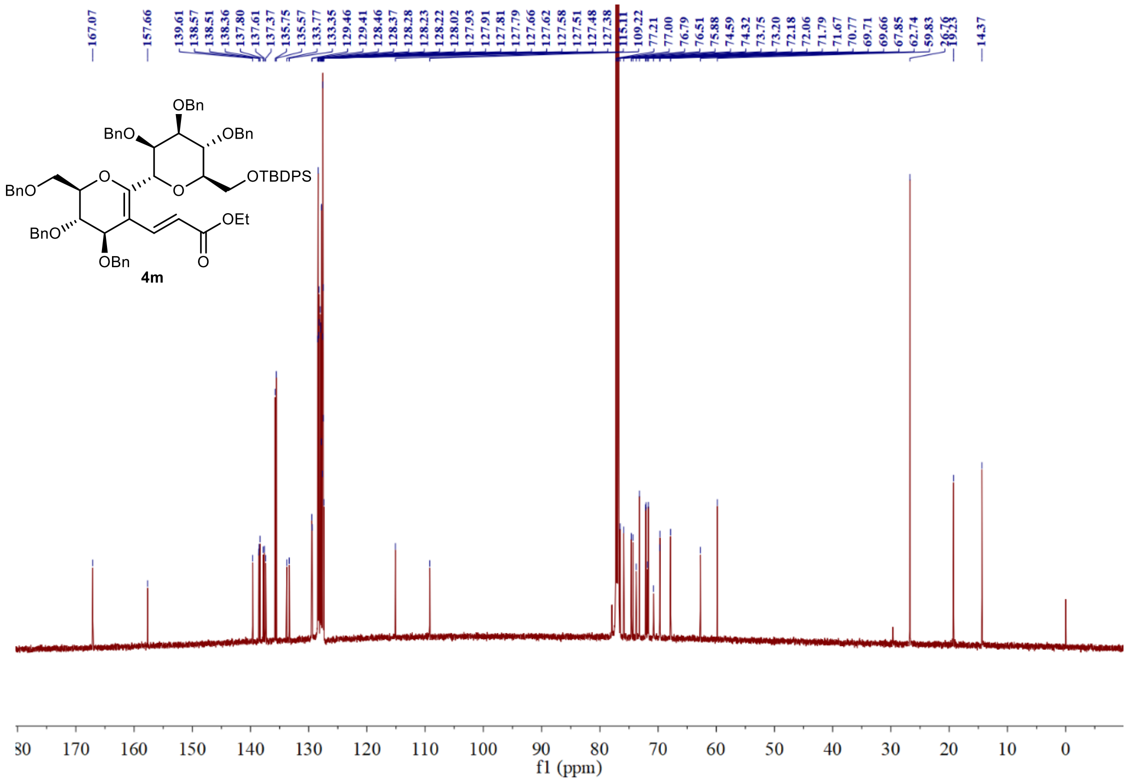

Supplementary Figure 84 ^{13}C spectra of (151 MHz, $CDCl_3$) compound **4m**

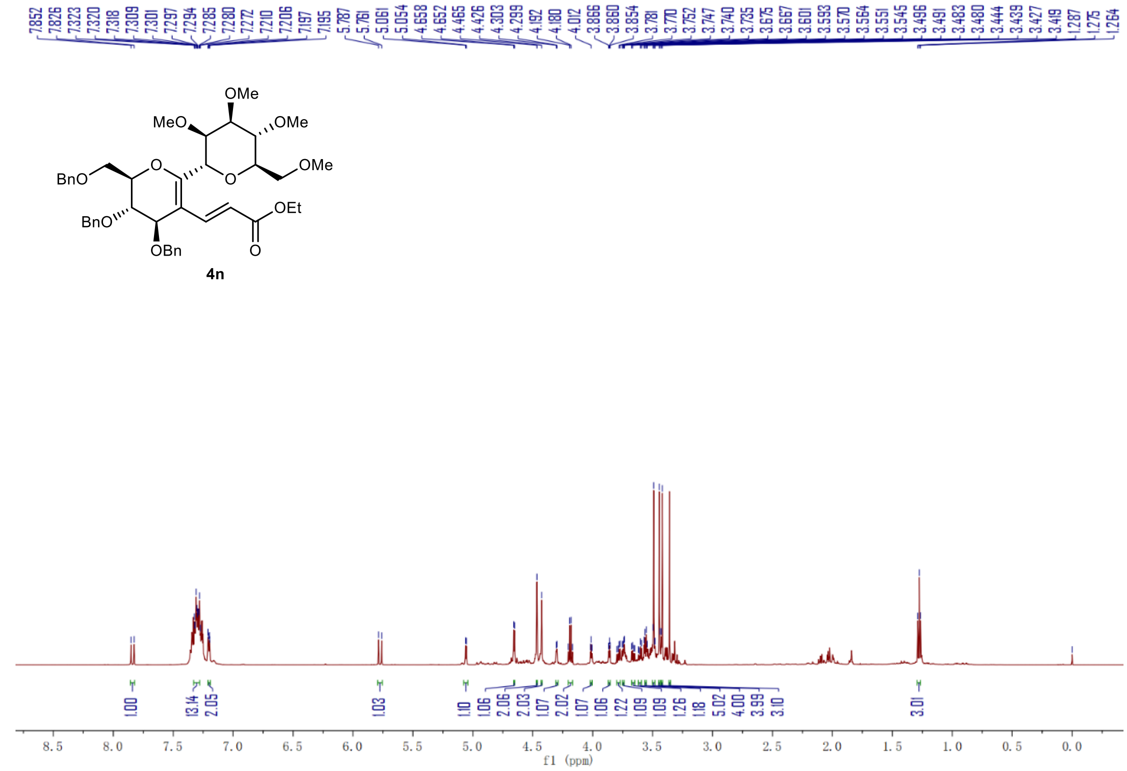

Supplementary Figure 85 ^{1}H NMR spectra of (600 MHz, $CDCl_3$) compound **4n**

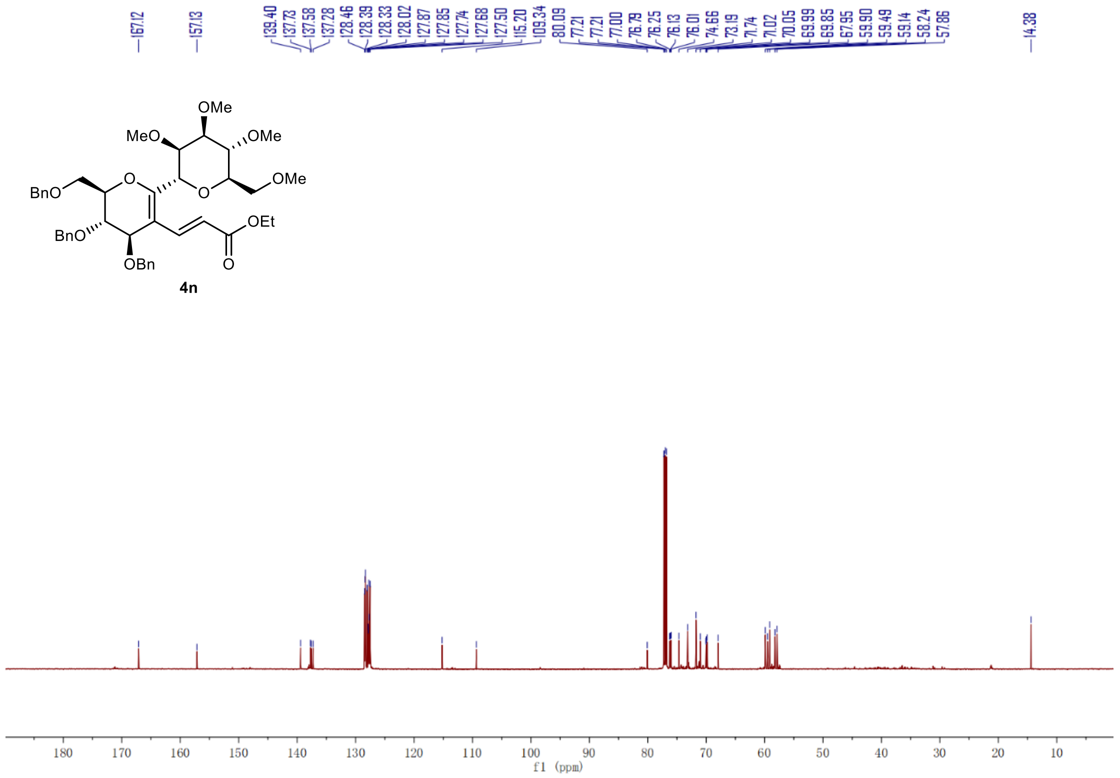

Supplementary Figure 86 ^{13}C spectra of (151 MHz, $CDCl_3$) compound **4n**

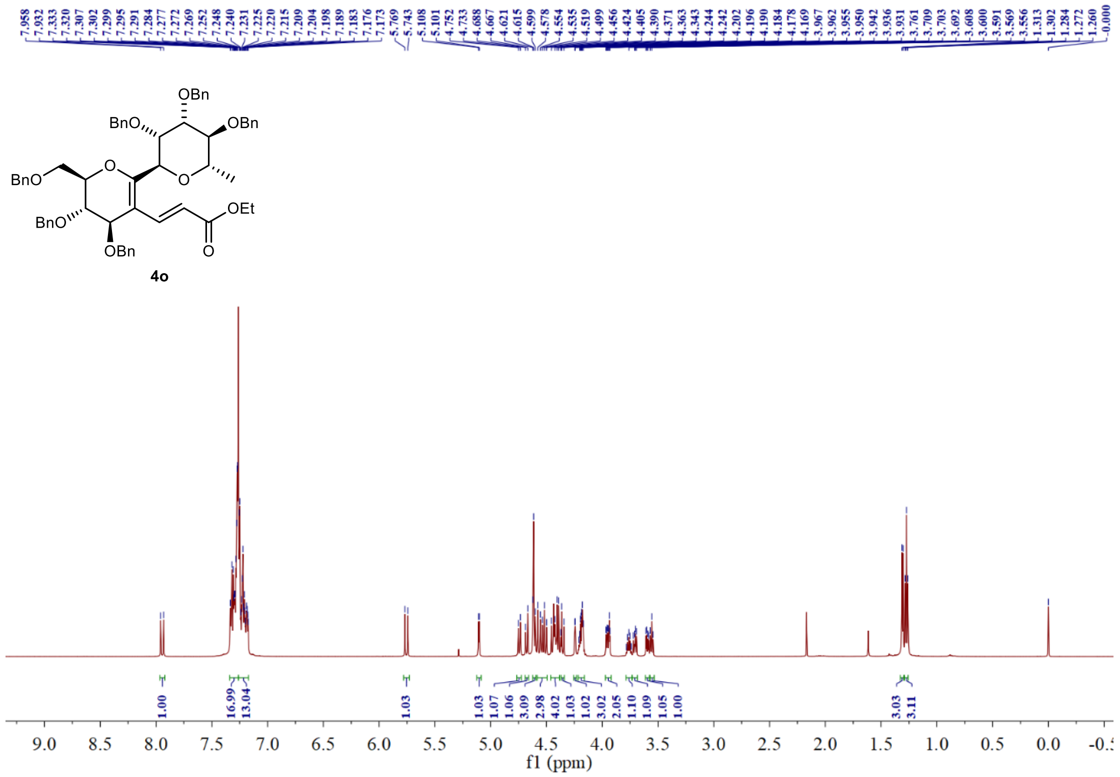

Supplementary Figure 87 ^{1}H NMR spectra of (600 MHz, $CDCl_3$) compound **4o**

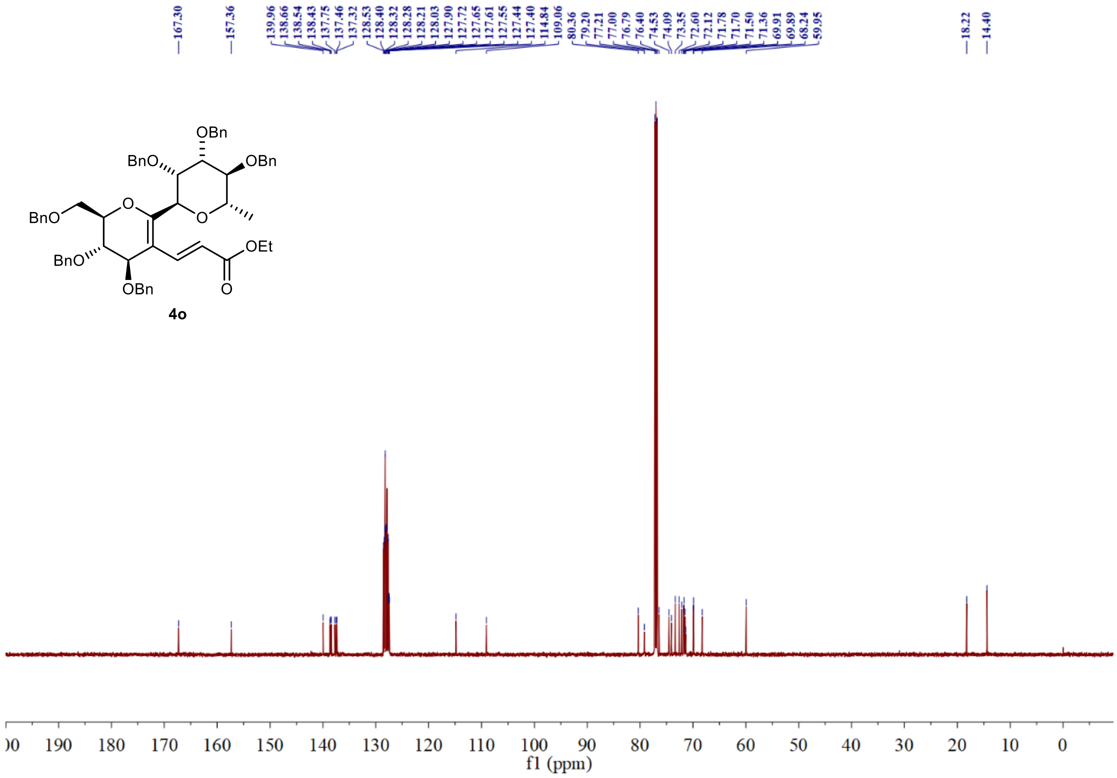

Supplementary Figure 88 ^{13}C spectra of (151 MHz, $CDCl_3$) compound **4o**

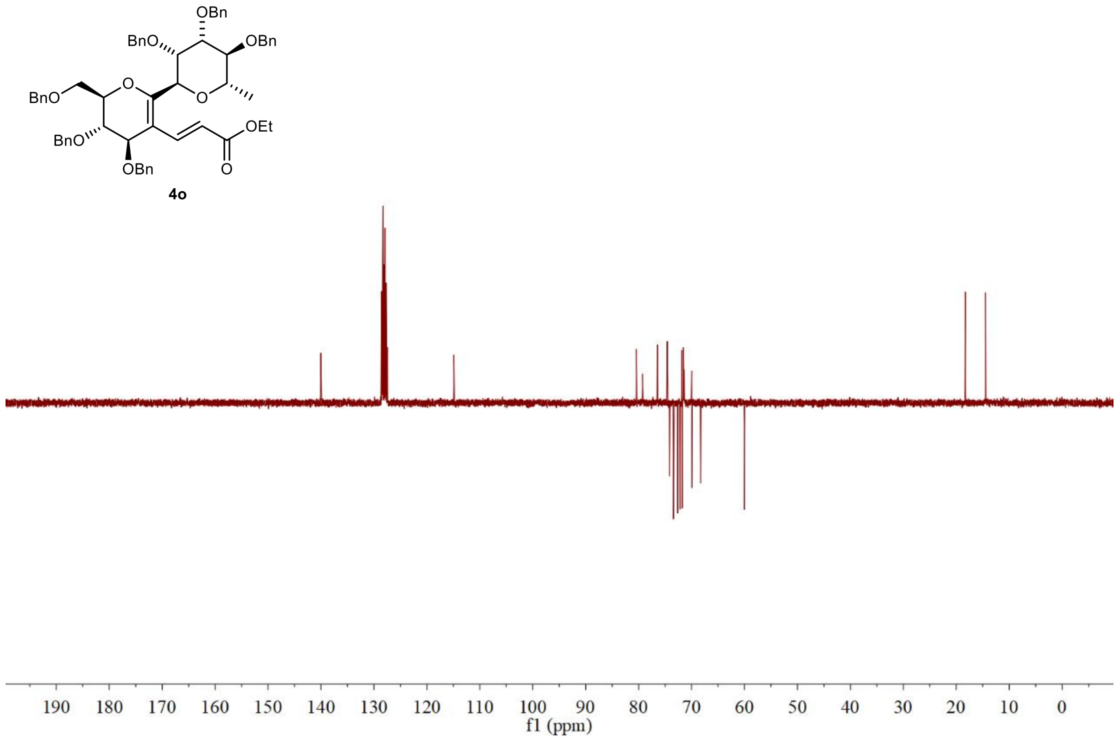

Supplementary Figure 89 DEPT spectra of compound **4o**

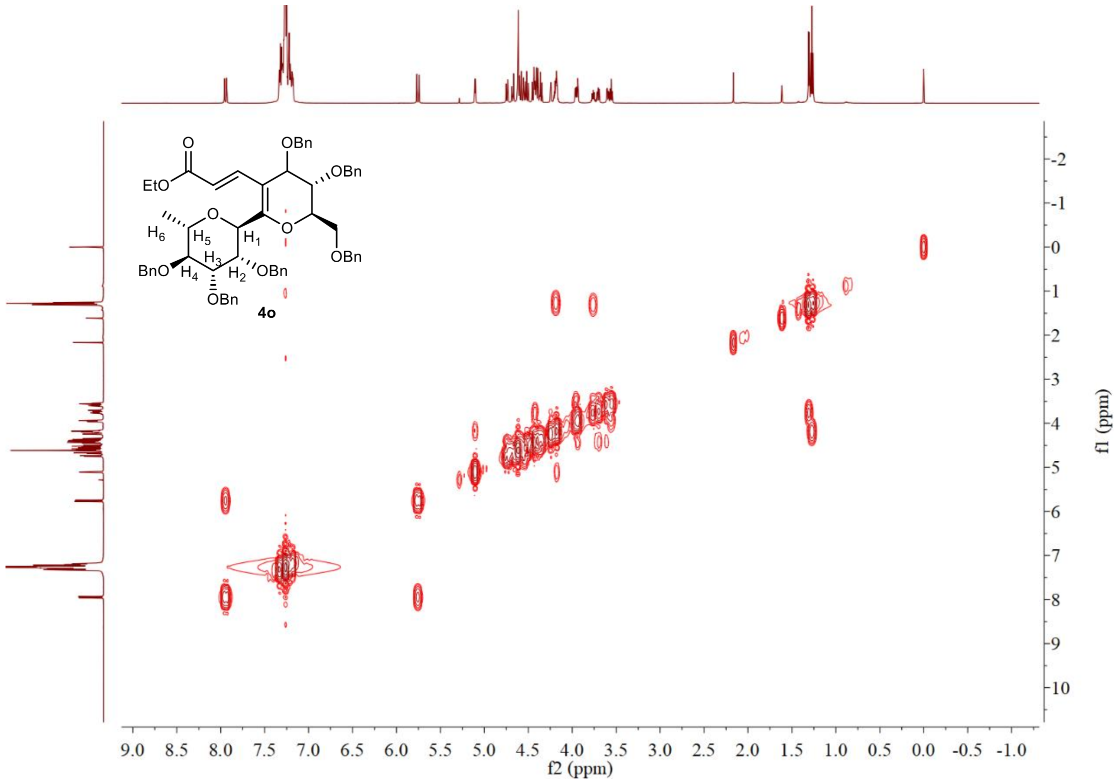

Supplementary Figure 90 COSY spectra of compound **4o**

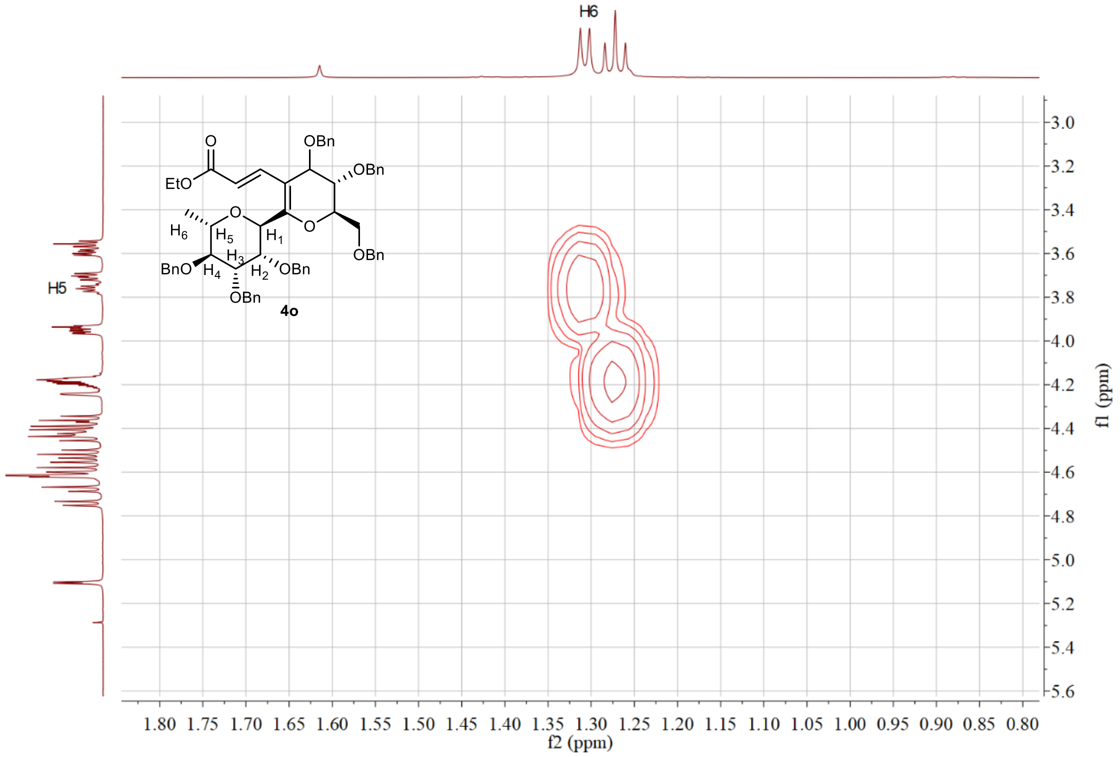

Supplementary Figure 91 COSY spectra of compound **4o**

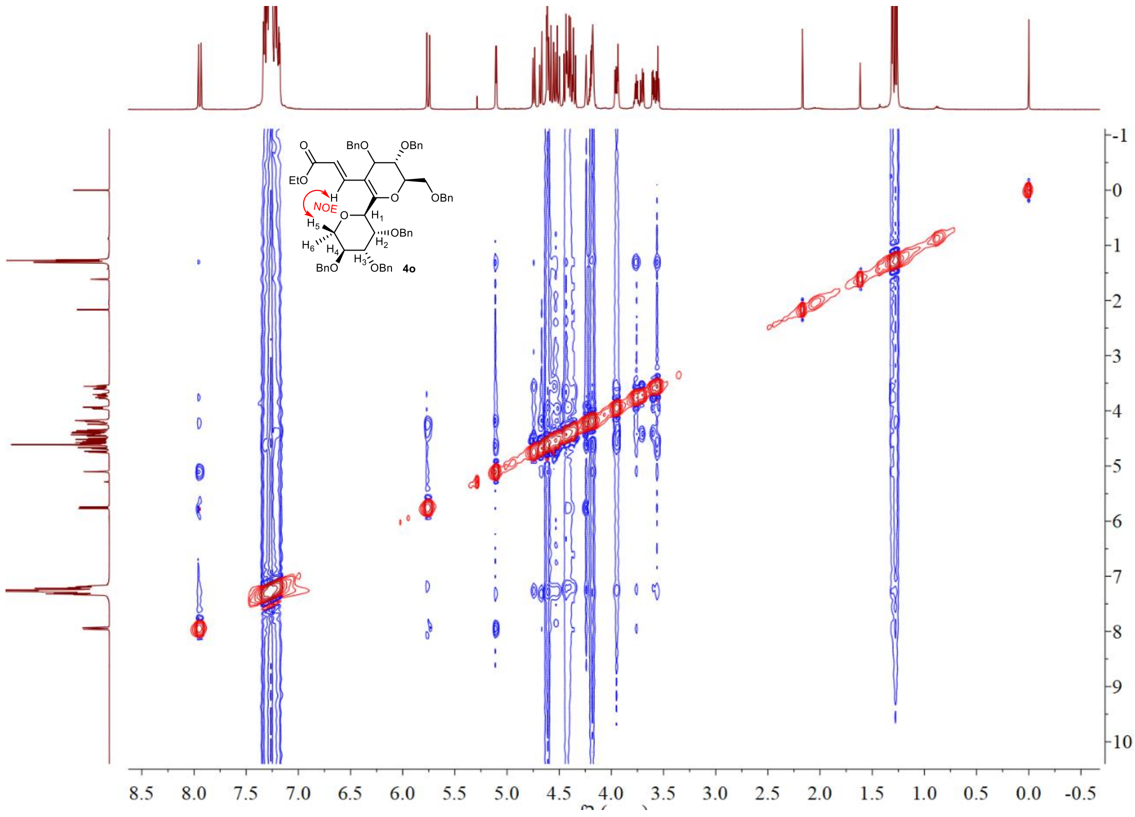

Supplementary Figure 92 NOESY spectra of compound **4o**

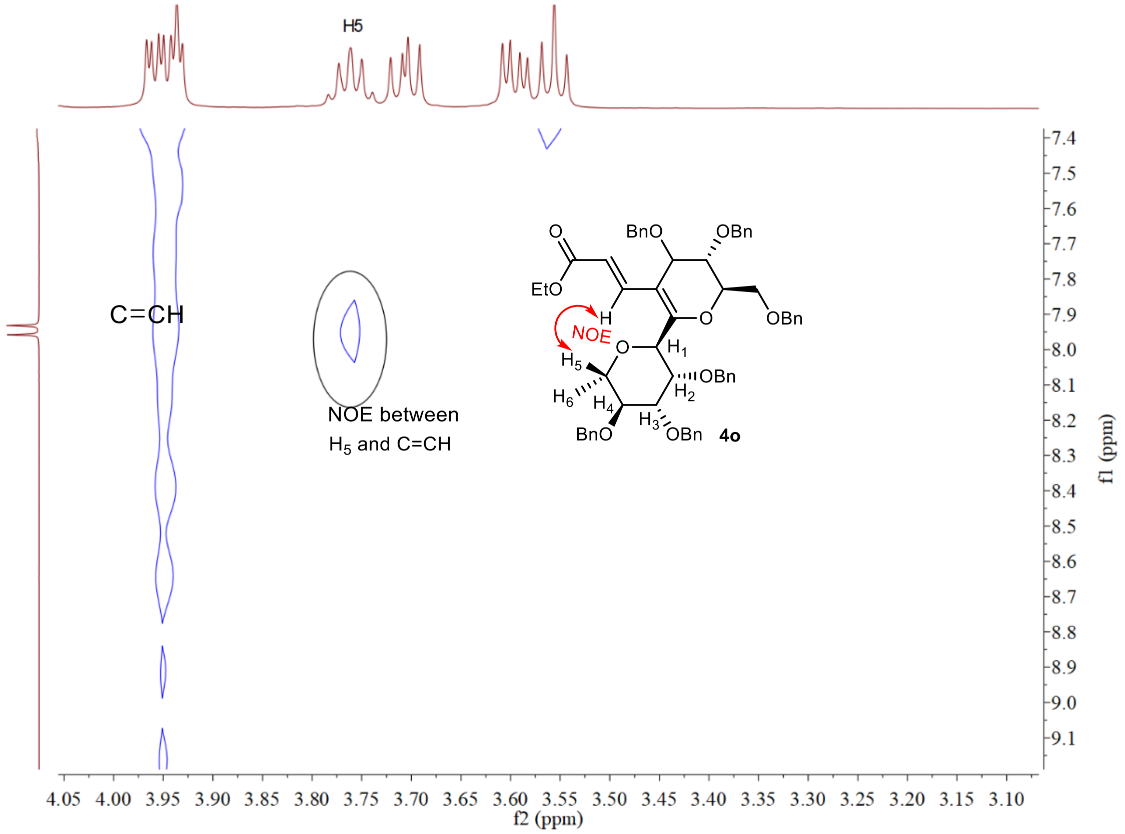

Supplementary Figure 93 NOESY spectra of compound **4o**

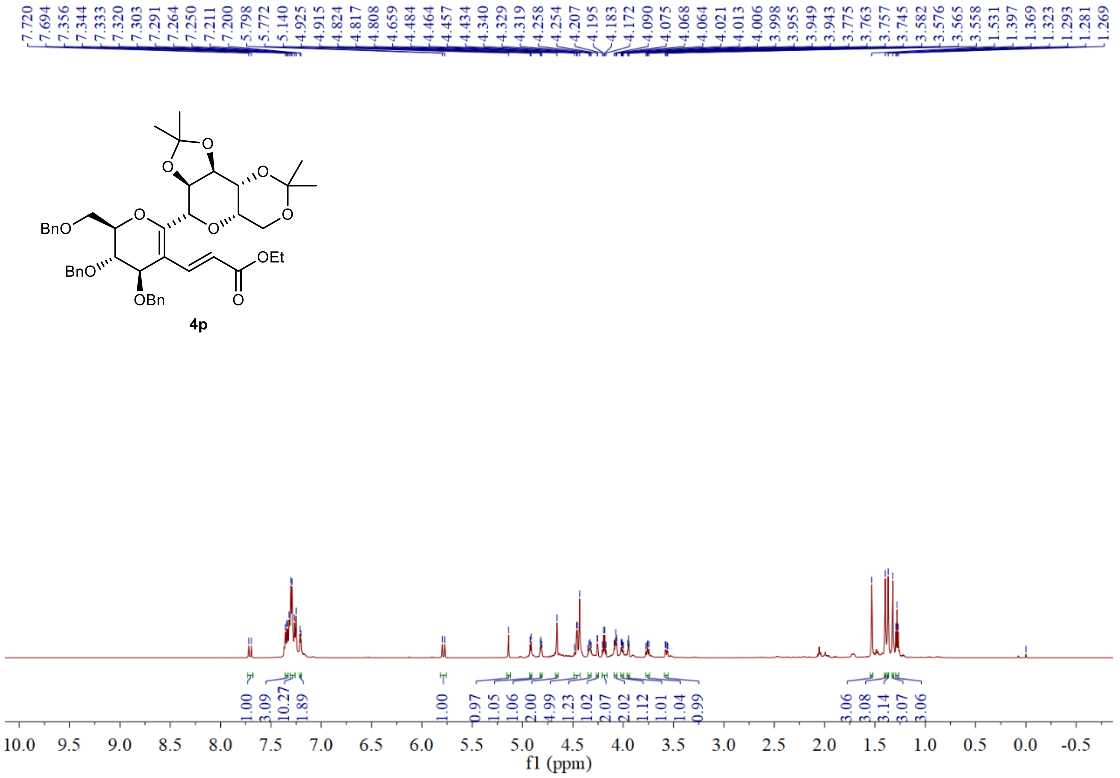

Supplementary Figure 94 ^{1}H NMR spectra of (600 MHz, $CDCl_3$) compound **4p**

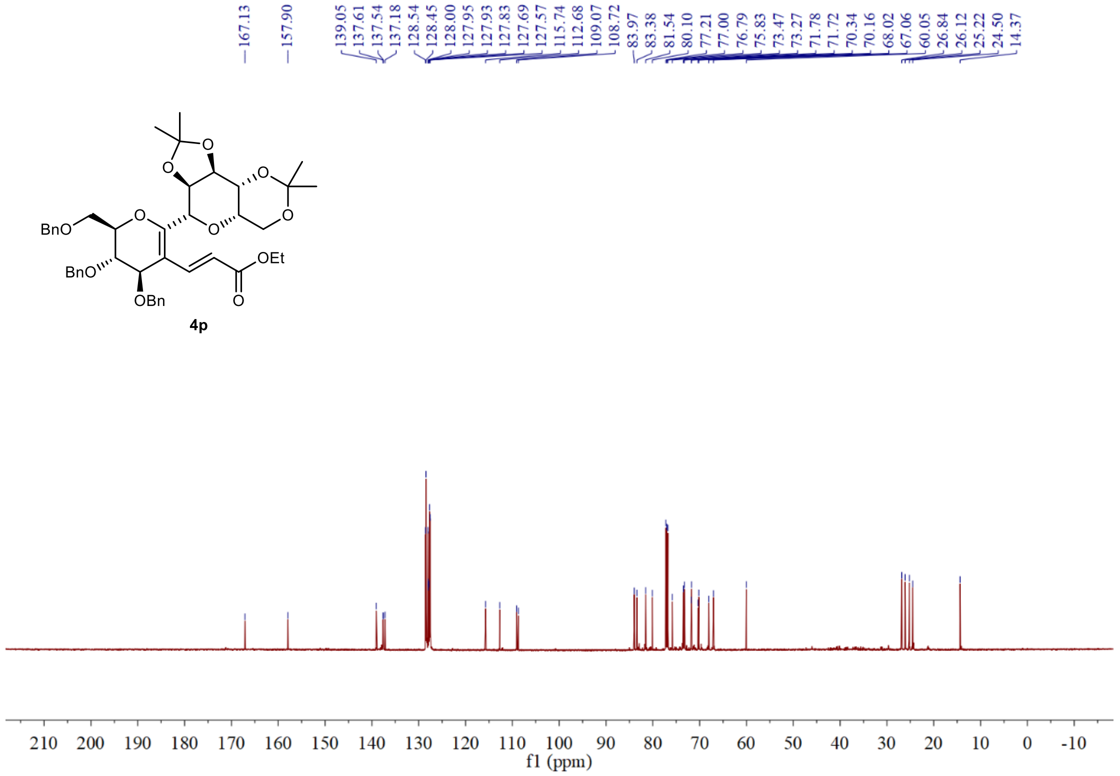

Supplementary Figure 95 ^{13}C spectra of (151 MHz, $CDCl_3$) compound **4p**

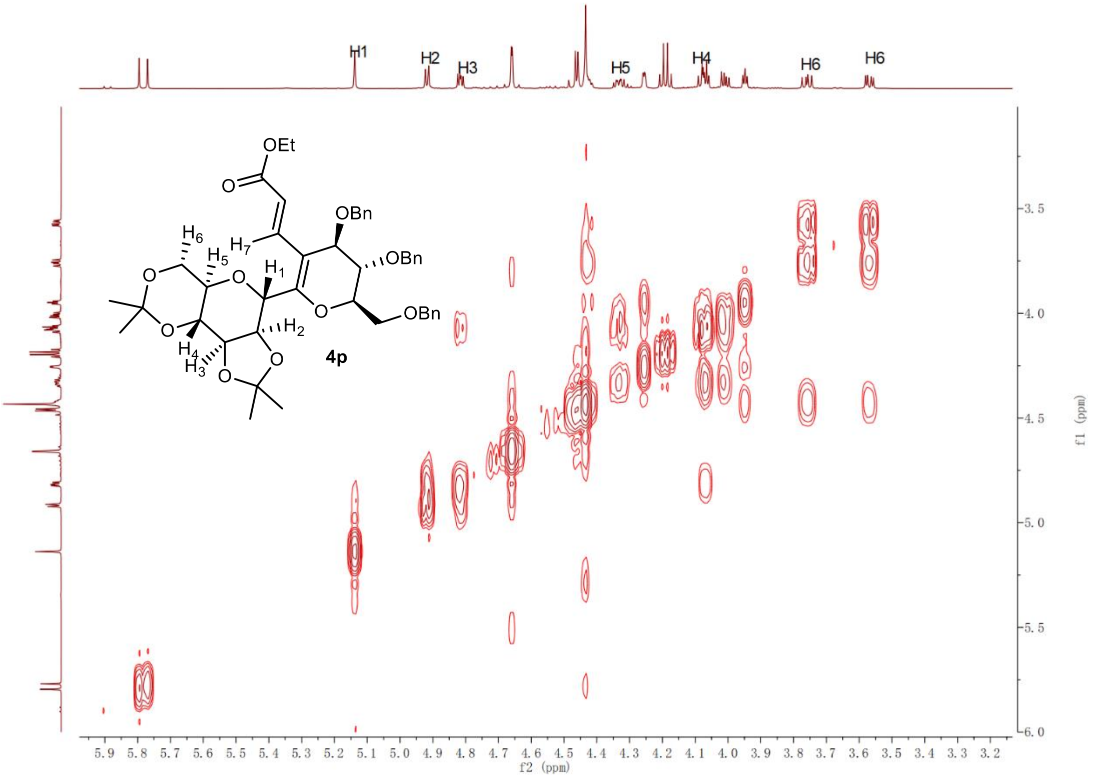

Supplementary Figure 96 COSY spectra of compound **4p**

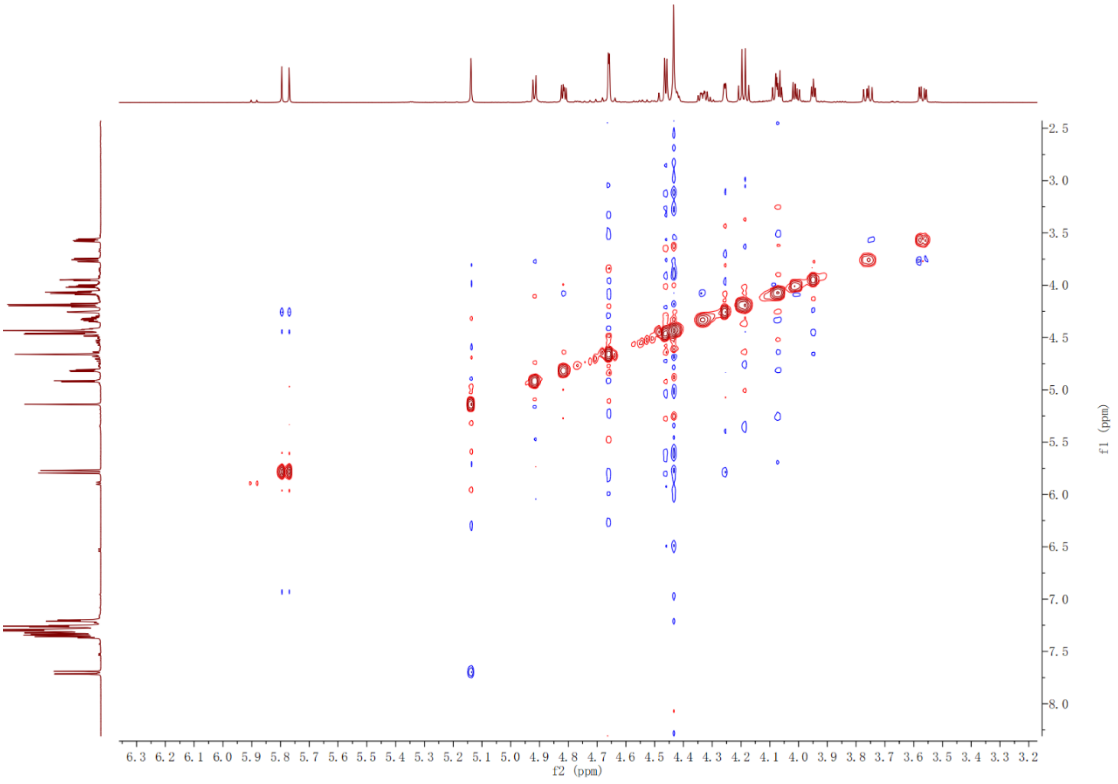

Supplementary Figure 97 NOESY spectra of compound **4p**

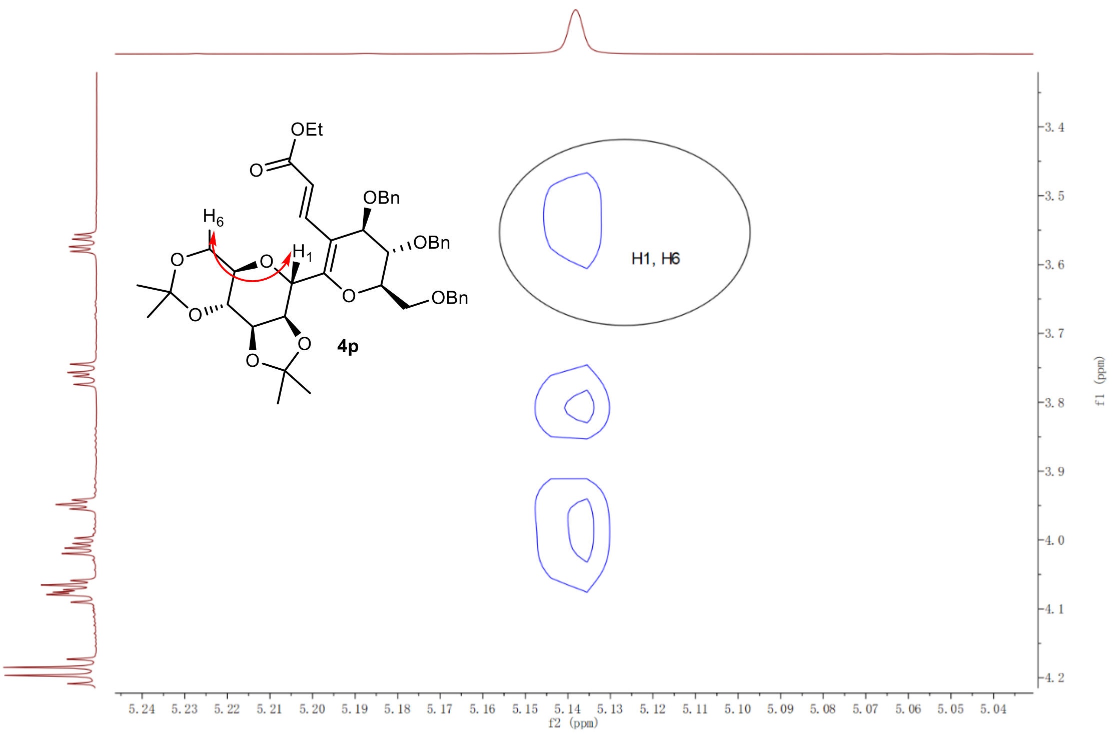

Supplementary Figure 98 NOESY spectra of compound **4p**

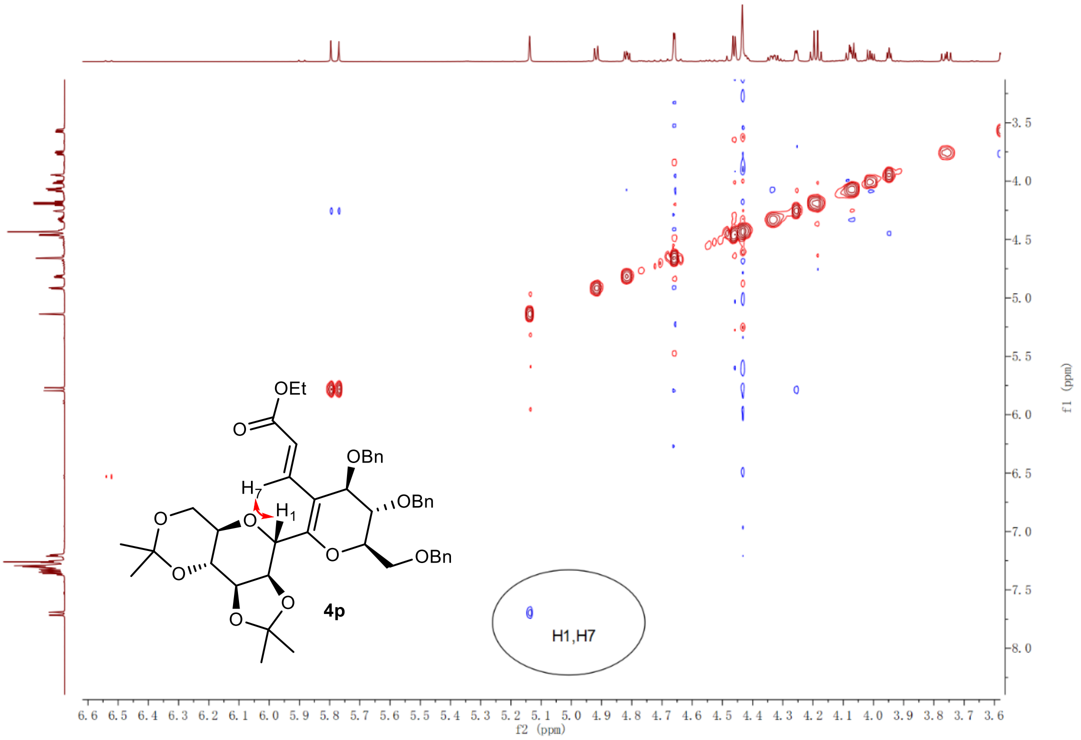

Supplementary Figure 99 NOESY spectra of compound **4p**

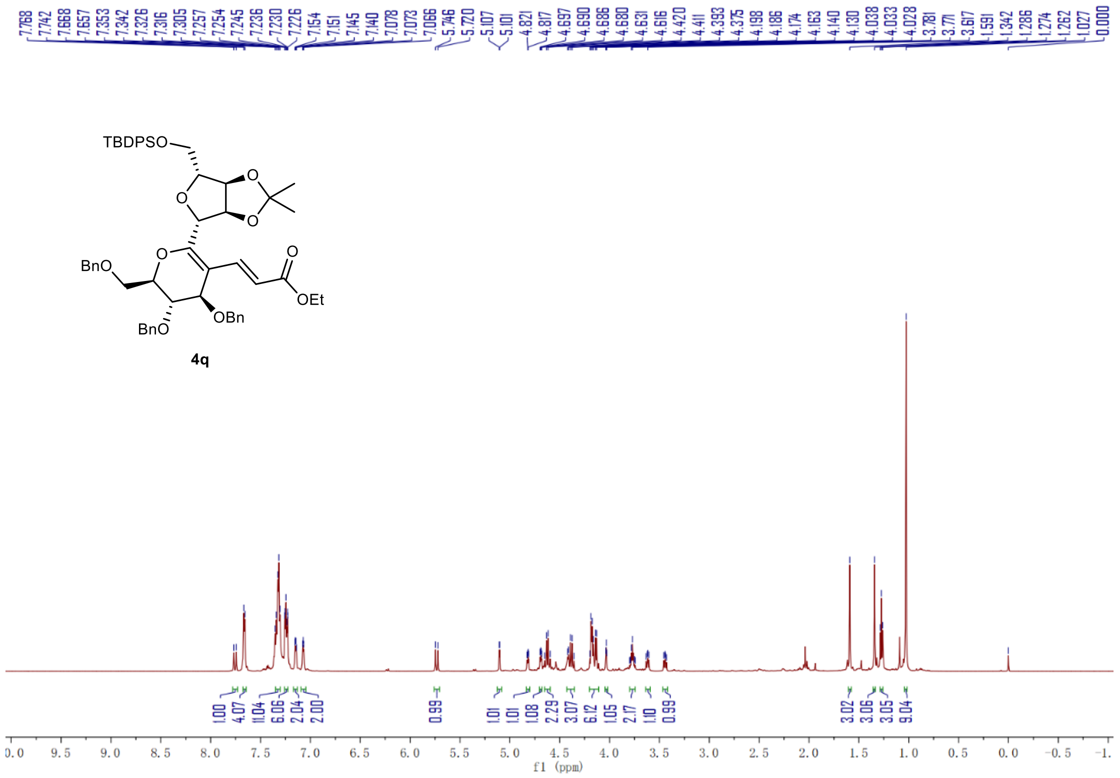

Supplementary Figure 100 ^{1}H NMR spectra of (600 MHz, $CDCl_3$) compound **4q**

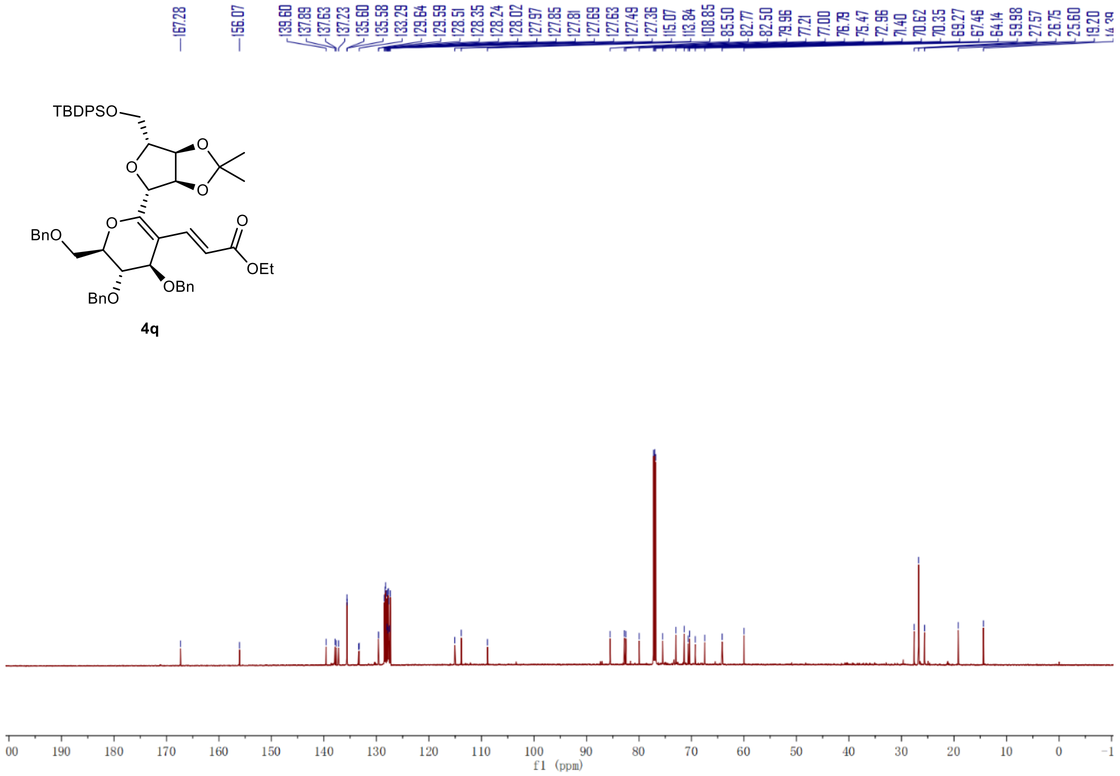

Supplementary Figure 101 ^{13}C spectra of (151 MHz, $CDCl_3$) compound **4q**

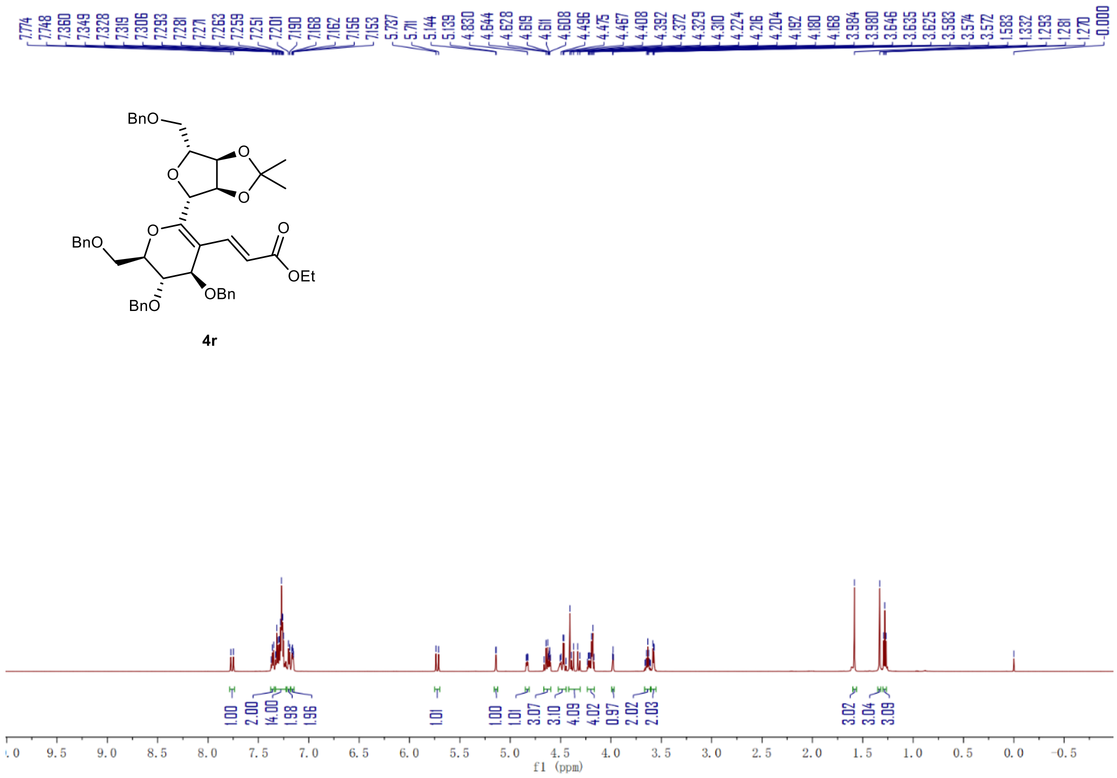

Supplementary Figure 102 ^{1}H NMR spectra of (600 MHz, $CDCl_3$) compound **4r**

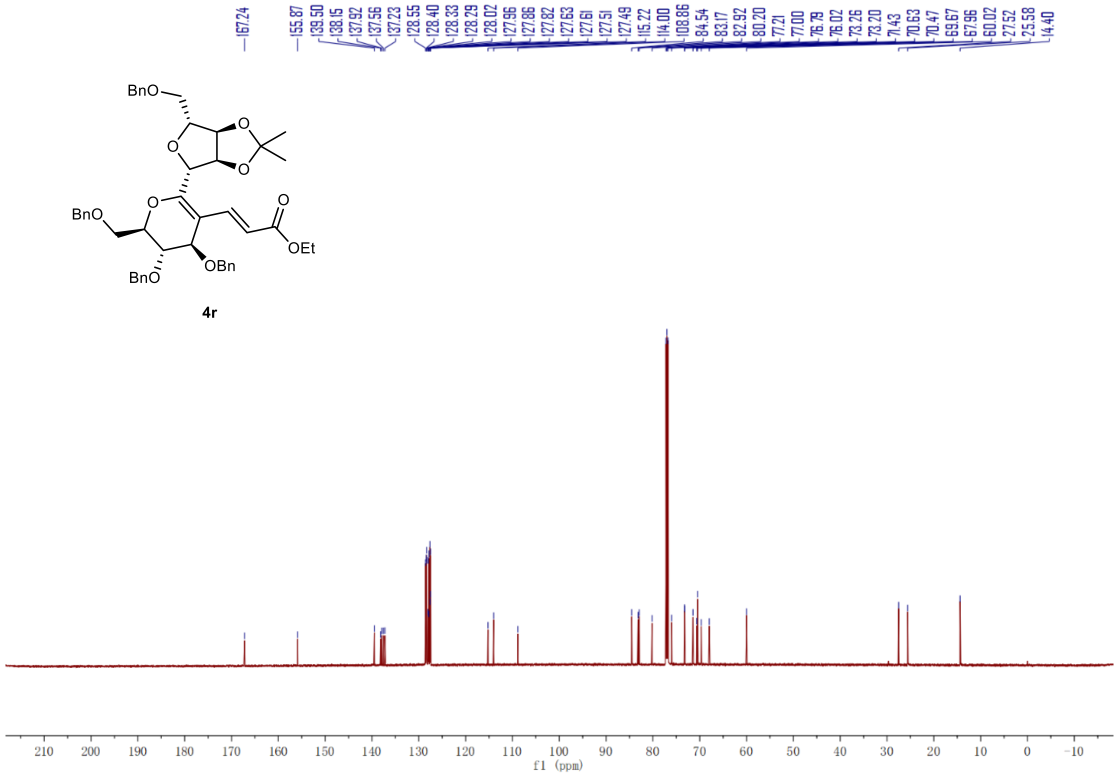

Supplementary Figure 103 ^{13}C spectra of (151 MHz, $CDCl_3$) compound **4r**

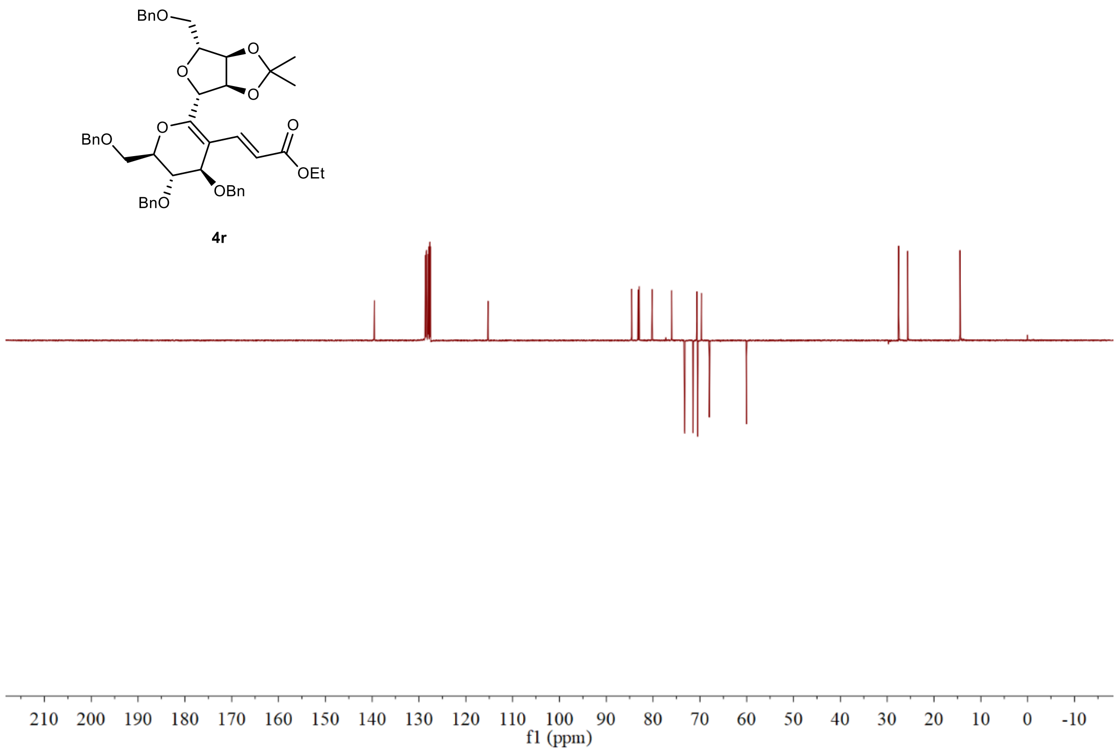

Supplementary Figure 104 DEPT spectra of compound **4r**

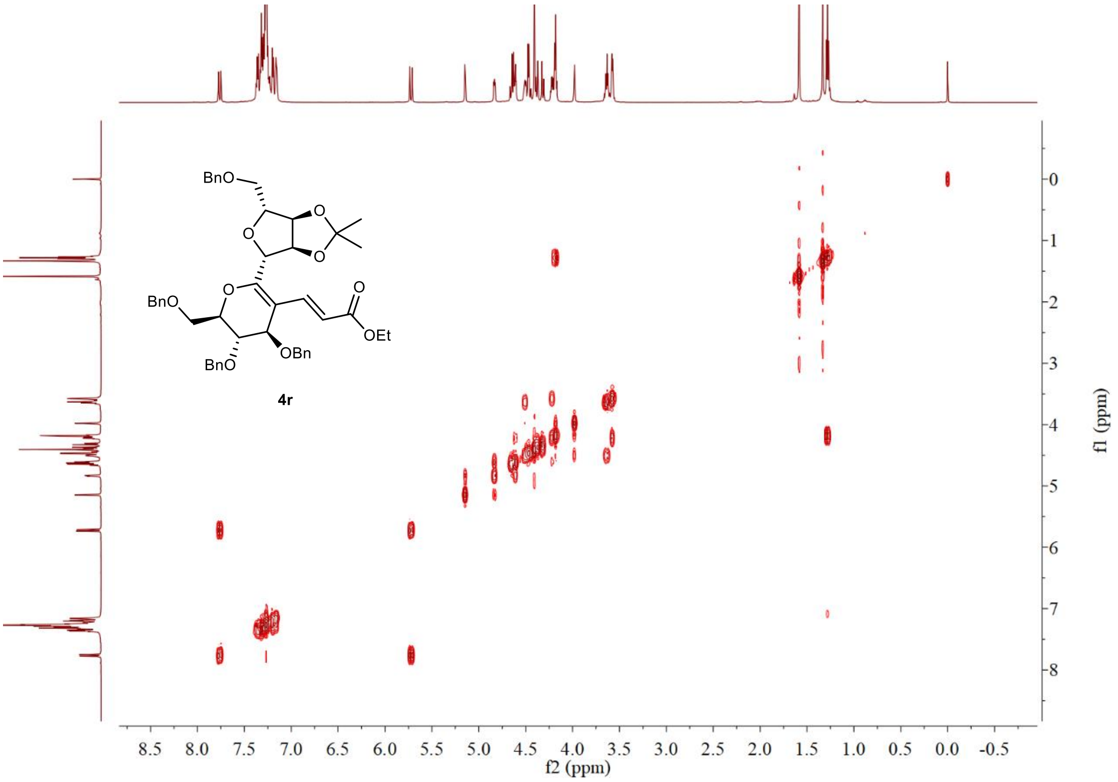

Supplementary Figure 105 COSY spectra of compound **4r**

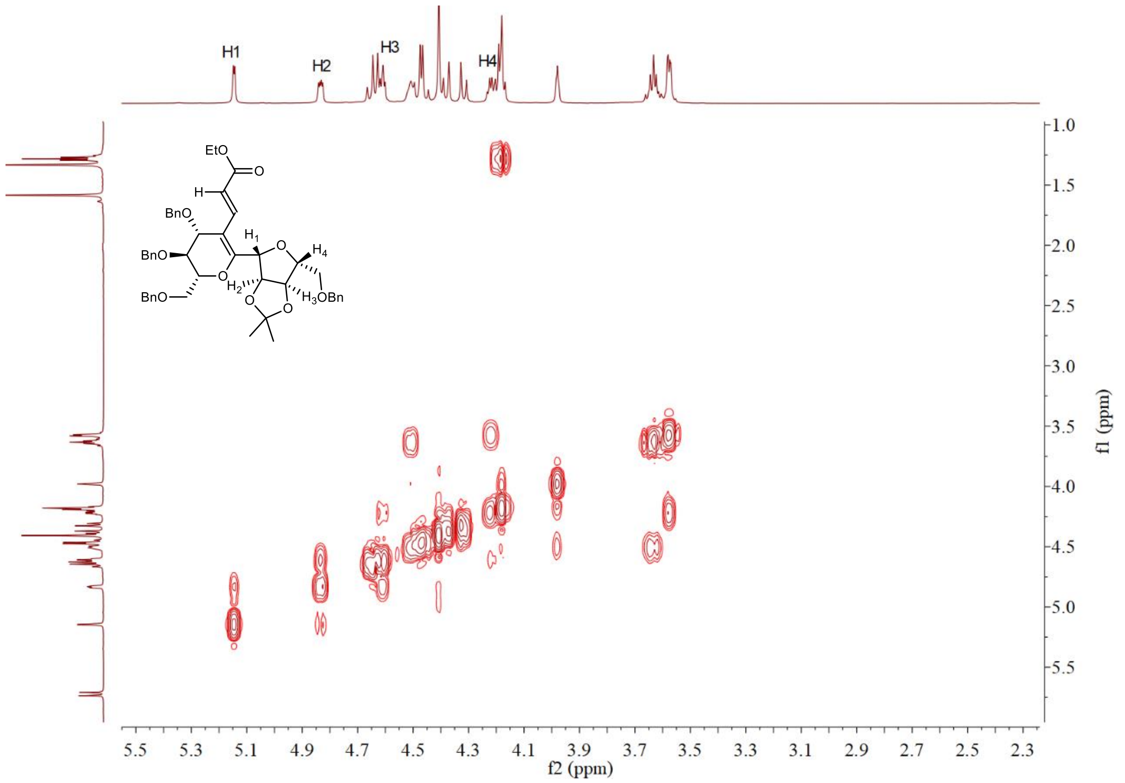

Supplementary Figure 106 COSY spectra of compound **4r**

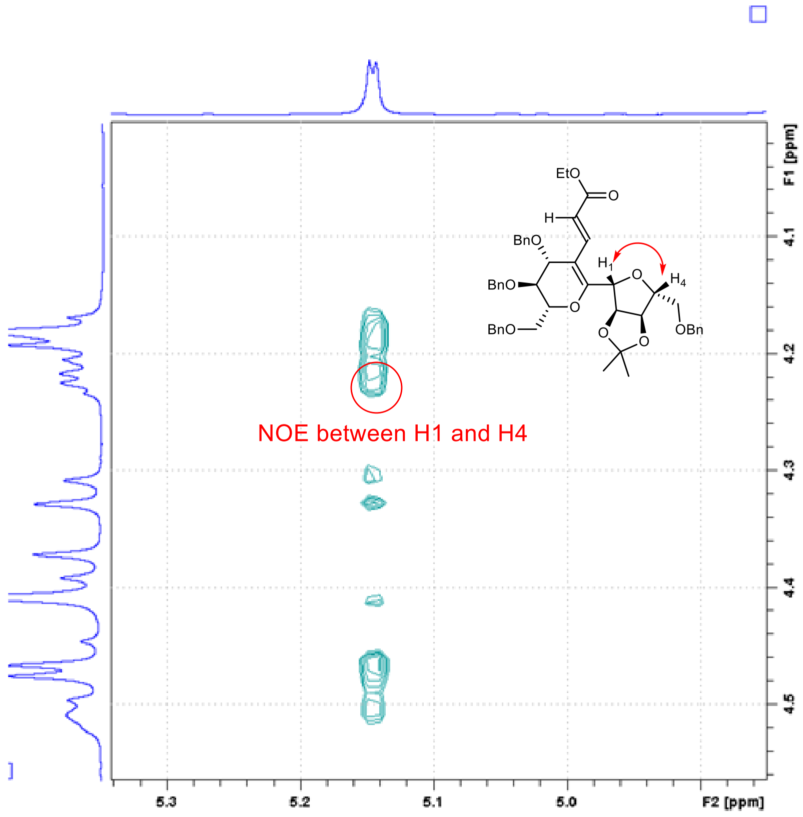

Supplementary Figure 107 NOESY spectra of compound **4r**

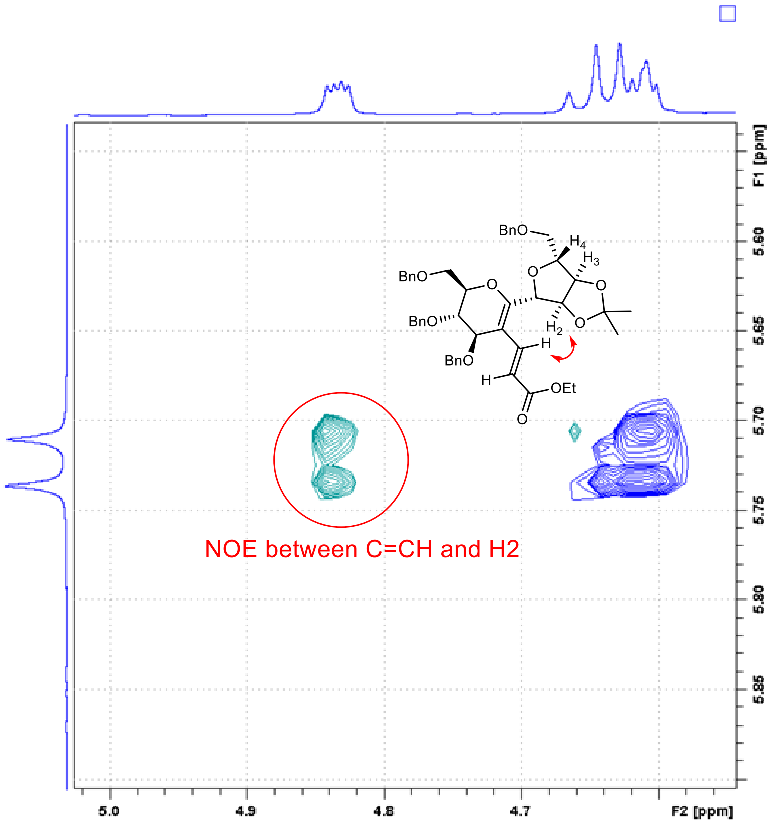

Supplementary Figure 108 NOESY spectra of compound **4r**

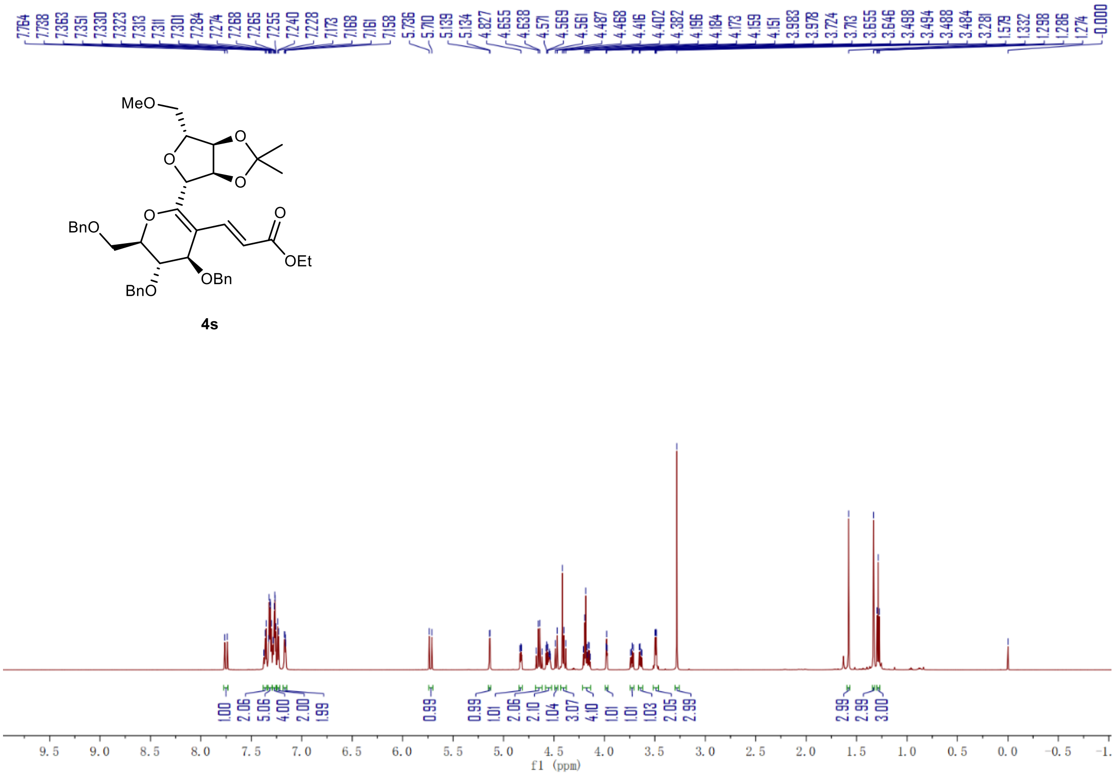

Supplementary Figure 109 ^{1}H NMR spectra of (600 MHz, $CDCl_3$) compound **4s**

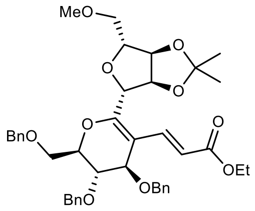

4s

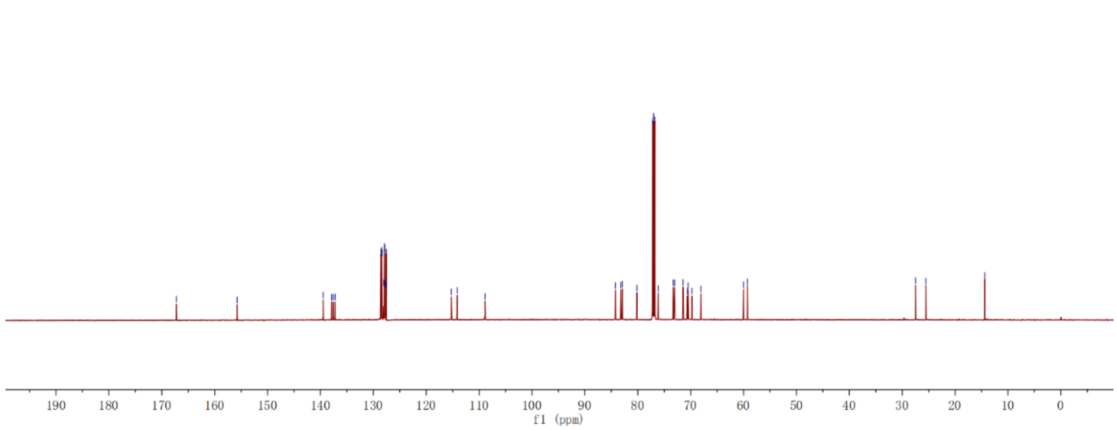

Supplementary Figure 110 ^{13}C spectra of (151 MHz, $CDCl_3$) compound **4s**

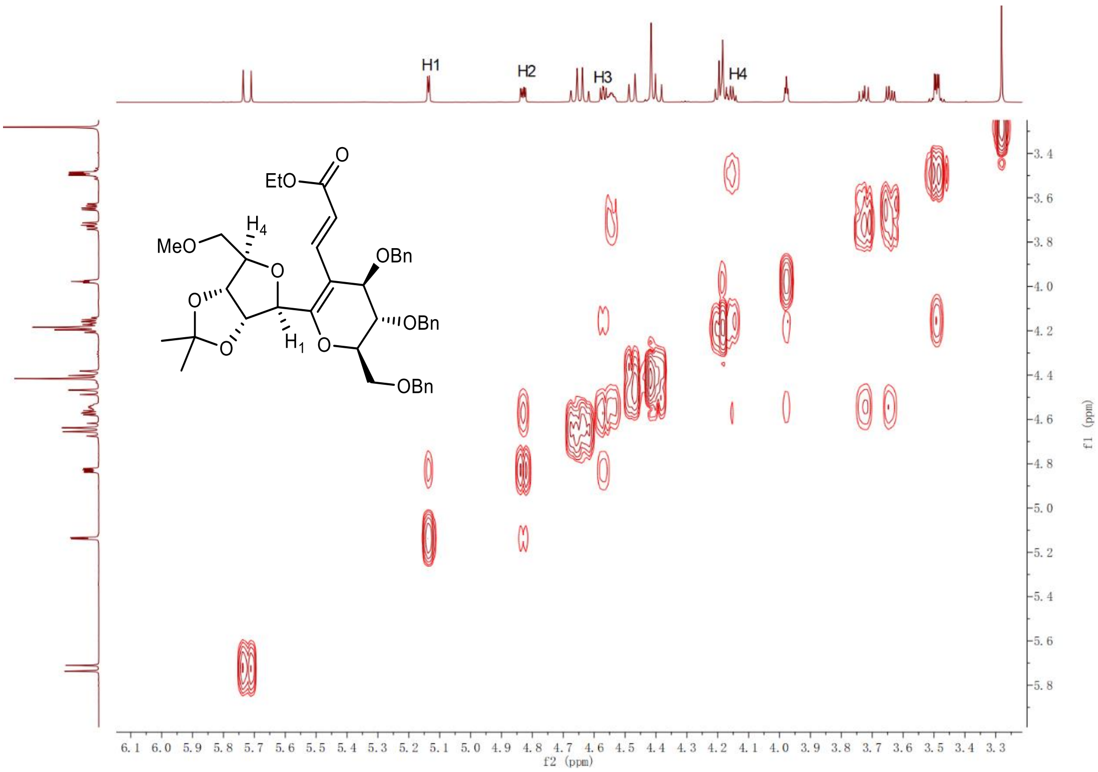

Supplementary Figure 111 COSY spectra of compound **4s**

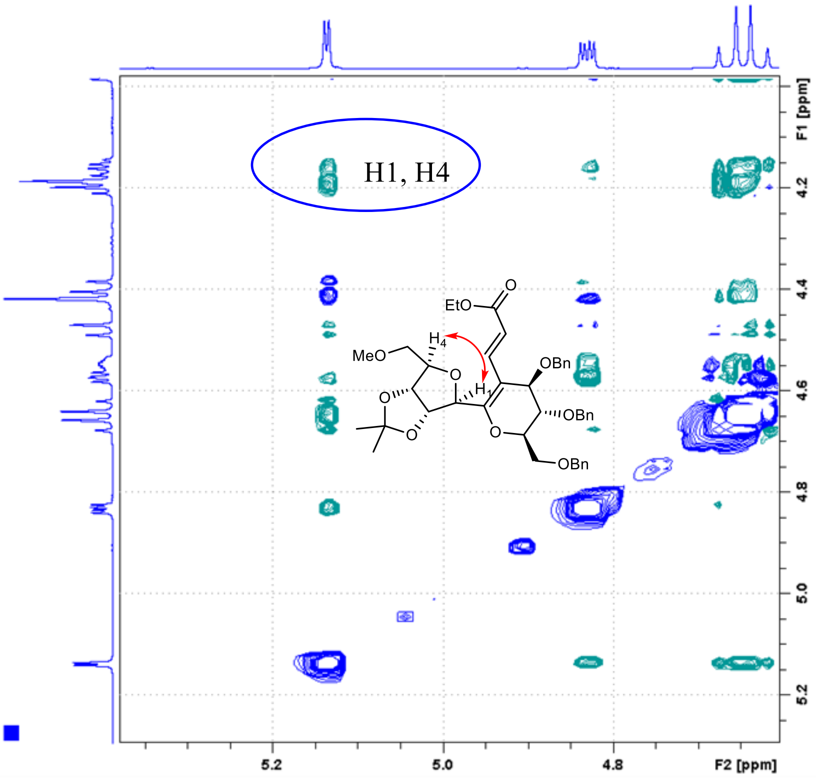

Supplementary Figure 112 NOESY spectra of compound **4s**

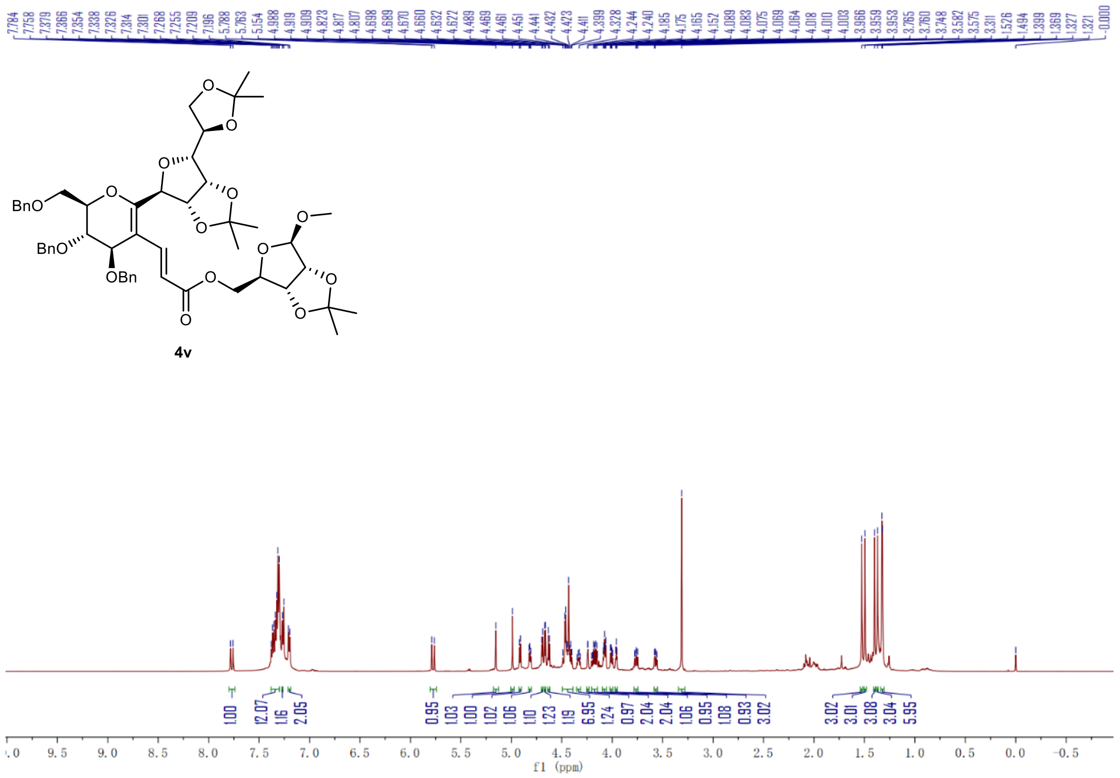

Supplementary Figure 113 ^{1}H NMR spectra of (600 MHz, $CDCl_3$) compound **4v**

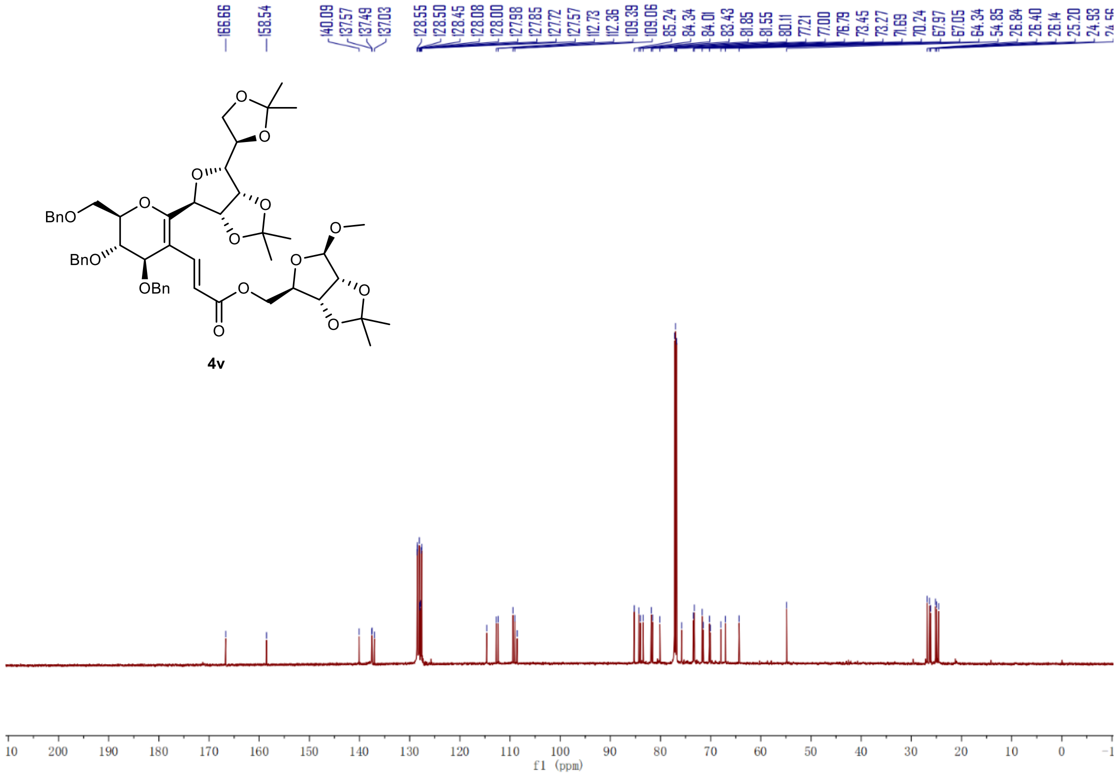

Supplementary Figure 114 ^{13}C spectra of (151 MHz, $CDCl_3$) compound **4v**

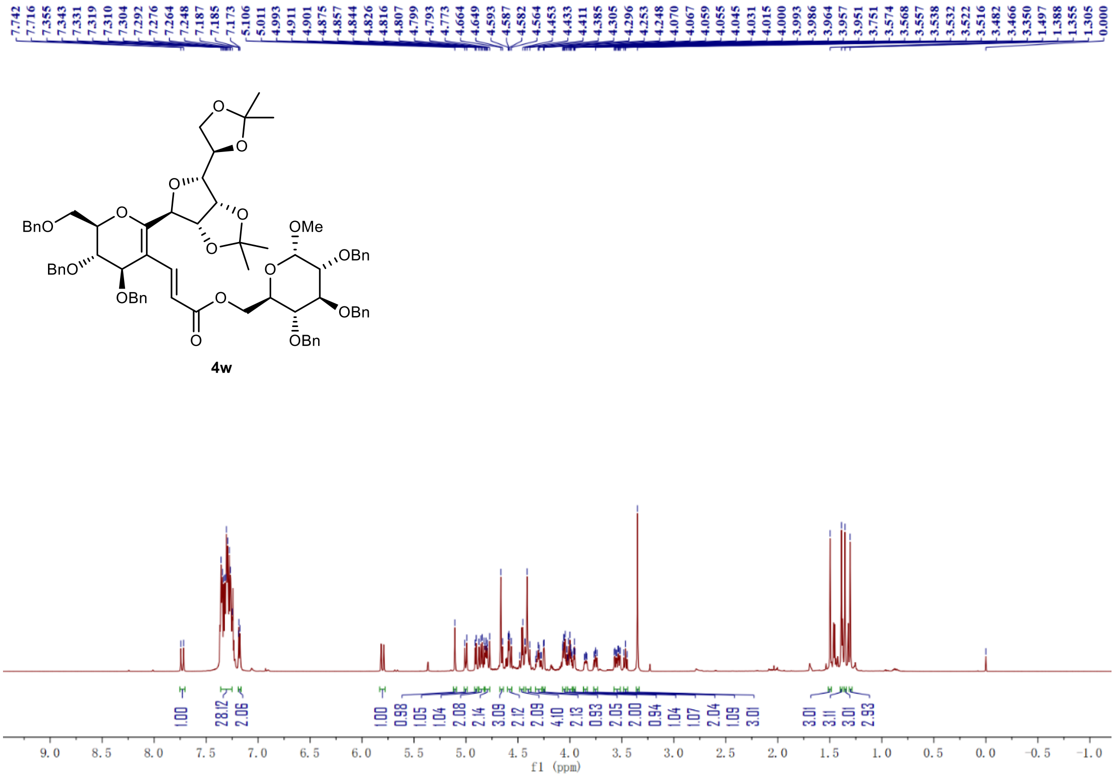

Supplementary Figure 115 ^{1}H NMR spectra of (600 MHz, $CDCl_3$) compound **4w**

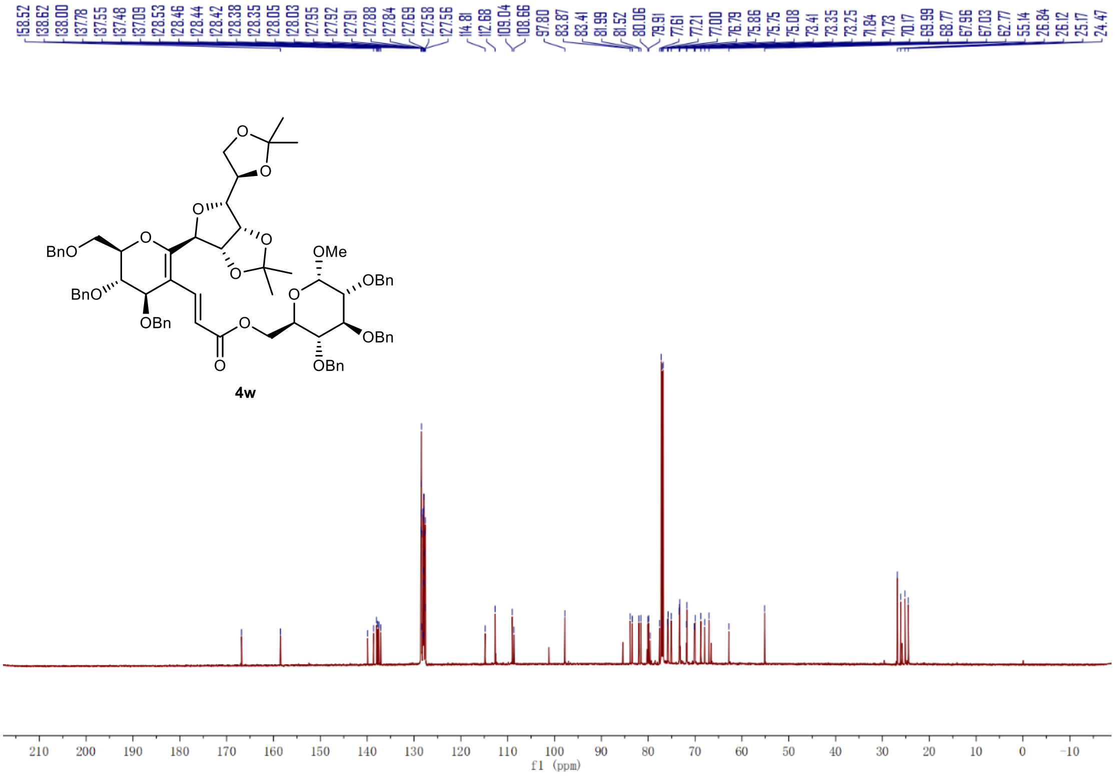

Supplementary Figure 116 ^{13}C spectra of (151 MHz, $CDCl_3$) compound **4w**

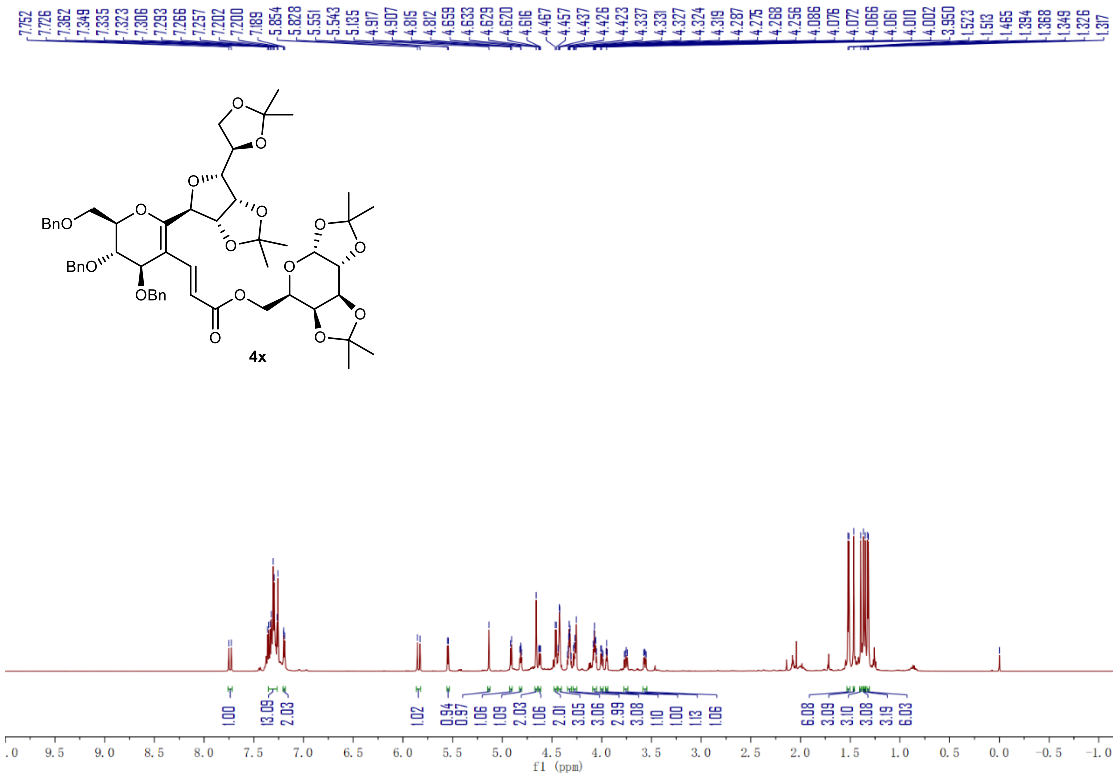

Supplementary Figure 117 ^{1}H NMR spectra of (600 MHz, $CDCl_3$) compound **4x**

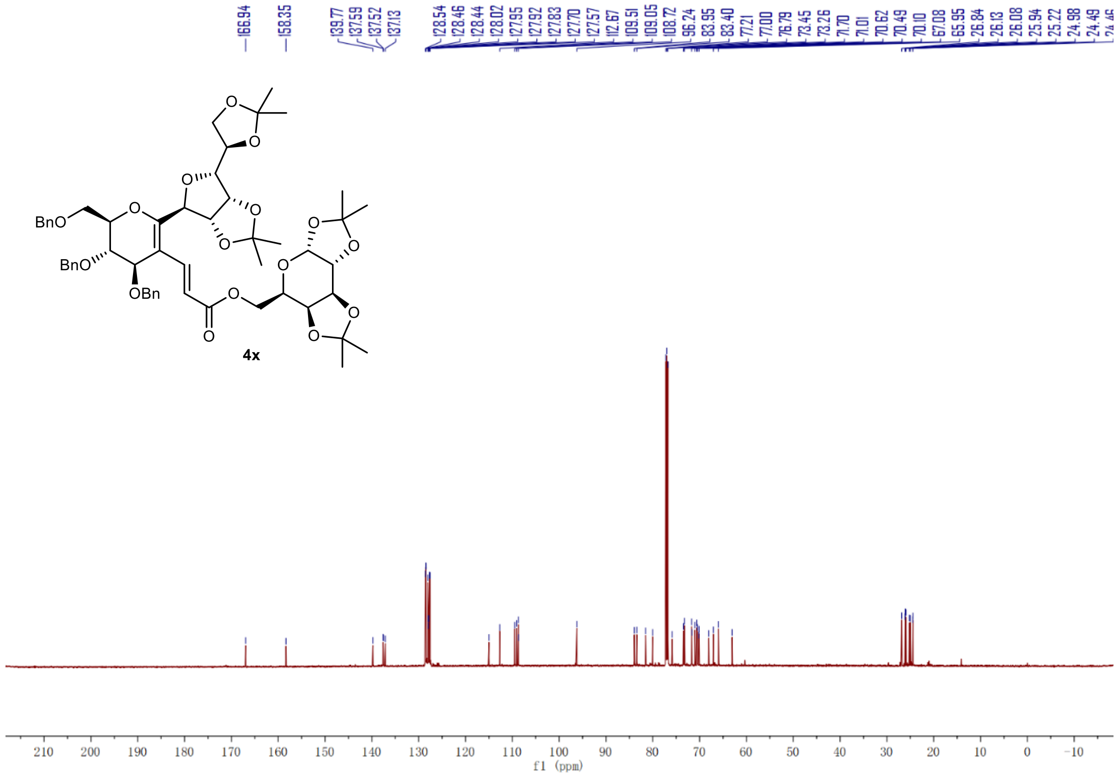

Supplementary Figure 118 ^{13}C spectra of (151 MHz, $CDCl_3$) compound **4x**

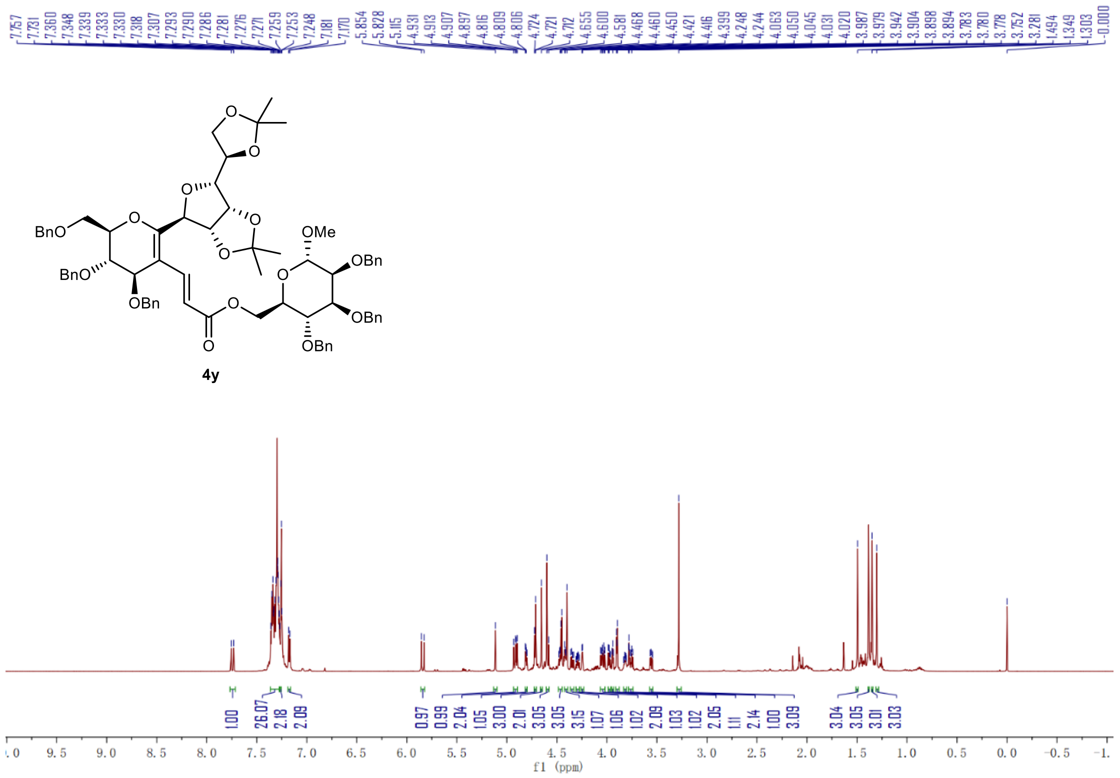

Supplementary Figure 119 ^{1}H NMR spectra of (600 MHz, $CDCl_3$) compound **4y**

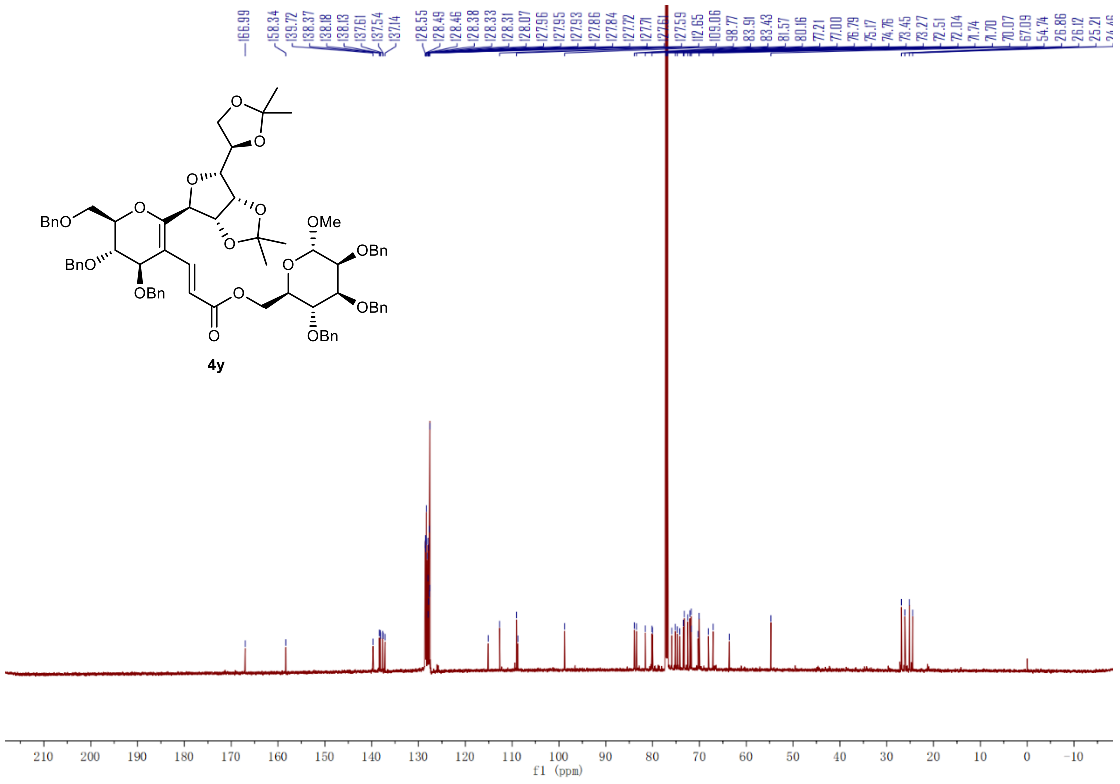

Supplementary Figure 120 ^{13}C spectra of (151 MHz, $CDCl_3$) compound **4y**

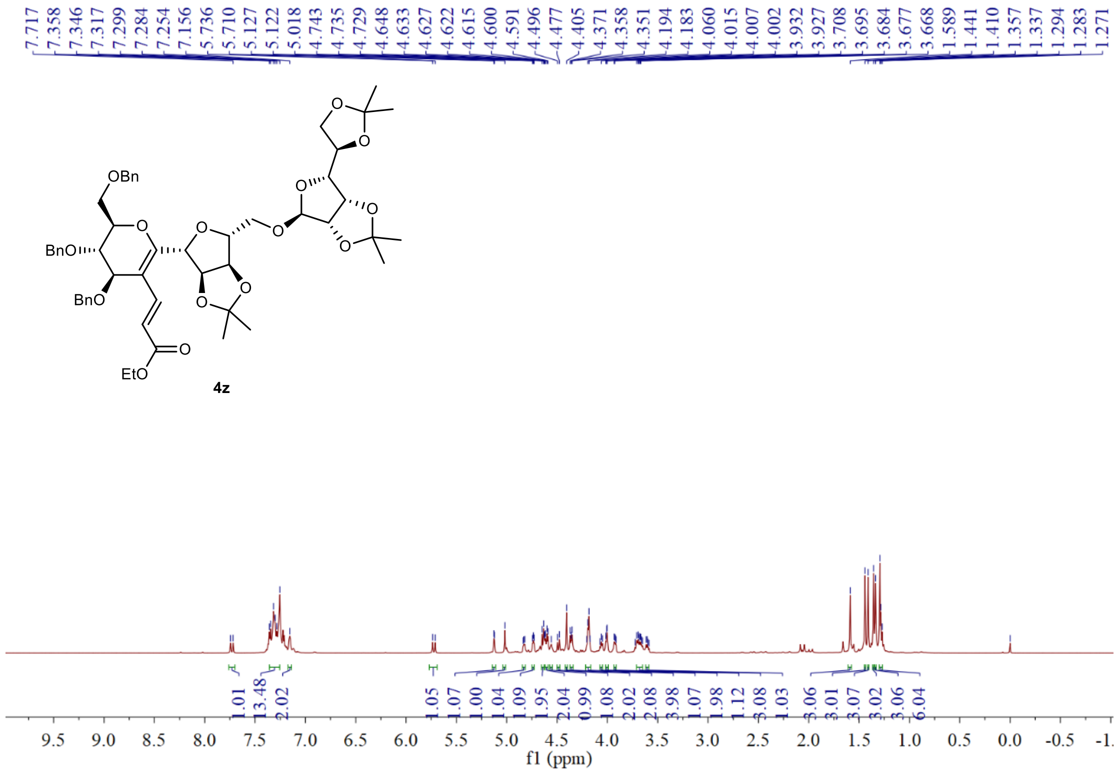

Supplementary Figure 121 ^{1}H NMR spectra of (600 MHz, $CDCl_3$) compound **4z**

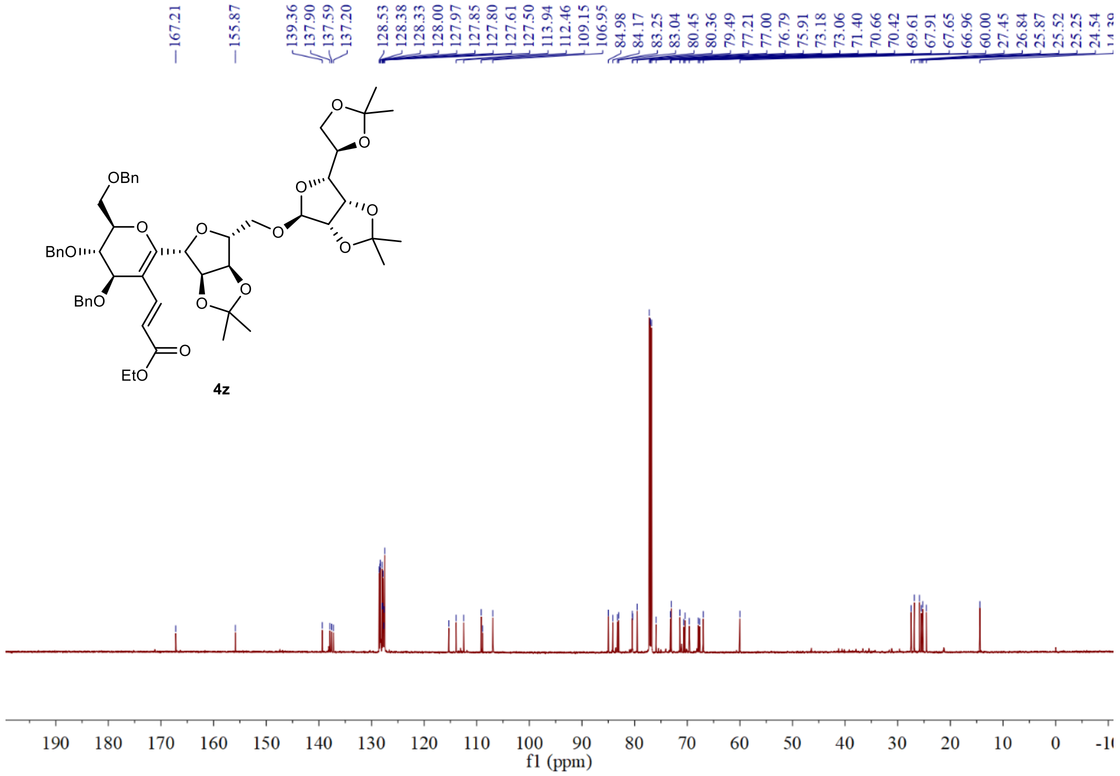

Supplementary Figure 122 ^{13}C spectra of (151 MHz, $CDCl_3$) compound **4z**

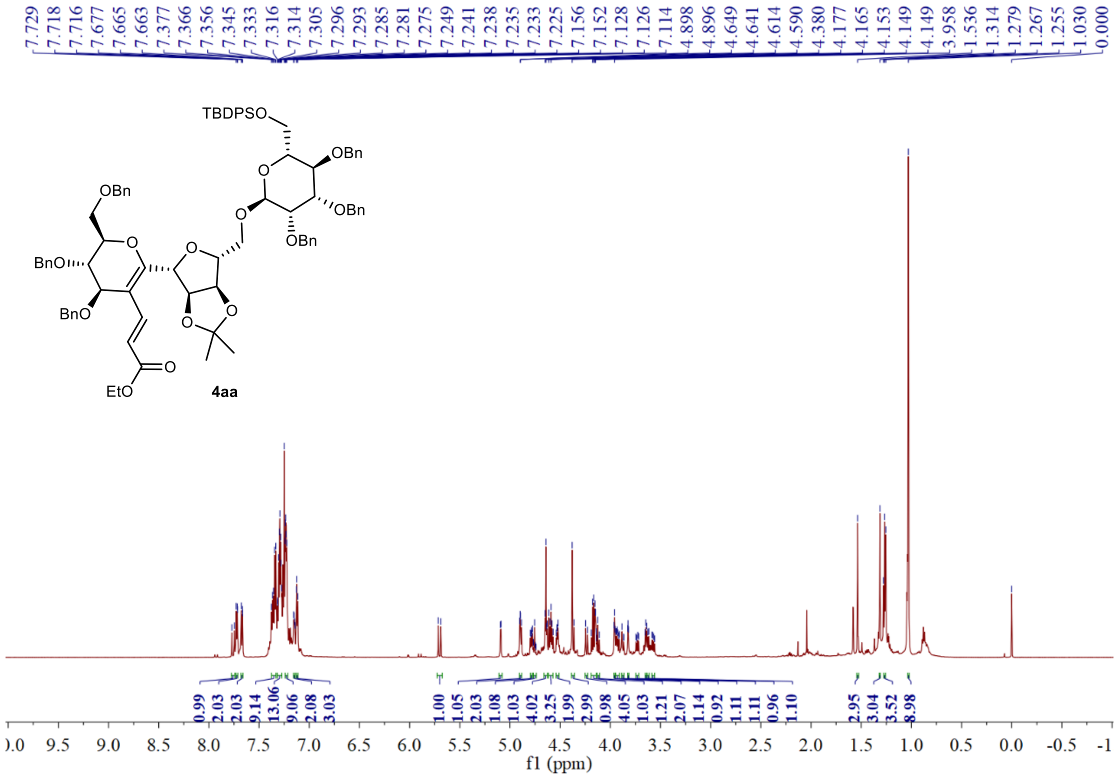

Supplementary Figure 123 ^{1}H NMR spectra of (600 MHz, $CDCl_3$) compound **4aa**

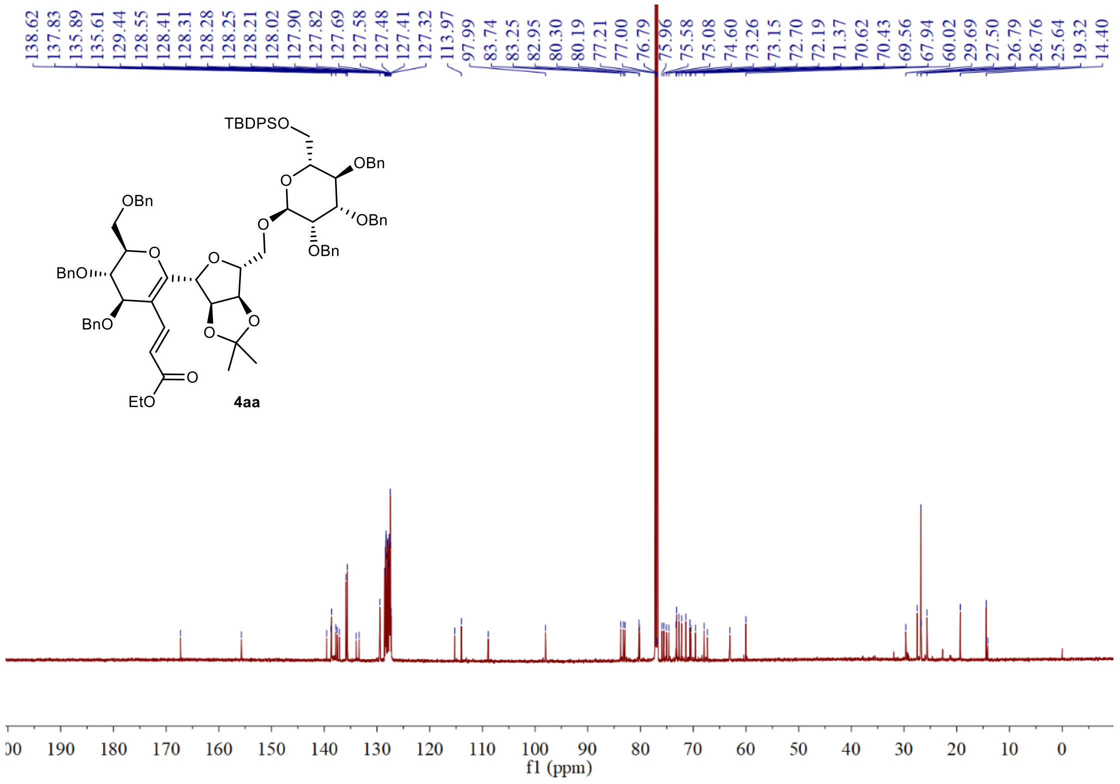

Supplementary Figure 124 ^{13}C spectra of (151 MHz, $CDCl_3$) compound **4aa**

(^{1}H NMR, 600 MHz, $CDCl_3$; ^{13}C {^{1}H} NMR, 151 MHz, $CDCl_3$)

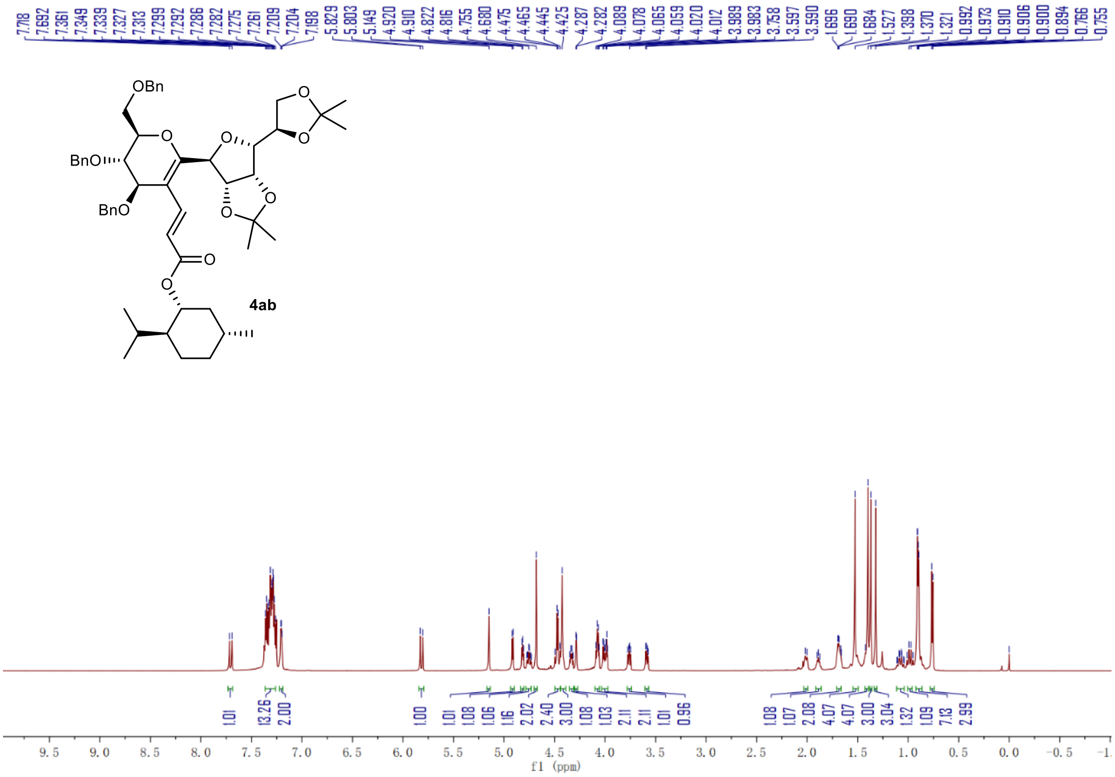

Supplementary Figure 125 ^{1}H NMR spectra of (600 MHz, $CDCl_3$) compound **4ab**

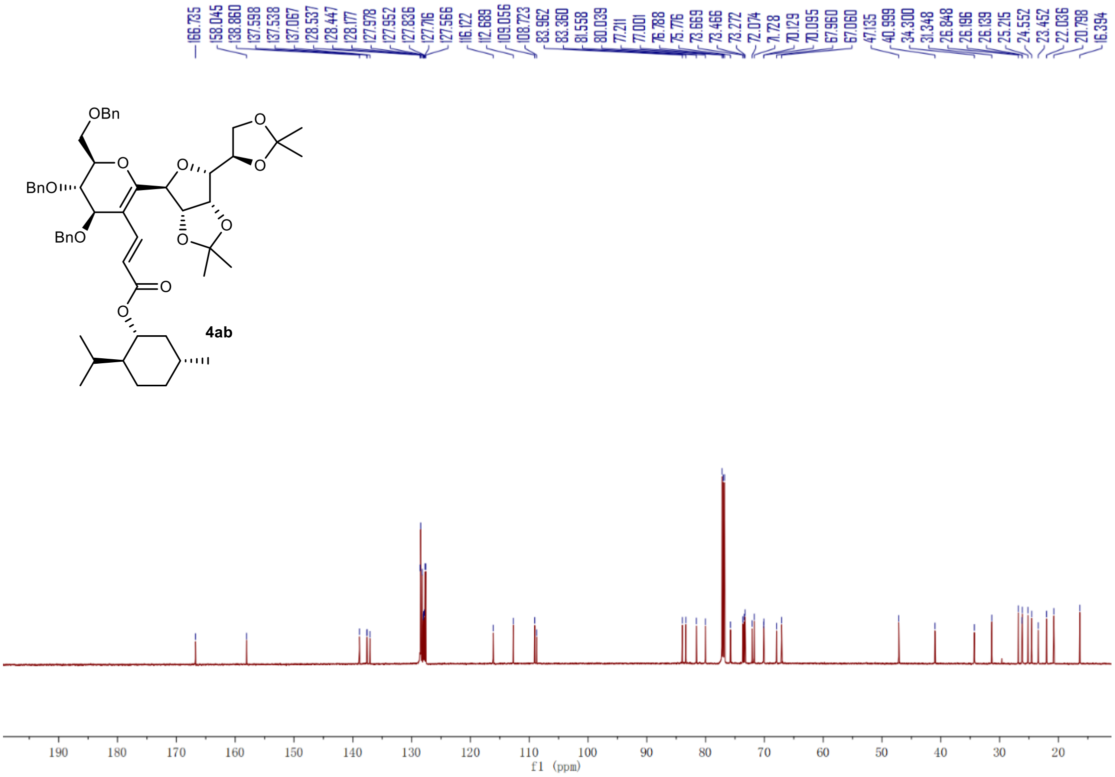

Supplementary Figure 126 ^{13}C spectra of (151 MHz, $CDCl_3$) compound **4ab**

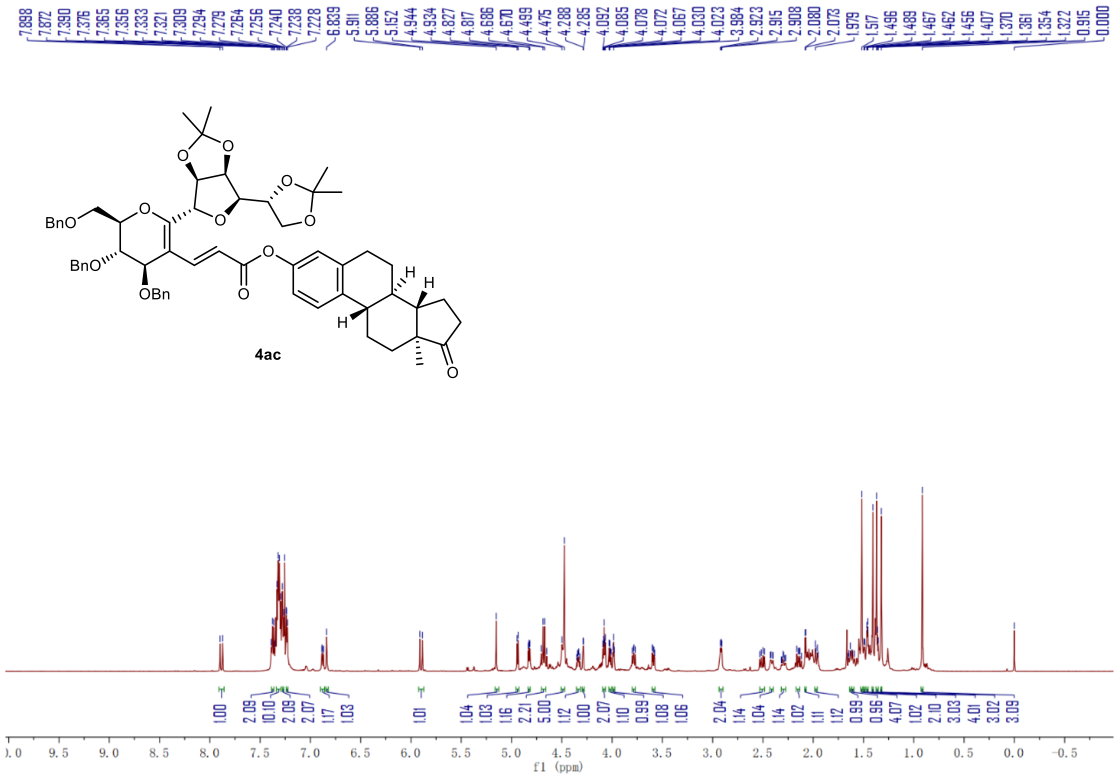

Supplementary Figure 127 ^{1}H NMR spectra of (600 MHz, $CDCl_3$) compound **4ac**

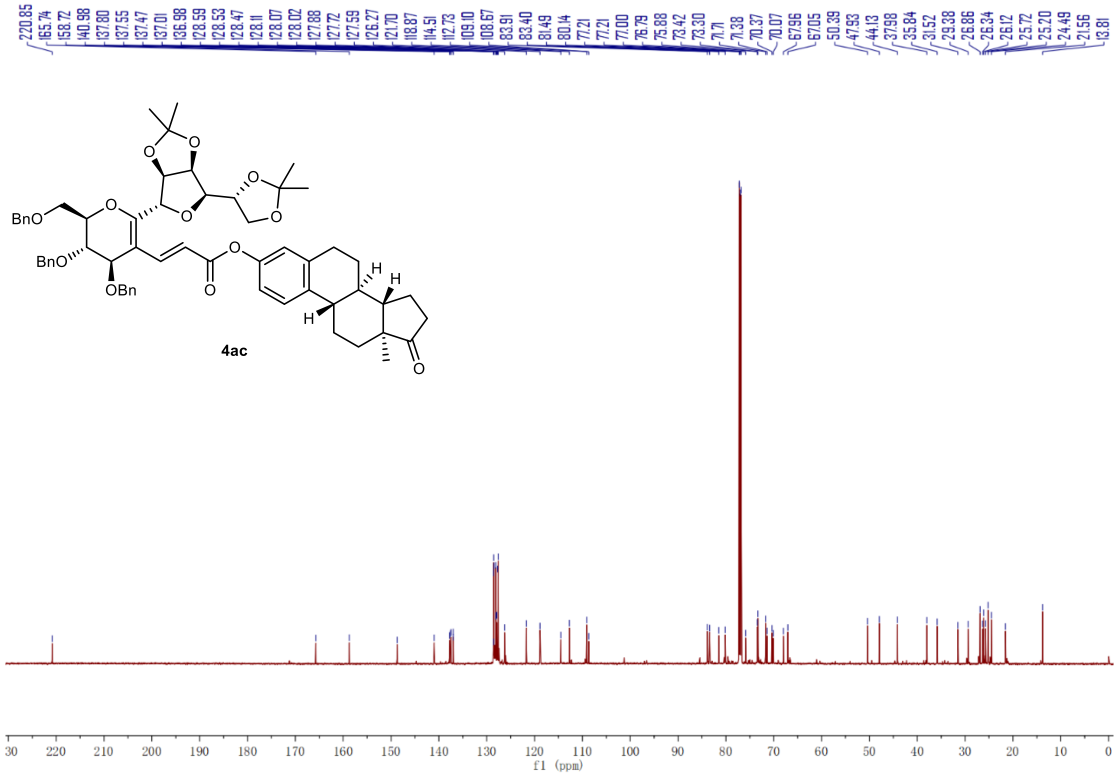

Supplementary Figure 128 ^{13}C spectra of (151 MHz, $CDCl_3$) compound **4ac**

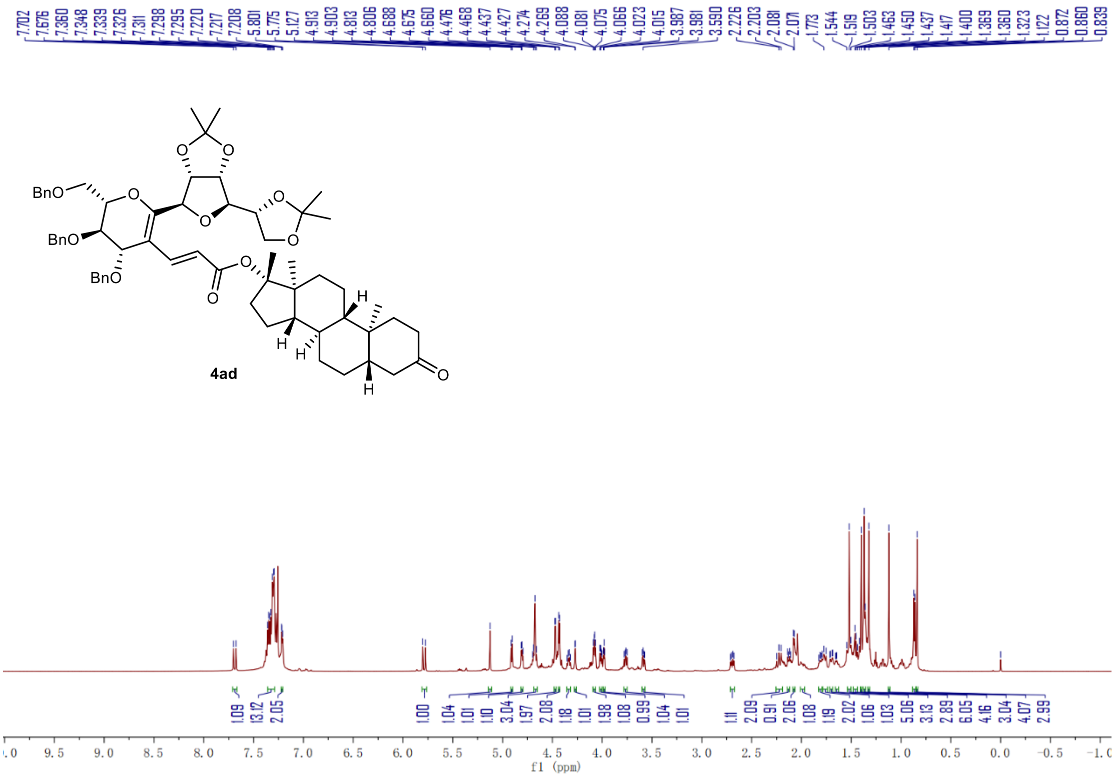

Supplementary Figure 129 ^{1}H NMR spectra of (600 MHz, $CDCl_3$) compound **4ad**

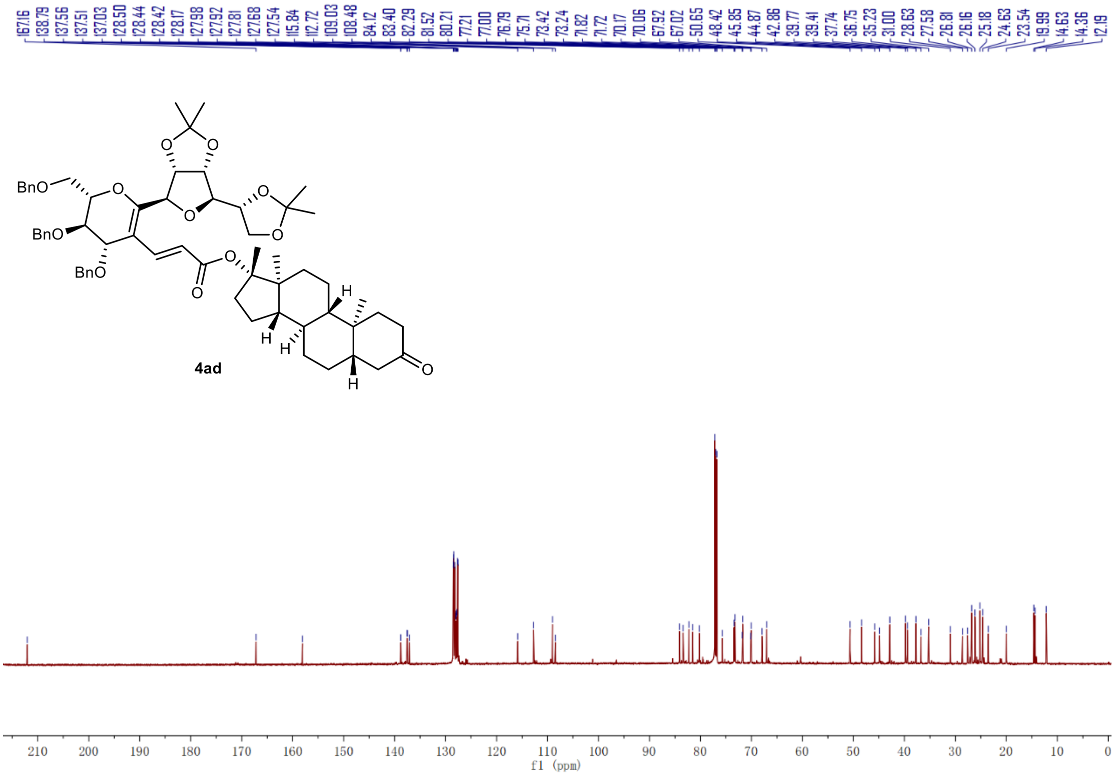

Supplementary Figure 130 ^{13}C spectra of (151 MHz, $CDCl_3$) compound **4ad**

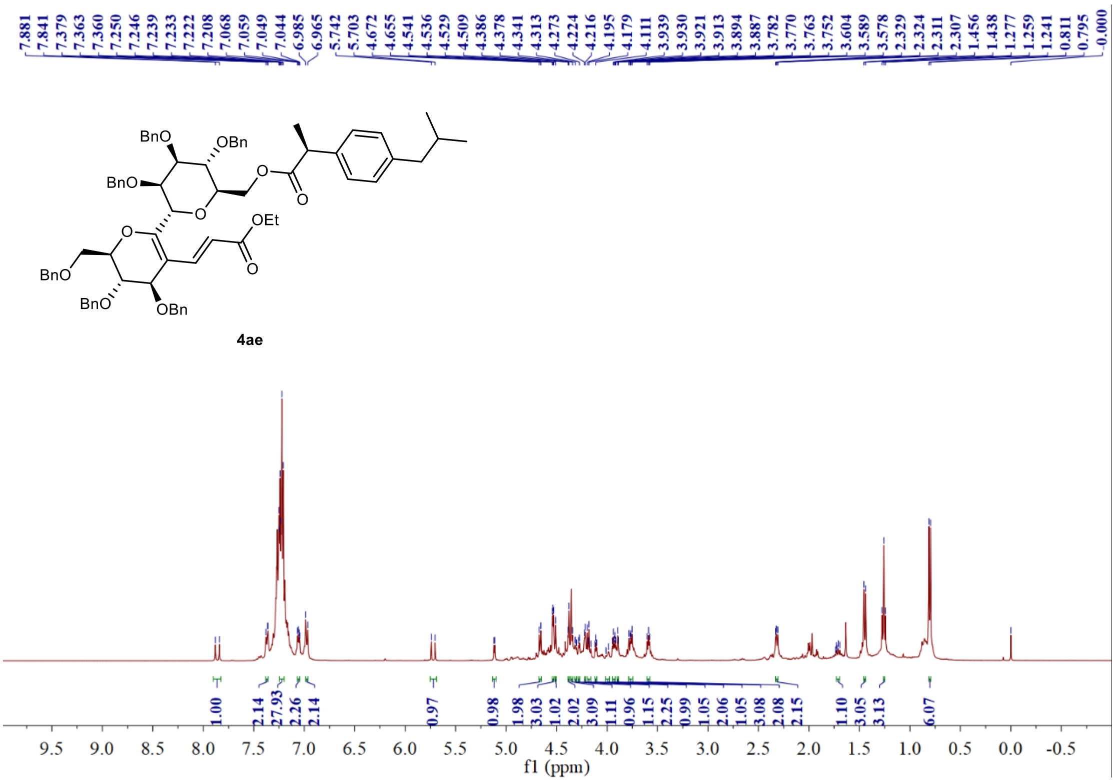

Supplementary Figure 131 ^{1}H NMR spectra of (600 MHz, $CDCl_3$) compound **4ae**

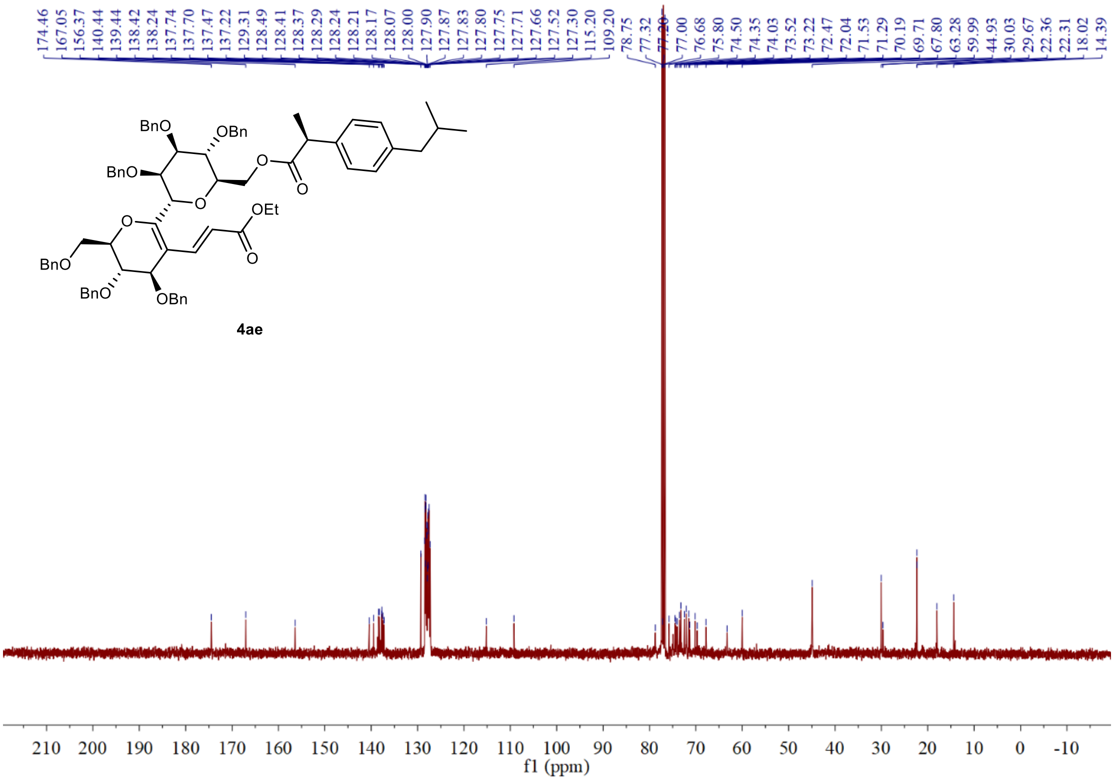

Supplementary Figure 132 ^{13}C spectra of (151 MHz, $CDCl_3$) compound **4ae**

(^{1}H NMR, 600 MHz, CDCl$_3$; ^{13}C {^{1}H} NMR, 151 MHz, CDCl$_3$)

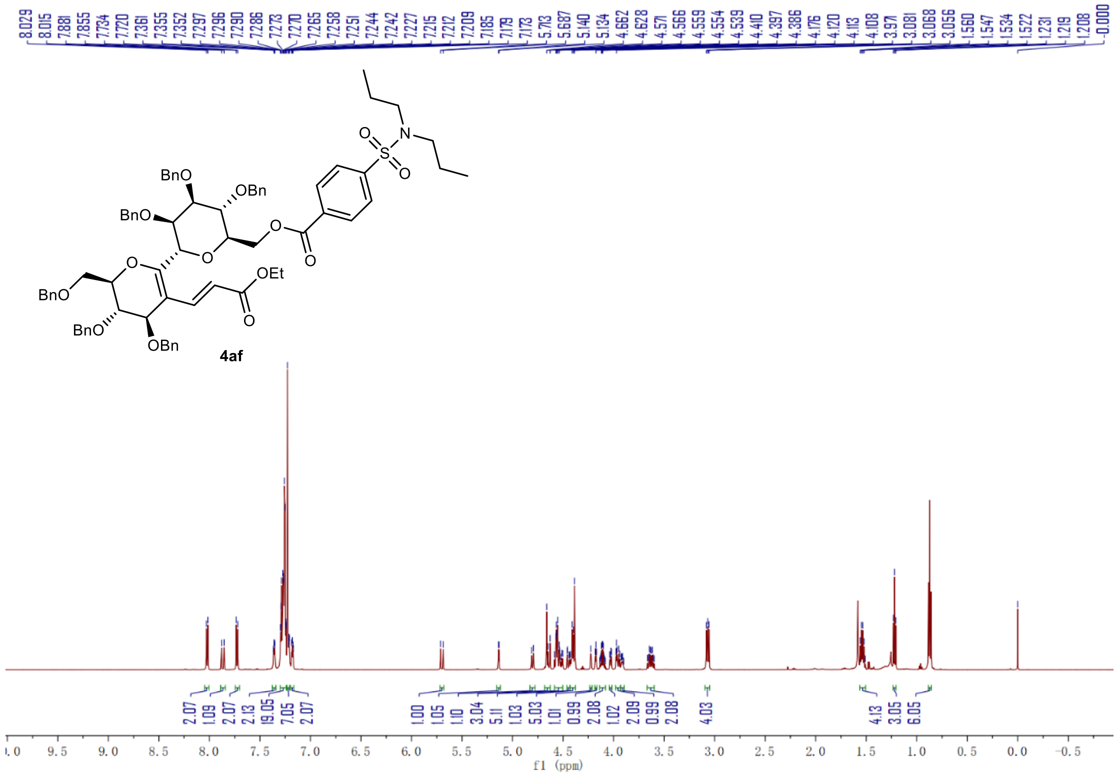

Supplementary Figure 133 ^{1}H NMR spectra of (600 MHz, CDCl$_3$) compound **4af**

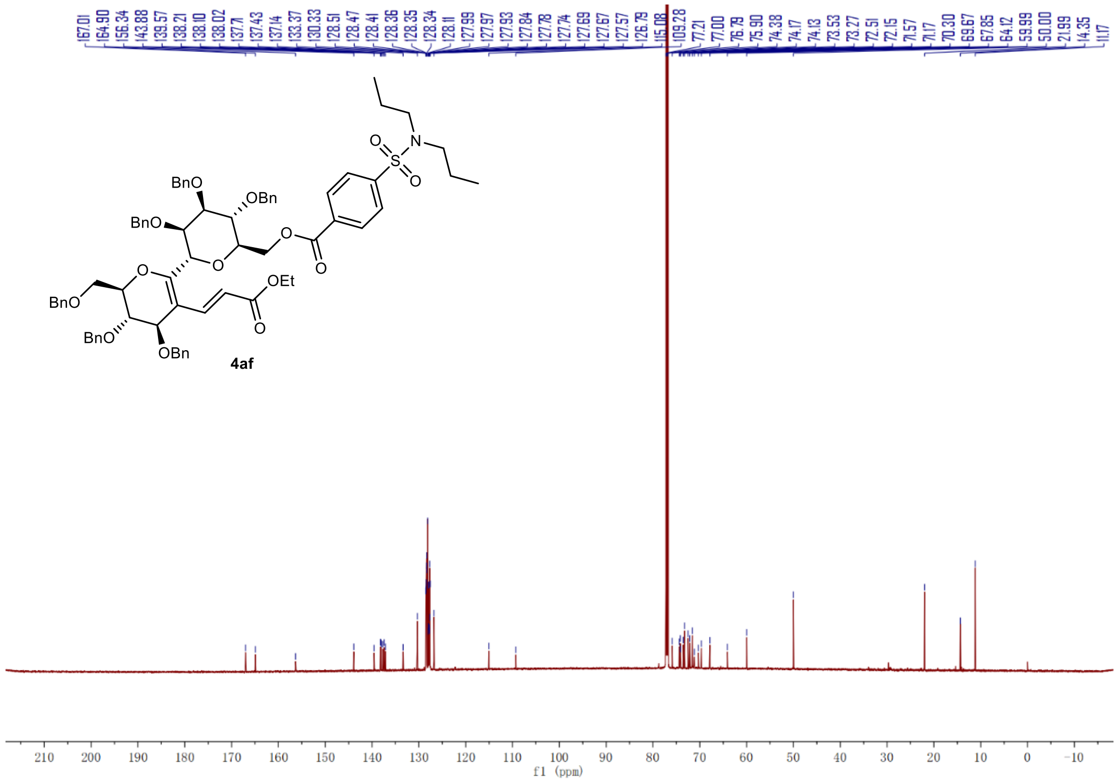

Supplementary Figure 134 ^{13}C spectra of (151 MHz, CDCl$_3$) compound **4af**

(^{1}H NMR, 600 MHz, CDCl$_3$; ^{13}C {^{1}H} NMR, 151 MHz, CDCl$_3$)

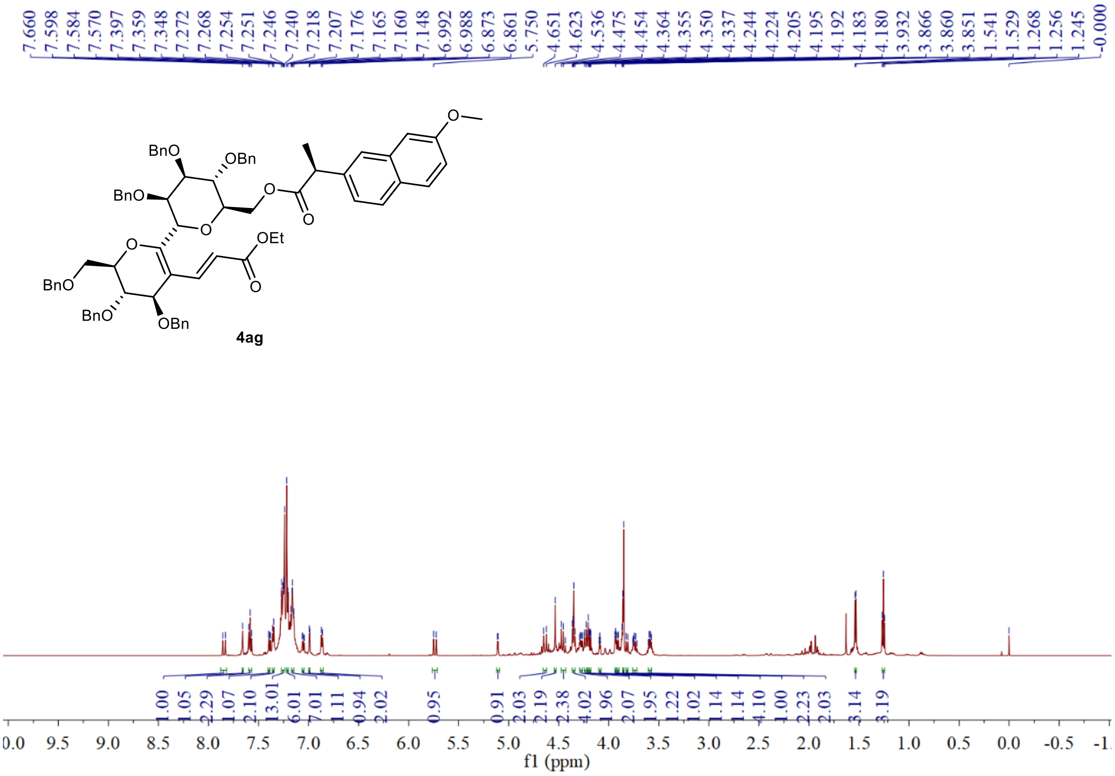

Supplementary Figure 135 ^{1}H NMR spectra of (600 MHz, CDCl$_3$) compound **4ag**

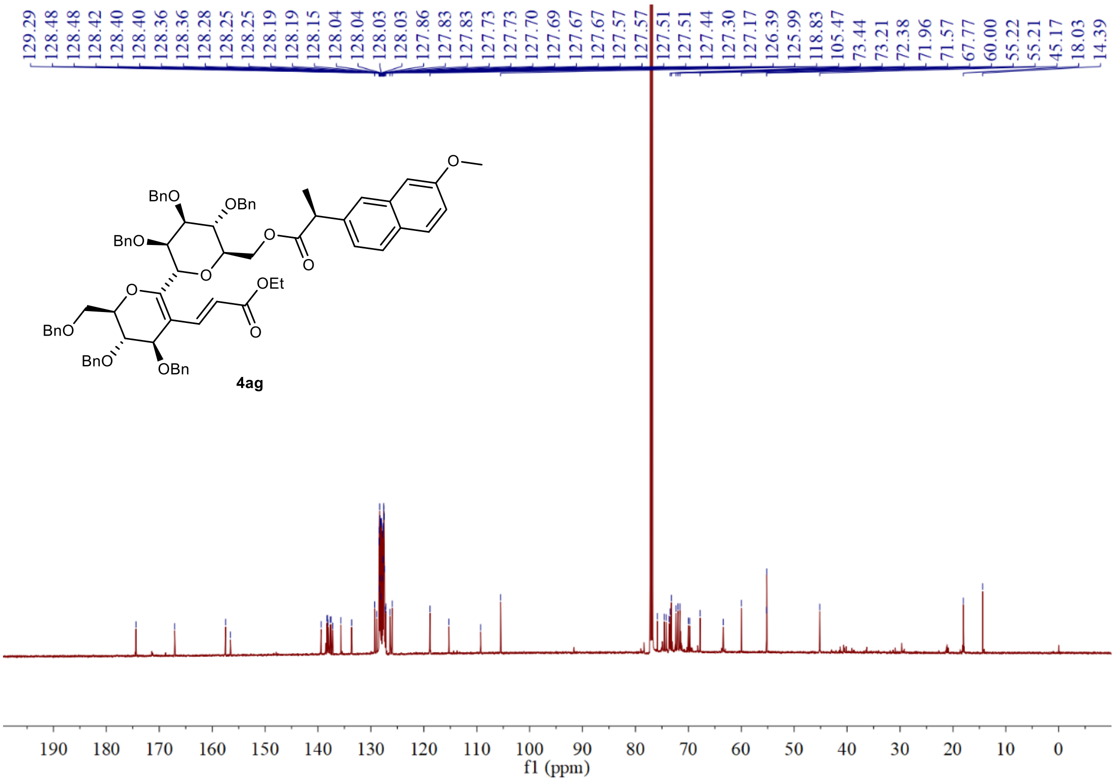

Supplementary Figure 136 ^{13}C spectra of (151 MHz, CDCl$_3$) compound **4ag**

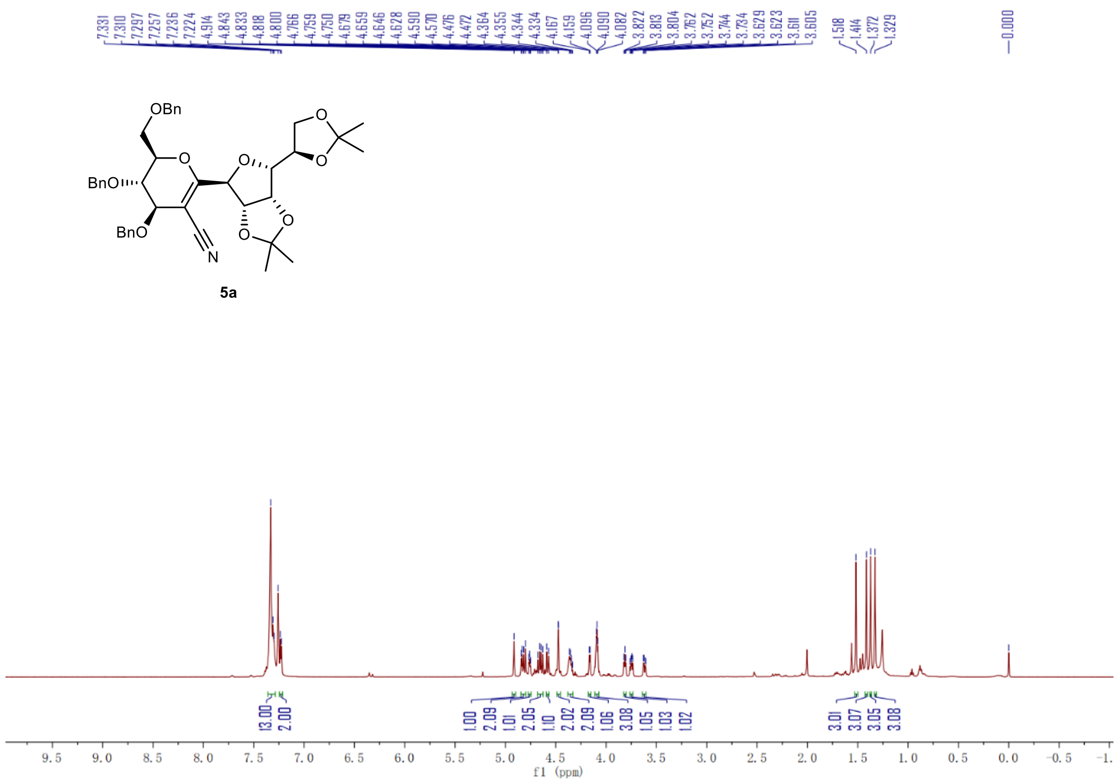

Supplementary Figure 137 ^{1}H NMR spectra of (600 MHz, $CDCl_3$) compound **5a**

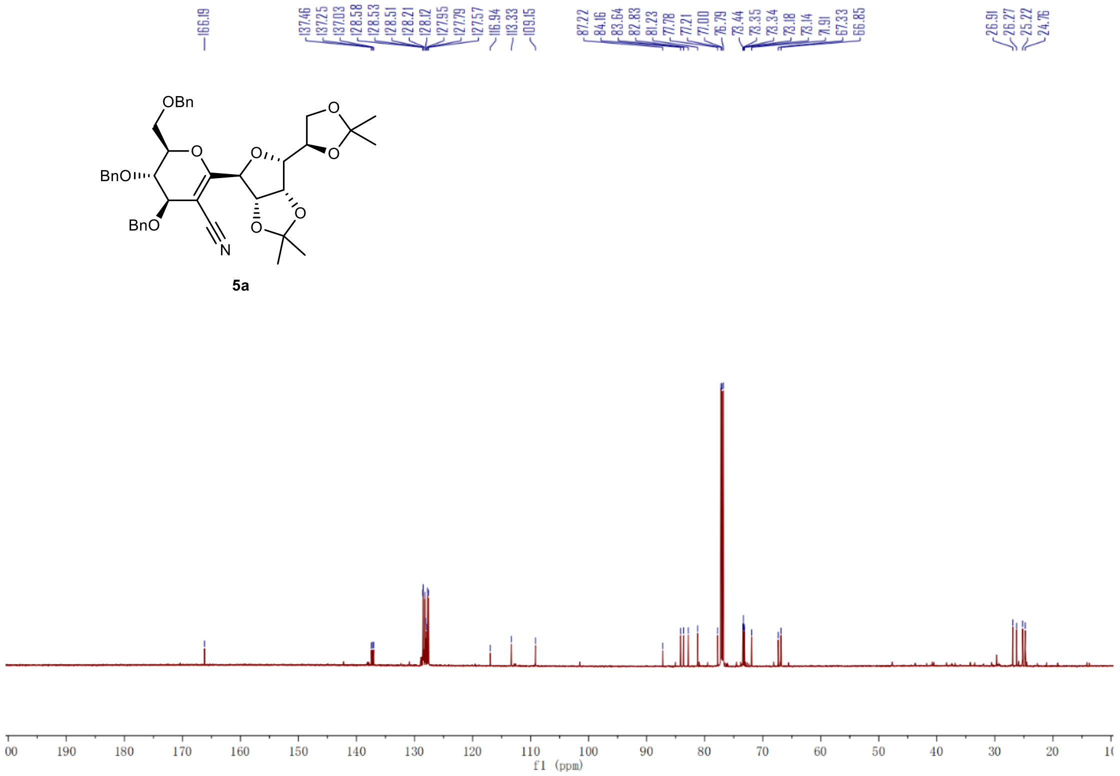

Supplementary Figure 138 ^{13}C spectra of (151 MHz, $CDCl_3$) compound **5a**

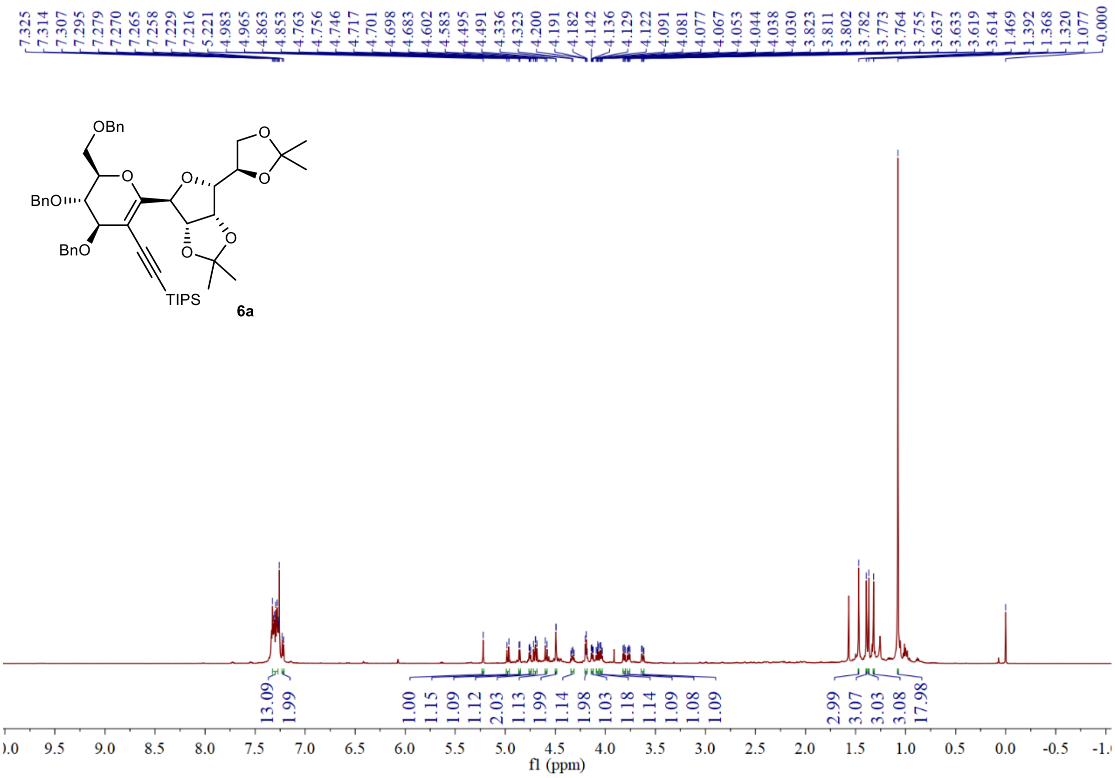

Supplementary Figure 139 ^{1}H NMR spectra of (600 MHz, $CDCl_3$) compound **6a**

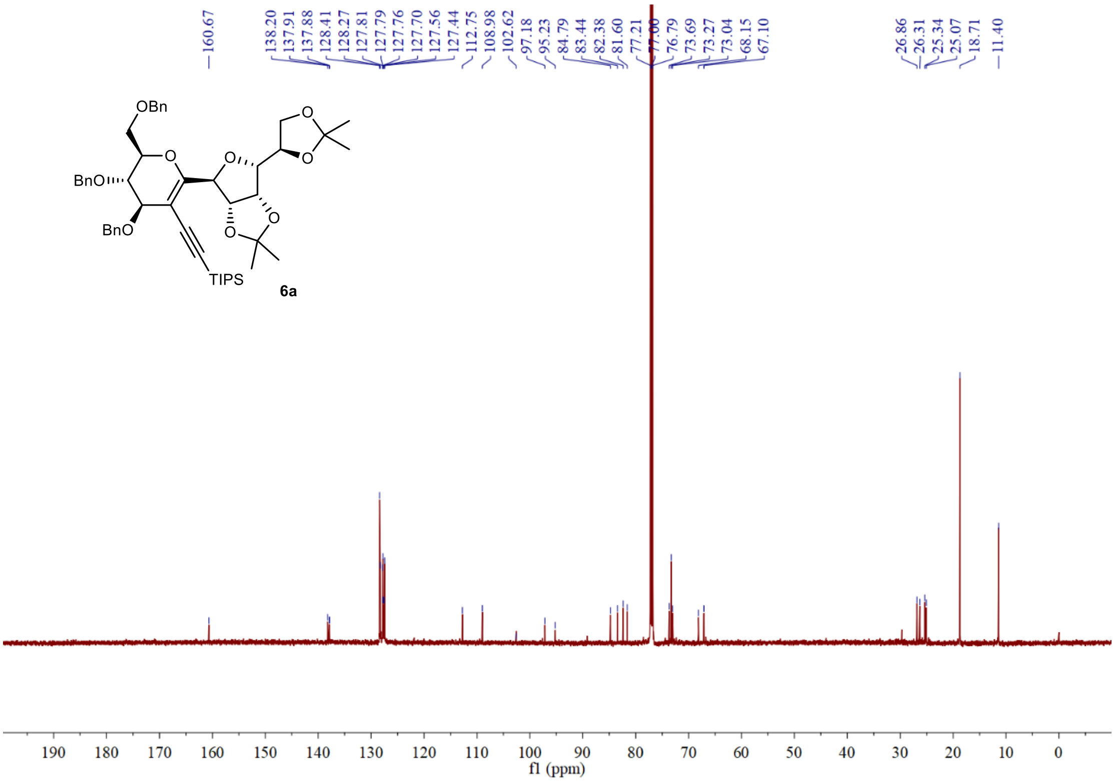

Supplementary Figure 140 ^{13}C spectra of (151 MHz, $CDCl_3$) compound **6a**

(^{1}H NMR, 600 MHz, CDCl3; ^{13}C {^{1}H} NMR, 151 MHz, CDCl3)

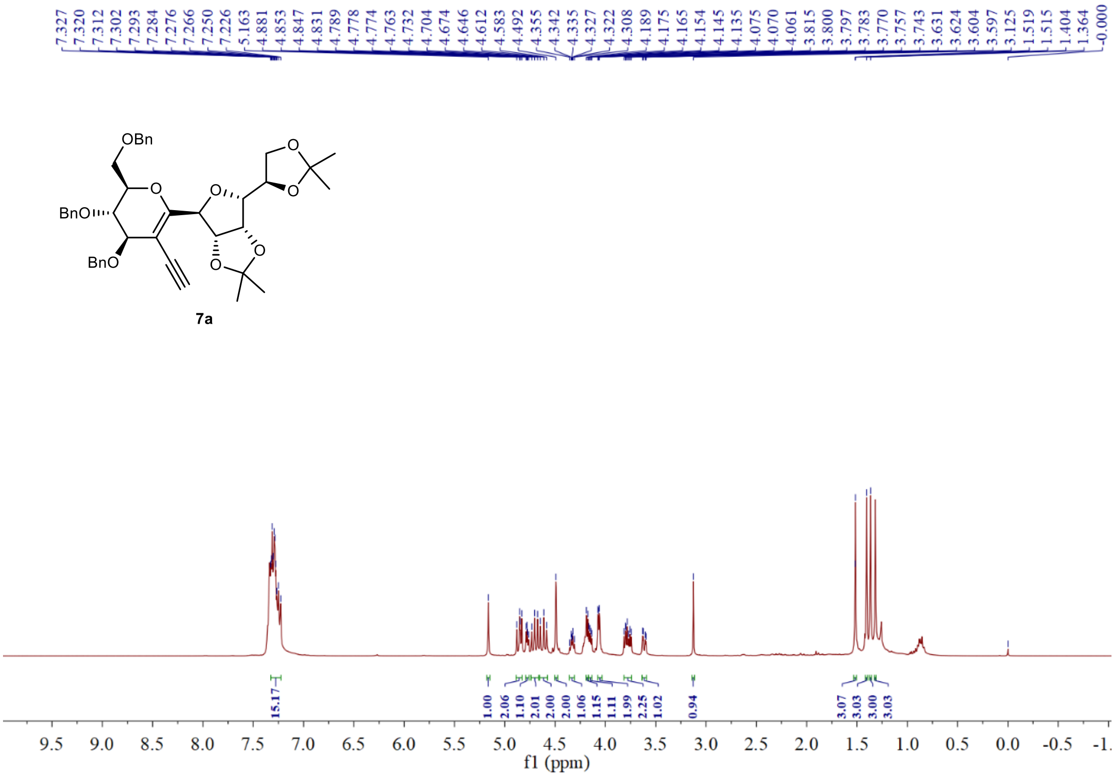

Supplementary Figure 141 ^{1}H NMR spectra of (600 MHz, CDCl3) compound **7a**

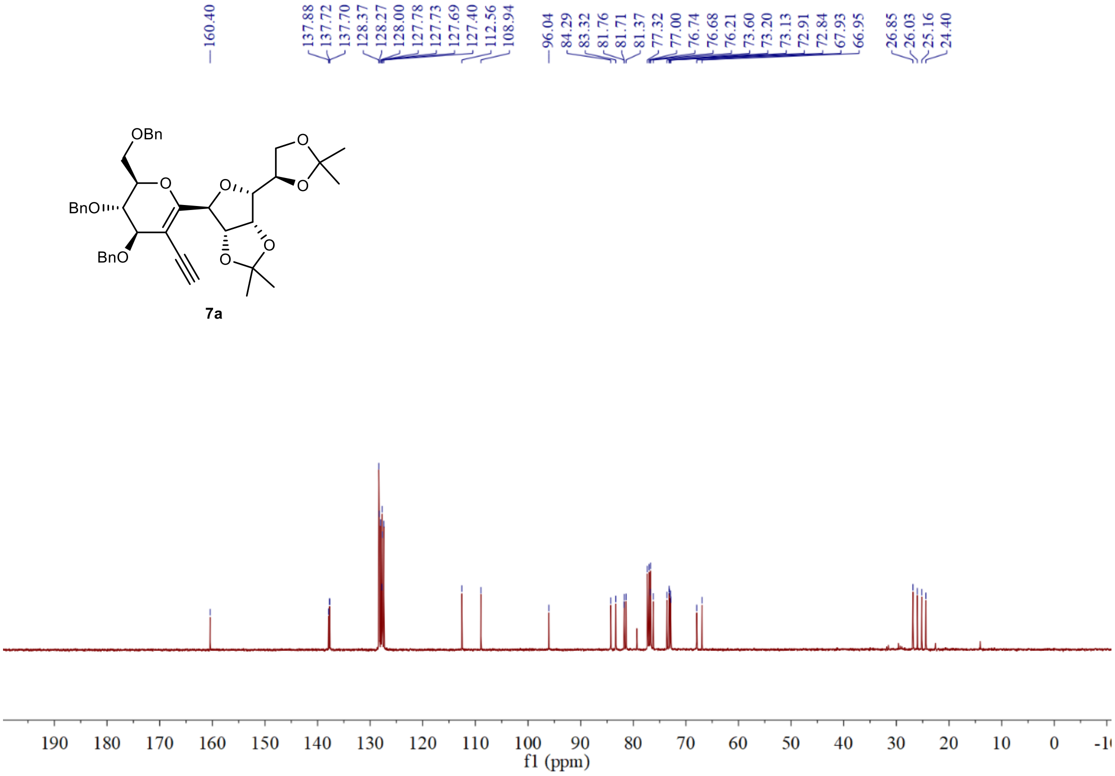

Supplementary Figure 142 ^{13}C spectra of (151 MHz, CDCl3) compound **7a**

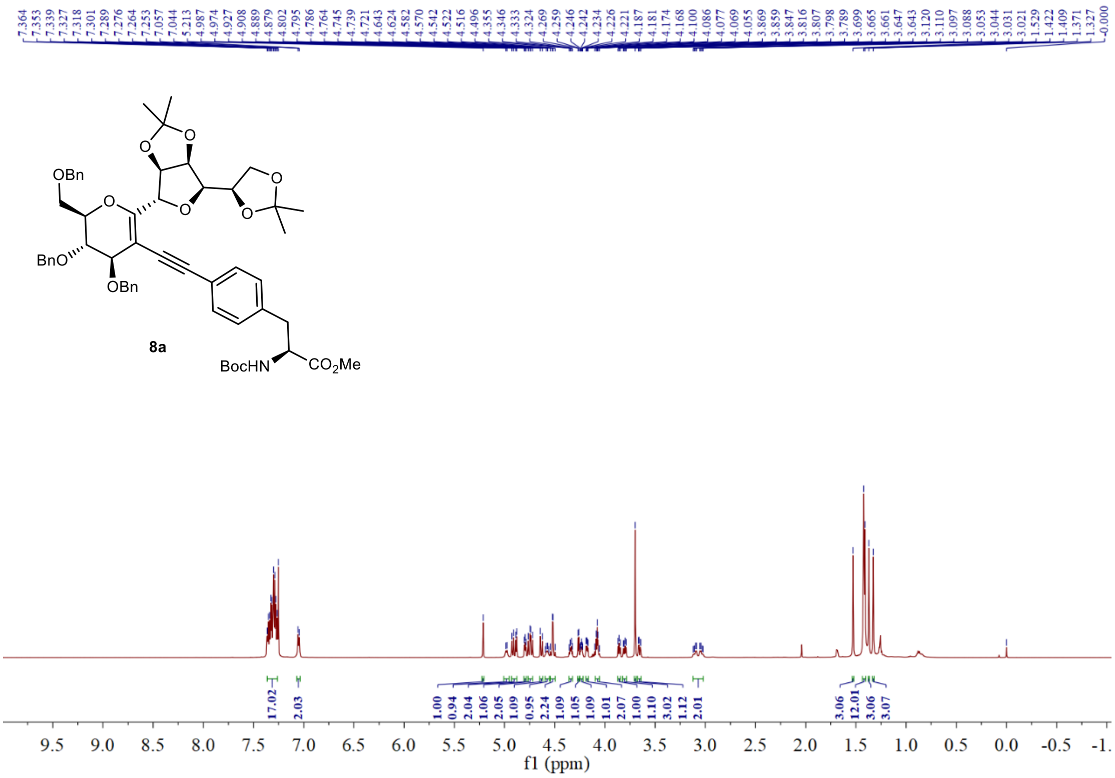

Supplementary Figure 143 ^{1}H NMR spectra of (600 MHz, $CDCl_3$) compound **8a**

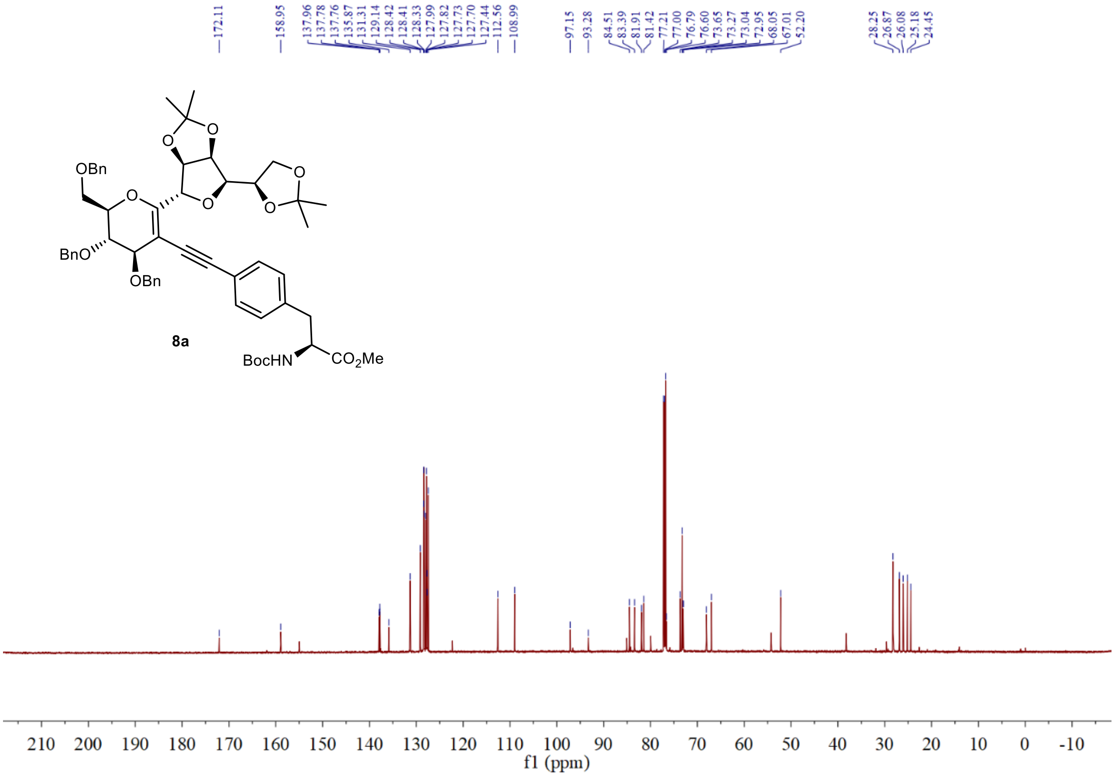

Supplementary Figure 144 ^{13}C spectra of (151 MHz, $CDCl_3$) compound **8a**

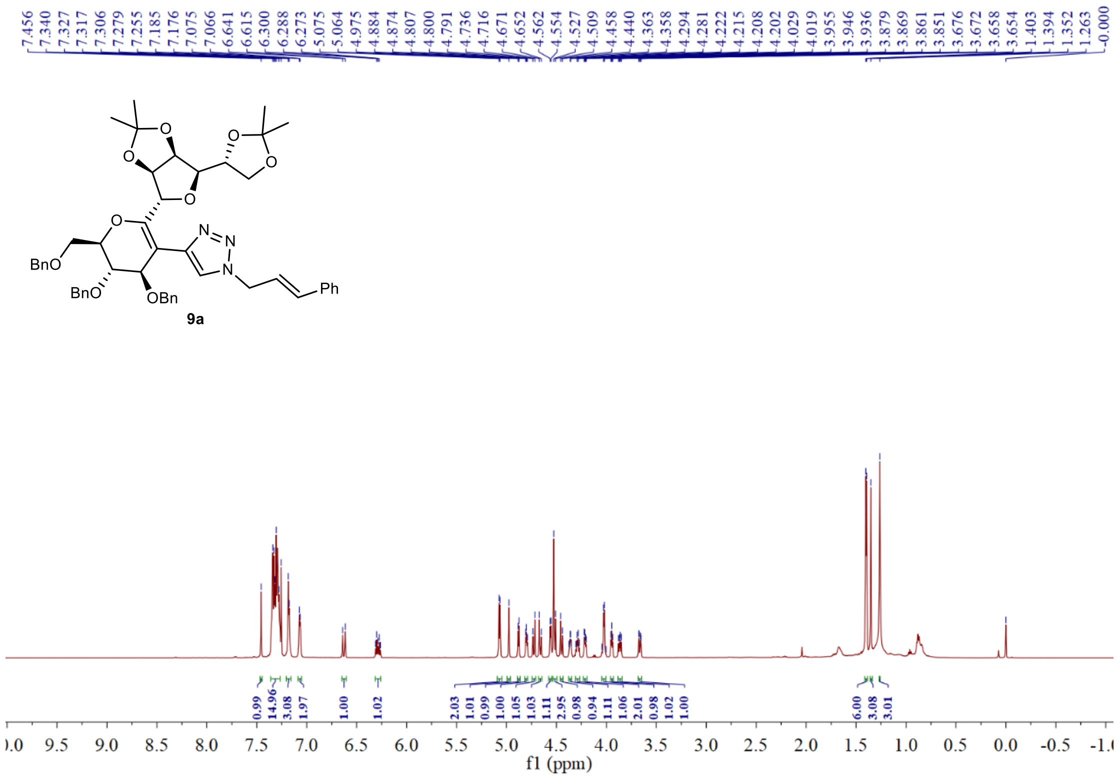

Supplementary Figure 145 ^{1}H NMR spectra of (600 MHz, $CDCl_3$) compound **9a**

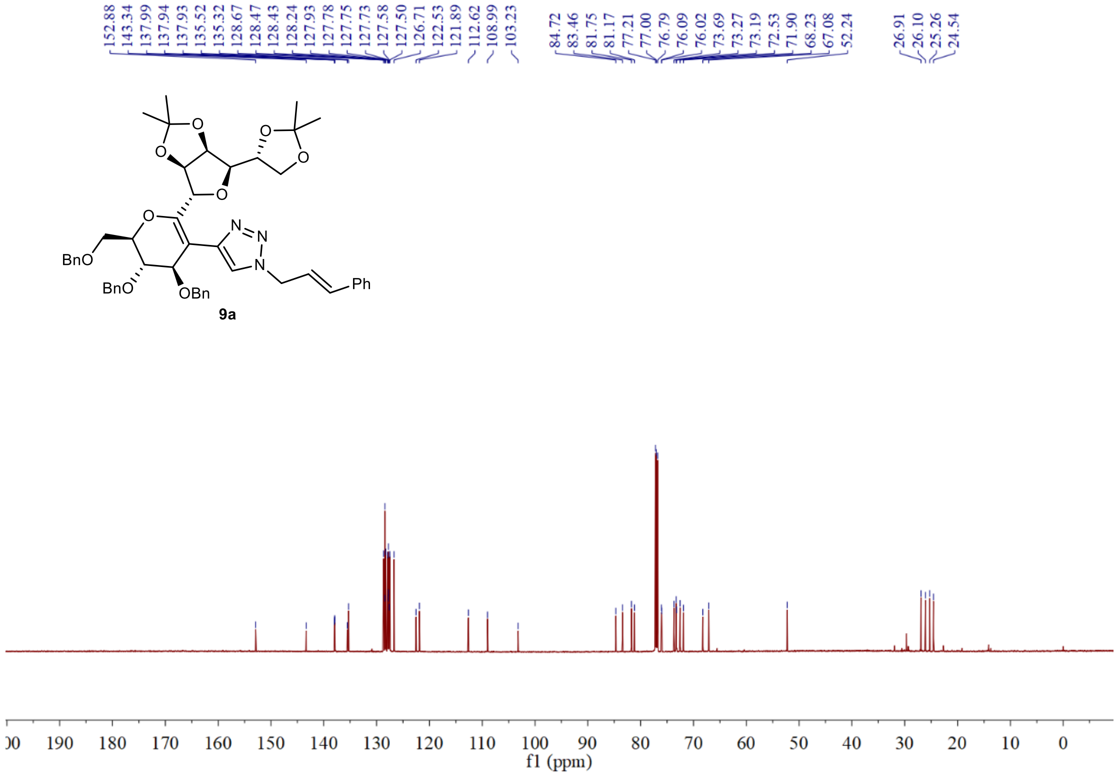

Supplementary Figure 146 ^{13}C spectra of (151 MHz, $CDCl_3$) compound **9a**

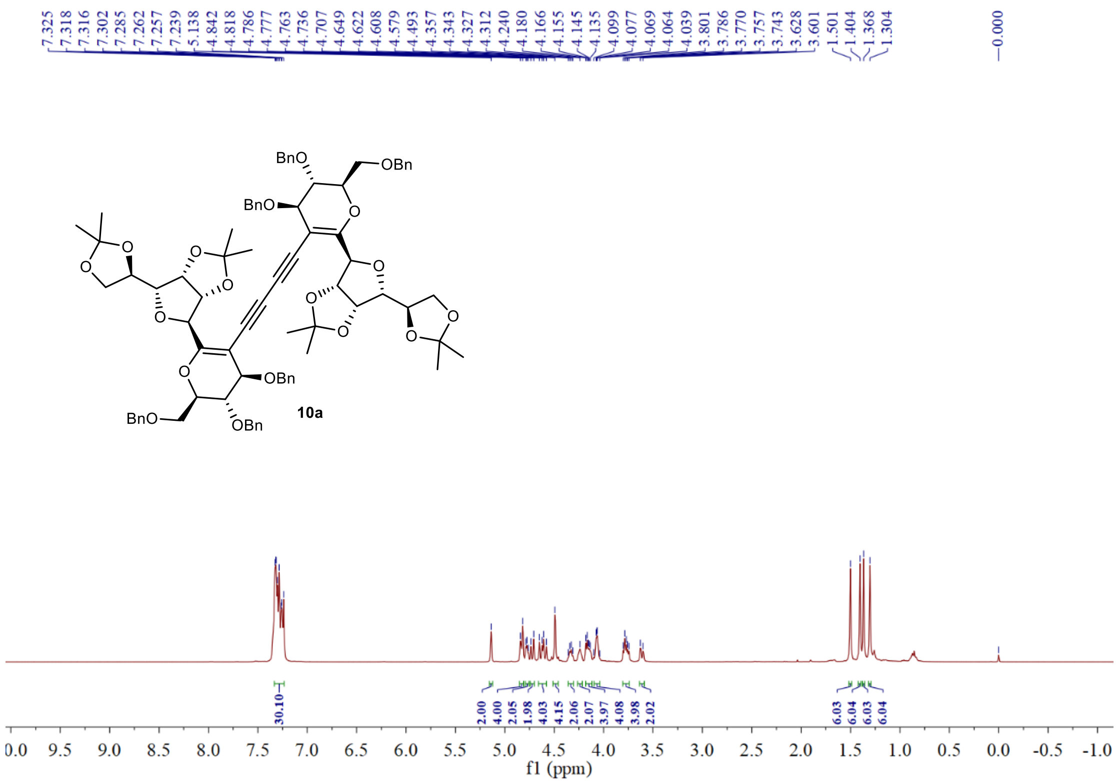

Supplementary Figure 147 ^{1}H NMR spectra of (400 MHz, $CDCl_3$) compound **10a**

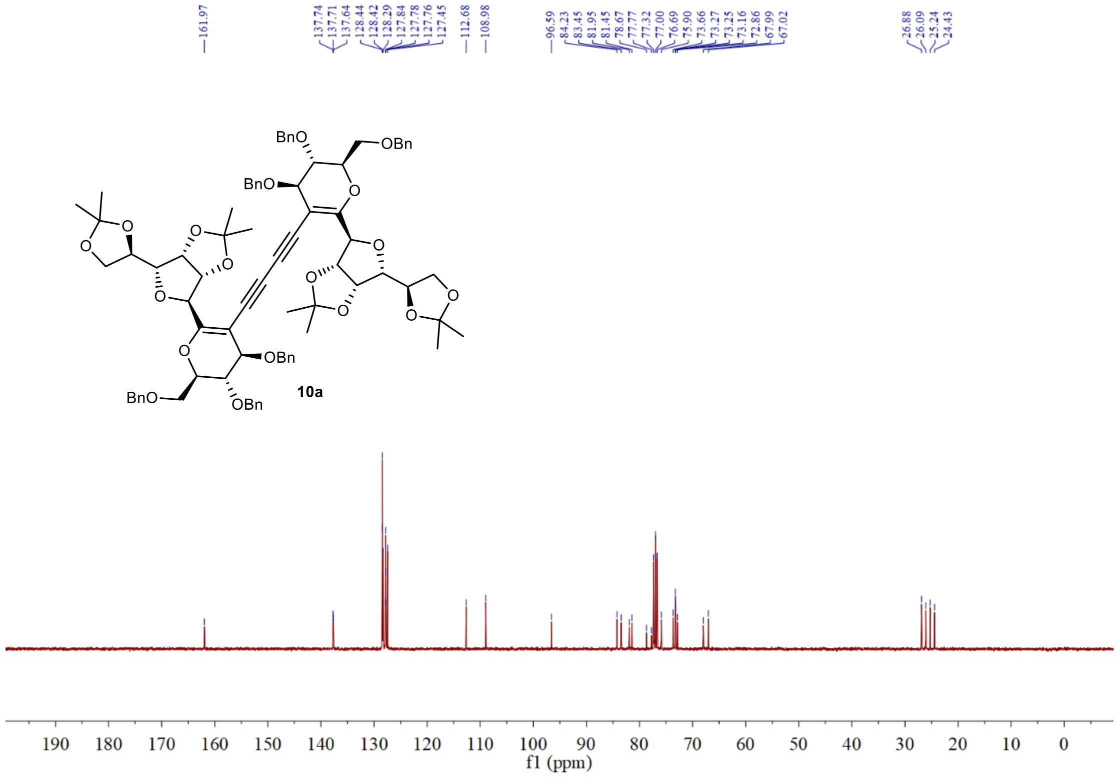

Supplementary Figure 148 ^{13}C spectra of (101 MHz, $CDCl_3$) compound **10a**

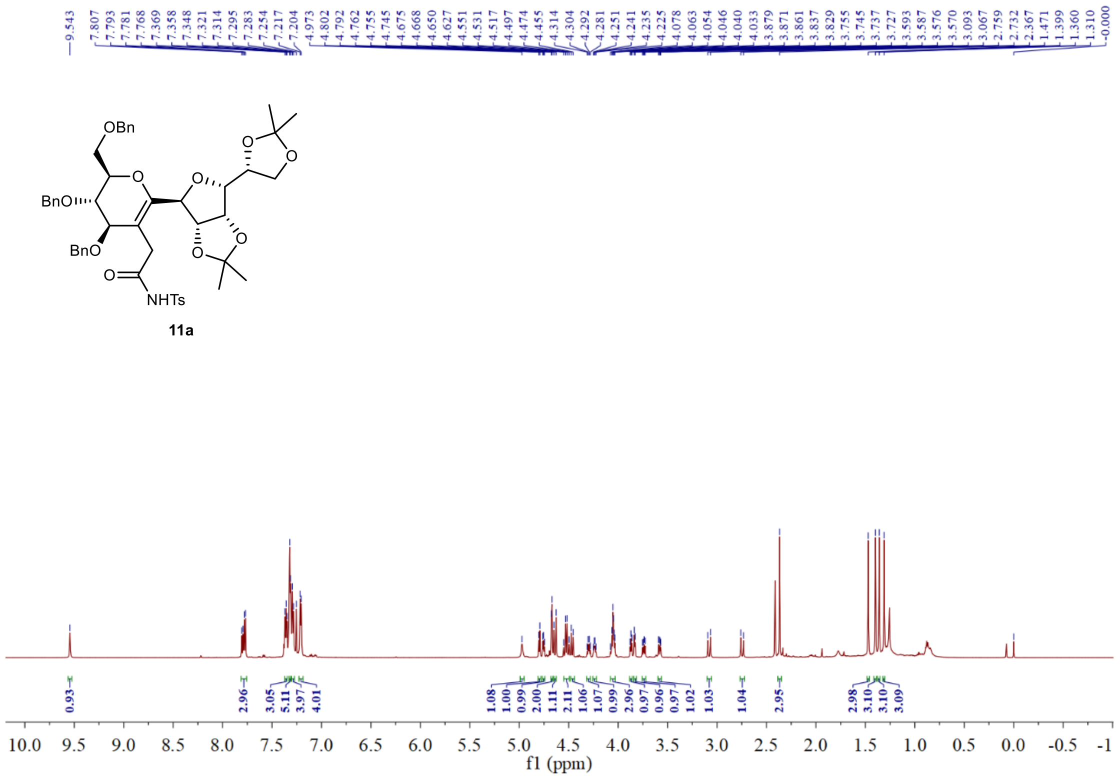

Supplementary Figure 149 ^{1}H NMR spectra of (600 MHz, $CDCl_3$) compound **11a**

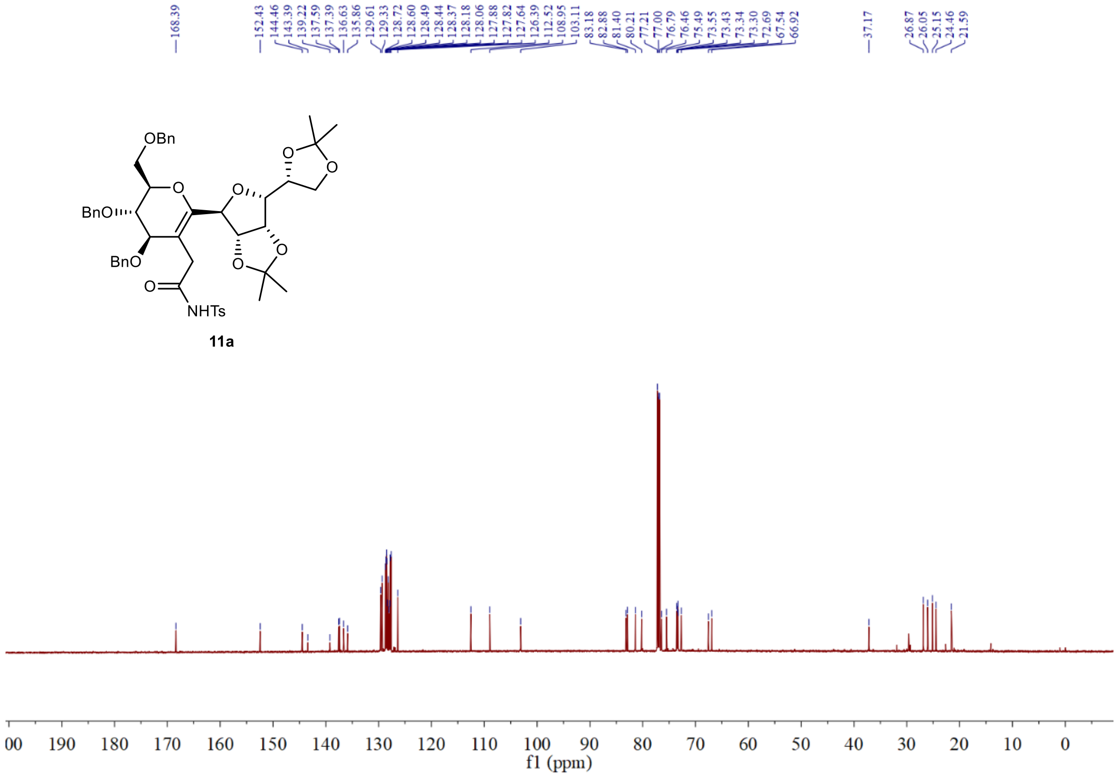

Supplementary Figure 150 ^{13}C spectra of (151 MHz, $CDCl_3$) compound **11a**

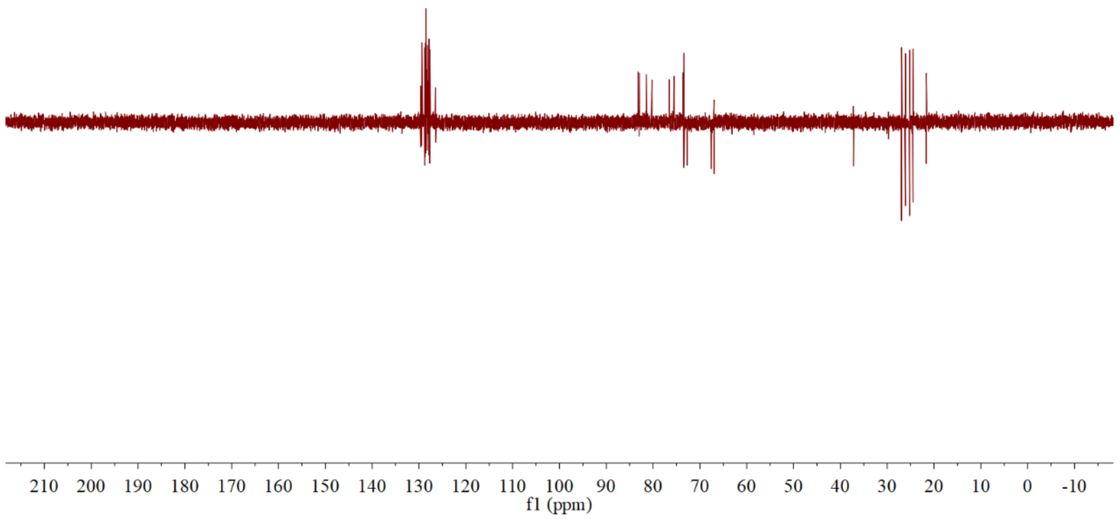

Supplementary Figure 151 DEPT spectra of compound **11a**

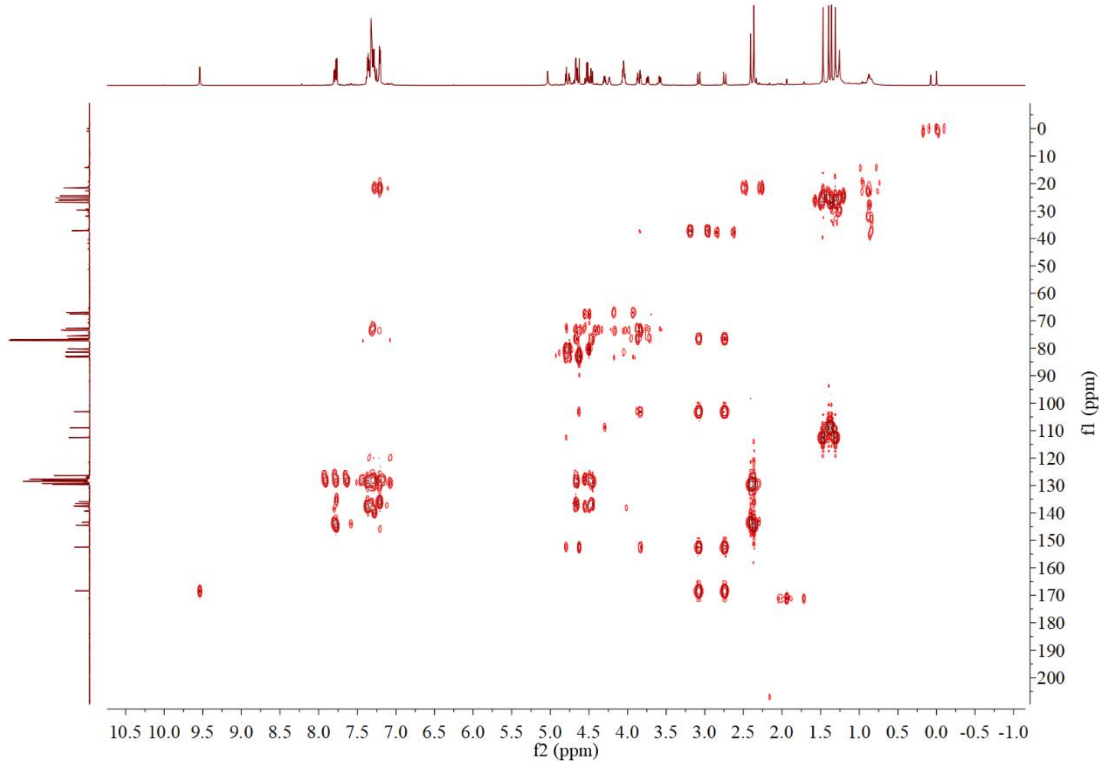

Supplementary Figure 152 HMBC spectra of compound **11a**

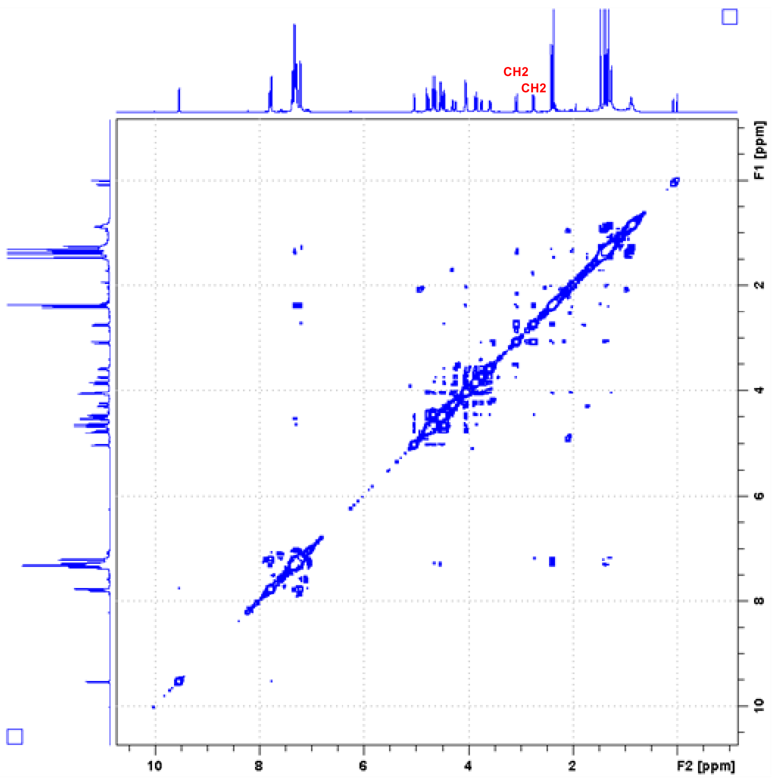

Supplementary Figure 153 COSY spectra of compound **11a**

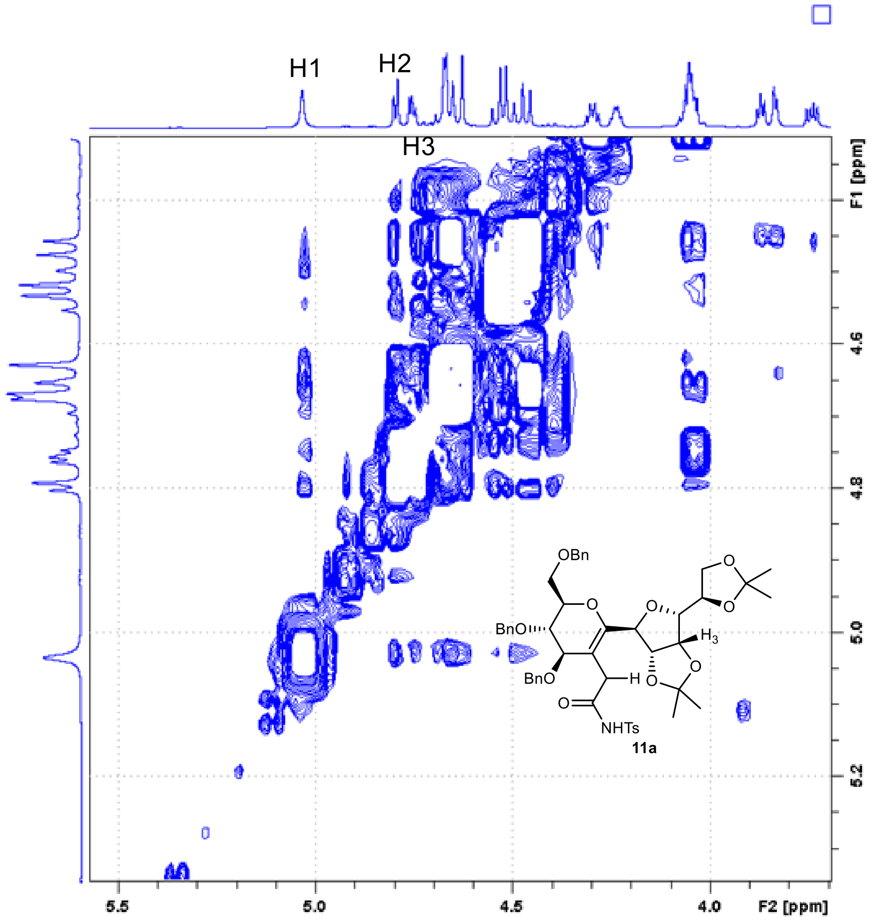

Supplementary Figure 154 COSY spectra of compound **11a**

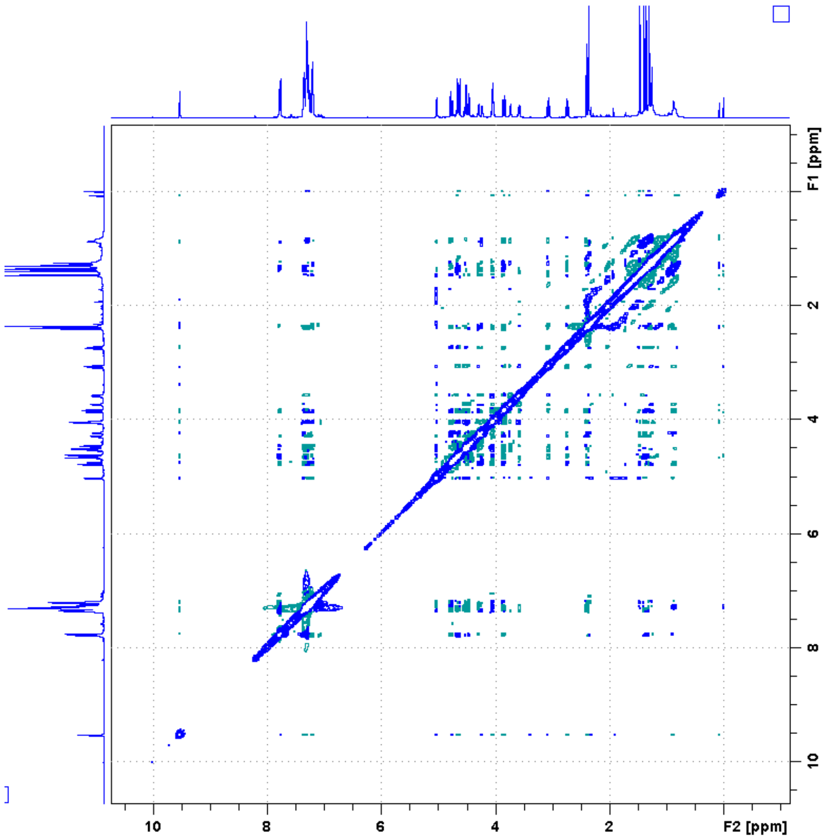

Supplementary Figure 155 NOESY spectra of compound **11a**

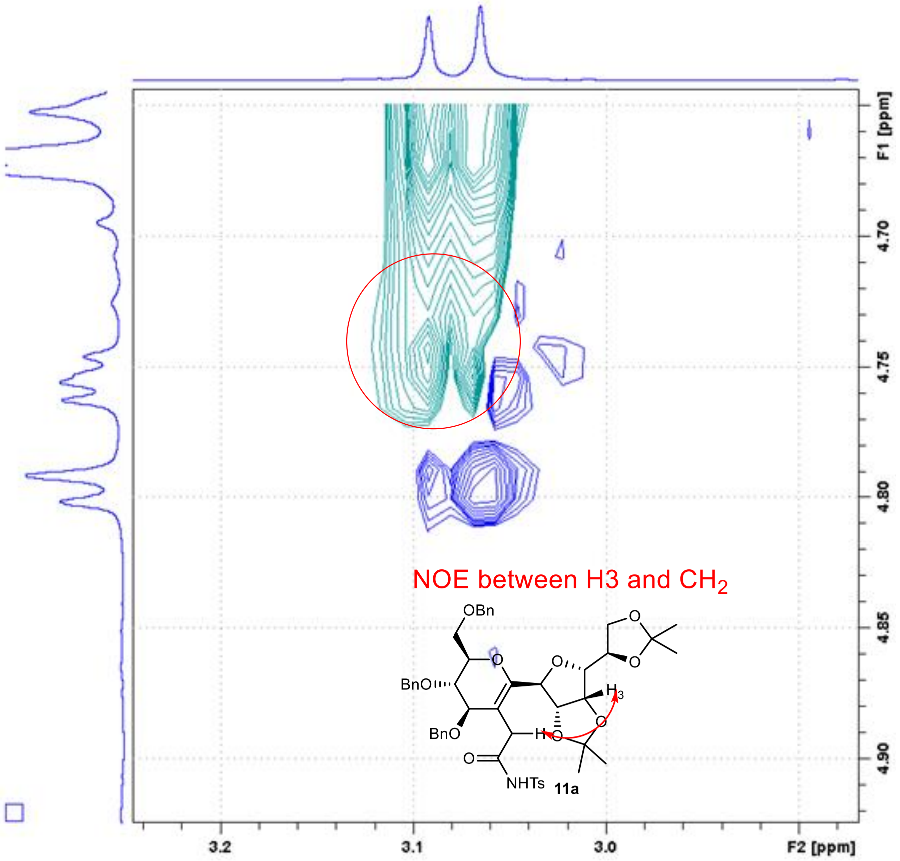

Supplementary Figure 156 NOESY spectra of compound **11a**

III. Supplementary References

1. Esteves, H. A., Darbem, M. P., Pimenta, D. C. & Stefani, H. A. Carbonylative Negishi-Type Coupling of 2-Iodoglycals with Alkyl and Aryl Halides. *Eur. J. Org. Chem.* **2019**, 7384-7388 (2019).
2. De Robichon, M., Branquet, D., Uziel, J., Lubin-Germain, N. & Ferry, A. Directed Nickel-Catalyzed pseudo-Anomeric C−H Alkynylation of Glycals as an Approach towards C-Glycoconjugate Synthesis. *Adv. Synth. Catal.* **363**, 5138-5148 (2021).
3. Malinowski, M., Van Tran, T., De Robichon, M., Lubin-Germain, N. & Ferry, A. Mild Palladium-Catalyzed Cyanation of Unprotected 2-Iodoglycals in Aqueous Media as Versatile Tool to Access Diverse C2-Glycoanalogues. *Adv. Synth. Catal.* **362**, 1184-1189 (2020).
4. Shi, W.-Y. et al. Highly regioselective and stereoselective synthesis of C-Aryl glycosides via nickel-catalyzed ortho-C–H glycosylation of 8-aminoquinoline benzamides. *Chem. Commun.* **57**, 8945-8948 (2021).
5. An, Y. et al. Palladium-catalyzed C–H glycosylation and retro Diels–Alder tandem reaction via structurally modified norbornadienes (smNBDs). *Chem. Sci.* **12**, 13144-13150 (2021).
6. Wang, Q. et al. Palladium-catalysed C−H glycosylation for synthesis of C-aryl glycosides. *Nat. Catal.* **2**, 793-800 (2019).
7. Gómez, A. M., Pedregosa, A., Casillas, M., Uriel, C. & López, J. C. Synthesis of C-1 Alkyl and Aryl Glycals from Pyranosyl or Furanosyl Chlorides by Treatment with Organolithium Reagents. *Eur. J. Org. Chem.* **2009**, 3579-3588 (2009).
8. An, S. et al. Palladium-Catalyzed O- and N-Glycosylation with Glycosyl Chlorides. *CCS Chem.* **3**, 1821-1829 (2020).
9. Zhao, G. et al. Nickel-Catalyzed Radical Migratory Coupling Enables C-2 Arylation of Carbohydrates. *J. Am. Chem. Soc.* **143**, 8590-8596 (2021).
10. Zhao, G., Yao, W., Mauro, J. N. & Ngai, M.-Y. Excited-State Palladium-Catalyzed 1,2-Spin-Center Shift Enables Selective C-2 Reduction, Deuteration, and Iodination of Carbohydrates. *J. Am. Chem. Soc.* **143**, 1728-1734 (2021).
11. Yao, W. et al. Excited-State Palladium-Catalyzed Radical Migratory Mizoroki–Heck Reaction Enables C2-Alkenylation of Carbohydrates. *J. Am. Chem. Soc.* **144**, 3353-3359

(2022).

12. Liu, S. et al. Late-Stage Macrocyclization of Bioactive Peptides with Internal Oxazole Motifs via Palladium-Catalyzed C–H Olefination. *Org. Lett.* **23**, 2933-2937 (2021).

13. Dahiya, A., Schoetz, M. D. & Schoenebeck, F. Orthogonal Olefination with Organogermanes. *Angew. Chem. Int. Ed.* **62**, e202310380 (2023).

14. Becke, A. D. Density-functional thermochemistry. III. The role of exact exchange. *J. Chem. Phys.* **98**, 5648-5652 (1993).

15. Lee, C., Yang, W. & Parr, R. G. Development of the Colle-Salvetti correlation-energy formula into a functional of the electron density. *Phys. Rev B.* **37**, 785-789 (1988).

16. Scalmani, G. & Frisch, M. J. Continuous surface charge polarizable continuum models of solvation. I. General formalism. *J. Chem. Phys.* **132**, 114110 (2010).

17. Grimme, S., Antony, J., Ehrlich, S. & Krieg, H. A consistent and accurate ab initio parametrization of density functional dispersion correction (DFT-D) for the 94 elements H-Pu. *J. Chem. Phys.* **132**, 154104 (2010).

18. Frisch, M. J. et al. Gaussian 09, Revision D.01, Wallingford CT: Gaussian, Inc. (2013).

19. Petersson, G. A. et al. A complete basis set model chemistry. I. The total energies of closed-shell atoms and hydrides of the first-row elements. *J. Chem. Phys.* **89**, 2193-2218 (1988).

20. Hay, P. J. & Wadt, W. R. Ab initio effective core potentials for molecular calculations. Potentials for the transition metal atoms Sc to Hg. *J. Chem. Phys.* **82**, 270-283 (1985).

21. Weigend, F. & Ahlrichs, R. Balanced basis sets of split valence, triple zeta valence and quadruple zeta valence quality for H to Rn: Design and assessment of accuracy. *Phys. Chem. Chem. Phys.* **7**, 3297-3305 (2005).

22. Weigend, F. Accurate Coulomb-fitting basis sets for H to Rn. *Phys. Chem. Chem. Phys.* **8**, 1057-1065 (2006).

23. Hellweg, A., Hättig, C., Höfener, S. & Klopper, W. Optimized accurate auxiliary basis sets for RI-MP2 and RI-CC2 calculations for the atoms Rb to Rn. *Theor. Chem. Acc.* **117**, 587-597 (2007).

24. Neese, F. Software update: the ORCA program system, version 4.0. *WIREs Comput. Mol.*

Sci. **8**, e1327 (2018).

25. Lu, T. & Chen, Q. Shermo: A general code for calculating molecular thermochemistry properties. *Comput. Theor. Chem.* **1200**, 113249 (2021).
26. Ho, J., Klamt, A. & Coote, M. L. Comment on the Correct Use of Continuum Solvent Models. *J. Phys. Chem. A*. **114**, 13442-13444 (2010).
27. Wang, Q. et al. Total Synthesis of C-α-Mannosyl Tryptophan via Palladium-Catalyzed C–H Glycosylation. *CCS Chem.* **3**, 1729-1736 (2020).
28. Wang, J., Zhou, Y., Xu, X., Liu, P. & Dong, G. Entry to 1,2,3,4-Tetrasubstituted Arenes through Addressing the "Meta Constraint" in the Palladium/Norbornene Catalysis. *J. Am. Chem. Soc.* **142**, 3050-3059 (2020).
29. Li, R. & Dong, G. Structurally Modified Norbornenes: A Key Factor to Modulate Reaction Selectivity in the Palladium/Norbornene Cooperative Catalysis. *J. Am. Chem. Soc.* **142**, 17859-17875 (2020).
30. Wang, J. & Dong, G. Palladium/Norbornene Cooperative Catalysis. *Chem. Rev.* **119**, 7478-7528 (2019).
